# Hyperpyramidalized alkenes with bond orders near 1.5 as synthetic building blocks

**DOI:** 10.1038/s41557-025-02055-9

**Published:** 2026-01-21

**Authors:** Jiaming Ding, Sarah A. French, Christina A. Rivera, Arismel Tena Meza, Dominick C. Witkowski, K. N. Houk, Neil K. Garg

**Affiliations:** 1 Department of Chemistry and Biochemistry, University of California, Los Angeles, Los Angeles, CA, USA.; 2 These authors contributed equally: Jiaming Ding, Sarah A. French.

## Abstract

Alkenes typically have trigonal planar geometries at each terminus, with favourable σ- and π-bonding leading to a bond order of ~2. Here we consider unusual alkenes that possess an extreme form of geometric distortion, termed hyperpyramidalization. In a hyperpyramidalized alkene, geometries deviate remarkably from the typical trigonal planar alkene geometry, leading to weak π-bonding and abnormal alkene bond orders approaching 1.5. Cubene and 1,7-quadricyclene, first validated in 1988 and 1979, respectively, but overlooked for decades since, are the focus of the present study. We leverage their unusually weak π-bonds in cycloadditions, enabling the construction of complex scaffolds and access to previously unrealized chemical space. The origins of the unusually low bond orders were investigated using computational methods. These efforts are expected to prompt future studies of molecules that display hyperpyramidalization or atypical bond orders.

Carbon–carbon double bonds (C=C), known as alkenes, are essential functional groups in organic chemistry. It is well understood that the geometry at each alkene carbon is typically trigonal planar, as seen in ethylene (**1**), which leads to maximal *p*-orbital overlap in the alkene (Fig. 1a). However, it is also possible to deviate from this conventional trigonal planar geometry when an alkene is generated in a confined ring system^1^. The present study focuses on a specific type of geometric change known as pyramidalization^2,3^, which is exemplified in the pyramidalized depiction of ethylene **1-p**, where the alkene carbon substituents no longer reside in the typical alkene plane. Pyramidalization ultimately leads to a weaking of the π-bond and lowering of bond order to below the typical bond order near 2 (ref. 4), as will be discussed more thoroughly later in this Article.

Several examples of alkenes that display pyramidalization are shown in Fig. 1b, along with their calculated Mayer bond orders (MBOs)^5,6^. Compounds **2**–**5** are historical examples of so-called pyramidalized alkenes that have been studied as early as the 1950s^2,3^, but have escaped the attention of synthetic chemists in recent years. *trans*-Cyclooctene (**6**) also displays pyramidalization and has been valuable in bioorthogonal chemistry^7,8^. Recently, anti-Bredt olefin **7** and derivatives^9–11^, which display multiple forms of geometric distortion including pyramidalization, have emerged for use in synthesis. Compounds **2**–**7** have weakened π-bonds, as seen by the decreased alkene bond orders below 2 that we have calculated (MBOs ranging from 1.68 to 1.91; see the Supplementary Information, part II, section J). Thus, this indicates a relationship between geometric distortion in the form of pyramidalization and non-integer bond orders for discrete alkenes. This is notable as non-integer alkene bond orders are most commonly associated with resonance effects based on historical studies^12^. For example, the non-integer bond order values computed for stable compounds such as s-*trans*-butadiene (**8**) and benzene (**9**) (Fig. 1c) are well known to arise from resonance (MBOs of 1.87 and 1.41, respectively; see the Supplementary Information, part II, section J).

Can discrete alkenes with even greater pyramidalization and smaller bond order values, particularly those near 1.5, be leveraged in chemical synthesis? This curiosity is notable as single, double and triple bonds are most commonly associated with integer bond orders of 1, 2 and 3, respectively, whereas such extreme weakening of a discrete bond is not yet a common consideration in synthetic chemistry. Moreover, the general use of pyramidalized alkenes in chemical synthesis has remained underdeveloped.

The present study focuses on two alkenes that display severe pyramidalization, which we describe here as hyperpyramidalization. This is exemplified by structure **1-hp** (Fig. 1d) and the two targets of interest: cubene (**10**) and 1,7-quadricyclene (**11**). While intriguing due to their unconventional geometries and unusually weak π-bonds (that is, bond orders of ~1.5), **10** and **11** were considered especially attractive targets owing to their possible use as building blocks in synthesis. Notably, rigid aliphatic frameworks have attracted considerable attention in medicinal chemistry in recent years due to their potential to improve drug-like properties and provide well-defined exit vectors^13–15^. Attractive features include increased metabolic stability^16^ due to higher C–H bond dissociation energies resulting from strain^17^, as well as improved lipophilicity and solubility^13^. Several recent studies of cubane functionalization are available, thus highlighting the interest in this burgeoning area^18–21^. As quadricyclanes have seen use in drug discovery^22^, bioorthogonal chemistry^23^ and energy storage applications^24^, new methods to synthesize substituted versions could be valuable in several applications.

Cubene (**10**) and 1,7-quadricyclene (**11**) were first studied decades ago in seminal experimental^25,26^ and computational studies^27–30^, but have been largely overlooked in modern synthesis. Key prior experimental studies of cubene include: (1) Eaton’s 1988 report of cubene generation from 1,2-diiodocubane using *tert*-butyllithium^25^, wherein a promising Diels–Alder trapping using a highly reactive diene (that is, 11,12-dimethylene-9,10-dihydro-9,10-ethanoanthracene) was reported, as well as the formation of cubane dimers that inferred the possibility of cubene intermediacy; and (2) Eaton’s 1995 synthesis of Kobayashi-type cubene precursor, which bears a trialkylsilyl group and adjacent iodide group on the cubane scaffold^31^. Using the same diene trap as in their prior studies, cubene generation was validated, providing evidence that Kobayashi-type precursors^32^ to cubene are promising. Since these seminal contributions, no further cubene experimental studies have been reported, presumably due to harsh reaction conditions used in the case of the 1988 study, the limited scope of trapping to one cycloaddition partner^25,31^ and some challenging or ‘capricious’ steps^33^ in the synthesis of Kobayashi-type cubene precursors. With regard to quadricyclene, several constitutional isomers have been considered in early reports^34^. We focus here on the 1,7-isomer, first generated by strong base-mediated dehydrohalogenation in a seminal 1979 study by Szeimies^26^. Formation of a Diels–Alder adduct with anthracene in modest yield confirmed the intermediacy of this highly distorted alkene, with subsequent reports^35,36^ demonstrating additional trapping reactions. However, the necessity of strong base and competitive formation of another isomer in some cases (that is, the 1,5-quadricyclene)^34^ have limited the scope of the early studies and the practical use of **11** in synthesis. A general, mild approach to 1,7-quadricyclene (**11**) has not been reported.

Here, we describe studies of cubene (**10**) and 1,7-quadricyclene (**11**) (Fig. 1d), including the development of their practical synthetic methods and computational investigations of these unusual strained intermediates. We report access to suitable Kobayashi-type precursors, as well as the generation and in situ trapping of the desired hyperpyramidalized alkenes in cycloadditions. Reactions proceed under mild conditions and provide access to unusual structures typically bearing four highly substituted contiguous carbon centres within a rigid, three-dimensional framework (that is, **12** or **13**). Moreover, we demonstrate the further elaboration of cycloadducts to give compounds with considerable structural complexity, including heterodimer **14** containing both the cubane and quadricyclane motifs. The geometric distortion parameters that ultimately lead to the low bond orders for cubene (**10**) and 1,7-quadricyclene (**11**), which are largely attributed to hyperpyramidalization of the alkene termini, are discussed. Overall, our discoveries provide a simple means to access exceedingly intricate structures of potential value for medicinal chemistry and other applications^13–15,18–24^, while also inspiring the future design and strategic manipulation of new intermediates that display hyperpyramidalization or non-integer bond orders in chemical synthesis.

## Results and discussion

### Structural analyses

The caged structures of cubene (**10**) and 1,7-quadricyclene (**11**) are severely distorted from conventional alkene geometries, which, in turn, leads to unusual properties. As prior ab initio calculations of cubene (**10**)^27,28^ and 1,7-quadricyclene (**11**)^29,30^ structure and properties were reported decades ago using the methods available at the time, we performed calculations on these compounds using modern levels of theory. More specifically, geometry-optimized structures, MBOs, olefin strain energies and diradical characters were computed using appropriate levels of theory (Supplementary Information, part II, sections B–D, J and K).

Several properties are notable (Fig. 2a). Both cubene (**10**) and 1,7-quadricyclene (**11**) are highly strained, possessing calculated olefinic strain energies of approximately 50 and 66 kcal mol^−1^, respectively. Confinement of the π-bond within the caged structures leads to severe geometric distortions associated with the alkenes in the form of bending and pyramidalization. More specifically, in cubene, the angles around the alkene termini are 93°, 93° and 99°, deviating substantially from typical alkene geometry (that is, 120° bond angles). These angles are a result of the rigid cubane framework. Correspondingly, rather than being trigonal planar, the alkene termini are pyramidalized to a severe extent. The pyramidalization angle as defined by Borden (*Φ*_p(Borden)_)^37^, which is applicable for alkenes with C_2V_ symmetry, is calculated for cubene (**10**) to be 85.2°. This parameter shows the remarkable extent by which the positioning of the two alkene substituents deviate from what is typically observed in a planar alkene. We also calculated the pyramidalization angle (*Φ*_p_) of **10** to be 31.6° as defined by Haddon’s π-orthogonal average vector theory^38^, which is generally useful for any given pyramidalized alkene carbon. This is an unusually high Haddon pyramidalization angle, and we propose that alkenes with severely pyramidalized termini—where the average *Φ*_p_ of the alkene termini exceeds 20°—can be classified as hyperpyramidalized alkenes. Similar hyperpyramidalization is present in the geometry-optimized structure of 1,7-quadricyclene (**11**), which has bond angles of 63°, 93° and 112°, reminiscent of the parent quadricyclane. The Haddon pyramidalization angles (*Φ*_p_) at the alkene termini are 33.2°, reflective of even greater pyramidalization in **11** compared with **10**. The overall geometric distortion leads to both cubene and quadricyclene having substantial diradical character (*y*_0_) of 14% and 13%, respectively, rendering them diradicaloids^39,40^. These values are purely theoretical, based on configuration interaction calculations on the ground states (rather than transition states). These diradical characters are notably larger than the diradical characters of non-distorted alkenes (7% calculated for **1**) and are related to the higher reactivity for **10** and **11** compared with typical alkenes. Lastly, we highlight the C=C bond lengths and the MBOs^5^ of these unusual tetra-substituted alkenes. The alkene bond lengths in **10** and **11** are 1.38 Å and 1.35 Å, respectively, slightly longer than the standard 1.33–1.34 Å alkene bond length. However, the MBOs are calculated to be approximately 1.59 and 1.55 for **10** and **11**, respectively, opposite to what would be expected based on the pioneering empirical model of bond order described by Pauling^41,42^. This reflects the unusual nature of these weak, non-conjugated π-bonds, which will be further discussed later in this Article.

### Precursor synthesis

With the aim of generating cubene (**10**) and quadricyclene (**11**) under mild reaction conditions, which, in turn, could enable broad use in synthesis, we sought to prepare Kobayashi-type precursors to these strained intermediates. Kobayashi precursors, typically characterized by the presence of a silyl substituent adjacent to a good leaving group (that is, sulfonates or halides) have become the go-to substrates for accessing strained intermediates^32^. The exact choice of silyl substituent and leaving group for a given Kobayashi precursor typically stems from a combination of factors including synthetic accessibility, stability and efficiency of strained intermediate generation as observed empirically. Although Kobayashi precursors typically require multistep synthesis, they allow efficient strained intermediate generation and trapping under mild, robust and user-friendly reaction conditions.

The synthesis of silyl iodide **17**, the precursor to cubene (**10**), was accomplished using Eaton’s strategy^31^ with modified protocols (Fig. 2b). Beginning from readily available amide **15**, which was purchased or can be made from the corresponding carboxylic acid^33^, β-silylation was achieved using a two-step sequence involving: (1) metallation and iodination with *N*-iodosuccinimide (NIS); and (2) halogen–metal exchange and quenching with trimethylsilyl chloride. Next, the amide was elaborated into the required leaving group through a three-step sequence involving reduction of the amide, oxidation to the carboxylic acid, and Barton decarboxylation, ultimately affording silyl iodide **17**. Although efforts to improve the route efficiency (for example, direct silylation of amide **15**) were unsuccessful, the current optimized protocols allow access to cubene precursor **17** in a reproducible and scalable fashion.

As a Kobayashi precursor to 1,7-quadricyclene (**11**) was not known, we developed the scalable route to silyl triflate **20** shown in Fig. 2c. It begins from readily available known bromo-ketal **18**, which is easily synthesized from commercially available bicyclo[2.2.1]hetp-5-en-2-one^43^. Halogen–metal exchange, followed by quenching with triethylsilyl chloride, enabled the introduction of the required silicon substituent. Treatment of the silylated intermediate with HCl gave rise to ketone **19** in 92% yield. Subsequent triflation provided a norbornadiene intermediate, which then underwent photochemical (2 + 2) cycloaddition^44^ to construct the quadricyclane core and furnish Kobayashi precursor **20**.

### Cubene generation, trapping and elaborations

With access to the Kobayashi precursors, we commenced experimental studies on these strained intermediates, beginning with efforts to generate and trap cubene (**10**) in Diels–Alder reactions (Fig. 3a). As noted earlier, Eaton had shown one example of a presumed Diels–Alder trapping^31^ using 11,12-dimethylene-9,10-dihydro-9,10-ethanoanthracene, yet the corresponding use of Kobayashi precursors to cubene (**10**) in Diels–Alder chemistry with other dienes has not been reported. Thus, we examined the reaction of silyl iodide **17** with a fluoride source in the presence of trapping agents to give cycloadducts (**12**). The fluoride source, stoichiometry, solvent, additives and temperature were varied, ultimately leading to the identification of optimal reaction conditions that involved the use of tetrabutylammonium fluoride (Bu_4_NF) in tetrahydrofuran (THF) at 40 °C. Under these mild conditions, precursor **17** was typically fully consumed.

As shown in Fig. 3a, our optimal conditions were amenable to the use of a variety of electron-rich dienes as trapping agents. The use of anthracenes **21** and **23** gave their respective cycloadducts **22** and **24**, in excellent yields (entries 1 and 2, respectively). As will be discussed later in more detail, it should be noted that these and other products made via this methodology have unusually complex structures, bearing four consecutive highly substituted carbon atoms (3° or 4°). We also examined the use of diphenylisobenzofuran (**25**) and furan **27**, which furnished oxygenated cycloadducts **26** and **28**, respectively (entries 3 and 4, respectively). Moreover, when pyrroles **29** and **31** were used, the respective azabicycles **30** (entry 5) and **32** (entry 6) were obtained. When electron-poor cyclopentadiene **33** was utilized (entry 7), the formation of adduct **34** was achieved, probably via an inverse electron-demand Diels–Alder cycloaddition^45,46^. Use of other Diels–Alder trapping agents, such as tropolone **35** and exocyclic diene **37**, gave the respective adducts **36** and **38** (entries 8 and 9, respectively).

The methodology permits access to several other exotic structures, as shown in Fig. 3b–e. Using 9-bromoanthracene as a diene trap, followed by treatment of the resulting Diels–Alder cycloadduct with silica gel, facilitated a dyotropic rearrangement^47^, ultimately yielding homocubane **39** in 57% yield (Fig. 3b). X-ray crystallography was used to corroborate this atypical structure. Moreover, this example highlights the privileged ability of cubanes to undergo unique rearrangements^48^ to access related caged moieties. We were also delighted to find that other types of cycloaddition were possible. Use of oxopyridinium **40** led to [3.2.1] azabicycle **41**, which boasts an unusual scaffold bearing three consecutive fully substituted stereocentres (Fig. 3c), presumably via (5 + 2) cycloaddition^49^ of the cubene (**10**) intermediate. (2 + 2) and (3 + 2) cycloadditions were also explored, several of which were unsuccessful, presumably because of unfavourable kinetics in forming a cubane-fused [2.2.2]-propellane or five-membered ring. However, the trapping of cubene (**10**) with ene carbamate **42** (ref. 50) delivered pyrrolidine **43**, which provides a rare example of a heterocycle-fused cubane derivative^51–54^ (Fig. 3d). A related heterocycle, bearing an isomeric pyrrolidine core in comparison with **43**, was accessed using an ozonolysis strategy with reductive work-up (**32**→**44**; Fig. 3e).

### Quadricyclene generation, trapping and elaborations

**Fig. 1 |:**
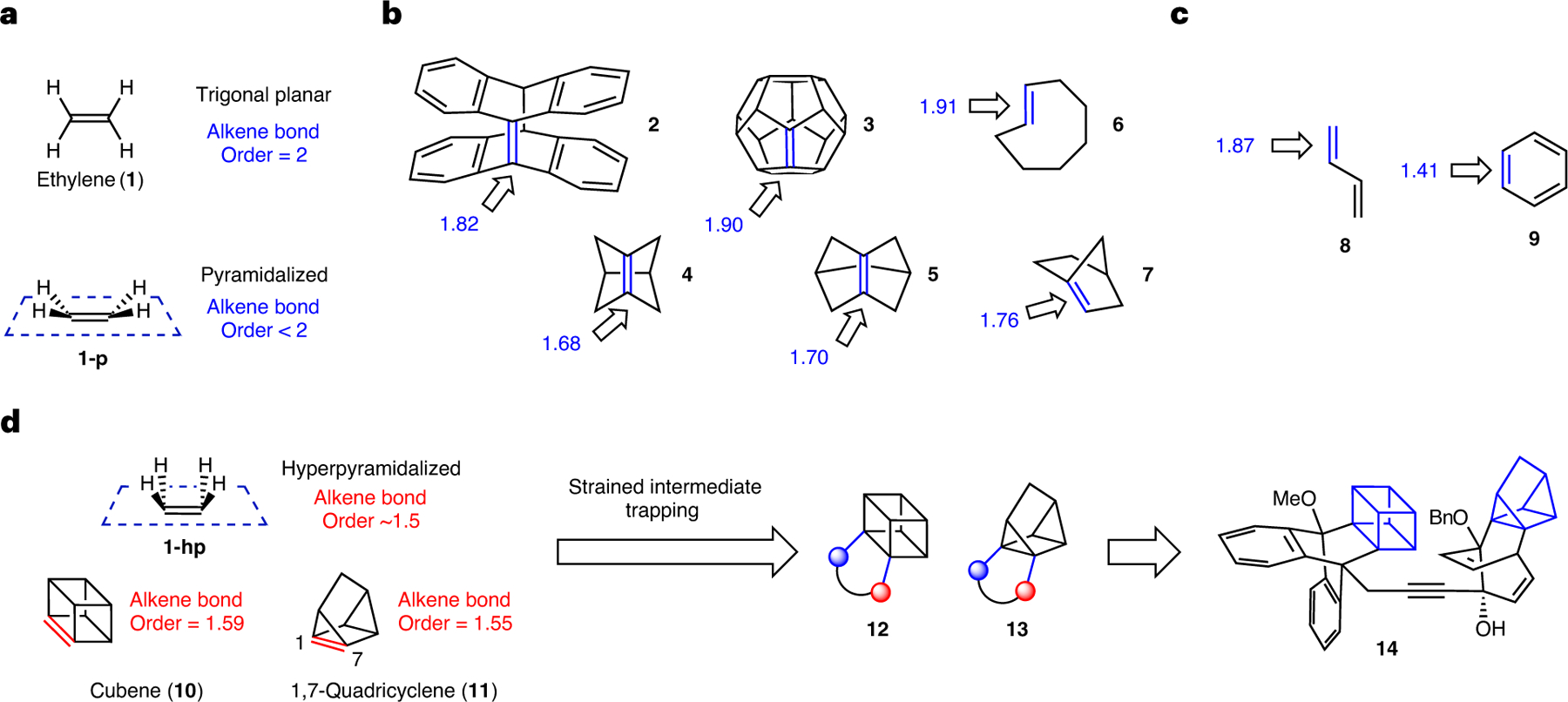
Background and overview. **a**, Geometry and bond order of typical ethylene (**1**), and reduction of alkene bond order by pyramidalization of carbon termini (**1-p**). **b**, Pyramidalized alkenes (**2**–**7**) with non-integer bond orders, wherein there is little or no resonance stabilization. **c**, Non-integer alkene bond orders as a result of resonance effects, as exemplified by **8** and **9**. **d**, Present study of cubene (**10**) and 1,7-quadricyclene (**11**), which have discrete hyperpyramidalized alkenes possessing non-integer bond orders near 1.5. The trapping and elaboration of **10** and **11** permit access to rigid, three-dimensional scaffolds. The depicted bond orders for **2**–**11** are calculated MBO values (⍵B97X-D/def2-TZVP). Me, methyl; Bn, benzyl group.

**Fig. 2 |:**
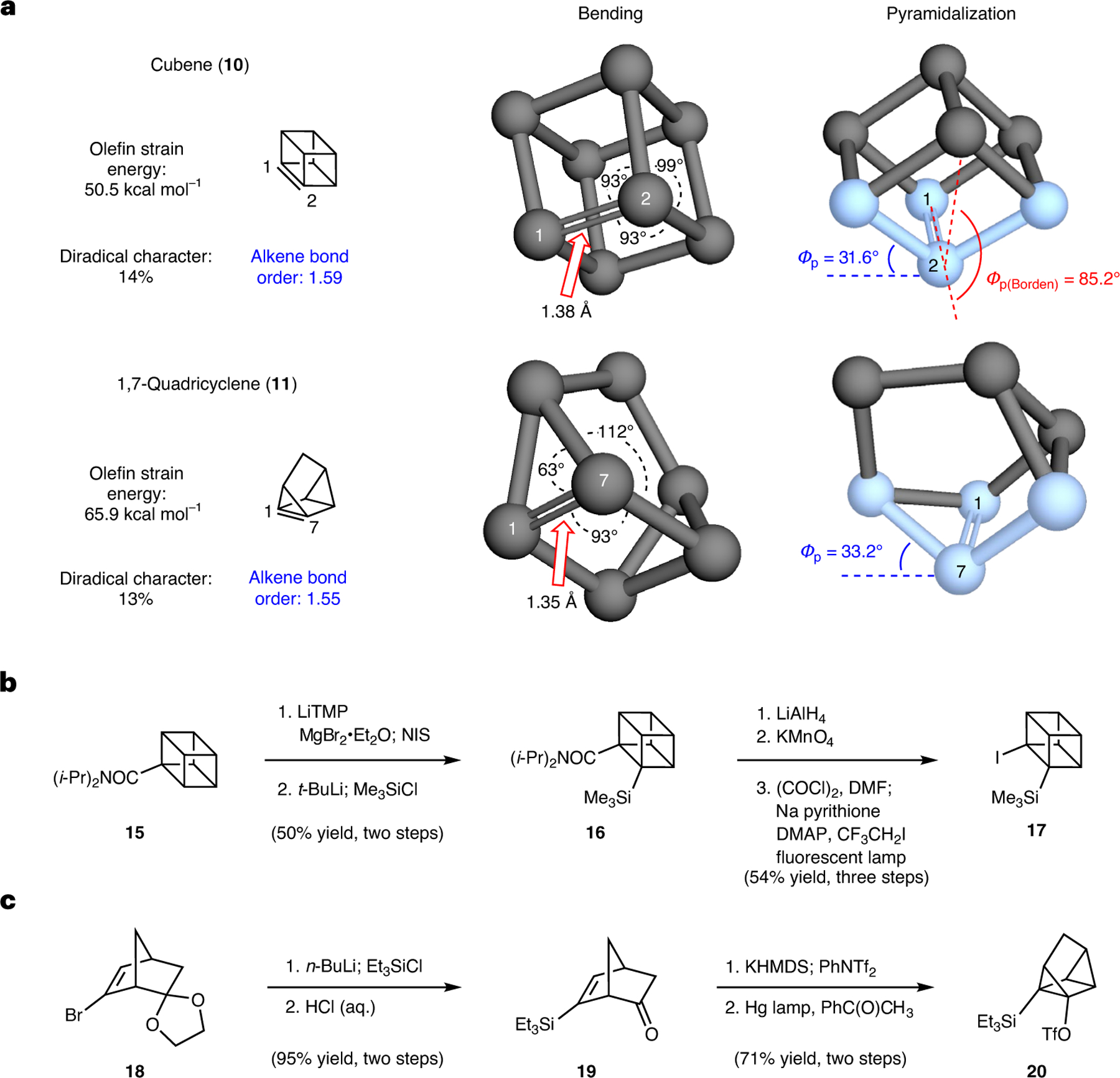
Bonding and structural properties of hyperpyramidalized alkenes cubene (10) and 1,7-quadricyclene (11) and synthetic routes to precursors 17 and 20. **a**, Structural properties of **10** and **11**. Geometry optimizations and MBOs computed at the ωB97X-D/def2-TZVP level of theory. Olefin strain energy calculated at the CCSD(T)/cc-pVTZ level of theory. Diradical character (*y*_0_) calculated at CASPT2/CASSCF(6,6)/aug-cc-pVDZ level of theory. **b**, Synthesis of cubene precursor **17** from amide **15** based on Eaton’s seminal route. **c**, Synthesis of 1,7-quadricyclene precursor **20** from ketal **18**. *Φ*_p_, pyramidalization angle; TMP, 2,2,6,6-tetramethylpiperidide; *t*-Bu, *tert*-butyl; *i*-Pr, *iso*-propyl; DMF, *N*,*N*-dimethylformamide; DMAP, 4-dimethylaminopyridine; Bu, butyl; HMDS, hexamethyldisilazane; Ph, phenyl; Tf, trifyl; Et, ethyl.

**Fig. 3 |:**
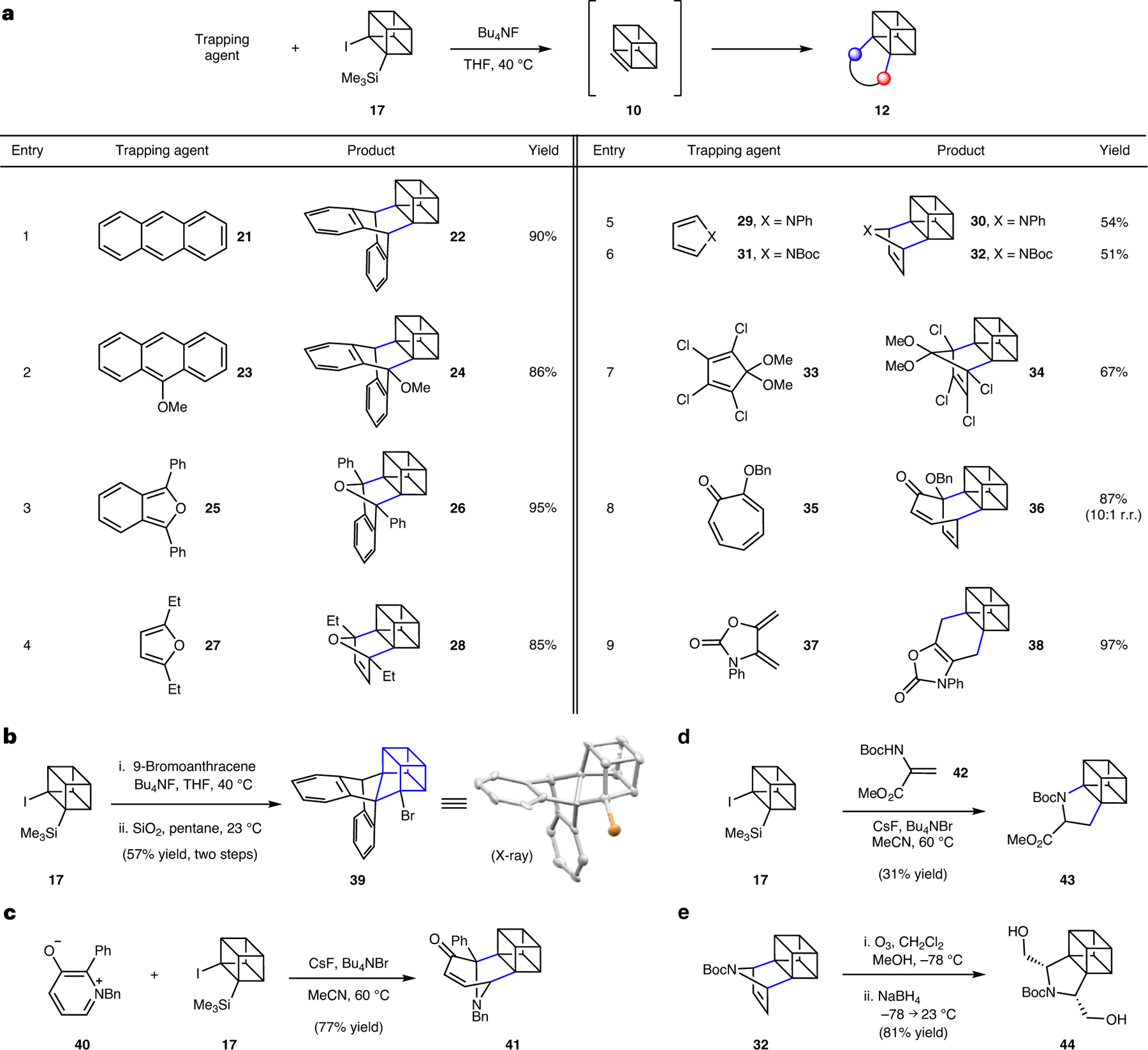
Experimental studies involving cubene (10). **a**, Conditions: **17** (1 equiv.), trapping agent (1.5–10 equiv.), Bu_4_NF (5.0 equiv.), THF (0.05 M), 40 °C, 2–71 h, sealed vessel. For entry 8, the observed regioselectivity ratio (r.r.) indicates distribution of constitutional isomers, with the major isomer formed being depicted. **b**, Access to homocubane **39** via Diels–Alder cycloaddition, followed by dyotropic rearrangement. **c**, (5 + 2) cycloaddition with oxidopyridinium **40** to furnish azabicycle **41**. **d**, (3 + 2) cycloaddition to introduce a five-membered ring fused to the cubane scaffold. **e**, Alternative approach to five-membered ring fused to cubane scaffold (that is, **44**) involving oxidative cleavage of cycloadduct **32**. Bu, butyl; Me, methyl; Ph, phenyl; Et, ethyl; Boc, butyloxycarbonyl; Bn, benzyl.

**Fig. 4 |:**
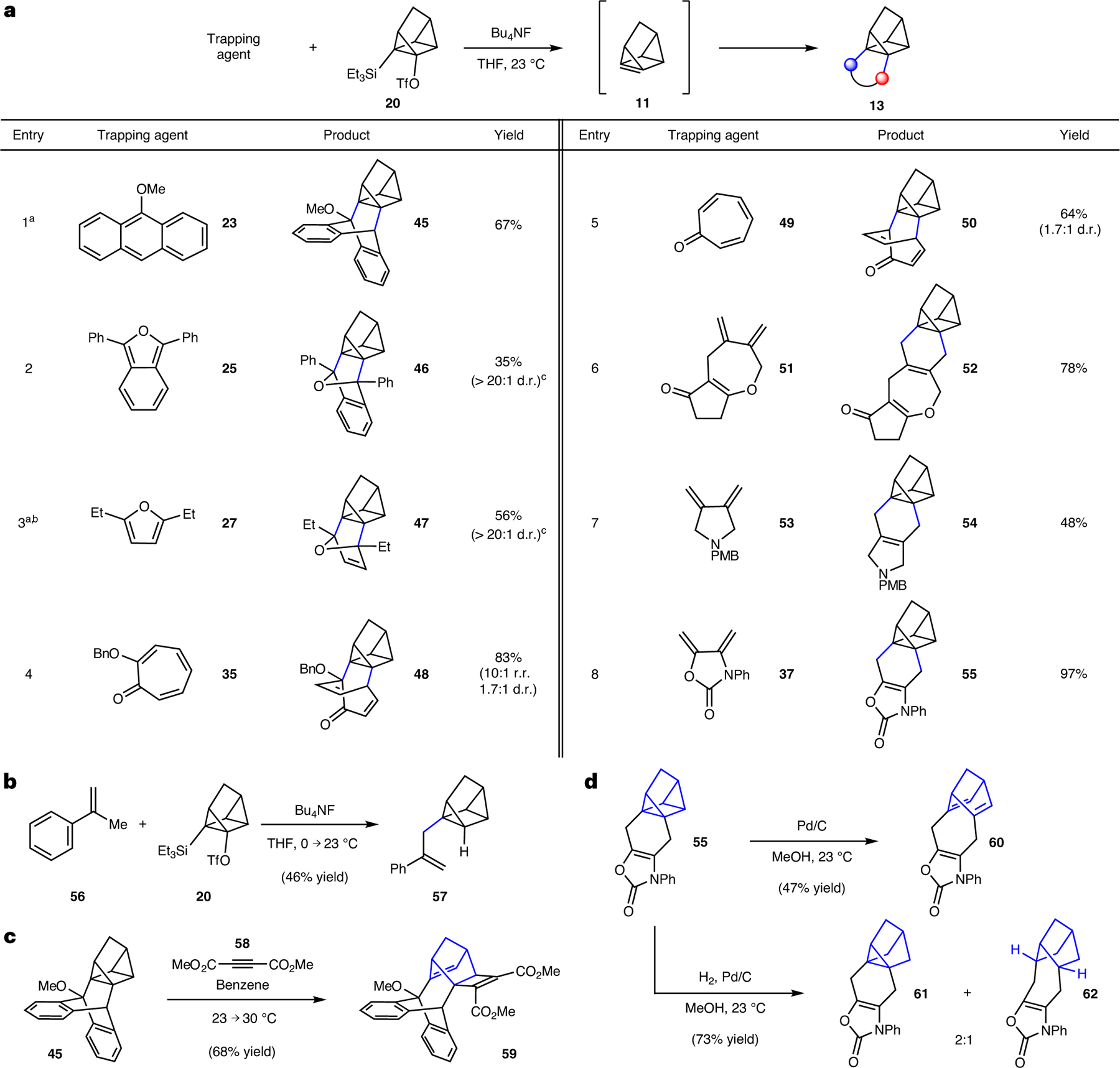
Experimental studies involving 1,7-quadricyclene (11). **a**, Conditions: **20** (1 equiv.), trapping agent (3–20 equiv.), BuN_4_F (5 equiv.), THF (0.05 M), 23 °C, 3–24 h, sealed vessel. Where applicable, observed diastereoselectivities and regioselectivities are reported as a ratio of isomers (diastereomeric ratio (d.r.) or regioisomeric ratio (r.r.), respectively), with the major isomer formed being depicted. ^a^Temperature: 0 → 23 °C. ^b^Yield determined by ^1^H NMR analysis with mesitylene as an external standard. ^c^Only the depicted diastereomer was observed likely due to instability of the other diastereomer. **b**, Ene reaction to give **56**. **c**, Elaboration of **45** to give cyclobutenyl-fused norbornene **59**. **d**, Pd/C mediated isomerization of adduct **55** to bridged norbornadiene **60** and hydrogenolysis of **55** to furnish **61** and **62**. Bu, butyl; Me, methyl; Ph, phenyl; Et, ethyl; PMB, *p*-methoxybenzyl; Bn, benzyl; Tf, triflyl.

As shown in Fig. 4a, 1,7-quadricyclene (**11**) generation and Diels–Alder trapping experiments were also performed. Similar to our studies of cubene (Fig. 3a), silyl triflate **20** was treated with fluoride in the presence of a diene trapping agent to give cycloadducts **13**. Through experimentation, we found that the use of Bu_4_NF in THF was generally optimal, although the preferred temperature in our studies of **11** was deemed to be 23 °C (versus 40 °C in our studies of cubene). Stoichiometry and reaction times were empirically optimized for each entry.

We were delighted to find that the trapping using 9-methoxyanthracene (**23**) furnished the desired cycloadduct **45**, bearing a bridged bicycle appended to the quadricyclane core (Fig. 4a, entry 1). Of note, this transformation proceeds more efficiently compared with the corresponding report by Szeimies^36^ (67% versus 38% yield) that utilized a dehydrohalogenation approach with a strong base, rather than the mild Kobayashi-type strategy shown herein. Trapping of furan derivatives **25** and **27** gave rise to oxabicycles **46** and **47**, respectively, with only *endo* products being observed (entries 2 and 3). Moreover, the use of tropolone **35** or tropone **49** afforded bicyclic enones **48** (entry 6) or **50** (entry 7), respectively, in good yields and moderate diastereoselectivities. Lastly, exocyclic dienes **51**, **53** and **37** were examined, each possessing a different heterocyclic framework (that is, tetrahydrooxepine, pyrrolidine or oxazolidinone, respectively) as shown in entries 6–8. The use of these dienes in 1,7-quadricyclene (**11**) trapping experiments provided fused cycloadducts **52**, **54** and **55**, respectively.

Other experiments involving quadricyclene or cycloadducts were performed, with key results shown in Fig. 4b–d. The Alder-ene reaction^55^ proceeded readily using **56** to furnish quadricyclane derivative **57**, which is adorned with a styrene-containing sidechain (Fig. 4b). This result demonstrates the viability of a group-transfer pericyclic reaction. Attempts to achieve other types of cycloaddition, such as (3 + 2) or (2 + 2) reactions, proved challenging, presumably due to disfavourable reaction kinetics, the instability of potential adducts due to their strain, or a combination thereof. Nonetheless, we pursued alternative approaches to access unique compounds by modifying the highly strained quadricyclane scaffold^56^. As shown in Fig. 4c, reaction of cycloadduct **45** and dimethyl acetylenedicarboxylate (**58**)^36^ gave rise to norbornene **59**, bearing a cyclobutene ring, presumably via a regioselective ring-opening cycloaddition process. Other modified scaffolds were accessible using palladium catalysis, as shown in Fig. 4d. Exposure of adduct **55** to catalytic palladium on carbon (Pd/C) led to isomerization^57^ of the quadricyclane core, giving bridged norbornadiene **60**. This could also be achieved thermally, albeit using prolonged reaction times, whereas the reverse reaction occurs using ultraviolet light (Supplementary Information, part I, section G). In addition, treatment of **55** with H_2_ and Pd/C furnished notricyclane **61** and bridged norbornane **62** (2:1 ratio of separable isomers).

### Structural features

Several features of the synthetic compounds depicted in Figs. 3 and 4 should be emphasized. The products formed from cycloaddition reactions, all arising from just two common Kobayashi precursors (that is, **17** and **20**), bear vicinal substitution stemming from the rigid cubane or quadricyclane cores. This is notable given that: (1) methods to prepare cubanes and 1,7-quadricyclanes with vicinal substitution patterns are underdeveloped; (2) having the di-substitution in the form of a ring or bicycle fused to the cubane or quadricyclane framework is rare; and (3) rigid, strained three-dimensional scaffolds are of current interest in medicinal chemistry^13–15^, as discussed earlier, but can often be difficult to access. Thus, the methodology we describe provides a practical means to access new and coveted scaffolds.

With regard to the specific ring systems accessible, we show the possibility of accessing both five- and six-membered rings fused to the cubane or quadricyclane frameworks (for example, **38**, **43** and **44** in Fig. 3a and **52**, **54** and **55** in Fig. 4a). In the case of products containing bridged bicyclic ring systems, [3.2.2]-, [3.2.1]-, [2.2.2]- and [2.2.1]-ring systems can be made (see entries 1–8 in Fig. 3a, **41** in Fig. 3c and entries 1–5 in Fig. 4a). Some limitations should also be noted. For example, (2 + 2) cycloadditions to give cyclobutyl-fused products have thus far proven challenging. Similarly, additions of nucleophiles to give mono-substituted cubanes or quadricyclanes have been met with mixed results and will be reported separately in due course. Nonetheless, the structural complexity that can be generated by this methodology and derivatization is underscored by the successful modifications of cycloadducts as shown in Figs. 3b,e and 4c,d.

Finally, most products obtained possess four newly formed consecutive, highly substituted carbon centres (3° or 4°), appended to the cubane or quadricyclane cores, which themselves are also composed primarily of 3° carbons. Thus, many of the compounds contain eight to ten consecutive highly substituted carbons (3° or 4°). The low bond order and consequent high reactivity of cubene (**10**) and 1,7-quadricyclene (**11**) are essential for enabling access to the exquisite architectures shown herein.

### Geometric distortion, bond order and synthesis of heterodimer 14

As previously discussed, cubene (**10**) and 1,7-quadricyclene (**11**) are unusual alkenes that are geometrically distorted in rather remarkable ways. The two major modes of distortion associated with **10** and **11** are bending and pyramidalization, which result from their rigid caged scaffolds. The impact of geometric distortion can be assessed by various parameters, such as: (1) strain energy, or Schleyer’s olefin strain energy^58^, which provides a useful means to compare similar structures, and their reactivities based on the relationship defined by the Evans–Polanyi principle^59^, (2) delocalization, as discussed by Sterling, Anderson and Duarte^60^, and (3) diradical character, which has been used recently to explain an unusual reaction of strained cyclic allenes that plausibly proceeds through a one-electron process^40,61^. The complex interplay between these parameters is the subject of ongoing investigations and will be described in due course. Nonetheless, in the present study, we focus on the impact of geometric distortion on alkene bonding, given the unusually low bond orders of ~1.5 seen in both cubene (**10**) and 1,7-quadricyclene (**11**) and the potential to use bond order as a guiding parameter in synthetic design. We provide insight into the relationship between geometric distortions and bond order, which are ultimately related to the high reactivity of **10** and **11**.

**Fig. 5 |:**
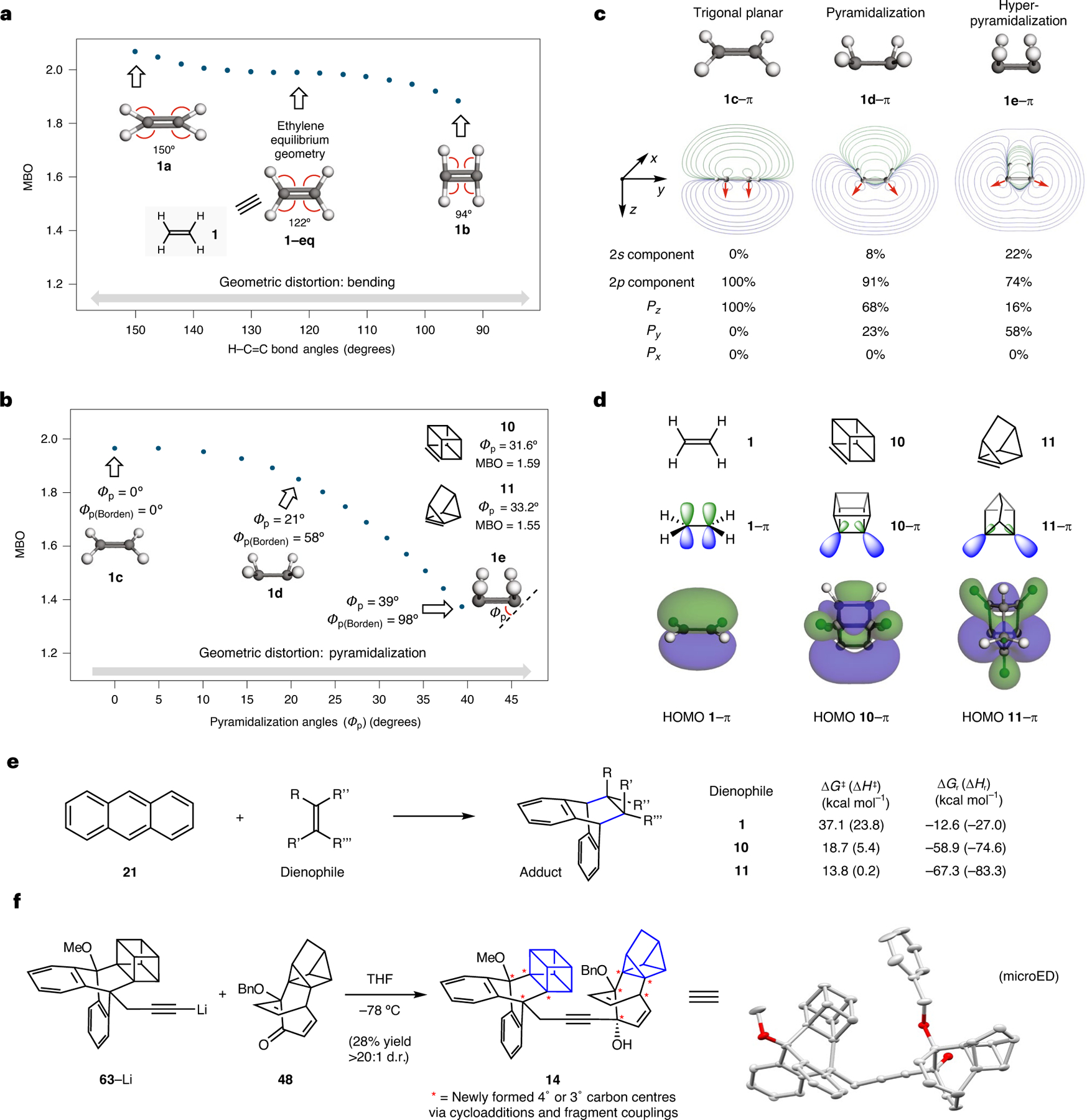
Studies pertaining to properties of geometrically distorted alkenes with low bond orders. **a**, MBO of systematically distorted ethylene at varying H–C=C bond angles to assess the effect of bending. **b**, MBO of systematically distorted ethylene at varying pyramidalization angles (*Φ*_p_). **c**, Comparison of π molecular orbital (π MO) contour plots and approximate AO composition of π MO of planar ethylene (**1c**), pyramidalized ethylene (**1d**) and hyperpyramidalized ethylene (**1e**). MO structures were obtained at the HF/6–31G(d,p) level of theory. **d**, Localized depiction of π MOs and calculated HOMOs of ethylene (**1**), cubene (**10**) and 1,7-quadricyclene (**11**). Geometry optimizations were performed at the ωB97X-D/def2-TZVP level of theory. MO structures were obtained at the HF/6–31G(d,p) level of theory. MO surfaces are plotted at an isovalue of ±0.023 a.u. **e**, Computations of the Diels–Alder reaction of anthracene **21** with either olefin **1**, **10** or **11**, performed at the ⍵B97XD/def2-TZVP/SMD(THF) level of theory. See the Supplementary Information for details. **f**, Unification of cubene and quadricyclene cycloadducts provides heterodimer **14**. Me, methyl; Bn, benzyl; microED, microcrystal electron diffraction.

Computational studies were performed using ethylene (**1**) as a model system, with geometric distortions being introduced systematically. For each distorted structure, the alkene bond order^5,6^ was calculated, with results for bending and pyramidalization summarized in Fig. 5a,b (see the Supplementary Information, part II, section I, for the MBO dependence on bond length). Figure 5a shows the bond order of systematically bent ethylene without pyramidalization. Beginning with ethylene at its equilibrium geometry (**1–eq**), the expected bond order value of roughly 2 is observed. In the distorted structure **1a**, the H–C=C bond angles are increased to ~150°, which gives a slight increase in the bond order (MBO 1.97 → 2.04). Conversely, a slight drop in the bond order is observed when the H–C=C bond angles are contracted to ~94°, as seen in **1b** (MBO 1.97 → 1.86). Overall, we conclude that bending of the H–C=C bonds, without pyramidalization, leads to only a minor change in bond order.

A much more pronounced impact on alkene bond order is observed when considering pyramidalization. As shown in Fig. 5b, both Borden (*Φ*_p(Borden)_) and Haddon (*Φ*_p_) pyramidalization angles were considered as ethylene was systematically pyramidalized and then hyperpyramidalized. Structure **1c** (*Φ*_p(Borden)_ and *Φ*_p_ = 0°) with all bond angles at 120° reflects ethylene near its equilibrium planar geometry, showing the expected alkene bond order value of ~2. Upon *syn*-pyramidalization of the two ethylene termini, the bond order drops remarkably. For example, in structure **1d** (*Φ*_p(Borden)_ ≈ 58° and *Φ*_p_ ≈ 21°), the bond order approaches 1.8. An even more drastic decrease of the alkene bond order was observed upon further increase of the pyramidalization angles. In the extreme case of hyperpyramidalized structure **1e** (*Φ*_p(Borden)_ ≈ 98° and *Φ*_p_ ≈ 39°), an alkene bond order approaching 1.4 is calculated. We have also plotted *Φ*_p_ versus bond order for other strained intermediates and observe similar correlations (Supplementary Information, part II, section I). These results generally align with the bond orders and pyramidalization angles of cubene (**10**) and 1,7-quadricyclene (**11**), suggesting that hyperpyramidalization is probably the major contributing factor to the low bond order seen in these species. We also highlight a point of contrast between resonance-stabilized alkenes and hyperpyramidalized alkenes. Although both have atypical non-integer bond orders below 2, resonance stabilization leads to increased stability, whereas hyperpyramidalization leads to decreased stability and higher reactivity.

Calculations were performed to gauge how increasing pyramidalization leads to a lowering of the alkene bond order. Orbital occupancy-perturbed MBO analysis^62^ indicates that the lowering of total alkene bond orders in pyramidalized and hyperpyramidalized alkenes is attributed to the weakening of π-bonding rather than σ-bonding (Supplementary Information, part II, section E). This is consistent with earlier studies by Borden, which suggest weakening of the π-bond in pyramidalized ethylene^37^. Indeed Borden et al. have shown that the *p* orbitals that typically constitute the π-bond in planar ethylene instead constitute the σ-bond in highly pyramidalized ethylene, resulting in a drop in CC overlap population^37^. We also studied the π-bonding in **1c**, **1d** and **1e** and carried out atomic orbital (AO) component analysis^63–65^ (Fig. 5c). The contour plots of the π molecular orbitals in alkenes **1c**–**1e** are shown. As one would expect for non-distorted ethylene with trigonal planar geometry at the alkene termini, orbital **1c**–π is composed of nearly 100% *p* orbitals, arising entirely from the *P*_*z*_ orbital contribution as defined by the *xyz* axes depiction. Upon pyramidalization as seen in **1d**–π, orbital mixing occurs^66,67^, with the resulting π-bond being composed of roughly 8% *s* character and 91% *p* character. With regard to the latter, the *P*_*z*_ contribution drops from 100% to 68%, while the *P*_*y*_ contribution increases from 0% to 23%. The increase in the *P*_*y*_ contribution is reflected in the contour map of **1d**–π and suggested by the change in directionality of the blue lobe, now tilting towards the *y* axis (see red arrows)^68^. In the case of hyperpyramidalization (see **1e**–π), the *s* character in the π-bond further increases to 22%, with a corresponding decrease in *p* orbital contribution to 74%, leading to *sp*^3^-like character. The *P*_*z*_ contribution lowers to only 16%, while the *P*_*y*_ contribution increases to 58%. The directionality of the lobes (see red arrows) is further tilted towards the *y* axis, away from the *z* axis, leading to a substantial reduction in effective hybrid orbital overlap. Overall, the studies shown in Fig. 5c demonstrate that, upon hyperpyramidalization, the substantial decrease in *P*_*z*_ character, with increases in *s* and *P*_*y*_ character, leads to an extreme lowering of the alkene bond order.

An analysis of the π molecular orbitals of cubene (**10**) and 1,7-quadricyclene (**11**) is shown in Fig. 5d. In comparison with ethylene (see **1**–π), the corresponding orbitals in cubene (**10**–π) and 1,7-quadricyclene (**11**–π) have increased *s* character due to hyperpyramidalization, as seen in pyramidalized variants of ethylene (see discussion of Fig. 5c), and are extended towards the exterior of the caged scaffold^66^. Moreover, the orbitals shown in **10** and **11** (see **10**–π and **11**–π, respectively) are no longer parallel^68^. Instead, the larger lobes are oriented away from one another outside of the caged scaffold, with the smaller lobes pointed towards each other within the cages (see **10**–π and **11**–π; also see the Supplementary Information, part II, section F & G), ultimately resulting in decreased overlap. Such orbital reorientation is most pronounced in the case of 1,7-quadricyclene (**11**), as the alkene termini are more pyramidalized in **11** compared with the alkene termini in cubene (**10**). The decrease in effective π-bonding in **10** and **11**, in comparison with ethylene (see highest occupied molecular orbital (HOMO) **1**–π), is also seen in the calculated molecular orbitals HOMO **10**–π and HOMO **11**–π. This consideration of the π molecular orbitals of **10** and **11** provides an explanation for the weak π-bonding and the consequential low alkene bond orders of the hyperpyramidalized alkene intermediates cubene (**10**) and 1,7-quadricyclene (**11**). The weakening of the alkene π-bond upon hyperpyramidalization correlates to an increase of the π HOMO energy, with a corresponding substantial lowering of the π lowest unoccupied molecular orbital (LUMO) energy, contributing to high reactivity and reaction exothermicity^27^.

Calculations were performed to assess the effect of hyperpyramidalization on transition state barriers and reaction exothermicity (Fig. 5e; also see the Supplementary Information, part II, section H, for transition state analysis using density functional theory). The reaction between ethylene (**1**) and anthracene (**21**) is calculated to proceed with a high activation barrier (Δ*G*^‡^ = 37.1 kcal mol^−1^) and high enthalpy of activation (Δ*H*^‡^ = 23.8 kcal mol^−1^). The reaction free energy (Δ*G*_r_) and enthalpy (Δ*H*_r_) are −12.6 and −27.0 kcal mol^−1^, respectively. By contrast, the corresponding cycloadditions involving the hyperpyramidalized alkenes in cubene (**10**) and quadricyclene (**11**) are calculated to be much more exothermic and exergonic, and the computed Δ*G*^‡^ for the Diels–Alder reactions of **10** and **11** with anthracene (**21**) were found to be only 18.7 and 13.8 kcal mol^−1^, respectively. The exothermicities (Δ*H*_r_) for the reactions of **10** and **11** were calculated to be −74.6 and −83.3 kcal mol^−1^, respectively. Thus, hyperpyramidalization allows cycloadditions to occur readily due to large strain release upon reaction. Consistent with the Bell–Evans–Polanyi relationship, the increase in exothermicity is accompanied by a lowering of the activation barrier by approximately half that amount.

The ultimate consequence of hyperpyramidalization is that cubene (**10**) and 1,7-quadricyclene (**11**) react rapidly^69^ as dienophiles in normal electron-demand Diels–Alder cycloadditions, despite being tetra-substituted without electron-withdrawing groups. Products bearing four newly formed contiguous 3° or 4° carbon centres are readily accessible, thus enabling the introduction of great structural complexity into caged scaffolds. Figure 5f provides a final demonstration of the high level of structural complexity attainable via this methodology. **63-Li** was obtained by lithiation of the corresponding alkyne, which, in turn, was prepared in one step from cubene precursor **17** (Supplementary Information, part I, section H). Treatment of this intermediate with 1,7-quadricyclene cycloadduct **48** led to union of the two fragments, ultimately affording heterodimer **14**. Of note, the tolerance of both coupling partners to such conditions involving organolithium chemistry bodes well for the future applicability of our methodology in multistep synthesis. Heterodimer **14**, the structure of which was confirmed by microcrystal electron diffraction (microED)^70^, possesses nine 3° or 4° carbon centres that were formed through the cycloaddition methodology and fragment coupling, thus pushing the limits of structural and stereochemical complexity attainable using strained intermediate chemistry.

## Conclusion

These studies harness the chemistry of cubene (**10**) and 1,7-quadricyclene (**11**) to provide a simple means to access exceedingly intricate chemical structures of value to medicinal chemists. These intermediates are unusual, as they possess hyperpyramidalized alkenes with weak π-bonding and, consequently, bond orders approaching 1.5. As such, we expect these studies will enable the future design and strategic manipulation of other unconventional intermediates that display hyperpyramidalization or non-integer bond orders for use in chemical synthesis.

## Methods

### Generation and trapping of cubene (10)

The general procedure for the generation and trapping of cubene (**10**) is described as follows. To a 2-dram vial equipped with a stir bar was added cubene precursor **17** (30 mg, 0.10 mmol, 1.0 equiv), followed by the trapping agent (0.15–1.0 mmol, 1.5–10 equiv.). The headspace of the reaction was purged with N_2_ for 5 min, then THF (1.5 ml) and Bu_4_NF (1.0 M in THF, 0.5 ml, 0.50 mmol, 5.0 equiv.) were added sequentially via syringe in a single portion. The vial was sealed with a Teflon-lined screw cap and stirred at 1,000 rpm at 40 °C. After the specified reaction time, the reaction vessel was allowed to cool to 23 °C. The crude mixture was filtered through a 0.5 × 2 cm silica gel plug, eluting with EtOAc (10 ml). The eluate was subsequently concentrated under reduced pressure to dryness, and the crude material was analysed by ^1^H nuclear magnetic resonance (NMR) spectroscopy. The sample for NMR analysis was then recombined with the crude residue and concentrated. Subsequent purification by flash column chromatography on silica gel or preparative thin-layer chromatography using appropriate eluents, followed by drying of the products under high vacuum, yields the corresponding cycloadducts.

### Generation and trapping of 1,7-quadricyclene (11)

The general procedure for the generation and trapping of 1,7-quadricyclene (**11**) is described as follows. To a 2-dram vial containing a stirred solution of 1,7-quadricyclene precursor **20** (35.5 mg, 0.10 mmol, 1.0 equiv.) and trapping agent (0.18–2.0 mmol, 1.8–20 equiv) in THF (1.5 ml) was added Bu_4_NF (1.0 M in THF, 0.5 ml, 0.50 mmol, 5.0 equiv.) in one portion. The pierced septum cap was sealed with melted paraffin wax and the reaction mixture was stirred at 800–1,200 rpm at 23 °C. After the specified reaction time, saturated aq. NH_4_Cl (2 ml) was added to quench the reaction. The crude mixture was extracted with diethyl ether/pentane (1:1, 3× 2 ml), and the organic extracts were dried over Na_2_SO_4_. The crude mixture was concentrated under reduced pressure to dryness, and the crude material was analysed by ^1^H NMR spectroscopy. The sample for NMR analysis was then recombined with the crude residue, concentrated, and purified by chromatography using appropriate eluents, followed by drying under high vacuum to yield the corresponding cycloadducts.

## Supplementary Material

SI

**Supplementary information** The online version contains supplementary material available at https://doi.org/10.1038/s41557-025-02055-9.

## Data Availability

Experimental procedures, characterization data, computational methods and computational data are provided in the Supplementary Information. Crystallographic data for the structures reported in this Article have been deposited at the Cambridge Crystallographic Data Centre, under registry numbers CCDC 2446782 (**39**) and 2456006 (**14**). Copies of the data can be obtained free of charge at https://www.ccdc.cam.ac.uk/structures. Reprints and permissions information is available at www.nature.com/reprints.
