# Supplementary material for: Hyperpyramidalized alkenes with bond orders near 1.5 as synthetic building blocks
Source: Nat Chem. 2026 Jan 21;18(5):913–22. doi: 10.1038/s41557-025-02055-9 (PMC12859755; doi:10.1038/s41557-025-02055-9)
Supplement: Supplementary file 1 — Supplementary Figs. 6–108, Tables 1–14, Experimental procedures and Computational details. [file 41557_2025_2055_MOESM1_ESM.pdf]

nature chemistry

Supplementary information

https://doi.org/10.1038/s41557-025-02055-9

Hyperpyramidalized alkenes with bond orders near 1.5 as synthetic building blocks

In the format provided by the authors and unedited

Supplementary Information – Table of Contents

Part I: Experimental Section 2

Materials and Methods 2

Experimental Procedures 5

- **A. Synthesis of Cubene Silyl Iodide Precursor 17** 5
- **B. Synthesis of 1,7-Quadricyclene Silyl Triflate Precursor 20** 9
- **C. Synthesis of Trapping Agents** 13
- **D. Diels–Alder Trapping Experiments of Cubene (10)** 19
- **E. Additional Reactivity of 10 and Elaboration of Cubene Adducts** 24
- **F. Diels–Alder Trapping Experiments of 1,7-Quadricyclene (11)** 30
- **G. Additional Reactivity of 11 and Elaboration of 1,7-Quadricyclene Adducts** 37
- **H. Synthesis of Heterodimer 14** 41

NMR Spectra 48

Part II: Computational Section 103

- **A. Computational Methods** 103
- **B. Choice of Computation Method and Benchmark Studies** 103
- **C. Olefin Strain Energies of Cubene (10) and 1,7-Quadricyclene (11)** 106
- **D. Diradical Character of Ethylene (1), Cubene (10) and 1,7-Quadricyclene (11)** 108
- **E. Orbital Occupancy-Perturbed Mayer Bond Order Analysis of 1c, 1d and 1e** 111
- **F. Frontier Molecular Orbitals of Cubene (10), and 1,7-Quadricyclene (11)** 113
- **G. Orbital Composition Analysis of 1c, 1d and 1e** 114
- **H. Transition State Geometries for Trapping of each Dienophile with Anthracene (21)** 117
- **I. Mayer Bond Order Analysis of Systematically Distorted Ethylene (1)** 121
- **J. Full Mayer Bond Orders Output of Compounds 2–9, *cis*-8, 10 and 11** 125
- **K. Cartesian Coordinates for Optimized Structures** 161

References 171

Part I: Experimental Section

Materials and Methods. Unless stated otherwise, reactions were conducted in flame-dried glassware under N_2 atmosphere, and commercially obtained reagents were used as received unless otherwise specified. Anhydrous solvents were either freshly distilled or passed through activated alumina columns, unless otherwise stated. Non-commercially available substrates were synthesized according to known preparations or following protocols specified in the Experimental Procedures. 1.0 M Tetrabutylammonium fluoride solution in THF (Bu_4NF), 2.5 M *n*-butyllithium solution in hexanes (*n*-BuLi), 1.7 M *t*-butyllithium solution in hexanes (*t*-BuLi), 2.0 M lithium aluminum hydride solution in THF ($LiAlH_4$), potassium bis(trimethylsilyl)amide (KHMDS), magnesium bromide diethyl etherate ($MgBr_2$•Et_2O), trimethylsilyl chloride (Me_3SiCl), oxalyl chloride ($(COCl)_2$), tetrabutylammonium bromide (Bu_4NBr), anthrone (**71**), dimethyl sulfate ($(CH_3)_2SO_4$), *p*-methoxybenzylamine, biacetyl, phenyl isocyanate, 2-ethylfuran, benzyl bromide (BnBr), *N*-Boc pyrrole (**31**), *N*-phenylpyrrole (**29**), 9-bromoanthracene (**74**), tropone (**49**), α-methylstyrene (**56**) and dimethyl acetylenedicarboxylate (**58**) were obtained from Sigma-Aldrich. Tropolone was purchased from TCI. Cubane-1-carboxylic acid was purchased from 001chemical. Amide **15** was purchased from WuXi AppTec. Bicyclo[2.2.1] hept-5-en-2-one was purchased from AABlocks. Triethylsilyl chloride (Et_3SiCl), *N*-phenyl-bis(trifluoromethanesulfonimide) ($PhNTf_2$) and 4-(dimethylamino)pyridine (DMAP) were obtained from Oakwood. Propargyl bromide (80 wt% in toluene) and potassium permanganate ($KMnO_4$) were purchased from Thermo Fisher Scientific Chemicals. 2,2,6,6-tetramethylpiperdine (TMP), *N*-iodosuccinimide (NIS), sodium pyrithione and 2,2,2-trifluoroiodoethane were obtained from AKSci. Cesium fluoride and 10% palladium on carbon were obtained from Strem Chemicals. 2-Phenylpyridin-3-ol (**70**) and dimethoxy-1,2,3,4-tetrachlorocyclopentadiene (**33**) were obtained from Combi-Blocks. Acetophenone was purchased from Alfa Aesar. 1,4-dibromo-2,3-bis(bromomethyl)but-2-ene, *N*-Boc-D-serine methyl ester, 1,3-Cyclopentadione (**67**), 4-phenyl-1,2,3-triazoline-3,5-dione and diphenylisobenzofuran (**25**) were obtained from Ambeed. Prior to use diphenylisobenzofuran (**25**) was purified by automated column chromatography. 2,2,6,6-Tetramethylpiperdine (TMP), trimethylsilyl chloride (Me_3SiCl) and triethylsilyl chloride (Et_3SiCl) were distilled over CaH_2 and stored in a Schlenk flask under N_2 prior to use. Oxalyl chloride was distilled and stored in a Schlenk flask under N_2 prior to use. Reaction temperatures at or above 23 °C were controlled using an

IKAmag temperature modulator and, unless stated otherwise, performed at room temperature (approximately 23 °C). Photochemical reactions were conducted with a Hanovia medium-pressure Hg lamp equipped with a Pyrex water jacket at the distance indicated, or with a 32W fluorescent grow lamp at the distance indicated, or with NEPTONION High Intensity UVB Reptile Bulb, UVB 10.0 Reptiles Bulb, LED 5W UV Light Bulb, UVB Compact Fluorescent Lamp, Suitable for Desert Reptiles (E27,110V) at the distance indicated. For ozone generation, ENALY 5000BF was used. Analytical thin layer chromatography (TLC) was conducted with EMD gel 60 F254 pre-coated plates (0.25 mm) or SiliaPlate glass-backed basic alumina (250 μm) TLC plates and visualized using a combination of UV light and $KMnO_4$, CAM or PMA staining. Preparative thin layer chromatography (TLC) was conducted with EMD gel 60 F254 pre-coated plates (0.5 mm) and visualized using UV light. Silicycle Siliaflash P60 (particle size 40 – 63 μm) was used for flash column chromatography and RediSep Gold Silica (20 – 40 μm) was used for automated chromatography on a CombiFlash NextGen 300+ instrument unless otherwise specified. ^{1}H NMR and 2D-NOESY spectra were recorded on Bruker spectrometers (at 300, 500 or 600 MHz) and are reported relative to the residual solvent signal. Data for ^{1}H NMR spectra are reported as follows: chemical shift (δ ppm), multiplicity, coupling constant (Hz), and integration. ^{13}C NMR spectra were recorded on Bruker spectrometers (125 or 150 MHz) and are reported relative to the residual solvent signal. Data for ^{13}C NMR are reported in terms of chemical shift, and, when necessary, multiplicity, and coupling constant (Hz). ^{19}F NMR spectra were recorded on Bruker spectrometers (at 565 MHz) and are reported in terms of chemical shift (δ ppm). ^{1}H NMR yields were determined by addition of an aliquot of mesitylene in a stock solution of C_6D_6 to the concentrated crude reaction mixture followed by subsequent ^{1}H NMR analysis. Uncorrected melting points were measured using a Digimelt MPA160 melting point apparatus. IR spectra were recorded on a Perkin-Elmer UATR Two FT-IR spectrometer and are reported in terms of frequency absorption (cm^{-1}). DART-MS spectra were collected on a Thermo Exactive Plus MSD (Thermo Scientific) equipped with an ID-CUBE ion source and a Vapur Interface (IonSense Inc.). Both the source and MSD were controlled by Excalibur software v. 3.0. The analyte was spotted onto OpenSpot sampling cards (IonSense Inc.) using CH_2Cl_2 as the solvent. Ionization was accomplished using UHP He plasma with no additional ionization agents. Diastereomeric ratios (d.r.) and regioselective ratios (r.r.) were determined by ^{1}H NMR analysis of crude reaction mixtures.

Cubyl Amide **15**,[1] vinyl bromide **18**,[2] *O*-benzyl tropolone (**35**),[3] 2,5-diethylfuran (**27**),[4] oxazolidinone **37**,[5] 9-methoxyanthracene (**23**),[6] ene carbamate **42**[7] and dibromide **68**[8] were prepared according to the literature procedures and all ^{1}H NMR spectral data matched those reported in the literature.

Experimental Procedures

A. Synthesis of Cubene Silyl Iodide Precursor 17

An overview of the route to **17** is as follows, beginning from either cubane-1-carboxylic acid or amide **15**. As indicated in the Materials and Methods section, amide **15** can be purchased from WuXi AppTec. It can also be synthesized from cubane-1-carboxylic acid following a known procedure.[1]

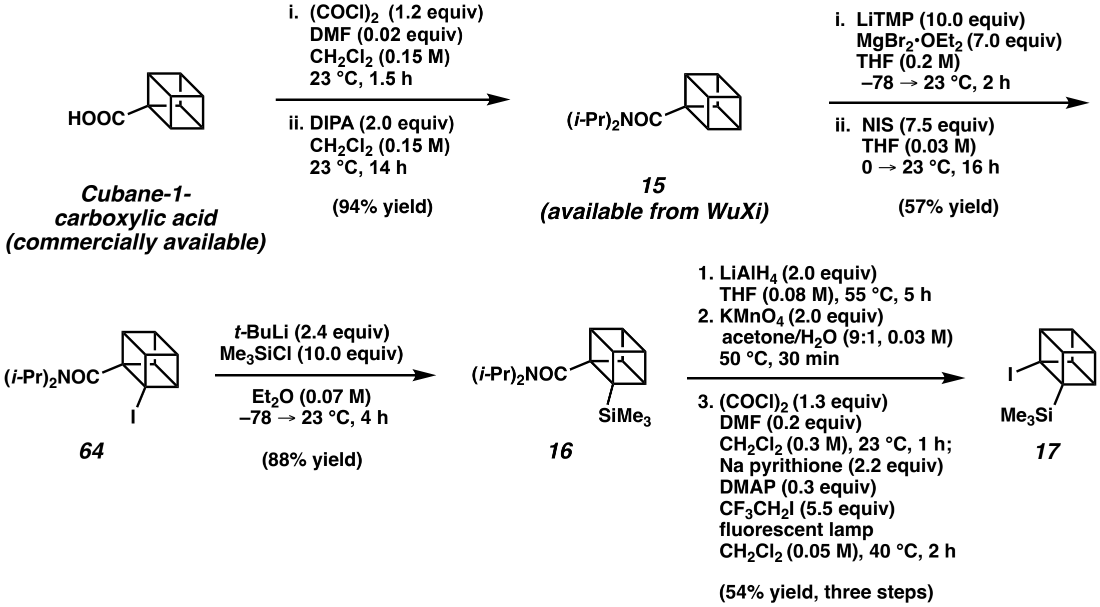

Note: The route to **17** follows what has previously been described by Eaton and co-workers,[9] but with modification of experimental details. We have found that the procedures reported here are reproducible and these have allowed access to multigram quantities of **17** during the course of our studies.

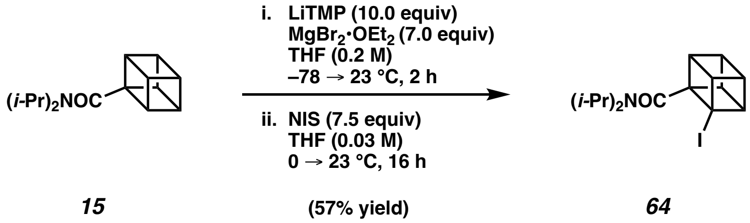

Iodo amide 64. *Note: It was found this reaction did not scale well above 1.0 g. Material throughput was achieved on a 1.0 g scale with respect to amide* ***15*** *in batches. Crude reaction mixtures were then combined for work up and purification. The following reagents and glassware are reported for a 1.0 g pass, which was performed in duplicate.*

To a 250 mL round bottom flask equipped with a stir bar was prepared a solution of 2,2,6,6-tetramethylpiperdine (7.50 mL, 43.2 mmol, 10 equiv) in THF (43.0 mL, 0.1 M) at –78 °C. *n*-BuLi (2.34 M in hexanes, 18.4 mL, 43.2 mmol, 10 equiv) was added dropwise via syringe over 5 min. The solution was brought to 23 °C and stirred for 30 min before cooling back to –78 °C. In a 500 mL round bottom flask equipped with a stir bar and containing a solution of amide **15** (1.00 g, 4.32 mmol) in THF (86.5 mL, 0.05 M) was added $MgBr_2{\bullet}Et_2O$ (7.80 g, 30.2 mmol, 7 equiv) in two ~4 g portions over 5 min at 23 °C (CAUTION: slight exotherm is observed). The resulting mixture was cooled to –78 °C then the freshly prepared base was added via cannula over 15 min. Following the addition of base, the reaction mixture was warmed to 23 °C and stirring continued for 2 h over which time the solution becomes dark brown. The mixture was then cooled to 0 °C before a solution of NIS (5.75 g, 32.4 mmol, 7.5 equiv) in THF (43.2 mL, 0.1 M) was added via syringe in one portion. The reaction was protected from light using aluminum foil and stirred at 23 °C. After 16 h saturated aq. $Na_2S_2O_3$ (300 mL) was added to the reaction mixture. At this point both 1.0 g reaction mixtures were combined in a single separatory funnel and the aqueous layer was extracted with EtOAc (3 x 100 mL). The combined organic extracts were sequentially washed with a mixture of deionized H_2O (2 x 100 mL) and aq. 1M HCl (2 x 20 mL), then brine (200 mL) then dried over Na_2SO_4, filtered, and concentrated under reduced pressure. The crude material was purified via flash column chromatography (0 → 5% benzene:EtOAc) to afford iodo amide **64** (1.76 g, 4.93 mmol, 57% yield) as a pale-brown solid. **Iodo amide 64**: R_f 0.50 (9:1 benzene:EtOAc); ^1H NMR (300 MHz, $CDCl_3$) δ 4.45 – 4.39 (m, 2H), 4.33 – 4.26 (m, 2H), 4.18 – 4.05 (m, 2H), 3.57 (sep, J = 6.5 Hz, 1H), 3.36 (sep, J = 6.8 Hz, 1H), 1.45 (d, J = 6.8 Hz, 6H), 1.31 (d, J = 6.5 Hz, 6H). Spectral data matched those previously reported.[1]

Note: In some cases, following column chromatography a colored impurity was present. Additional purification by crystallization of ***64*** *from hot pentanes was successful in providing pale-brown product.*

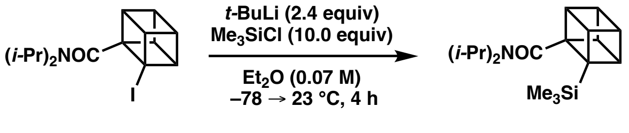

64 (88% yield) **16**

Silyl amide 16. In a 100 mL round bottom flask equipped with a stir bar was prepared a solution of Et_2O (30 mL, 0.1 M) and *t*-BuLi (1.7 M, 4.15 mL, 2.4 equiv) at –78 °C. A solution of iodo amide **64** (1.05 g, 2.94 mmol, 1.0 equiv) in Et_2O (11.8 mL, 0.25 M) was then added dropwise over 5 min. The resulting solution was stirred at –78 °C for 1 h, before Me_3SiCl (3.73 mL, 29.4 mmol, 10 equiv) was added dropwise via syringe over 2 min. The solution was brought to 23 °C. After 4 h the reaction mixture was diluted with deionized H_2O (50 mL), then saturated aq. $NaHCO_3$ (50 mL). The aqueous layer was extracted with EtOAc (4 x 50 mL), then the combined organic extracts were washed with brine (100 mL), dried over Na_2SO_4, filtered, and concentrated under reduced pressure. The crude material was purified via automated column chromatography (0 → 40% hexanes:EtOAc) to afford silyl amide **16** (789 mg, 2.60 mmol, 88% yield) as a white solid. **Silyl amide 16**: R_f 0.50 (9:1 hexanes:EtOAc); ^{1}H NMR (300 MHz, $CDCl_3$) δ 4.20 – 4.13 (m, 2H), 4.10 – 4.03 (m, 1H), 3.99 – 3.91 (m, 1H), 3.78 – 3.72 (m, 2H), 3.55 (sep, J = 6.6 Hz, 1H), 3.28 (sep, J = 6.8 Hz, 1H), 1.40 (d, J = 6.9 Hz, 6H), 1.19 (d, J = 6.6 Hz, 6H), 0.03 (s, 9H). Spectral data matched those previously reported.[9]

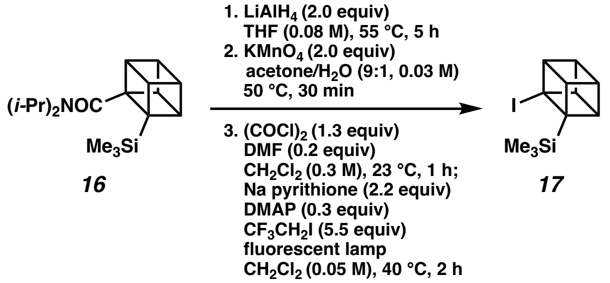

(54% yield, three steps)

Silyl iodide 17. To a 100 mL round bottom flask equipped with a stir bar was added silyl amide **16** (766 mg, 2.52 mmol, 1.0 equiv), THF (31.5 mL, 0.08 M) then $LiAlH_4$ (2 M in THF, 2.5 mL, 5.05 mmol, 2.0 equiv) at 23 °C via syringe in one portion. The flask was then equipped with an air condenser then placed in an aluminum block preheated to 55 °C. After 5 h, the mixture was cooled to 0 °C before saturated aq. potassium sodium tartrate (30 mL) was added. The mixture was vigorously stirred until two distinct layers formed before the aqueous layer extracted with EtOAc (3 x 20 mL). The combined organic extracts were washed with brine (50 mL), dried over Na_2SO_4,

filtered and concentrated under reduced pressure to provide tertiary amine intermediate (703 mg) as a yellow oil. The crude material was used directly in the following step.

To a 100 mL round bottom flask equipped with a stir bar and amine intermediate (703 mg) was added acetone (23.8 mL, 0.1 M). The solution was brought to 50 °C before a mixture of $KMnO_4$ (751 mg, 4.75 mmol, 2.0 equiv) in deionized H_2O (9.50 mL, 0.1 M) and acetone (47.5 mL, 0.05 M) was added in one portion. After 30 min the mixture was cooled to 23 °C then added to a 1M aq. solution of $NaHSO_3$ (200 mL). 1M HCl (10 mL) was added and the solution then turned from purple/brown to colorless. The aqueous layer extracted with EtOAc (3 x 75 mL). The combined organic extracts were washed with brine (50 mL), dried over Na_2SO_4, filtered and concentrated under reduced pressure to provide crude carboxylic acid (554 mg) as a pale-yellow oil. The crude material was thoroughly dried under high vacuum then used directly in the following step.

In a 25 mL round bottom flask equipped with a stir bar, was prepared a solution of carboxylic acid intermediate (554 mg), CH_2Cl_2 (8.4 mL, 0.3 M) and DMF (40 µL 503 µmol, 0.2 equiv). Oxalyl chloride (286 µL, 3.271 mmol, 1.3 equiv) was added in one portion at 23 °C. After stirring for 1 h the mixture was concentrated under reduced pressure then dried under high vacuum for 1 h giving the acyl chloride intermediate. In a 50 mL round bottom flask that was capped with a rubber septum, was prepared a mixture of sodium pyrithione (825 mg, 5.54 mmol, 2.2 equiv), DMAP (92 mg, 754 µmol, 0.3 equiv), 2,2,2-trifluoroiodoethane (1.35 mL, 13.84 mmol, 5.5 equiv) in CH_2Cl_2 (8.3 mL, 0.3 M). This reaction flask was placed in a preheated oil bath set to 40 °C before a solution of the freshly prepared acyl chloride in CH_2Cl_2 (16.7 mL, 0.15 M) was added dropwise via syringe. The rubber septum was then replaced with a flame dried waterless condenser and the reaction vessel was irradiated 10 cm from a 32W fluorescent grow lamp for 2 h. The reaction was then cooled to 23 °C before CH_2Cl_2 (10 mL) and deionized H_2O (30 mL) were added. They layers were separated before the aqueous layer was further extracted with CH_2Cl_2 (2 x 20 mL). The combined organic extracts were washed with aq. 1M HCl (30 mL), then brine (30 mL) and dried over Na_2SO_4, filtered and concentrated under reduced pressure. Purification via automated column chromatography (0% → 5% hexanes:EtOAc) yielded silyl iodide **17** (405 mg, 1.36 mmol, 54% yield, three steps) as a colorless oil. **Silyl iodide 17**: 0.81 (100% hexanes); ^1H NMR (500 MHz, $CDCl_3$) δ 4.43– 4.39 (m, 2H), 4.26– 4.21 (m, 1H), 4.20 – 4.16 (m, 1H), 4.08 – 4.05 (m, 2H), 0.04 (s, 9H). Spectral data matched those previously reported.[9]

B. Synthesis of 1,7-Quadricyclene Silyl Triflate Precursor 20

An overview of the synthetic route to **20** is as follows, beginning from commercially available bicyclo[2.2.1]hetp-5-en-2-one (purchased from AAblocks).

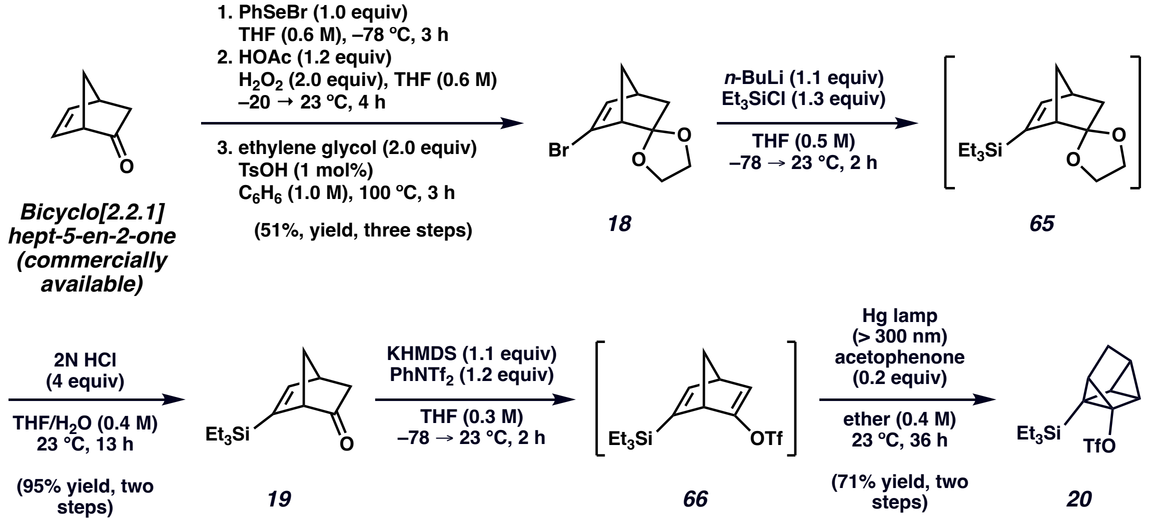

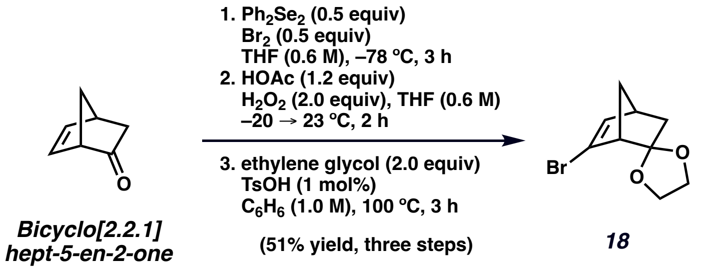

Bromo ketal **18** was synthesized from commercially available bicyclo[2.2.1]hept-5-en-2-one according to the reported procedure with modifications.[2] To a flame-dried 1L round bottom flask equipped with a stirbar was added diphenyl diselenide (29.2 g, 93.4 mmol, 0.5 equiv). The flask is evacuated and back-filled with N_2 three times then THF (150 mL) was added. Bromine (14.9 g, 4.8 mL, 93.4 mmol, 0.5 equiv) was added via syringe over 1 min to afford a solution of PhSeBr in THF. A separate flame-dried 1L 3-neck round bottom flask equipped with a stirbar and a flame-dried 250 mL addition funnel was prepared. To the flask was added bicyclo[2.2.1]hept-5-en-2-one (20.2 g, 187 mmol, 1.0 equiv) via syringe in one portion, before THF (150 mL) was added via cannula. The resulting solution was cooled to –78 ºC. The solution of PhSeBr prepared above was transferred into the addition funnel via cannula, then added to the reaction mixture dropwise over

20 min. The brown-red solution was stirred at –78 ºC for 2 hours. The solution was then warmed up to 23 ºC. The solution was then transferred to a 1L round bottom flask, rinsing with Et_2O (50 mL), and concentrated under reduced pressure to afford the crude bromo selenide as a deep-red oil, which is used directly in the next step without further purification.

In a 1L round bottom flask, the crude bromo selenide was dissolved in THF (300 mL), and the resulting solution was cooled to –20 ºC. Hydrogen peroxide (42.4 g, 38.2 mL, 30 wt% in H_2O, 374 mmol, 2.0 equiv) is added dropwise via syringe over 15 min, during which the bath temperature was kept at –20 °C. The mixture is then stirred for 2.5 h, during which the cooling bath was slowly warmed up to 10 °C. The flask was then warmed to 23 °C and stirred for another 1.5 h. The crude reaction mixture was diluted with Et_2O (2 L) and deionized H_2O (200 mL). The mixture was vigorously shaken before the aqueous layer was separated then discarded. The organic layer was then washed sequentially with saturated aq. Na_2CO_3 (150 mL), saturated aq. $NaHCO_3$ (2 x 150 mL), saturated aq. $Na_2S_2O_3$ (150 mL) and brine (200 mL). The organic layer was dried over Na_2SO_4, filtered, and concentrated under reduced pressure to give the crude product as a red oil. The crude product was purified using automated column chromatography (0% → 20% hexanes:EtOAc) to afford bromo-ketone (23.6 g) as a yellow oil, which is used in the next step without characterization.

To a 250 mL round bottom flask was added above-prepared bromo-ketone (23.6 g), ethylene glycol (14.1 mL, 252 mmol, 2.0 equiv), *p*-TsOH•H_2O (240 mg, 1.26 mmol, 1 mol%) and benzene (126 mL, 1.0 M). The flask was equipped with a Dean-stark trap and the mixture was heated at reflux (heating mantle set at 105 ºC) for 3 hours. The mixture was then cooled to 23 ºC and saturated aq. $NaHCO_3$ (100 mL) was added in one portion. Et_2O (100 mL) and deionized H_2O (100 mL) were added. The organic layer was separated and the aqueous layer was further extracted with Et_2O (3 x 100 mL). The combined organic extracts were dried over Na_2SO_4, filtered and concentrated under reduced pressure to afford the crude product as a viscous oil, which solidified upon storage in freezer at –20ºC. Crude product was transferred into a 250 mL Erlenmeyer flask and CH_3CN (30 mL) was added. The solution was heated to reflux (using a heatgun), and cooled slowly to 23 ºC over 30 min, then placed in a –20 ºC freezer for 1 h. The pale-yellow crystals were then collected by filtration and rinsed with ca. 10 mL of cold CH_3CN (chilled in the –20 ºC freezer).

The crystals were dried under high vacuum to afford the first batch of product as a pale yellow solid. A second crop of crystal product were obtained by concentrating the filtrate from the first crop under a reduced pressure. The semi-solid product was transferred to a 125 mL Erlenmeyer flask then was dissolved in CH_3CN (25 mL) at 23 ºC, and cooled in a –20 ºC freezer. To induce crystallization, a chip of crystalline product was added. After 30 min at –20 ºC, the second batch of pale yellow crystalline product was collected via filtration. Both crops of crystals were combined to give bromo ketal **18** (21.9 g, 94.8 mmol, 51% yield, 3 steps). **Bromo ketal 18**: R_f 0.64 (5:1 hexanes:EtOAc); ^{1}H NMR (600 MHz, $CDCl_3$): δ 6.35 (d, J = 3.2 Hz, 1H), 4.05 – 3.90 (m, 4H), 2.87 (m, 1H), 2.74 (m, 1H), 1.96 (ddt, J = 8.8, 3.7, 1.9 Hz, 1H), 1.91 (dd, J = 12.6, 3.7 Hz, 1H), 1.75 (d, J = 8.9 Hz, 1H), 1.67 (dd, J = 12.6, 3.6 Hz, 1H). Spectral data matched those previously reported.[2]

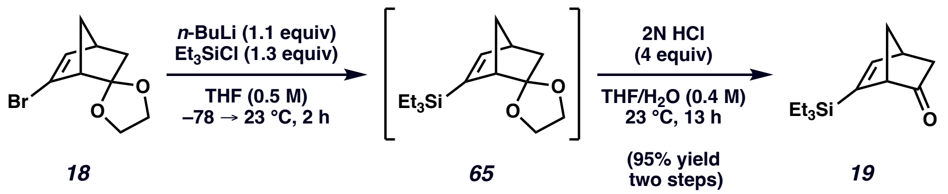

Ketone 19. A 1 L round bottom flask equipped with a stir bar containing bromo ketal **18** (30.3 g, 131 mmol, 1.0 equiv) was fitted with a 250 mL glass addition funnel. The flask was evacuated and back-filled with N_2 three times, before THF (262 mL, 0.5 M) was added. *n*-BuLi (2.5 M in hexanes, 58 mL, 140 mmol, 1.1 equiv) was then added dropwise at –78 ºC over 10 min. The solution was stirred at –78 °C for 1 h, before triethylsilyl chloride (29.0 mL, 170 mmol, 1.3 equiv) was added dropwise via syringe over 10 min. The solution was allowed to slowly warm up to 23 °C over 1 h and the reaction mixture was then quenched with saturated aq. $NaHCO_3$ (100 mL). The organic layer was separated and the aqueous layer was extracted with 1:1 diethyl ether/hexanes (3 x 50 mL). The combined organic extracts were combined and dried over Na_2SO_4, filtered, and concentrated under reduced pressure to give crude vinyl silane **65**, which was directly used in the next step.

The crude material was then transferred into a 1 L round bottom flask equipped with a stir bar and dissolved in THF (260 mL, 0.5 M). Then aq. 2M HCl (260 mL, 4.0 equiv) was added in one portion. The biphasic mixture was vigorously stirred under air at 23 ºC for 13 h, before hexanes

(100 mL) was added. The organic layer was separated and the aqueous layer was extracted with hexanes (2 x 100 mL). The combined organic extracts were dried over Na_2SO_4, filtered and concentrated under reduced pressure to afford crude silyl ketone. The crude material was then purified via automated column chromatography (0% → 5% hexanes:EtOAc) to afford ketone **19** (27.5 g, 124 mmol, 95% yield, two steps) as a pale-yellow liquid. **Ketone 19**: R_f 0.17 (90:5:5 hexanes/diethyl ether/CH_2Cl_2); ^{1}H NMR (600 MHz, $CDCl_3$): δ 6.86 (d, J = 2.7 Hz, 1H), 3.19 (brs, 1H), 3.07 (brs, 1H), 2.14 – 2.09 (m, 1H), 1.97 (d, J = 9.7 Hz, 1H), 1.96 – 1.93 (m, 1H, overlapping signal), 1.80 (dd, J = 16.5, 4.4 Hz, 1H), 0.90 (t, J = 7.9 Hz, 9H), 0.63 – 0.56 (m, 6H); ^{13}C NMR (150 MHz, $CDCl_3$): δ 215.9, 154.0, 142.2, 59.4, 51.2, 41.7, 37.9, 7.4, 3.1; IR (film): 2955, 2876, 1741, 733, 719 cm^{-1}; HRMS-APCI (*m/z*) [M+H]$^+$ calcd for $C_{13}H_{23}OSi^+$, 223.1513; found 223.1512.

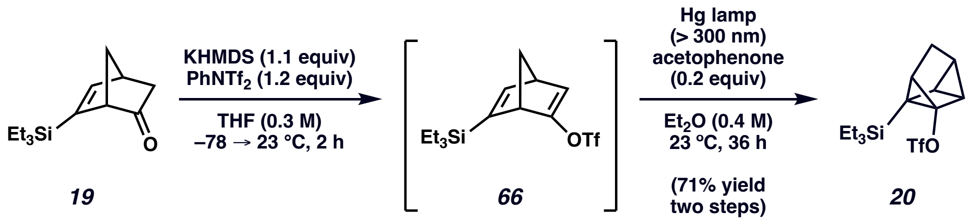

Silyl triflate 20. To a 250 mL round bottom flask equipped with a stir bar was added KHMDS (4.94 g, 24.8 mmol, 1.1 equiv) in the N_2–filled glovebox. The flask was sealed with rubber septum and removed from the glovebox. To the flask was then added THF (40 mL, 0.55 M) via syringe and the resulting solution was cooled to –78 °C. Ketone **19** (5.01 g, 22.5 mmol, 1.0 equiv) was then added in one portion via syringe and the resulting solution was stirred at –78 ºC for 30 min. In a separate round bottom flask, phenyl triflimide (9.66 g, 27.0 mmol, 1.2 equiv) was dissolved in THF (16 mL, 1.40 M). The resulting solution was transferred to the reaction mixture dropwise via cannula at –78 ºC over 5 min, followed by rinsing with additional THF (16 mL, 1.40 M). The resulting mixture was stirred at –78 ºC for 10 min, then allowed to warm up to 0 °C by transferring the reaction flask into an ice water bath. The mixture was stirred at 0 ºC for another 1 h. The flask was then allowed to warm up to 23 ºC and stirred for another 15 min, before saturated aq. $NaHCO_3$ (20 mL), deionized H_2O (20 mL) and hexanes (100 mL) were added sequentially to quench the reaction. The organic layer was collected and the aqueous layer was extracted with hexanes (2 x 40 mL). The organic extracts were combined and washed with brine (50 mL), dried over Na_2SO_4,

and concentrated under reduced pressure to afford crude product. The crude material was purified by filtration through a silica gel plug, eluting with hexanes (500 mL) to afford enol triflate **66** (7.31 g, 20.6 mmol) as a clear colorless liquid, which was directly subjected to the next step.

To a 100 mL Pyrex Schlenk tube equipped with a stir bar evacuated and back-filled with N_2 for 3 times was added triflate **66** (7.22 g, 20.4 mmol, 1.0 equiv) via pipette under rapid N_2 flow. The tube was sealed with rubber septum, then acetophenone (0.48 g, 0.47 mL, 4.0 mmol, 0.20 equiv) and diethyl ether (50 mL, 0.4 M) were added via syringe. The septum was sealed with electrical tape, and the Schlenk tube was placed 10 cm from a high-pressure mercury lamp equipped with Pyrex water cooling jacket and irradiated at 23 °C for 36 h. NEt_3 (1 mL) was then added to the reaction mixture to neutralize acidic material formed during the reaction. The mixture was then concentrated under reduced pressure to afford crude product. The crude material was purified via automated column chromatography (hexanes) to afford silyl triflate **20** (5.62 g, 15.9 mmol, 71% yield, two steps) as a colorless liquid. **Silyl triflate 20**: R_f 0.40 (hexanes); ^{1}H NMR (600 MHz, C_6D_6): δ 2.13 (dt, J = 11.4, 1.3 Hz, 1H), 2.05 (dd, J = 6.4, 4.8 Hz, 1H), 1.97 (q, J = 1.5 Hz, 1H), 1.56 (dt, J = 11.4, 1.5 Hz, 1H), 1.29 (dd, J = 6.3, 4.5 Hz, 1H), 1.21 (tq, J = 4.5, 1.4 Hz, 1H), 0.92 (t, J = 7.9 Hz, 9H), 0.57 – 0.42 (m, 6H); ^{13}C NMR (150 MHz, C_6D_6): δ 118.8 (q, J = 320 Hz), 80.4, 34.1, 31.3, 27.8, 24.0, 22.9, 15.0, 7.5, 3.3; ^{19}F NMR (565 MHz, C_6D_6) δ –75.5; IR (film): 2957, 2879, 1420, 1209, 1144 cm^{-1}.

C. Synthesis of Trapping Agents

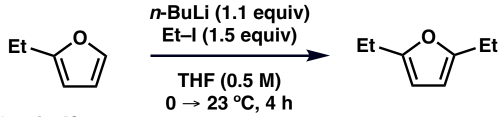

2-ethylfuran *27*

2,5-Diethylfuran (**27**) was synthesized from commercially available 2-ethylfuran according to a reported procedure.[4] **2,5-Diethylfuran** (**27**): R_f 0.92 (20:1 *n*-pentane:Et_2O); ^{1}H NMR (600 MHz, $CDCl_3$): δ 5.86 (s, 2H), 2.61 (q, J = 7.5 Hz, 4H), 1.22 (t, J = 7.6 Hz, 6H). Spectral data matched those previously reported.[4]

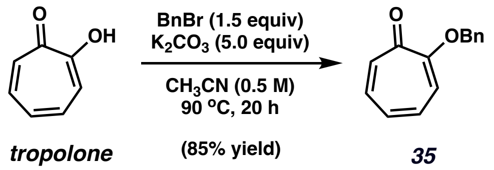

O-Benzyl tropolone (**35**) was synthesized from commercially available tropolone according to a reported procedure.[3] ***O*-Benzyl tropolone** (**35**): R_f 0.32 (1:1 hexanes:EtOAc); ^{1}H NMR (600 MHz, $CDCl_3$): δ 7.43 (m, 2H), 7.39 – 7.36 (m, 2H), 7.34 – 7.30 (m, 1H), 7.26 – 7.23 (m, 1H), 7.21 (ddd, *J* = 12.2, 7.7, 1.2 Hz, 1H), 6.97 (t, *J* = 10.5 Hz, 1H), 6.87 – 6.80 (ddd, *J* = 10.3, 7.7, 1.2 Hz, 1H), 6.80 – 6.76 (d, *J* = 9.9 Hz, 1H), 5.26 (s, 2H). Spectral data matched those previously reported.[3]

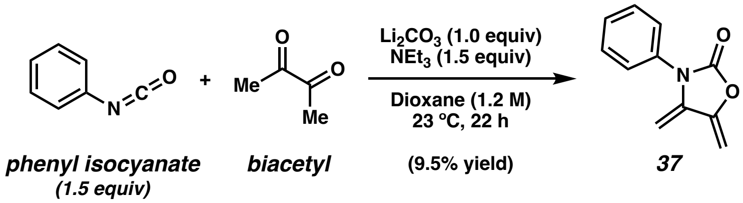

Oxazolidinone **37** was synthesized from commercially available phenyl isocyanate and diacetyl according to the reported procedure.[5] **Oxazolidinone 37**: R_f 0.60 (5:1 hexanes:EtOAc); ^{1}H NMR (600 MHz, $CDCl_3$) δ 7.54 – 7.47 (t, *J* = 7.4 Hz, 2H), 7.45 – 7.39 (t, *J* = 7.3 Hz, 1H), 7.38 – 7.33 (d, *J* = 7.4 Hz, 2H), 4.98 (d, *J* = 3.5 Hz, 1H), 4.93 (d, *J* = 3.6 Hz, 1H), 4.76 (d, *J* = 3.1 Hz, 1H), 4.36 (d, *J* = 3.1 Hz, 1H). Spectral data matched those previously reported.[5]

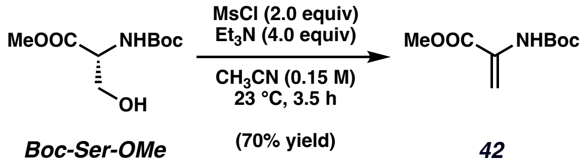

Ene-carbamate **42** was synthesized from commercially available Boc-Ser-OMe according to the reported procedure.[7] **Ene-carbamate 42**: R_f 0.58 (9:1 hexanes:EtOAc); ^{1}H NMR (600 MHz, $CDCl_3$): δ 7.00 (s, 1H), 6.15 (s, 1H), 5.71 (s, 1H), 3.82 (d, *J* = 2.1 Hz, 3H), 1.47 (d, *J* = 2.1 Hz, 9H). Spectral data matched those previously reported.[7]

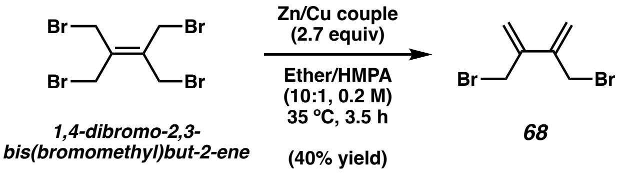

Dibromide **68** was synthesized from commercially available 1,4-dibromo-2,3-bis(bromomethyl)but-2-ene according to the reported procedure.[8] **Dibromide 68**: R_f 0.71 (10:1 hexanes:CH_2Cl_2); ^{1}H NMR (600 MHz, $CDCl_3$): δ 5.54 (s, 2H), 5.51 (s, 2H), 4.17 (s, 4H). Spectral data matched those previously reported.[8]

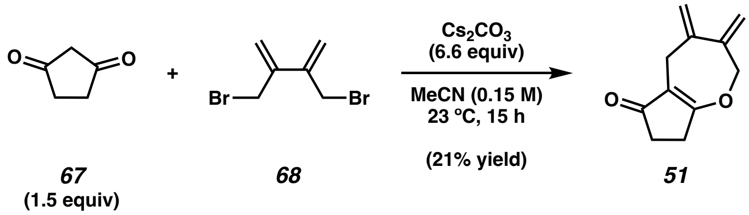

Diene 51 To a 250 mL round bottom flask equipped with a stir bar and a rubber septum that contained freshly prepared dibromide **68** (2.60 g, 10.8 mmol, 1.0 equiv) purged with N_2 was quickly added 1,3-cyclopentadione (**67**) (1.59 g, 16.3 mmol, 1.5 equiv) and cesium carbonate (23.3 g, 71.5 mmol, 6.6 equiv) under rapid flow of N_2. The flask was then sealed with N_2 lined rubber septum and MeCN (72 mL, 0.15 M) was added via syringe. The resulting suspension was stirred at 23 ºC for 15 h. Diethyl ether (100 mL) was then added to the reaction mixture, and the resulting mixture was filtered through a pad of celite, eluting with diethyl ether (3 x 50 mL). The filtrate was concentrated under reduced pressure to give crude material, which was purified by automated column chromatography (40 → 100% hexanes:EtOAc) to afford diene **51** (401 mg, 2.27 mmol, 21% yield) as a pale yellow oil. **Diene 51**: R_f 0.56 (1:1 hexanes:EtOAc); ^{1}H NMR (600 MHz, $CDCl_3$): δ 5.62 (s, 1H), 5.34 (s, 1H), 5.19 (s, 1H), 4.94 (s, 1H), 4.84 (s, 2H), 3.24 (s, 2H), 2.48 – 2.45 (m, 2H), 2.45 – 2.42 (m, 2H); ^{13}C NMR (150 MHz, $CDCl_3$): δ 204.5, 184.7, 144.1, 143.5, 116.8, 116.4, 111.7, 73.7, 33.7, 28.2, 27.1; IR (film): 2926, 1690, 1619, 1345, 1265 cm^{-1}; HRMS-APCI (*m/z*) $[M+H]^+$ calcd for $C_{11}H_{13}O_2^+$, 177.0910; found 177.0909.

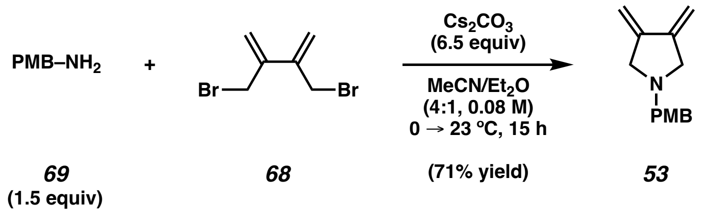

Diene 53. To a 250 mL round bottom flask equipped with a stir bar was added cesium carbonate (8.8 g, 27 mmol, 6.5 equiv). The flask was sealed with a rubber septum, evacuated and back-filled with N_2, followed by addition of MeCN (41 mL, 1.0 M) and *p*-methoxybenzyl amine (**69**) (875 mg, 6.4 mmol, 1.5 equiv) via syringe. The mixture was cooled to 0 ºC in an ice water bath, and a solution of dibromide **68** (1.00 g, 4.2 mmol, 1.0 equiv) in diethyl ether (10 mL) was added dropwise via syringe over 5 min. The reaction mixture was allowed to slowly warm up to 23 ºC. After 15 h the resulting mixture was filtered through a pad of celite, eluting with diethyl ether (~150 mL). The filtrate was concentrated under reduced pressure to give crude material, which was purified by automated column chromatography (0 → 50% hexanes:EtOAc) to afford diene **53** (640 mg, 3.0 mmol, 71% yield) as a colorless oil. **Diene 53**: R_f 0.60 (3:1 hexanes:EtOAc); ^{1}H NMR (600 MHz, $CDCl_3$): δ 7.28 – 7.24 (overlapped with solvent peak, 2H), 6.88 (d, J = 8.4 Hz, 2H), 5.37 (t, J = 2.2 Hz, 2H), 4.90 (s, 2H), 3.80 (s, 3H), 3.59 (s, 2H), 3.30 (s, 4H); ^{13}C NMR (150 MHz, $CDCl_3$): δ 158.9, 144.7, 130.7, 130.2, 113.8, 103.5, 60.0, 59.9, 55.4; IR (film): 3081, 2933, 2763, 1613, 1513, 1249 cm^{-1}; HRMS-APCI (*m/z*) $[M+H]^+$ calcd for $C_{14}H_{18}NO^+$, 216.1383; found 216.1382.

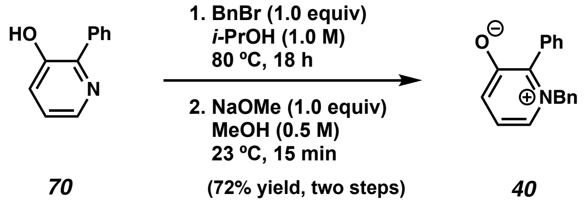

Oxidopyridinium 40. In a scintillation vial equipped with a stir bar was prepared a solution of 2-phenylpyridin-3-ol (**70**) (500 mg, 2.9 mmol, 1.0 equiv) in isopropanol (2.9 mL, 1.0 M) at 23 °C. Benzyl bromide (350 µL, 2.9 mmol, 1.0 equiv) was then added in one portion and the vial was sealed with a Teflon-lined cap. The reaction mixture was stirred at 80 °C for 18 h under ambient atmosphere. The reaction was then cooled to 23 °C and concentrated under reduced pressure to provide the crude product, which was purified by silica gel column chromatography (10% CH_2Cl_2:MeOH) to afford *N*-benzylpyridinium bromide intermediate (732 mg) as a tan foam. This

was used directly in the subsequent step.

To a solution of *N*-benzylpyridinium bromide intermediate (732 mg, 2.1 mmol, 1.0 equiv) in MeOH (1.0 mL, 2.0 M) at 0 °C was added a solution of NaOMe, freshly prepared from sodium metal (49 mg, 2.1 mmol, 1 equiv) in MeOH (3.0 mL, 0.7 M) under N_2. The mixture was stirred for 15 min at 23 °C, then the reaction was diluted with isopropanol (2 mL) and concentrated under reduced pressure. The resulting slurry was filtered through a pad of Celite, eluting with isopropanol (8 mL). The eluate was concentrated under reduced pressure and placed under high vacuum to afford oxidopyridinium **40** (559 mg, 2.1 mmol, 72% yield, two steps) as an orange solid. **Oxidopyridinium 40**: R_f 0.70 (1:1 hexanes:EtOAc); mp: 161.9 °C; ^{1}H NMR (600 MHz, $CDCl_3$): δ 7.44 – 7.37 (m, 4H), 7.33 – 7.28 (m, 3H), 7.28 – 7.24 (overlapped with solvent peak, 2H), 7.23 – 7.20 (m, 1H), 7.06 (d, *J* = 5.5 1H), 6.91 (d, *J* = 6.7, 2H), 5.18 (s, 2H); ^{13}C NMR (125 MHz, $CDCl_3$): δ 169.0, 146.1, 134.4, 134.3, 132.0, 130.0, 129.4, 129.2, 129.0, 128.9, 127.1, 125.8, 121.7, 61.5; IR (film): 3062, 2925, 1573, 1480, 700 cm^{-1}; HRMS-APCI (*m/z*) [M+H]$^+$ calcd for $C_{18}H_{16}NO^+$, 262.1226; found 262.1248.

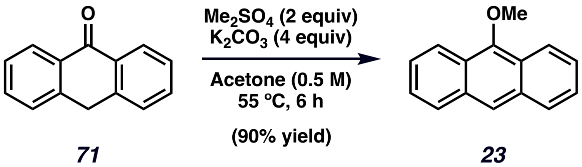

71 23

9-Methoxyanthracene (**23**) was synthesized from commercially available **71** according to the reported procedure.[6] **9-Methoxyanthracene** (**23**): R_f 0.63 (9:1 hexanes:EtOAc); ^{1}H NMR (600 MHz, $CDCl_3$): δ 8.31 (d, *J* = 8.7 Hz, 2H), 8.23 (s, 1H), 8.02 – 7.99 (d, *J* = 7.7 Hz, 2H), 7.52 – 7.45 (m, 4H), 4.16 (s, 3H). Spectral data matched those previously reported.[6]

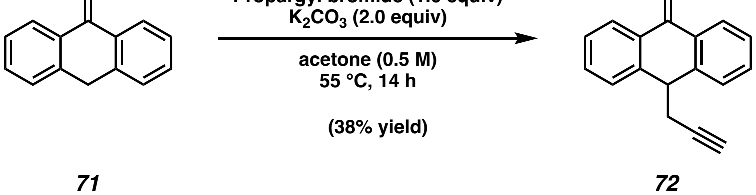

71 72

Alkyne 72. A 100 mL round bottom flask equipped with a stir bar was prepared a solution of anthrone (**71**) (1.00 g, 5.15 mmol, 1.0 equiv) in acetone (10.3 mL, 0.5 M). K_2CO_3 (1.42 g, 10.3 mmol, 2.0 equiv) added before propargyl bromide (80 wt% in toluene, 558 μL, 5.15 mmol, 1.0 equiv) was added via syringe in a single portion. The reaction flask was equipped with an air condenser, purged with N_2, and placed in a preheated aluminum heating mantle set at 55 °C. After 14 h, the solution was cooled to 23 °C before saturated aq. NH_4Cl (20 mL) was added. The aqueous layer was extracted with EtOAc (3 x 20 mL). The combined organic extracts were washed with brine (50 mL), dried over Na_2SO_4, filtered and concentrated under reduced pressure. The crude material was purified by automated chromatography (0 → 40% hexanes:EtOAc), followed by flash chromatography on silica get (0 → 20% hexanes:EtOAc) to afford alkyne **72** (457 mg, 1.97 mmol, 38% yield) as a yellow solid. **Alkyne 72**: R_f 0.21 (9:1 hexanes:EtOAc); ^{1}H NMR (500 MHz, $CDCl_3$) δ 8.31 (dd, J = 7.9, 1.5 Hz, 2H), 7.62 (ddd, J = 7.7, 7.2, 1.4 Hz, 2H), 7.56 (ddt, J = 7.6, 1.2, 0.6 Hz, 2H), 7.52 – 7.44 (m, 2H), 4.43 (t, J = 5.5 Hz, 1H), 2.81 (dd, J = 5.5, 2.6 Hz, 2H), 1.87 (t, J = 2.6 Hz, 1H). Spectral data matched those previously reported.[10]

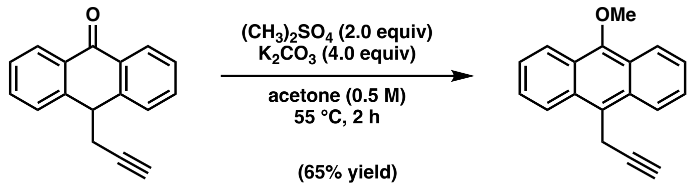

72 *73*

Anthracene 73. In a scintillation vial equipped with a stir bar was prepared a mixture of alkyne **72** (267 mg, 1.15 mmol, 1.0 equiv), K_2CO_3 (635 mg, 4.6 mmol, 4.0 equiv) and acetone (2.3 mL, 0.5 M). Dimethyl sulfate (220 μL, 2.30 mmol, 2.0 equiv) was then added via syringe in a single portion and the reaction flask was sealed with a Teflon-lined cap under ambient atmosphere then placed in preheated aluminum heating block set at 55 °C. After 2 h, the solution was cooled to 23 °C before deionized H_2O (10 mL) and EtOAc (10 mL) were added. The layers were separated, and the aqueous layer was further extracted with EtOAc (2 x 10 mL). The combined organic extracts were washed with brine (20 mL), dried over Na_2SO_4, filtered and concentrated under reduced pressure. The crude material was purified by recrystallization (boiling 5% hexanes:EtOAc) to give anthracene **73** (184 mg, 0.75 mmol, 65% yield) as a yellow solid. **Anthracene 73**: R_f 0.47 (9:1 hexanes:EtOAc); ^{1}H NMR (600 MHz, $CDCl_3$) δ 8.39 (d, J = 8.3, 2H), 8.32 (d, J = 8.8 Hz, 2H),

7.60 – 7.56 (m, 2H), 7.53 – 7.50 (m, 2H), 4.43 (d, J = 2.7 Hz, 2H), 4.14 (s, 3H), 2.08 (t, J = 2.7 Hz, 1H). Spectral data matched those previously reported.[11]

D. Diels–Alder Trapping Experiments of Cubene (10)

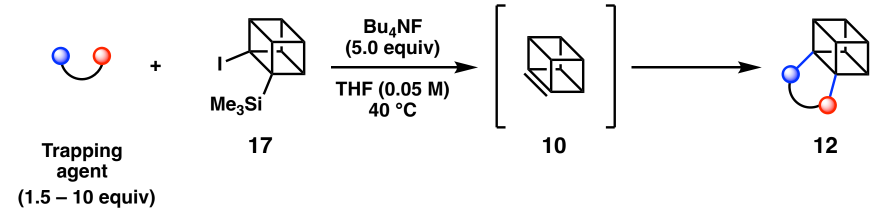

General Procedure 1 for the generation and trapping of cubene (10). To a 2-dram vial equipped with a stir bar was added cubene precursor **17** (30 mg, 0.10 mmol, 1.0 equiv), followed by the trapping agent (0.15-1.0 mmol, 1.5–10 equiv). The headspace of the reaction was purged with N_2 for 5 min, then THF (1.5 mL) and Bu_4NF (1.0 M in THF, 0.5 mL, 0.50 mmol, 5.0 equiv) were added sequentially via syringe in single portion. The vial was sealed with a Teflon-lined screw cap and stirred at 1000 rpm at 40 °C. After the specified reaction time, the reaction vessel was allowed to cool to 23 °C. The crude mixture was filtered through a 0.5 x 2 cm silica gel plug, eluting with EtOAc (10 mL). The eluate was subsequently concentrated under reduced pressure to dryness, and the crude material was analyzed by ^{1}H NMR spectroscopy. The sample for NMR analysis was then recombined with the crude residue and concentrated. Subsequent purification by flash column chromatography on silica gel or preparative TLC, with drying of the products under high vacuum, yielded cycloadducts **12**.

Any deviation from the above procedure is designated in the schemes below. All average yields reported for scope studies reflect the results of two isolation experiments with isolated yields within 10%.

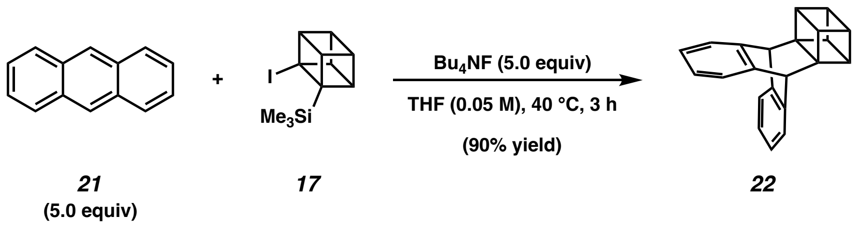

Cycloadduct 22. Followed General Procedure 1. Purification by automated column chromatography (0 → 5% hexanes:EtOAc) yielded cycloadduct **22** (90% yield, average of two experiments) as a white solid. **Cycloadduct 22**: R_f 0.52 (9:1 hexanes:EtOAc); mp: 210.5 °C; ^{1}H

NMR (600 MHz, $CDCl_3$): δ 7.28 (dd, *J* = 5.3, 3.3 Hz, 4H), 7.15 (dd, *J* = 5.4, 3.2 Hz, 4H), 4.39 (s, 2H), 3.83 – 3.80 (m, 2H), 3.81 – 3.30 (m, 4H); ^{13}C NMR (150 MHz, $CDCl_3$): δ 138.6, 126.1, 125.2, 52.7, 47.7, 45.2; IR (film): 2979, 1458, 774, 742, 622 cm^{-1}; HRMS-APCI (*m/z*) $[M+H]^+$ calcd for $C_{22}H_{17}^+$, 281.1324; found 281.1314.

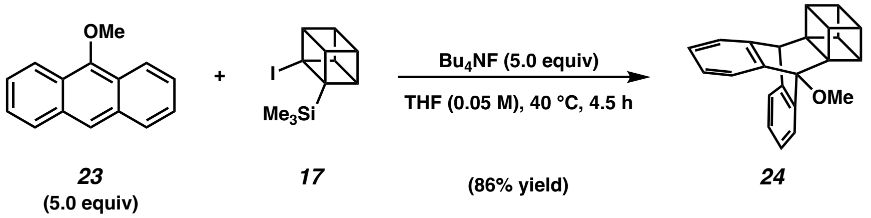

Cycloadduct 24. Followed General Procedure 1. Purification by flash column chromatography (10 → 33% hexanes:benzene) yielded cycloadduct **24** (86% yield, average of two experiments) as a white solid. **Cycloadduct 24**: R_f 0.15 (4:1 hexanes:benzene); mp 159 °C; ^{1}H NMR (600 MHz, $CDCl_3$): δ 7.58 – 7.54 (m, 2H), 7.28 (dd, *J* = 7.2, 1.3 Hz, 2H), 7.23 (td, *J* = 7.5, 1.3 Hz, 2H), 7.16 (td, *J* = 7.4, 1.3 Hz, 2H), 4.30 (s, 1H), 3.93 – 3.88 (m, 1H), 3.84 (dtd, *J* = 7.6, 5.2, 2.6 Hz, 1H), 3.78 (s, 3H), 3.51 (ddd, *J* = 5.3, 4.1, 2.4 Hz, 2H), 3.41 – 3.38 (m, 2H); ^{13}C NMR (150 MHz, $CDCl_3$): δ 139.4, 137.6, 126.24, 126.23, 124.7, 121.9, 82.5, 55.6, 54.6, 53.1, 46.9, 45.7, 45.6, 44.80, 44.78; IR (film): 2960, 1449, 1308, 968, 746 cm^{-1}; HRMS-APCI (*m/z*) $[M+H]^+$ calcd for $C_{23}H_{19}O^+$, 311.1430; found 311.1422.

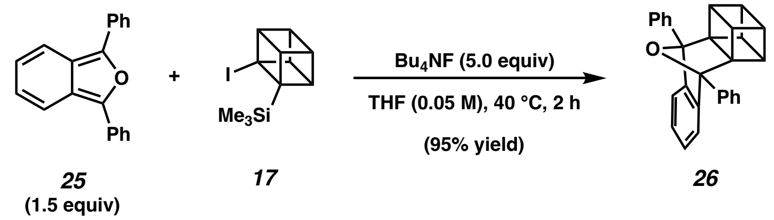

Cycloadduct 26. Followed General Procedure 1. Purification by automated column chromatography on basic alumina (0 → 20% hexanes:EtOAc) yielded cycloadduct **26** (95% yield, average of two experiments) as a pale yellow solid. **Cycloadduct 26**: R_f 0.76 (9:1 hexanes:EtOAc); mp: 158.3 °C; ^{1}H NMR (600 MHz, $CDCl_3$): δ 7.58 (d, *J* = 7.7 Hz, 4H), 7.48 – 7.44 (m, 4H), 7.41 – 7.37 (m, 2H), 7.18 (dd, *J* = 5.4, 3.0 Hz, 2H), 6.94 (dd, *J* = 5.4, 3.0 Hz, 2H), 4.07 – 4.03 (m, 2H), 3.93 (t, *J* = 3.3 Hz, 2H), 3.42 (t, *J* = 3.3 Hz, 2H); ^{13}C NMR (150 MHz, $CDCl_3$): δ 143.9, 136.9, 128.6, 128.1, 127.7, 127.2, 120.1, 91.9, 55.9, 45.9, 45.6, 44.9; IR (film): 2960, 1447, 1308, 969, 746 cm^{-1}; HRMS-APCI (*m/z*) $[M+H]^+$ calcd for $C_{28}H_{21}O^+$, 373.1586; found 373.1597.

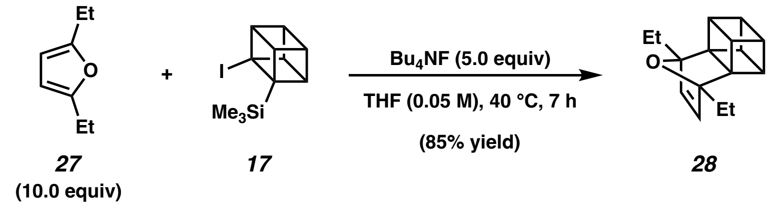

Cycloadduct 28. Followed General Procedure 1. Purification by automated column chromatography on neutral alumina (0 → 20% pentane:Et_2O) yielded cycloadduct **28** (85% yield, average of two experiments) as a colorless oil. **Cycloadduct 28**: R_f 0.52 (9:1 hexanes:EtOAc); ^{1}H NMR (600 MHz, $CDCl_3$): δ 6.10 (s, 2H), 3.94 – 3.91 (m, 2H), 3.76 – 3.75 (m, 2H), 3.43 – 3.42 (m, 2H), 1.99 – 1.86 (m, 4H), 1.03 (t, J = 7.5 Hz, 6H); ^{13}C NMR (150 MHz, $CDCl_3$): δ 131.9, 91.2, 53.5, 45.6, 45.2, 43.4, 23.1, 10.0; IR (film): 2963, 1463, 1207, 918, 720 cm^{-1}; HRMS-APCI (*m/z*) $[M+H]^+$ calcd for $C_{16}H_{19}O^+$, 227.1430; found 227.1427.

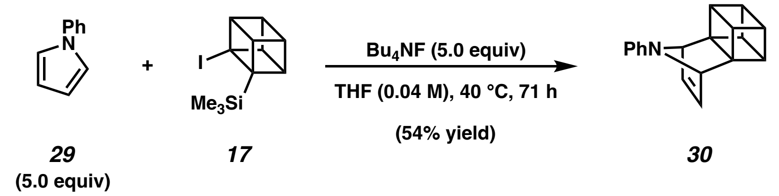

Cycloadduct 30. Followed a modified version of General Procedure 1 by altering the amount of THF (0.04 M, 2.0 mL). Purification by flash column chromatography (pentane → 5% hexanes:EtOAc) afforded cycloadduct **30** (54% yield, average of two experiments) as a white solid. **Cycloadduct 30**: R_f 0.36 (9:1 hexanes:EtOAc); mp: 99–101 °C; ^{1}H NMR (600 MHz, $CDCl_3$): δ 7.14 (dd, J = 8.6, 7.2 Hz, 2H), 6.84 (d, J = 7.6 Hz, 2H), 6.79 (tt, J = 7.3, 1.1 Hz, 1H), 6.03 (br s, 2H), 4.83 (br s, 2H), 3.94 – 3.90 (m, 2H), 3.73 – 3.69 (m, 2H), 3.49 – 3.45 (m, 2H); ^{13}C NMR (150 MHz, $CDCl_3$): δ 147.6, 128.7, 127.9, 120.1, 118.1, 65.8, 49.2, 46.5, 45.8, 45.7; IR (film): 2977, 1598, 1496, 1312, 691 cm^{-1}; HRMS-APCI (*m/z*) $[M+H]^+$ calcd for $C_{18}H_{16}N^+$, 246.1277; found 246.1277.

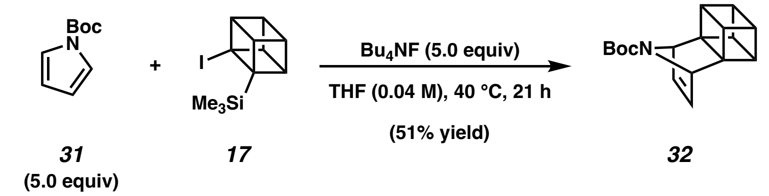

Cycloadduct 32. Followed a modified version of General Procedure 1 by altering the amount of THF (0.04 M, 2.0 mL). Purification by flash column chromatography (0 → 2% hexanes:EtOAc) afforded cycloadduct **32** (51% yield, average of two experiments) as an off-white solid.

Cycloadduct 32: R*f* 0.20 (19:1 hexanes:EtOAc); mp: 95–97 °C; ^{1}H NMR (600 MHz, $CDCl_3$): δ 6.10 – 6.00 (m, 2H), 4.94 – 4.74 (m, 2H), 3.94 – 3.89 (m, 2H), 3.73 – 3.70 (m, 2H), 3.46 – 3.40 (m, 2H), 1.41 (s, 9H); ^{13}C NMR (150 MHz, $CDCl_3$): δ 155.5, 129.2, 128.7, 79.8, 62.5, 62.0, 49.3, 49.2, 45.9, 45.7, 45.5, 45.3, 45.2, 28.4; IR (film): 2976, 1697, 1365, 1172, 866 cm^{-1}; HRMS-APCI (*m/z*) [M+H]$^+$ calcd for $C_{17}H_{20}NO_2^+$, 270.1489; found 270.1480.

*Note: **32** was obtained as a mixture of rotamers. These data represent empirically observed chemical shifts from the ^{1}H and ^{13}C NMR spectra.*

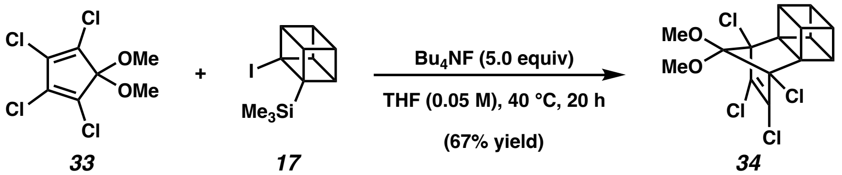

33 (5.0 equiv) *17* *34*

Cycloadduct 34. Followed General Procedure 1 with a modified purification procedure. After the specified reaction time, the reaction vessel was allowed to cool to 23 °C. The crude mixture was filtered through a 0.5 x 2 cm silica gel plug, eluting with EtOAc (10 mL). The eluate was subsequently concentrated under reduced pressure to dryness, and the crude material was analyzed by ^{1}H NMR spectroscopy. Excess 5,5-Dimethyl-1,2,3,4-tetrachlorocyclopentadine was removed by reacting with 4-phenyl-1,2,3-triazoline-3,5-dione. To the scintillation vial which contained the crude reaction mixture was added a stir bar, 4-phenyl-1,2,3-triazoline-3,5-dione (122 mg, 6.96 mmol, 7 equiv), then CH_2Cl_2 (1 mL). After stirring at 23 °C for 3 h, mixture was concentrated under reduced pressure to dryness. Purification of the residue by automated column chromatography (0 → 40% hexanes:CH_2Cl_2) then preparatory TLC (20% hexanes:CH_2Cl_2) yielded cycloadduct **34** (67% yield, average of two experiments) as a white solid. **Cycloadduct 34**: R*f* 0.45 (4:1 hexanes:CH_2Cl_2); mp 142.8 °C; ^{1}H NMR (600 MHz, $CDCl_3$): δ 4.07 – 4.03 (m, 2H), 3.96 – 3.93 (m, 2H), 3.73 – 3.71 (m, 2H), 3.57 (s, 3H), 3.55 (s, 3H); ^{13}C NMR (150 MHz, $CDCl_3$): δ 126.6, 112.7, 78.6, 57.3, 52.5, 52.2, 47.2, 45.6, 45.5; IR (film): 2973, 1591, 1464, 1183, 953, 867 cm^{-1}; HRMS-APCI (*m/z*) [M+H]$^+$ for calcd $C_{15}H_{13}Cl_4O_2^+$, 364.9664; found 364.9669.

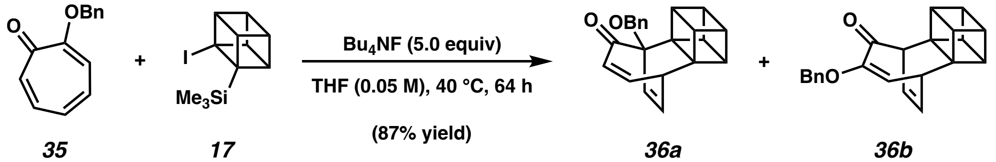

35 (5.0 equiv) *17* *36a* *36b*

Cycloadduct 36a and **36b.** Followed General Procedure 1. Regioselectivity ratio (r.r.) was determined by integration of the ^{1}H NMR spectrum of the crude reaction mixture (10:1 **36a**:**36b**, average of 2 experiments). Purification by automated column chromatography (0 → 40% hexanes:EtAOc) yielded an inseparable mixture of cycloadducts **36a** and **36b** (87% yield, average of two experiments) as a colorless solid. Characterization was necessarily performed on the mixture of isomers, with only the discernable peaks for **36b** tabulated below. **Cycloadducts 36a** and **36b**: R_f 0.30 (9:1 hexanes:EtOAc); ^{1}H NMR (600 MHz, $CDCl_3$, **36a**): δ 7.52 – 7.48 (m, 2H), 7.38 – 7.32 (m, 2H), 7.30 – 7.26 (m, 1H), 6.87 (dd, *J* = 11.4, 8.1 Hz, 1H), 6.24 (dd, *J* = 9.0, 6.8 Hz, 1H), 6.03 (d, *J* = 11.4, 1H), 5.83 (d, *J* = 9.0 Hz, 1H), 4.91 (d, *J* = 10.4, 1H), 4.36 (d, *J* = 10.4, 1H), 4.08 – 4.03 (m, 1H), 3.96 (ddq, *J* = 7.5, 5.1, 2.4 Hz, 1H), 3.88 (tq, *J* = 4.4, 2.2 Hz, 1H), 3.85 (tt, *J* = 4.7, 2.2 Hz, 1H), 3.77 (tt, *J* = 4.6, 2.2 Hz, 1H), 3.69 (tt, *J* = 4.6, 2.1 Hz, 1H), 3.50 (dd, *J* = 8.1, 6.9 Hz, 1H); ^{1}H NMR (600 MHz, $CDCl_3$, **36b**): δ 7.32 (m, 4H), 6.45 (dd, *J* = 8.1, 7.2 Hz, 1H), 5.97 (d, *J* = 9.1 Hz, 1H), 5.94 (m, 1H), 4.81 (d, *J* = 12.7 Hz, 1H), 3.64 – 3.61 (m, 1H), 3.56 – 3.53 (m, 2H), 3.42 (dd, *J* = 9.2, 7.0 Hz, 1H), 3.34 (m, 1H); ^{13}C NMR (150 MHz, $CDCl_3$, **36a** and **36b**): δ 193.4, 190.5, 152.0, 148.6, 139.0, 136.8, 134.4, 131.1, 130.8, 129.9, 128.6, 128.4, 128.1, 127.9, 127.7, 127.2, 123.7, 121.8, 89.0, 70.4, 68.0, 56.1, 54.3, 54.0, 52.7, 49.7, 48.2, 47.12, 47.06, 46.99, 46.91, 46.86, 46.4, 46.0, 45.9, 45.31, 45.25, 45.20, 45.1, 43.6, 39.6, 37.2; IR (film): 2976, 1669, 1204, 1119, 1078 cm^{-1}; HRMS-APCI (*m/z*) $[M+H]^+$ calcd for $C_{22}H_{19}O_2^+$, 315.1380; found 315.1371.

The structure of **36a** was verified by 2D-NOESY, as the following interaction was observed:

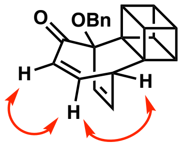

36a

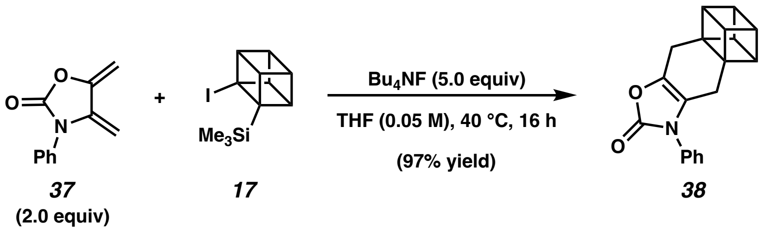

Cycloadduct 38. Followed General Procedure 1. Purification by automated column chromatography (0 → 20% hexanes:EtOAc) yielded cycloadduct **38** (97% yield, average of two

experiments) as an off white solid. **Cycloadduct 38**: R*f* 0.12 (9:1 hexanes:EtOAc); mp: 161.9 °C; ^{1}H NMR (500 MHz, $CDCl_3$): δ 7.45 (t, *J* = 7.8 Hz, 2H), 7.37 – 7.31 (m, 3H), 4.02 – 3.97 (m, 1H), 3.97–3.92 (m, 1H), 3.87 – 3.82 (m, 2H), 3.82 – 3.77 (m, 2H), 2.76 (t, *J* = 2.0 Hz, 2H), 2.58 (t, *J* = 2.0 Hz, 2H); ^{13}C NMR (125 MHz, $CDCl_3$): δ 154.9, 134.2, 133.9, 129.5, 127.9, 126.0, 119.4, 51.8, 50.7, 49.69, 49.68, 45.4, 45.3, 26.0, 25.9; IR (film): 2971, 2840, 1755, 1501, 1387 cm^{-1}; HRMS-APCI (*m/z*) $[M+H]^+$ calcd for $C_{19}H_{16}NO_2^+$, 290.1175; found 290.1183.

E. Additional Reactivity of 10 and Elaboration of Cubene Adducts

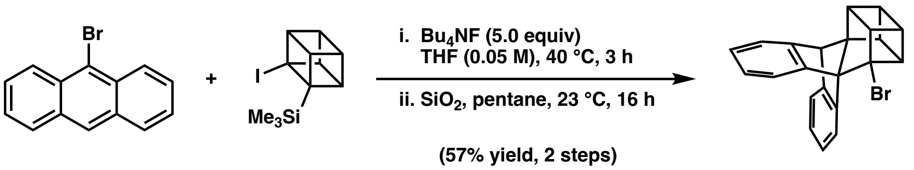

74 (5.0 equiv) *17* *39*

Cycloadduct 39. To a 2-dram vial equipped with a stir bar was added iodide **17** (14.9 mg, 49.3 µmol, 1.0 equiv) and 9-bromoanthracene (**74**) (66.0 mg, 247 µmol, 5.0 equiv). The headspace of the reaction was then purged with nitrogen for 5 min then THF (0.75 mL) and Bu_4NF (1.0 M in THF, 0.25 mL, 247 µmol, 5.0 equiv) were added sequentially via syringe in one portion. The vial was sealed with a Teflon-lined screw cap and stirred at 1000 rpm at 40 °C for 3 h. Once the reaction was complete as determined by TLC analysis, the reaction vessel was allowed to cool to 23 °C. The reaction mixture was filtered through a 0.5 x 2 cm silica gel plug, eluting with EtOAc (10 mL). The eluate was subsequently concentrated to dryness and then filtered through a 0.5 x 2 cm silica gel plug, eluting with CH_2Cl_2 (10 mL). The eluate was subsequently concentrated to dryness and suspended in pentane and placed under a stream of N_2. Silica gel (200 mg) was added in a single portion and the mixture was stirred at 23 °C for 16 h. Once the reaction was complete as determined by TLC analysis, the reaction was filtered over cotton, eluting with CH_2Cl_2 (10 mL). The eluate was concentrated under reduced pressure. The crude product was purified by flash chromatography (pentane) to afford cycloadduct **39** (10.1 mg, 28.1 µmol, 57% yield, two steps) as a white solid. Crystals suitable for X-ray diffraction studies were obtained by dissolving **39** in minimal amount of diethyl ether in 1-dram vial, followed by slow evaporation of solvent. **Cycloadduct 39**: R*f* 0.35 (19:1 hexanes:EtOAc); mp: >210 °C; ^{1}H NMR (600 MHz, $CDCl_3$): δ 7.61 – 7.55 (m, 2H), 7.29 – 7.25 (overlapping with solvent peak, 2H), 7.06 – 7.00 (m, 4H), 4.30

(s, 1H), 3.84 – 3.77 (m, 2H), 3.53–3.48 (br s, 1H), 3.14–3.06 (m, 3H); ^{13}C NMR (150 MHz, $CDCl_3$): δ 151.7, 142.2, 126.0, 125.6, 123.1, 122.8, 92.0, 86.8, 61.8, 58.6, 50.1, 45.1, 41.7, 35.2; IR (film): 2997, 1437, 1122, 854, 750 cm^{-1}; HRMS-APCI (*m/z*) $[M+H]^+$ calcd for $C_{22}H_{16}Br^+$, 359.0430; found 359.0423. Structure of **39** was confirmed by X-ray crystallography.

Crystal Structure Analysis for 39

A colorless, plate-shaped crystal was mounted on the goniometer. Data were collected from a shock-cooled single crystal at 100(2) K on a Bruker D8 three-axis goniometer three-circle diffractometer with a sealed X-ray tube using a Triumph as monochromator and a Bruker Apex-II CCD detector. The diffractometer was equipped with an Oxford Cryostream 700 low temperature device and used MoK_α radiation (λ = 0.71073 Å). All data were integrated with SAINT V8.41 and a multi-scan absorption correction using TWINABS Bruker was applied.[12,13] The structure was solved by direct methods with SHELXT 2018/2 and refined by full-matrix least-squares methods against F^2 using SHELXL-2019/2.[14,15] All non-hydrogen atoms were refined with anisotropic displacement parameters. All hydrogen atoms were refined isotropic on calculated positions using a riding model with their U_{iso} values constrained to 1.5 times the U_{eq} of their pivot atoms for terminal sp^3 carbon atoms and 1.2 times for all other carbon atoms. CIF file was generated using FinalCif.[16] Refined on single component and de-twinned composite reflections using HKLF 5. All non-hydrogen atoms were refined without distance restraints or constraints and using a global RIGU restraint. All hydrogen atoms were refined on riding positions. Deposition Number 2446782 contains the supplementary crystallographic data for compound **39**. These data are provided free of charge by the joint Cambridge Crystallographic Data Centre and Fachinformationszentrum Karlsruhe Access Structures service www.ccdc.cam.ac.uk/structures.

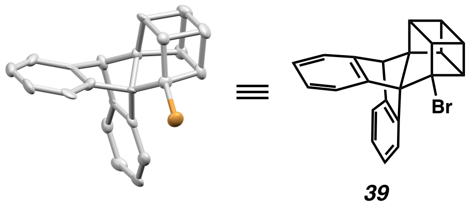

39

Fig. 6. ORTEP representation of X-ray crystallographic structure **39** (CCDC Registry # 2446782) with the ellipsoids shown at 85% probability level. Carbon – grey, hydrogen – omitted for clarity, bromine – orange.

From the CIF/PLATON check, the following alerts for compound **39** were reported.

Alert level B

```
PLAT213_ALERT_2_B Atom C17 has ADP max/min Ratio .... 4.7 oblate
```

Author Response: This error is likely caused by underweighting of the BASF. This is also evidenced by a large q peak corresponding to the bromine of the other domain, which could be refined as full molecule disorder instead.

Table 1. Crystal data and structure refinement for compound **39**.

CCDC number	2446782
Empirical formula	$C_{22}H_{15}Br$
Formula weight	359.25
Temperature [K]	100(2)
Crystal system	monoclinic
Space group (number)	$C2$ (5)
a [Å]	15.741(3)
b [Å]	7.2853(13)
c [Å]	13.637(2)
α [°]	90
β [°]	106.650(3)
γ [°]	90
Volume [Å^3]	1498.4(5)
Z	4
ρ_{calc} [gcm^{-3}]	1.593
μ [mm^{-1}]	2.740
F(000)	728
Crystal size [mm^3]	0.054×0.136×0.298
Crystal color	colorless
Crystal shape	plate
Radiation	MoK_α (λ=0.71073 Å)
2θ range [°]	5.40 to 52.74 (0.80 Å)
Index ranges	$-19 \le h \le 19$<br>$-9 \le k \le 9$<br>$-16 \le l \le 17$
Reflections collected	3072
Independent reflections	3072<br>R_{int} = 0.0429<br>R_{sigma} = 0.0495
Completeness to θ = 25.242°	99.9 %
Data / Restraints / Parameters	3072 / 243 / 209
Absorption correction T_{min}/T_{max} (method)	0.6104 / 0.7457 (multi-scan)
Goodness-of-fit on F^2	1.065
Final R indexes [$I \geq 2\sigma(I)$]	R_1 = 0.0382<br>wR_2 = 0.0877
Final R indexes [all data]	R_1 = 0.0486<br>wR_2 = 0.0939
Largest peak/hole [eÅ^{-3}]	1.83/−0.48
Flack X parameter	-0.056(10)

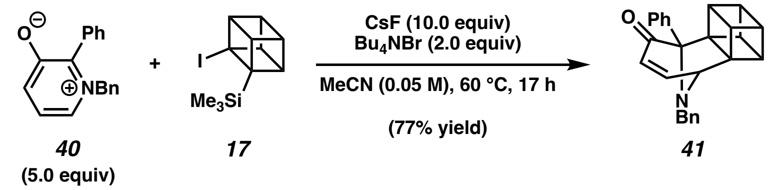

Cycloadduct 41. To a 2-dram vial was added oxidopyridinium **40** (134.0 mg, 0.51 mmol, 5 equiv) which was dried under high vacuum for 30 min before use. Iodide **17** (30.9 mg, 0.10 mmol, 1.0 equiv), CsF (155.0 mg, 1.02 mmol, 10 equiv), Bu_4NBr (65.9 mg, 0.20 mmol, 2 equiv) and an oven dried stir bar were sequentially added. The headspace of the reaction was then purged with nitrogen for 5 min, then MeCN (2 mL, 0.05 M) was added. The vial was sealed with a Teflon-lined screw cap and stirred at 1000 rpm at 60 °C for 17 h. Once the reaction was complete as determined by TLC analysis, the reaction vessel was allowed to cool to 23 °C. The reaction mixture was filtered through a 0.5 x 2 cm silica gel plug, eluting with EtOAc (10 mL). The eluate was concentrated under reduced pressure. The crude product was purified by flash column chromatography (0 → 10% hexanes:EtOAc) to afford cycloadduct **41** (28.5 mg, 78.4 μmol, 77% yield) as a yellow oil.

Cycloadduct 41: R_f 0.25 (9:1 hexanes:EtOAc); ^{1}H NMR (600 MHz, $CDCl_3$): δ 7.67 – 7.62 (m, 2H), 7.40 – 7.29 (m, 6H), 7.29 – 7.22 (overlapped with solvent peak, 2H), 6.87 (dd, *J* = 9.7, 5.1 Hz, 1H), 6.28 (d, *J* = 9.7 Hz, 1H), 4.07 (d, *J* = 5.1 Hz, 1H), 4.03 (ddt, *J* = 5.7, 3.9, 1.9 Hz, 1H), 3.99 (qt, *J* = 5.2, 2.4 Hz, 1H), 3.95 (tq, *J* = 5.2, 2.5 Hz, 1H), 3.74 – 3.67 (m, 4H), 3.60 (br s, 1H); ^{13}C NMR (150 MHz, $CDCl_3$) δ 194.6, 146.4, 139.8, 136.5, 130.6, 128.6, 128.5, 128.1, 127.9, 127.1, 127.0, 79.8, 60.3, 58.1, 55.2, 50.3, 48.3, 46.9, 46.84, 46.81, 46.2, 45.7; IR (film): 2981, 1674, 907, 725, 699 cm^{-1}; HRMS-APCI (*m/z*) $[M+H]^+$ calcd for $C_{26}H_{21}NO^+$, 364.1696; found 364.1680.

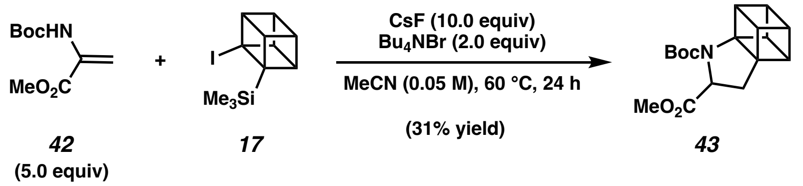

Cycloadduct 43. To a 2-dram vial equipped with a stir bar was added iodide **17** (30.9 mg, 0.10 mmol, 1.0 equiv), ene carbamate **42** (103.0 mg, 0.51 mmol, 5 equiv), CsF (155 mg, 1.02 mmol, 10 equiv) and Bu_4NBr (65.9 mg, 0.20 mmol, 2 equiv). The headspace of the reaction was then purged with nitrogen for 5 min, and then MeCN (2 mL, 0.05 M) was added. The vial was sealed with a Teflon-lined screw cap and stirred at 1000 rpm at 60 °C for 24 h. Once the reaction was

complete as determined by TLC analysis, the reaction vessel was allowed to cool to 23 °C. The reaction mixture was filtered through a 0.5 x 2 cm silica gel plug, eluting with EtOAc (10 mL). The eluate was concentrated under reduced pressure. The crude product was purified by preparatory TLC (6:1 hexanes:EtOAc) to yield cycloadduct **43** (9.7 mg, 32.0 µmol, 31% yield) as a yellow oil. **Cycloadduct 43**: R_f 0.40 (6:1 hexanes:EtOAc); ^{1}H NMR (500 MHz, $CDCl_3$) δ 4.58 (ddd, *J* = 46.8, 9.2, 2.4 Hz, 1H), 4.33 – 4.05 (m, 2H), 3.97 – 3.81 (m, 2H), 3.75 (d, *J* = 10.0 Hz, 1H), 3.72 (br s, 4H), 2.55 – 2.42 (m, 1H), 2.26 (dd, *J* = 15.0, 2.4 Hz, 1H), 1.43 (d, *J* = 8.4 Hz, 9H); ^{13}C NMR (125 MHz, $CDCl_3$) δ 174.2, 174.0, 154.7, 153.6, 80.7, 80.1, 72.4, 71.7, 65.3, 64.6, 56.7, 55.9, 54.4, 53.9, 52.5, 52.4, 52.3, 52.2, 51.5, 48.20, 48.15, 47.8, 47.7, 45.8, 45.5, 45.3, 42.1, 41.7, 35.8, 35.3, 28.5, 28.4; IR (film): 2980, 1747, 1381, 1167, 728 cm^{-1}; HRMS-APCI (*m/z*) $[M+H]^+$ calcd for $C_{17}H_{22}NO_4^+$, 304.1543; found 304.1544.

*Note: **43** was obtained as a mixture of rotamers. These data represent empirically observed chemical shifts from the ^{1}H and ^{13}C NMR spectra.*

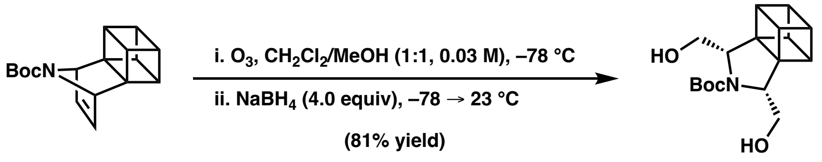

32 *44*

Diol 44. To a scintillation vial equipped with a stir bar was added cycloadduct **32** (16.2 mg, 60.1 µmol, 1.0 equiv). The headspace of the reaction was then purged with nitrogen for 5 min, and then CH_2Cl_2 (1.0 mL) and MeOH (1.0 mL) were added. The reaction was cooled to –78 °C. A stream of O_3 was bubbled through the solution for 0.5 h until persistence of blue color (indicating consumption of starting material). The mixture was then sparged with N_2 until the blue color disappeared before $NaBH_4$ (9.1 mg, 241 µmol, 4.0 equiv) was added in one portion. The vial was sealed with a Teflon-lined cap and allowed to warm to 23 °C over 2.5 h. Once the reaction was complete as determined by TLC analysis, the reaction was diluted with CH_2Cl_2 (25 mL) and washed with a 10% aqueous solution of citric acid (25 mL). The organic layer was separated and concentrated under reduced pressure. The crude product was purified by flash column chromatography (1 → 5% CH_2Cl_2:MeOH) to afford diol **44** (14.8 mg, 48.5 µmol, 81% yield) as a colorless oil. **Diol 44**: R_f 0.21 (19:1 CH_2Cl_2:MeOH); ^{1}H NMR (600 MHz, $CDCl_3$): δ 4.38 – 3.77 (m, 10H), 3.70 – 3.60 (m, 2H), 1.48 (s, 9H); ^{13}C NMR (150 MHz, $CDCl_3$): δ 156.5, 80.8, 65.0,

64.2, 63.4, 59.4, 58.2, 49.4, 47.6, 47.1, 45.4, 28.5; IR (film): 3359, 2977, 1660, 1391, 1366 cm^{-1}; HRMS-APCI (*m/z*) $[M+H]^+$ calcd for $C_{17}H_{24}NO_4^+$, 306.1700; found 306.2694.

*Note: **44** was obtained as a mixture of rotamers. These data represent empirically observed chemical shifts from the ^1H and ^{13}C NMR spectra.*

F. Diels–Alder Trapping Experiments of 1,7-Quadricyclene (11)

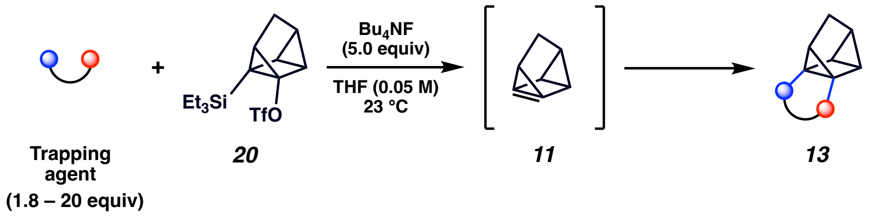

General Procedure 2 for the generation and trapping of 1,7-quadricyclene (11). To a 2-dram vial containing a stirred solution of silyl triflate **20** (35.5 mg, 0.10 mmol, 1.0 equiv) and trapping agent (1.8–20 equiv) in THF (1.5 mL) was added Bu_4NF (1.0 M in THF, 0.50 mmol, 5.0 equiv) in one portion. The septa cap was sealed with melted paraffin wax and the reaction mixture was stirred at 800–1200 RPM at 23 °C. After a certain reaction time, saturated aq. NH_4Cl (2 mL) was added to quench the reaction. The crude mixture was extracted with diethyl ether/pentane (1:1, 3 x 2 mL) and the organic extracts are dried over Na_2SO_4. The crude mixture was concentrated under a reduced pressure to dryness, and the crude material was analyzed by ^1H NMR spectroscopy. The sample for NMR analysis was then recombined with the crude residue, concentrated, and purified by chromatography and dried under high vacuum to yield cycloadducts **13**.

Note: Sealing of the reaction vessel with paraffin wax was taken as an extra precaution, but not required for the success of the reaction.

Any deviation from the above procedure is designated in the schemes below. All average yields reported for scope studies reflect the results of two isolation experiments with isolated yields within 10%.

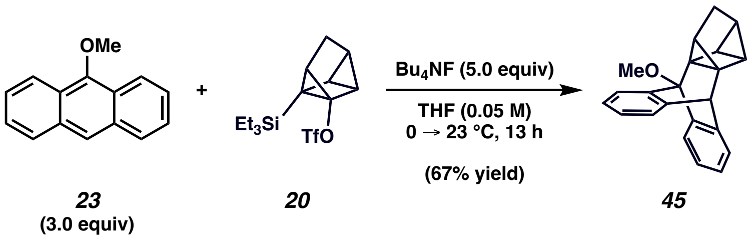

Cycloadduct 45. Followed a modified version of General Procedure 2. Reaction was carried out at 0 °C, then slowly warmed up to 23 °C. Purification by flash column chromatography (10:89:1 → 20:79:1 benzene:hexanes:NEt_3) provided cycloadduct **45**[17] (67% yield, average of two experiments) as a colorless film. **Cycloadduct 45**: R_f 0.32 (4:1 hexanes:benzene); ^{1}H NMR (600 MHz, C_6D_6): δ 8.01 (d, J = 7.4 Hz, 1H), 7.45 (d, J = 7.5 Hz, 1H), 7.23 (d, J = 7.3 Hz, 1H), 7.16 – 7.14 (overlapped with solvent peak, 1H), 7.04 (td, J = 7.4, 1.3 Hz, 1H), 7.01 – 6.96 (m, 2H), 6.92 (td, J = 7.4, 1.3 Hz, 1H), 4.15 (s, 1H), 3.59 (s, 3H), 1.89 (dt, J = 11.4, 1.5 Hz, 1H), 1.85 (dt, J = 11.4, 1.5 Hz, 1H), 1.30 (br s, 1H), 1.29 (t, J = 4.7 Hz, 1H), 1.21 (t, J = 4.7 Hz, 1H), 1.19 – 1.17 (m, 1H); ^{13}C NMR (150 MHz, C_6D_6): δ 146.1, 145.1, 142.6, 140.6, 126.3, 126.2, 126.1, 125.7, 124.5, 123.7, 122.4, 121.2, 84.9, 54.4, 44.7, 40.7, 33.6, 32.1, 27.9, 23.1, 13.4, 12.1; IR (film): 3066, 2930, 1721, 1456, 1218 cm^{-1}; HRMS-APCI (*m/z*) $[M+H]^+$ calcd for $C_{22}H_{19}O^+$, 299.1430; found 299.1428.

*Note: Cycloadduct **45** was found to be sensitive to unbuffered silica gel.*

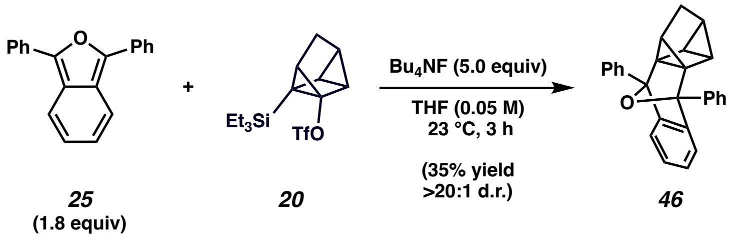

Cycloadduct 46. Followed General Procedure 2. Following concentration under reduced pressure, the crude residue was obtained (>20:1 d.r., average of two experiments). Purification by automated column chromatography (0 → 10% hexanes:EtOAc) provided cycloadduct **46** (35% yield; average of two experiments) as a yellow oil. **Cycloadduct 46**: R_f 0.48 (20:1 hexanes:EtOAc); ^{1}H NMR (600 MHz, $CDCl_3$): δ 7.60 (d, J = 7.2 Hz, 4H), 7.46 – 7.36 (m, 8H), 7.14 – 7.10 (m, 2H), 2.92 (br s, 1H), 2.42 (br s, 2H), 1.71 – 1.65 (m, 1H), 1.34 (d, J = 5.0 Hz, 2H); ^{13}C NMR (125 MHz, $CDCl_3$): δ 149.9, 137.1, 128.8, 128.6, 128.4, 126.6, 120.8, 91.9, 45.1, 34.7, 34.4, 23.8, 13.8; IR (film):

3057, 2932, 1689, 1444, 965 cm^{-1}; HRMS-APCI (*m/z*) $[M+H]^+$ calcd for $C_{27}H_{21}O^+$, 361.1587; found 361.1582.

The structure of cycloadduct **46** was verified by 2D-NOESY, as the following interactions were observed:

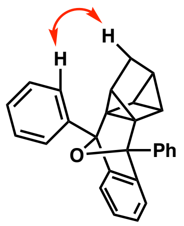

46

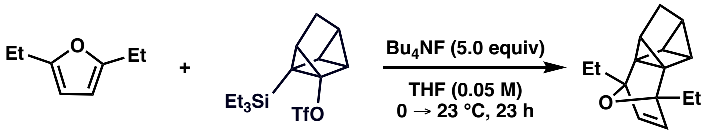

27 (20.0 equiv) ***20*** ***47***

Cycloadduct 47. Followed a modified version of General Procedure 2. Reaction was carried out at 0 ºC, then slowly warmed up and stirred at 23 ºC. Concentration of crude reaction mixture was performed under a rapid flow of N_2 stream to afford crude product. Yield of cycloadduct **47** (56% yield, >20:1 d.r., average of two experiments) was determined by integration of ^{1}H NMR of crude reaction mixture using mesitylene as an external standard. An analytical sample of cycloadduct **47** was obtained by flash column chromatography using basic alumina (5:94:1 diethyl ether:pentane:NEt_3), followed by carefully concentrating the fractions under mild vacuum (>300 mbar) to give cycloadduct **47** as a colorless oil. **Cycloadduct 47**: R_f 0.52 (10:1 pentane:diethyl ether on basic Al_2O_3); ^{1}H NMR (600 MHz, C_6D_6): δ 6.50 (s, 2H), 2.90 (m, 1H), 2.15 (t, J = 1.5 Hz, 2H), 1.91 (q, J = 7.5 Hz, 4H), 1.45 (ddt, J = 6.5, 4.8, 1.5 Hz, 1H), 1.38 (d, J = 5.2 Hz, 2H), 1.04 (t, J = 7.5 Hz, 6H); ^{13}C NMR (150 MHz, C_6D_6): δ 139.3, 91.6, 48.1, 35.5, 34.9, 25.6, 24.5, 14.2, 9.9; IR (film): 3057, 2962, 2929, 2856, 1458 cm^{-1}; HRMS-APCI (*m/z*) $[M+H]^+$ calcd for $C_{15}H_{19}O^+$: 215.1430; found 215.1429.

*Note: Cycloadduct **47** was found to be very sensitive to silica gel and was moderately sensitive to basic alumina even after triethylamine buffering. As the result, it is empirically observed that the column purification of **47** needs to be completed within 5 minutes.*

The structure of **47** was verified by 2D-NOESY, as the following interaction was observed:

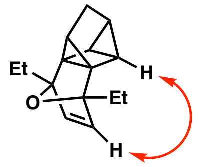

47

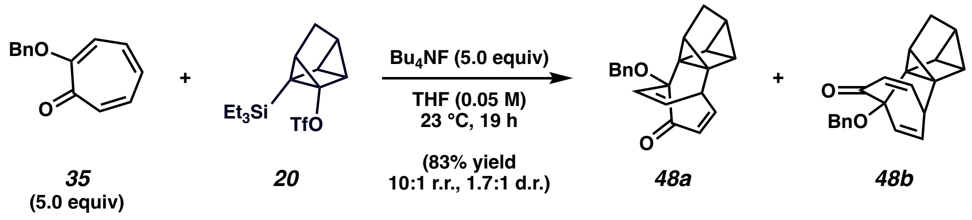

Cycloadducts 48a and **48b**. Followed General Procedure 2. Regioselectivity ratio (r.r.) and diastereoselectivity ratio (d.r.) were determined by integration of the ^{1}H NMR spectrum of crude reaction mixture (10:1 r.r. (**48a+48b**): minor regioisomer (not shown), 1.7:1 d.r. for **48a:48b**, average of 2 experiments). Purification by flash column chromatography (10% hexanes:EtOAc) provided cycloadducts **48a** and **48b** (83% yield, average of two experiments) as a colorless oil. Analytical samples of pure **48a** and **48b** were obtained via preparative TLC (90:5:5 hexanes:diethyl ether:CH_2Cl_2). **Cycloadduct 48a**: R_f 0.18 (10:1 hexanes:EtOAc); ^{1}H NMR (600 MHz, C_6D_6): δ 7.55 (d, J = 7.5 Hz, 2H), 7.20 (t, J = 7.7 Hz, 2H), 7.11 (t, J = 7.7 Hz, 1H), 6.49 (dd, J = 11.1, 7.2 Hz, 1H), 6.10 (dd, J = 11.1, 0.4 Hz, 1H), 5.69 (d, J = 9.1 Hz, 1H), 5.57 (dd, J = 9.1, 6.8 Hz, 1H), 5.04 (d, J = 11.7 Hz, 1H), 4.61 (d, J = 11.7 Hz, 1H), 2.65 (t, J = 7.0 Hz, 1H), 2.10 (d, J = 1.2 Hz, 1H), 1.85 – 1.82 (m, 1H), 1.67 – 1.64 (m, 1H), 1.55 (t, J = 4.8 Hz, 1H), 1.29 (t, J = 4.8 Hz, 1H), 1.23 (tq, J = 5.0, 1.5 Hz, 1H); ^{13}C NMR (150 MHz, C_6D_6): δ 193.6, 150.7, 140.3, 133.8, 132.7, 130.6, 128.4, 128.0, 127.4, 88.8, 68.2, 38.9, 36.1, 34.4, 32.7, 27.0, 24.9, 15.9, 15.5; IR (film): 3061, 2927, 2858, 1676, 1119 cm^{-1}; HRMS-APCI (*m/z*) $[M+H]^+$ calcd for $C_{21}H_{19}O_2^+$, 303.1380; found 303.1376; **Cycloadduct 48b:** R_f 0.29 (10:1 hexanes:EtOAc); ^{1}H NMR (600 MHz, C_6D_6): δ 7.63 (d, J = 7.5 Hz, 2H), 7.22 (t, J = 7.6 Hz, 2H), 7.15 – 7.11 (overlapped with solvent peak, 1H), 6.31 (d, J = 8.3 Hz, 1H), 6.18 (dd, J = 8.3, 6.3 Hz, 1H), 5.95 (dd, J = 11.2, 8.6 Hz, 1H), 5.65 (d, J = 11.1 Hz, 1H), 5.26 (d, J = 10.9 Hz, 1H), 4.46 (d, J = 10.9 Hz, 1H), 2.85 (dd, J = 8.6, 6.3 Hz, 1H), 1.94 – 1.86 (m, 3H), 1.68 (t, J = 4.7 Hz, 1H), 1.24 (dt, J = 4.7, 1.5 Hz, 1H), 1.20 (t, J = 4.7 Hz, 1H); ^{13}C NMR (150 MHz, C_6D_6): δ 192.4, 147.7, 140.0,

137.3, 134.5, 130.5, 128.6, 128.5, 127.6, 90.2, 67.8, 37.9, 37.1, 33.9, 27.2, 25.9, 22.9, 13.8, 11.8; IR (film): 3055, 2927, 2856, 1674, 1109 cm^{-1}; HRMS-APCI (*m/z*) [M+H]$^+$ calcd for $C_{21}H_{19}O_2^+$, 303.1380; found 303.1376.

The structure of **48a** and **48b** were verified by 2D-NOESY, as the following interactions were observed:

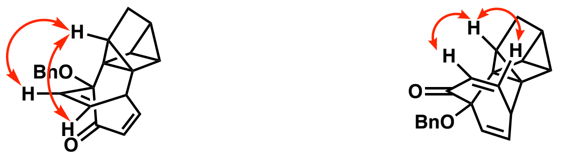

48a *48b*

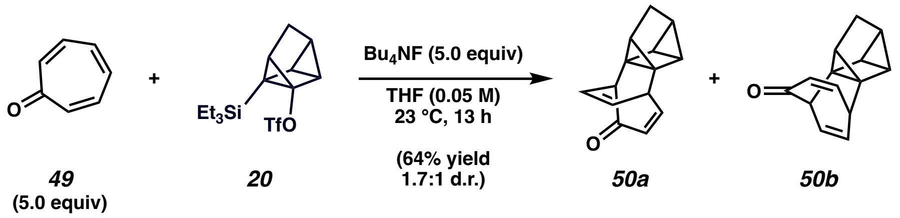

Cycloadduct 50a and **50b.** Followed General Procedure 2. Diastereoselectivity ratio (d.r.) was determined by integration of the ^{1}H NMR spectrum of crude reaction mixture (1.7:1, **50a**:**50b**, average of 2 experiments). Purification by automated column chromatography (0 → 10% hexanes:EtOAc) provided an inseparable mixture of cycloadducts **50a** and **50b** (64% yield, average of two experiments) as a pale-yellow film. Characterization was necessarily performed on the mixture of isomers. **Cycloadduct 50a** and **50b**: R_f 0.63 (4:1 hexanes:EtOAc); ^{1}H NMR (600 MHz, C_6D_6) **50a:** δ 6.54 (dd, *J* = 11.1, 7.2 Hz, 1H), 6.05 – 6.01 (m, 1H), 5.71 (dd, *J* = 8.8, 7.0 Hz, 1H), 5.41 (dd, *J* = 8.7, 7.2 Hz, 1H), 3.70 (dd, *J* = 7.2, 2.1 Hz, 1H), 2.77 (t, *J* = 7.1 Hz, 1H), 1.95 (d, *J* = 1.8 Hz, 1H), 1.91 (dt, *J* = 2.9, 1.5 Hz, 2H), 1.58 (t, *J* = 4.8 Hz, 1H), 1.37 (t, *J* = 4.8 Hz, 1H), 1.31 (tq, *J* = 4.9, 1.5 Hz, 1H); **50b:** δ 6.33 (dd, *J* = 8.1, 6.5 Hz, 1H), 6.06 – 6.03 (m, 2H, overlapping signal), 5.63 (dd, *J* = 11.1, 1.3 Hz, 1H), 3.84 (dd, *J* = 6.7, 1.6 Hz, 1H), 2.93 (dd, *J* = 8.7, 6.4 Hz, 1H), 1.94 – 1.92 (m, 2H), 1.89 (t, *J* = 1.5 Hz, 1H), 1.34 (t, *J* = 4.7 Hz, 1H), 1.27 (td, *J* = 4.8, 1.4 Hz, 1H), 1.23 (t, *J* = 4.7 Hz, 1H); ^{13}C NMR (150 MHz, C_6D_6) **50a:** δ 195.3, 151.0, 136.7, 131.6, 126.4, 54.1, 38.9, 36.8, 33.0, 32.6, 25.2, 24.3, 15.4, 15.3; **50b:** 193.0, 148.7, 138.8, 131.6, 129.1, 53.7, 38.7, 37.2, 34.2, 28.7, 25.0, 23.5, 14.3, 14.1; IR (film): 3051, 2928, 1668, 1623, 1376 cm^{-1}; HRMS-APCI (*m/z*) [M+H]$^+$ calcd for $C_{14}H_{13}O^+$, 197.0961; found 197.0958.

*Note: Cycloadducts **50a** and **50b** were obtained as a mixture of diastereomers. Multiple stereoisomers are present in the NMR spectra. The structures are indicated on the spectrum, and the ratio of each isomer is determined by integration of crude ^{1}H NMR. Cycloadduct **50b** was found to be significantly more volatile than cycloadduct **50a**. Therefore, the ^{1}H NMR spectrum of isolated products indicated a 10:1 ratio of **50a** and **50b**.*

The structure of **50a** was verified by 2D-NOESY, as the following interactions were observed:

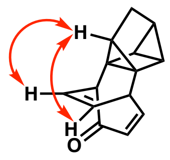

50a

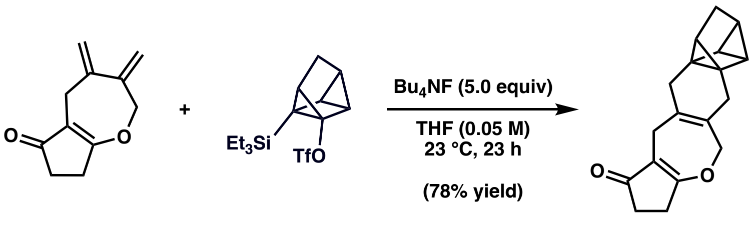

Cycloadduct 52. Followed General Procedure 2. Purification by automated column chromatography (0 → 5% hexanes:acetone) provided cycloadduct **52** (78% yield, average of two experiments) as a colorless oil. **Cycloadduct 52**: R_f 0.33 (4:1 hexanes:acetone); ^{1}H NMR (600 MHz, C_6D_6): δ 4.23 (d, J = 12.1 Hz, 1H), 4.01 (d, J = 12.1 Hz, 1H), 2.91 (s, 2H), 2.51 (d, J = 18.1 Hz, 1H), 2.45 (d, J = 19.3 Hz, 1H), 2.10 – 2.05 (m, 1H), 2.04 – 2.00 (m, 3H), 2.00 – 1.97 (m, 2H), 1.92 –1.89 (m, 2H), 1.40 (t, J = 5.0 Hz, 1H), 1.37 (t, J = 5.1 Hz, 1H), 1.34 – 1.31 (m, 1H), 0.98 – 0.96 (m, 1H); ^{13}C NMR (150 MHz, C_6D_6): δ 202.5, 184.0, 139.4, 125.3, 115.3, 72.3, 32.8, 32.3, 32.2, 29.5, 27.9, 27.3, 27.1, 24.8, 20.2, 20.1, 18.84, 18.82; IR (film): 2926, 2860, 1618, 1402, 1264 cm^{-1}; HRMS-APCI (m/z) [M+H]$^+$ calcd for $C_{18}H_{19}O_2^+$, 267.1380; found 267.1377.

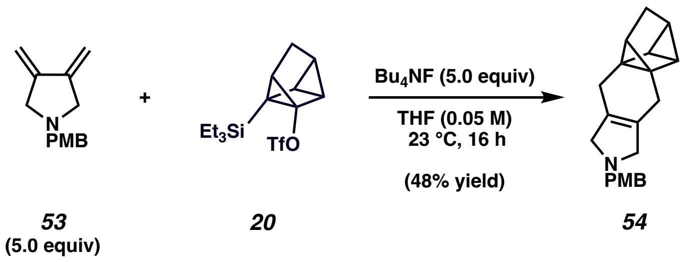

Cycloadduct 54. Following a modified version of General Procedure 2. Reaction mixture was directly concentrated under reduced pressure without aqueous workup to obtain crude material. Purification by preparative TLC (50% hexanes:EtOAc, with 1% NH_4OH (with respect to the amount of solvent added) provided cycloadduct **54** (48% yield, average of two experiments) as a colorless oil. **Cycloadduct 54**: R_f 0.22 (1.5:1 hexanes:EtOAc); ^{1}H NMR (500 MHz, $CDCl_3$): δ 7.27 – 7.23 (overlapped with solvent peak, 2H), 6.87 – 6.83 (m, 2H), 3.80 (s, 3H), 3.71 (br s, 2H), 3.36 – 3.22 (m, 4H), 2.40 (d, J = 15.7 Hz, 2H), 2.11 (d, J = 15.8 Hz, 2H), 2.02 (s, 2H), 1.53 – 1.48 (overlapped with residual water peak, 3H), 1.09 (s, 1H); ^{13}C NMR (150 MHz, $CDCl_3$): δ 158.8, 131.8, 130.0, 129.7, 113.8, 62.2, 60.1, 55.4, 32.4, 32.1, 25.3, 21.8, 21.5, 18.8; IR (film): 3047, 2858, 2841, 1612, 1513 cm^{-1}; HRMS-APCI (*m/z*) $[M+H]^+$ calcd for $C_{21}H_{24}NO^+$, 306.1852; found 306.1851.

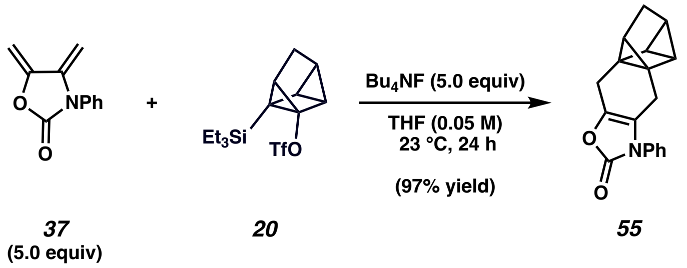

Cycloadduct 55. Followed General Procedure 2. Purification by automated column chromatography (0 → 50% hexanes:EtOAc) provided cycloadduct **55** (97% yield, average of two experiments) as a white amorphous foam. **Cycloadduct 55**: R_f 0.28 (9:1 hexanes:EtOAc); ^{1}H NMR (500 MHz, $CDCl_3$): δ 7.49 – 7.41 (m, 2H), 7.38 – 7.29 (m, 3H), 2.99 (d, J = 16.6 Hz, 1H), 2.88 (d, J = 16.6 Hz, 1H), 2.69 (d, J = 17.0 Hz, 1H), 2.47 (d, J = 16.8 Hz, 1H), 2.08 (dt, J = 14.5, 11.0 Hz, 2H), 1.67 – 1.62 (m, 1H), 1.61 – 1.54 (overlapped with residual water peak, 2H), 1.29 – 1.24 (m, 1H); ^{13}C NMR (125 MHz, $CDCl_3$): δ 155.0, 134.12, 134.06, 129.5, 128.0, 125.9, 119.7, 32.4, 31.9, 25.4, 22.2, 21.1, 20.5, 20.4, 18.5, 18.4; IR (film): 2927, 1761, 1699, 1503, 706 cm^{-1};

HRMS-APCI (*m/z*) $[M+H]^+$ calcd for $C_{18}H_{16}NO_2^+$, 278.1176; found 278.1173.

G. Additional Reactivity of 11 and Elaboration of 1,7-Quadricyclene Adducts

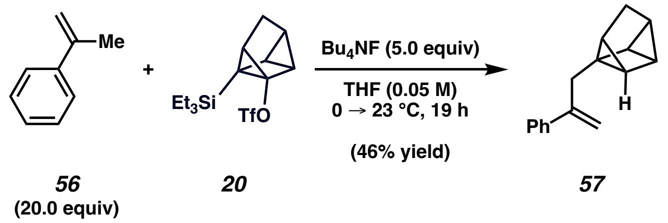

Styrene 57. In a 2-dram vial containing a stirred solution of silyl triflate **20** (35.5 mg, 0.10 mmol, 1.0 equiv) and α-methylstyrene (237 mg, 2.00 mmol, 20.0 equiv) in THF (1.5 mL) at 0 ºC was added Bu_4NF (1.0 M in THF, 0.50 mmol, 5.0 equiv) in one portion. The pierced septa cap was sealed with melted paraffin wax and the reaction mixture was allowed to slowly warm up to 23 °C. After 19 h saturated aq. NH_4Cl (2 mL) was added to quench the reaction. The crude mixture was extracted with pentane (4 mL) and the organic layer was collected. The aqueous layer was extracted with diethyl ether/pentane (1:1, 2 x 4 mL), and the combined organic extracts were dried over Na_2SO_4, filtered and concentrated under reduced pressure to afford a crude mixture. Purification by automated column chromatography (hexanes) yielded adduct **57** (9.5 mg, 0.046 mmol, 46% yield) as a colorless liquid. **Styrene 57**: R_f 0.46 (hexanes); ^{1}H NMR (600 MHz, C_6D_6): δ 7.37 – 7.31 (m, 2H), 7.17 – 7.14 (overlapped with solvent peak , 2H), 7.12 – 7.08 (m, 1H), 5.36 – 5.33 (m, 1H), 5.14 (q, *J* = 1.5 Hz, 1H), 2.83 (d, *J* = 15.9 Hz, 1H), 2.49 (d, *J* = 16.0 Hz, 1H), 1.95 (dt, *J* = 10.7, 1.4 Hz, 1H), 1.92 (d, *J* = 10.9 Hz, 1H), 1.45 (ddd, *J* = 6.1, 4.7, 2.3 Hz, 1H), 1.42 (dd, *J* = 4.7, 1.8 Hz, 1H), 1.40 (dq, *J* = 4.4, 1.9 Hz, 1H), 1.28 (tt, *J* = 4.7, 1.5 Hz, 1H), 1.07 (dq, *J* = 4.7, 1.5 Hz, 1H); ^{13}C NMR (150 MHz, C_6D_6): δ 146.9, 142.3, 128.4, 127.6, 126.6, 113.0, 35.5, 32.4, 28.8, 27.0, 25.1, 19.2, 18.1, 13.9; IR (film): 3059, 2928, 2858, 1628, 1495 cm^{-1}; HRMS-APCI (*m/z*) $[M+H]^+$ calcd for $C_{16}H_{17}^+$, 209.1325; found 209.1323.

Note: Sealing of the reaction vessel with paraffin wax was taken as an extra precaution, but not required for the success of the reaction.

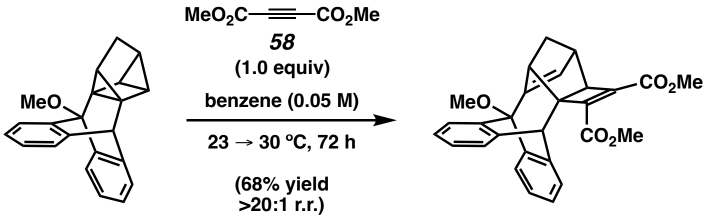

45 ***59***

Norbornene 59. A 1-dram vial containing a stir bar was charged sequentially with cycloadduct **45** (6.5 mg, 22 µmol, 1.0 equiv), dimethyl acetylenedicarboxylate (**58**) (3.1 mg, 22 µmol, 1.0 equiv), and benzene (0.44 mL, 0.05 M). The vial was then sealed with a Teflon-lined cap, and stirred for 48 h at 23 °C. After 48 h, the reaction was warmed to 30 °C and left stirring for an additional 24 h. The mixture was concentrated under reduced pressure. ^{1}H NMR analysis of the reaction mixture indicated >20:1 rr. Purification by preparative TLC (13% hexanes:EtOAc) afforded adduct **59** (6.5 mg, 15 µmol, 68% yield) as a colorless oil. **Norbornene 59**: R_f 0.55 (23% hexanes:EtOAc); ^{1}H NMR (500 MHz, C_6D_6): δ 7.73 (d, *J* = 7.5 Hz, 1H), 7.63 (d, *J* = 7.5 Hz, 1H), 7.40 (d, *J* = 7.4 Hz, 1H), 7.14 – 7.04 (m, 2H), 7.01 – 6.94 (m, 1H), 6.83 – 6.75 (m, 2H), 5.38 – 5.35 (m, 1H), 4.19 (s, 1H), 3.57 (s, 3H), 3.42 (s, 3H), 3.37 (s, 3H), 2.52 (br s, 1H), 2.43 (s, 2H), 1.27 (d, *J* = 10.2 Hz, 1H), 1.11(d, *J* = 10.2 Hz, 1H); ^{13}C NMR (125 MHz, C_6D_6): δ 162.1, 161.9, 153.4, 148.5, 146.9, 144.5, 141.9, 141.7, 141.4, 140.1, 127.8, 127.2, 126.5, 126.3, 126.0, 125.3, 122.1, 118.4, 81.6, 59.2, 55.1, 51.4, 51.3, 51.2, 49.8, 48.3, 42.5, 36.3; IR (film): 3068, 2951, 2855, 1721, 1435 cm^{-1}; HRMS-APCI (*m/z*) $[M+H]^+$ calcd for $C_{28}H_{25}O_5^+$, 441.1697; found 441.1697.

The structure of compound **59** was verified by 2D-NOESY, as the following interactions were observed:

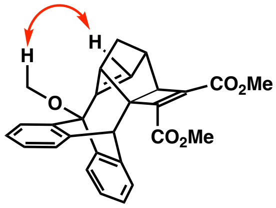

59

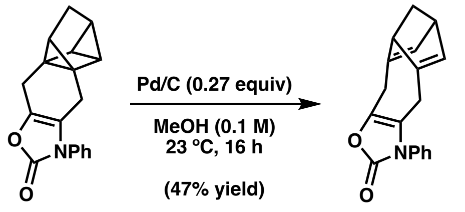

55 **60**

Norbornadiene 60. A 1-dram vial containing a magnetic stir bar was charged with palladium on carbon (7.5 mg, 10% palladium by weight, 7.0 µmol, 0.27 equiv). To this vial was added cycloadduct **55** (7.2 mg, 26 µmol, 1.0 equiv) in MeOH (0.26 mL, 0.1 M) via syringe. The vial was then sealed with a Teflon-lined cap, and stirred at 23 °C. After stirring for 16 h, the mixture was filtered through a plug of Celite, eluting with EtOAc (10 mL). The eluate was concentrated under reduced pressure to afford the crude product. Purification by preparative TLC (16% hexanes:EtOAc) afforded adduct **60** (3.4 mg, 12 µmol, 47% yield) as a colorless oil. **Norbornadiene 60**: R_f 0.31 (20:3 hexanes:EtOAc); ^{1}H NMR (500 MHz, C_6D_6): δ 7.06 – 6.94 (m, 3H), 6.93 – 6.88 (m, 2H), 6.20 – 6.17 (m, 1H), 6.11 – 6.07 (m, 1H), 3.44 – 3.40 (m, 1H), 3.38 – 3.32 (m, 1H), 3.25 – 3.18 (m, 1H), 2.92 – 2.88 (m, 1H), 2.80 – 2.72 (m, 1H), 2.65 – 2.58 (m, 1H), 2.03 – 1.93 (m, 2H); ^{13}C NMR (125 MHz, C_6D_6): 154.1, 153.2, 152.9, 143.0, 142.7, 134.8, 133.6, 129.4, 121.4, 61.6, 57.7, 52.5, 29.5, 28.4; IR (film): 3065, 2960, 1762, 1385, 958 cm^{-1}; HRMS-APCI (*m/z*) $[M+H]^+$ calcd for $C_{18}H_{16}NO_2^+$, 278.1176; found 278.1176.

The structure of norbornadiene **60** was verified by 2D-NOESY, as the following interactions were observed:

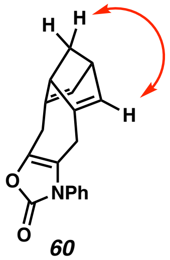

60

Note: Based on the suggestion of a referee, initial scouting experiments were carried out to probe the thermal stability of **55** and the photochemical back-conversion of **60** to **55**. Thermal retro [2+2] conversion of **55** to norbornadiene **60** was carried out in toluene-d_8. The reaction proceeded to completion after heating at 120 °C for 115 h, demonstrating the thermal conversion of **55** to **60**.

UV irradiation of the resulting diene **60**, 10 cm from a UVB lamp (~290–320 nm) for 24 h in the presence of 10 mol% acetophenone in C_6D_6 affected [2+2] cycloaddition to regenerate quadricyclane **55** (~55% yield by ^{1}H NMR analysis). These results suggest the potential of these bridged quadricyclane scaffolds to be used as energy storage materials.

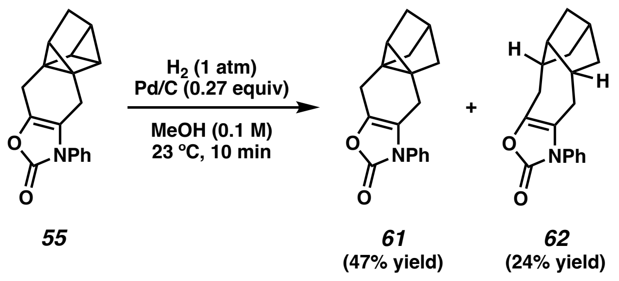

Nortricyclane 61 and norbornane 62. To a 1-dram vial containing cycloadduct **55** (13.0 mg, 47 µmol, 1.0 equiv) under N_2 was added palladium on carbon (13.5 mg, 10% palladium by weight, 12.7 µmol, 0.27 equiv) followed by methanol (0.47 mL) at 23 ºC. Then the reaction mixture was sparged with H_2 (balloon) for 5 min, and stirred under H_2 atmosphere for another 10 min. The reaction mixture was then diluted with EtOAc (1 mL), and filtered through a Celite plug. The filtrate was concentrated under reduced pressure to afford crude material. Purification by preparative TLC (33% hexanes:EtOAc) yielded nortricyclane **61** (6.4 mg, 23 µmol, 47% yield) and bridged norbornane **62** (3.1 mg, 11 µmol, 24% yield) as colorless oils. **Nortricyclane 61**: R_f 0.42 (4:1 hexanes:EtOAc); ^{1}H NMR (600 MHz, C_6D_6): δ 7.16 – 7.14 (overlapped with solvent peak, 2H), 7.11 – 7.04 (t, *J* = 7.5 Hz, 2H), 7.00 – 6.94 (t, *J* = 7.5 Hz, 1H), 2.60 (dt, *J* = 16.5, 3.4 Hz, 1H), 2.47 (dt, *J* = 16.5, 2.9 Hz, 1H), 2.20 (dt, *J* = 16.4, 3.1 Hz, 1H), 2.14 (dt, *J* = 16.4, 2.9 Hz, 1H), 1.75 (m, 1H), 1.25 (d, *J* = 10.3 Hz, 1H), 1.21 (d, *J* = 10.3 Hz, 1H), 1.16 (d, *J* = 10.0 Hz, 1H), 1.07 (d, *J* = 10.0 Hz, 1H), 1.03 (d, *J* = 10.0 Hz, 1H), 0.97 (d, *J* = 9.9 Hz, 1H), 0.59 (s, 1H); ^{13}C NMR (150 MHz, C_6D_6): δ 154.7, 135.1, 133.4, 129.3, 127.2, 125.7, 119.1, 38.7, 38.6, 34.5, 32.3, 22.4, 22.0, 21.7, 21.7, 20.2; IR (film): 2935, 2862, 1765, 1712, 1503 cm^{-1}; HRMS-APCI (*m/z*) $[M+H]^+$ calcd for $C_{18}H_{18}NO_2^+$, 280.1332; found 280.1330. **Norbornane 62**: R_f 0.32 (4:1 hexanes:EtOAc); ^{1}H NMR (600 MHz, C_6D_6): δ 7.10 – 7.04 (m, 4H), 6.98 – 6.95 (m, 1H), 2.45 – 2.42 (m, 2H), 2.08 – 2.03 (m, 1H), 2.03 – 2.01 (m, 1H), 1.99 – 1.96 (m, 1H), 1.74 – 1.68 (m, 1H), 1.67 – 1.63 (m, 2H), 1.52 (tdd, *J* = 11.9, 4.9, 3.0 Hz, 1H), 1.36 (tdd, *J* = 11.7, 4.8, 3.0 Hz, 1H), 1.15 (d, *J* = 9.4 Hz, 1H), 1.12 – 1.08 (m, 1H), 0.65 (ddd, *J* = 12.3, 6.4, 2.3 Hz, 1H), 0.54 (ddd, *J*

= 12.0, 6.2, 2.3 Hz, 1H); ^{13}C NMR (150 MHz, C_6D_6): δ 154.5, 135.1, 134.7, 129.3, 127.7, 127.5, 120.5, 46.9, 43.3, 38.3, 36.2, 35.2, 34.8, 34.4, 27.3, 24.4; IR (film): 2940, 2862, 1761, 1705, 1501 cm^{-1}; HRMS-APCI (*m/z*) [M+H]$^+$ calcd for $C_{18}H_{20}NO_2^+$, 282.1489; found 282.1485.

The structure of **62** was verified by 2D-NOESY, as the following interactions were observed:

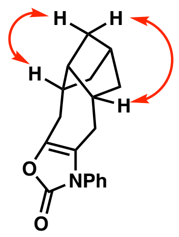

62

H. Synthesis of Heterodimer 14

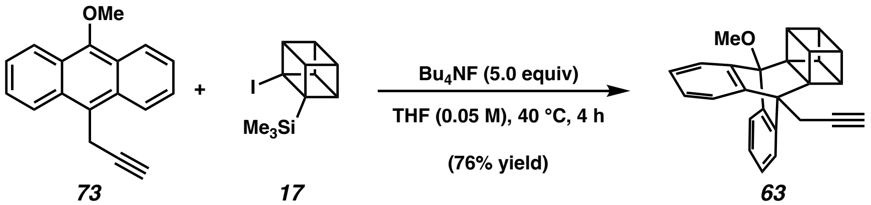

Cycloadduct 63. Following general procedure 1. Purification by automated column chromatography (0 → 40% hexanes:CH_2Cl_2) yielded cycloadduct **63** (26.4 mg, 76% yield) as a colorless oil. **Cycloadduct 63**: R_f 0.30 (9:1 hexanes:EtOAc); ^{1}H NMR (600 MHz, $CDCl_3$) δ 7.58 (dd, *J* = 7.4, 1.5 Hz, 2H), 7.52 (d, *J* = 7.4 Hz, 2H), 7.27 – 7.21 (overlapped with solvent peak, 4H), 3.92 – 3.88 (m, 1H), 3.86 – 3.82 (m, 1H), 3.77 (s, 3H), 3.53 – 3.50 (m, 4H), 3.25 (d, *J* = 2.7 Hz, 2H), 2.12 (t, *J* = 2.7 Hz, 1H); ^{13}C NMR (150 MHz, $CDCl_3$) δ 140.0, 137.8, 126.3, 126.2, 122.8, 121.8, 81.7, 81.6, 72.2, 57.2, 55.6, 54.6, 46.0, 45.4, 44.9, 44.82, 44.75, 17.5; IR (film): 3294, 2956, 1454, 1222, 1099 cm^{-1}; HRMS-APCI (*m/z*) [M+H]$^+$ calcd for $C_{26}H_{21}O^+$, 349.1587; found 349.1571.

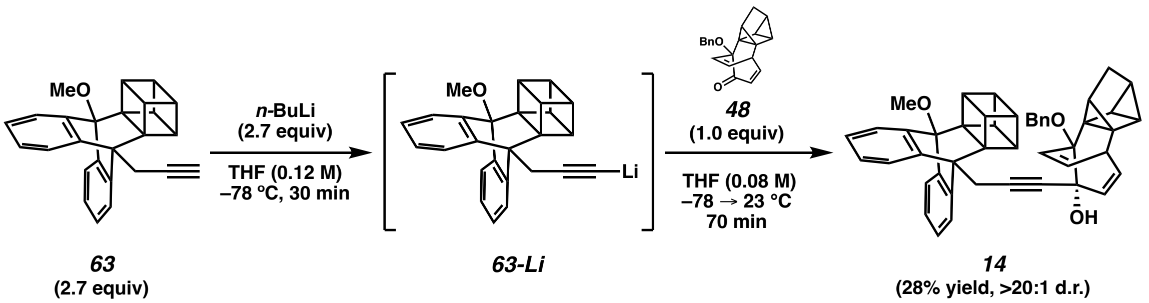

Heterodimer 14. In a dram vial was azeotroped **63** (23.3 mg, 66.9 µmol, 2.7 equiv) from THF (3 x 1 mL) before an oven dried stir bar and THF (150 µL, 0.16 M) were added. The resulting solution was cooled to –78 °C before *n*-BuLi (0.18 M in hexanes, 0.38 mL, 0.68 mmol, 2.7 equiv) was added via syringe dropwise over ~1 min. The solution was stirred for 30 min before a solution of enone **48** (7.6 mg, 25 µmol, 1.0 equiv) in THF (150 µL, 0.16 M) was added via syringe dropwise over ~1 min. After stirring at –78 °C for 10 min, the mixture was brought to 23 °C for 1 h. The reaction was then quenched by the sequential addition of deionized H_2O (1 mL) and brine (1 mL). The aqueous layer was extracted with EtOAc (3 x 1 mL) and the combined organic phase was filtered through a 0.5 x 1 cm Na_2SO_4 plug. The eluate was concentrated under reduced pressure. The crude product was purified by preparative TLC (10% hexanes:EtOAc) to afford heterodimer **14** (>20:1 d.r., 4.5 mg, 6.9 µmol, 28% yield) as a colorless oil. **Heterodimer 14**: R_f 0.26 (9:1 hexanes:EtOAc); ^{1}H NMR (500 MHz, C_6D_6) δ 7.81 (ddd, *J* = 12.6, 7.4, 1.3 Hz, 2H), 7.57 (d, *J* = 7.6 Hz, 1H), 7.47 (d, *J* = 7.5 Hz, 1H), 7.23 – 7.20 (m, 2H), 7.18 – 7.10 (overlapped with solvent, 4H), 7.07 (tdd, *J* = 7.5, 2.1, 1.4 Hz, 2H), 6.03 (d, *J* = 10.9 Hz, 1H), 5.87 (dd, *J* = 11.0, 7.1 Hz, 1H), 5.81 – 5.74 (m, 2H), 4.40 (d, *J* = 11.9 Hz, 1H), 4.34 (d, *J* = 11.9 Hz, 1H), 3.69 (br s, 1H), 3.64 – 3.59 (m, 6H), 3.51 – 3.48 (m, 2H), 3.47 (s, 1H), 3.18 (d, *J* = 16.8 Hz, 1H), 3.13 (d, *J* = 16.8 Hz, 1H), 2.59 (t, *J* = 6.4 Hz, 1H), 2.41 (t, *J* = 5.0 Hz, 1H), 1.94 (dt, *J* = 11.1, 1.5 Hz, 1H), 1.84 (dt, *J* = 11.2, 1.5 Hz, 1H), 1.69 (br s, 1H), 1.51 (t, *J* = 5.0 Hz, 1H), 1.40 – 1.37 (m, 1H); ^{13}C NMR (125 MHz, C_6D_6) δ 140.7, 139.8, 138.82, 138.80, 135.0, 133.5, 132.7, 130.3, 128.60, 128.56, 127.5, 127.4, 126.36, 126.35, 126.33, 126.31, 123.47, 123.5, 122.3, 122.2, 85.0, 82.9, 82.1, 81.4, 74.5, 66.6, 57.4, 55.3, 55.0, 46.4, 45.7, 45.4, 45.08, 45.05, 45.01, 34.6, 32.7, 31.6, 29.0, 25.2, 22.0, 18.3, 16.3, 14.3; IR (film): 3401, 2975, 1454, 1222, 1097, 1115 cm^{-1}; HRMS-APCI (*m/z*) [M+H]$^+$ calcd for $C_{47}H_{39}O_3^+$, 651.2894; found 651.2924.

The newly formed stereocenter present in heterodimer **14** was established by 2D-NOESY, as the following interactions were observed:

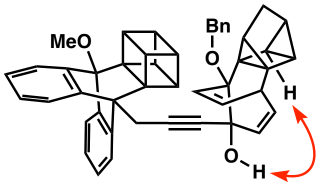

14

Crystal structure analysis of heterodimer 14.

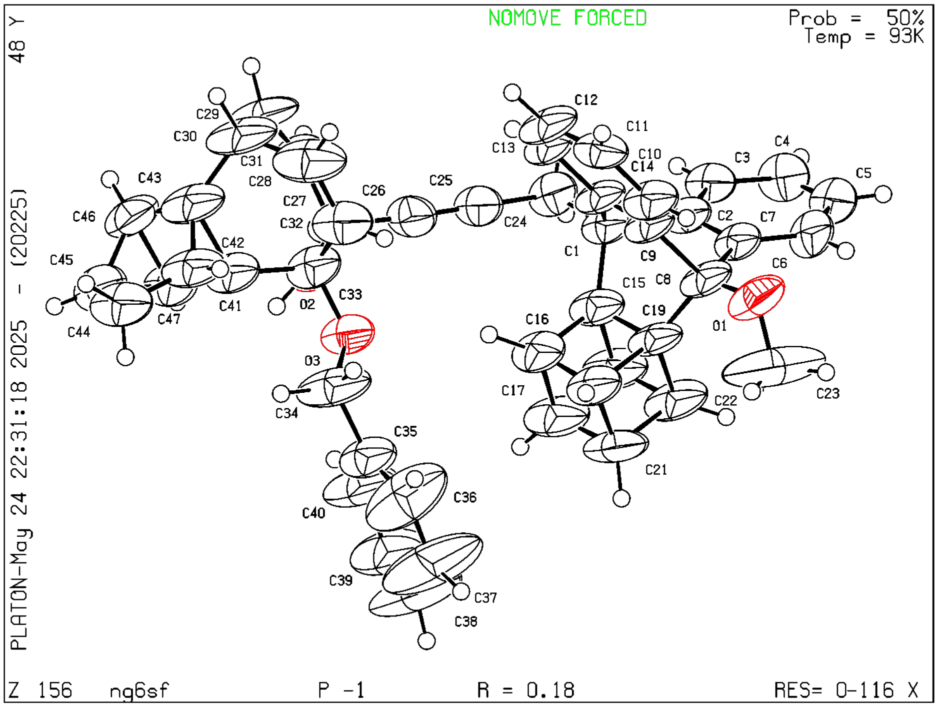

Fig. 7. ORTEP representation of CryoEM MicroED crystallographic structure of **14** (CCDC Registry # 2456006) with the ellipsoids shown at 50% probability level.

Crystals of heterodimer **14** (ng6sf) were grown by evaporation of diethyl ether. Colorless nanocrystals were mounted on a TEM Grid. Data for heterodimer **14** were collected at 93 K on a Thermo Scientific Talos F200C with an FEG electron source and a Thermo Scientific Ceta 16M detector. The diffractometer used 200 keV electrons as a radiation source (λ = 0.0251 Å). All data were integrated and merged with XDS.[18] The structure was solved by direct methods with SHELXT 2018/2 and refined by full-matrix least-squares methods against F^2 using neutral atom scattering factors in SHELXL-2019/2.[14,15,19] All non-hydrogen atoms were refined with

anisotropic displacement parameters. All hydrogen atoms were refined with isotropic displacement parameters. This report and the CIF were generated using FinalCif.[16]

Refinement Details:

All non-hydrogen atoms were refined as anisotropic ellipsoids with a global rigid body restraint. The carbon atoms of a poorly-shaped phenyl ring were restrained to fit a plane. All hydrogen atoms except for the OH were refined using a riding model, and the OH hydrogen atom was included with a displacement parameter linked to the oxygen atom, and the OH bond length was fixed at 0.9 Angstroms.

Table 2. Selected crystallographic parameters for heterodimer **14**.

CCDC number	2456006
Empirical formula	$C_{47}H_{38}O_3$
Formula weight	650.77
Temperature [K]	93
Crystal system	triclinic
Space group (number)	*P* (2)
a [Å]	8.730(2)
b [Å]	13.510(7)
c [Å]	14.820(7)
α [°]	99.30(3)
β [°]	106.86(3)
γ [°]	103.44(3)
Volume [Å^3]	1576.9(12)
Z	2
ρ_{calc} [gcm^{-3}]	1.371
F(000)	288
Crystal colour	colorless
Crystal shape	nanocrystals
Radiation	200 keV electrons (λ=0.0251 Å)
2θ range [°]	0.10 to 1.80 (0.80 Å)
Index ranges	$-10 \leq h \leq 10$
	$-16 \leq k \leq 16$
	$-18 \leq l \leq 18$
Reflections collected	33561
Independent reflections	5427
	R_{int} = 0.1865
	R_{sigma} = 0.1437
Completeness to θ = 0.863°	84.9 %
Data / Restraints / Parameters	5427 / 499 / 456
Goodness-of-fit on F^2	1.538
Final R indexes [$I \geq 2\sigma(I)$]	R_1 = 0.1774<br>wR_2 = 0.4520
Final R indexes [all data]	R_1 = 0.2519<br>wR_2 = 0.4955
Largest peak/hole [eÅ^{-3}]	0.20/−0.21
Extinction coefficient	6806(9)

From the CIF/PLATON check, the following alerts for compound **14** were reported.

Alert level A

```
PLAT029_ALERT_3_A _diffrn_measured_fraction_theta_full value Low
. 0.849 Why?
```

Author Response: Preferred orientation of nanocrystals on the TEM grid caused a cone of uncollectable data.

```
PLAT084_ALERT_3_A High wR2 Value (i.e. > 0.25....... 0.50 Report
```

Author Response: Inadequate merging of data and likely full molecule disorder roughly along the b axis caused a high wR2.

```
PLAT417_ALERT_2_A Short Inter D-H..H-D H2 ..H2 . 1.74 Ang.
-x,1-y,1-z = 2_566 Check
```

Author Response: A hydrogen bond between two adjacent molecules can be seen in the grown structure. The offending hydrogen atom may be better refined as disordered over two positions.

Alert level B

```
RINTA01_ALERT_3_B The value of Rint is greater than 0.18
Rint given 0.186
```

Author Response: Merging of data from 5 nanocrystals caused a high Rint.

```
PLAT020_ALERT_3_B The Value of Rint is Greater Than 0.12
......... 0.186 Report
```

Author Response: Merging of data from 5 nanocrystals caused a high Rint.

```
PLAT082_ALERT_2_B High R1 Value
.................................. 0.18 Report
```

Author Response: Inadequate merging of data and likely full molecule disorder roughly along the b axis caused a high R1.

```
PLAT340_ALERT_3_B Low Bond Precision on C-C Bonds
............... 0.01518 Ang.
```

Author Response: Poor data quality and likely full molecule disorder caused low precision C-C bonds.

```
PLAT411_ALERT_2_B Short Inter H...H Contact H37 ..H37 . 1.98
Ang.
```

```
-x,1-y,-z = 2_565 Check
```

Author Response: Disorder in the phenyl ring that was not refined causes this close contact.

```
PLAT414_ALERT_2_B Short Intra D-H..H-X H2 ..H47 . 1.85 Ang.
x,y,z = 1_555 Check
```

Author Response: The position of H2, the O-H proton, is not clear based on the data, This close contact may or may not be a feature of the compound.

```
PLAT911_ALERT_3_B Missing FCF Refl Between Thmin & STh/L= 0.600
861 Report
-1 6 0, -1 7 0, 0 7 0, -2 8 0, -2 9 0, 2 -8 1,
0 -6 1, 0 -5 1, 1 -5 1, 2 -5 1, 0 -4 1, 0 -3 1,
1 -3 1, 0 -2 1, -7 -1 1, 0 -1 1, -1 7 1, -2 8 1,
0 9 1, -2 10 1, 0-12 2, 0-11 2, 0-10 2, 0 -9 2,
0 -8 2, 1 -8 2, 0 -7 2, 1 -7 2, 0 -6 2, 1 -6 2,
( 831 More Missing: see the .ckf listing file)
```

Author Response: Preferred orientation of nanocrystals on the TEM grid caused a cone of uncollectable data.

NMR Spectra

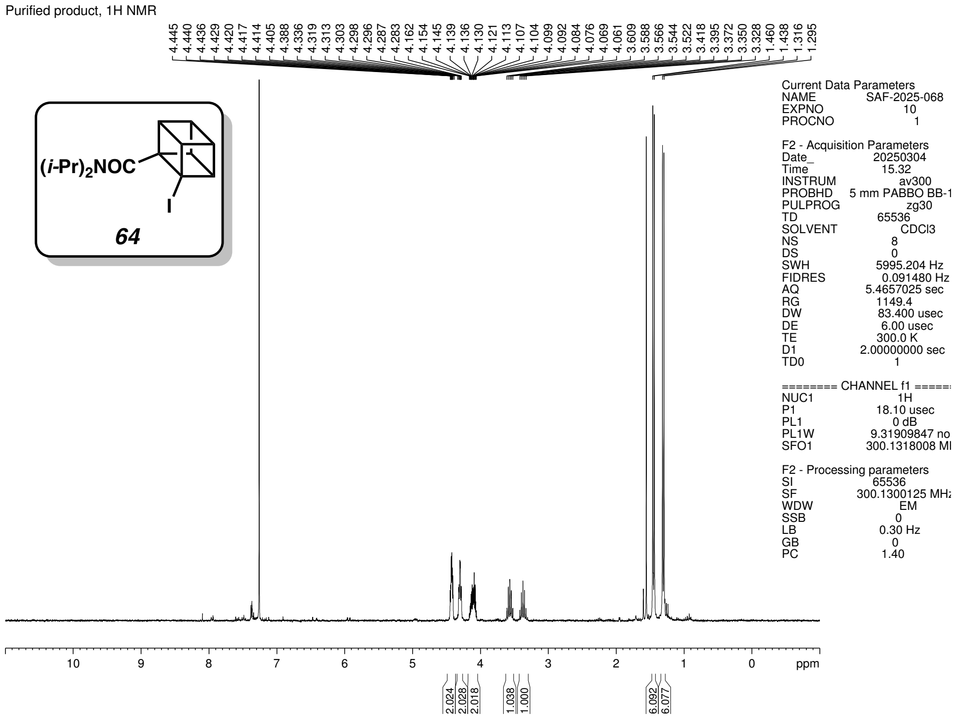

Fig. 8. ^{1}H NMR spectrum of compound **64** in $CDCl_3$ (300 MHz).

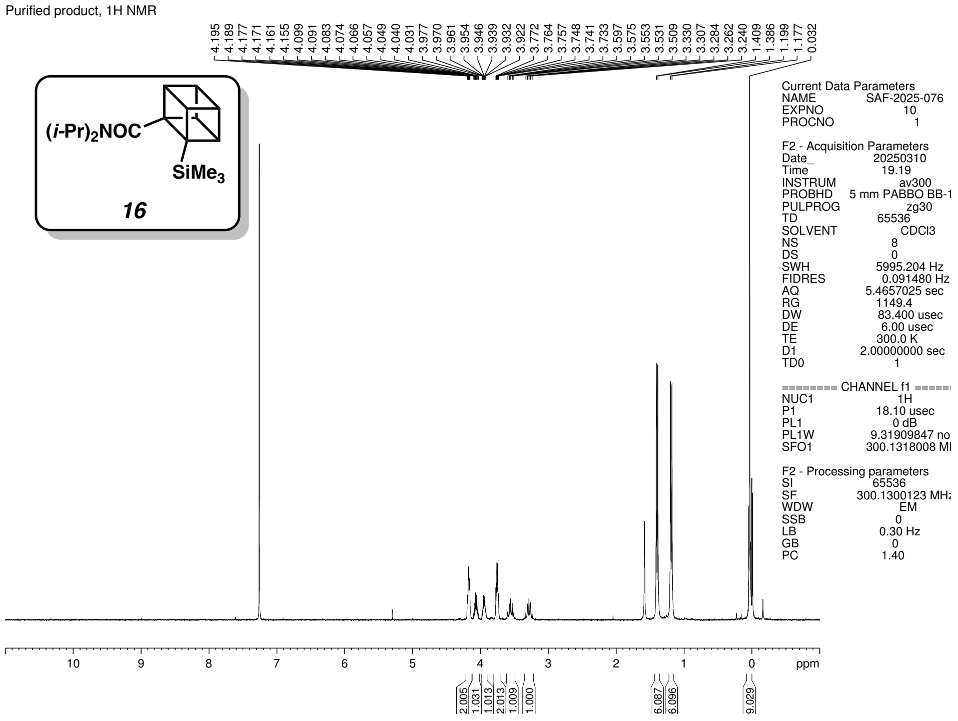

Fig. 9. ^{1}H NMR spectrum of compound **16** in $CDCl_3$ (300 MHz).

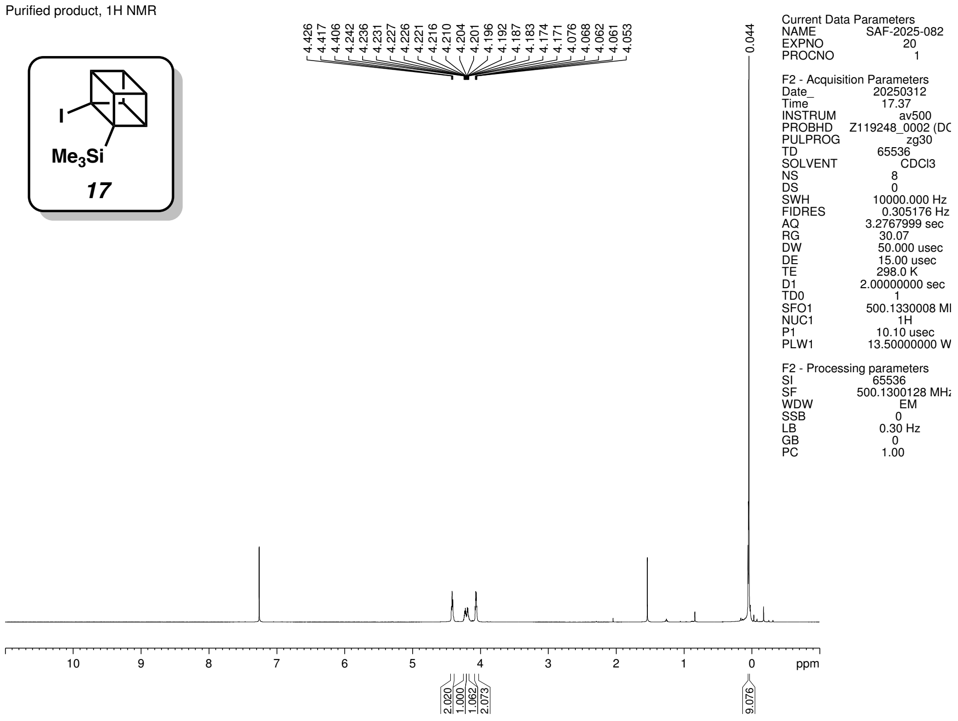

Fig. 10. ^{1}H NMR spectrum of compound **17** in $CDCl_3$ (500 MHz).

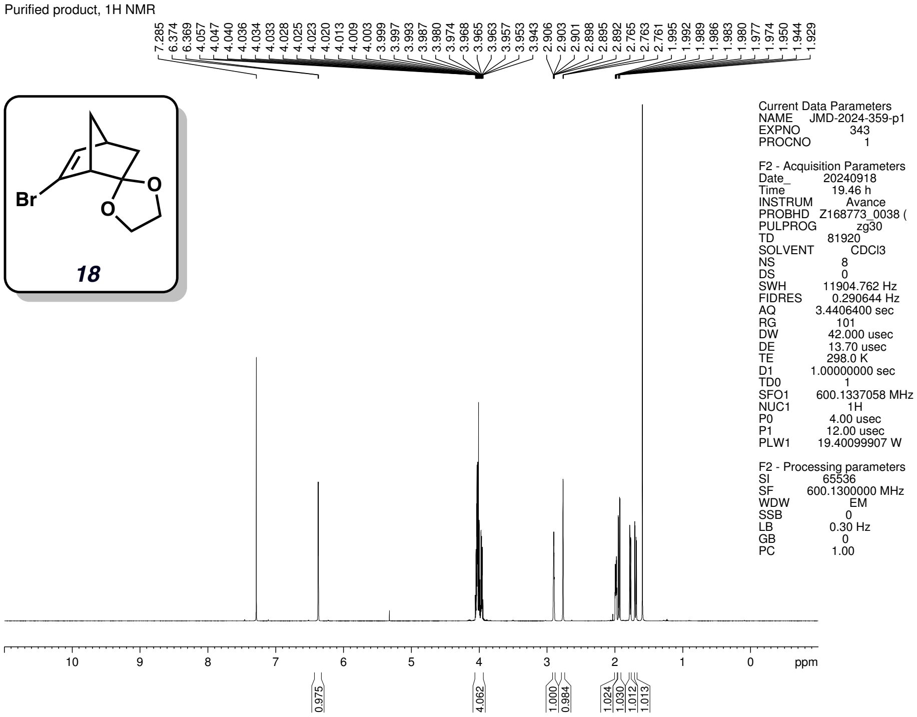

Fig. 11. ^{1}H NMR spectrum of compound **18** in $CDCl_3$ (600 MHz).

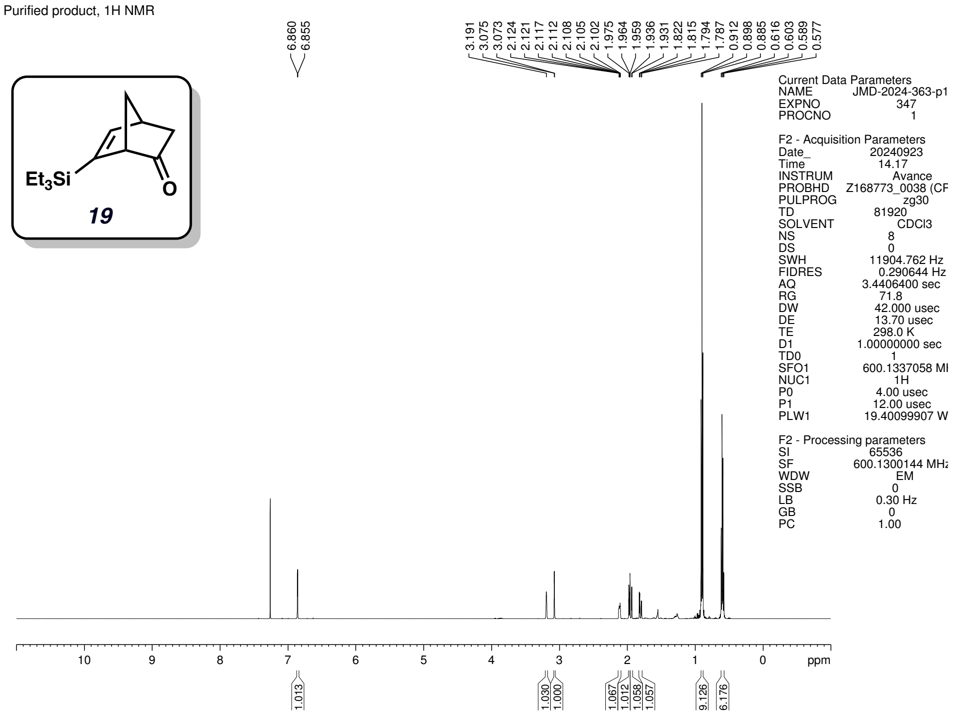

Fig. 12. ^{1}H NMR spectrum of compound **19** in $CDCl_3$ (600 MHz).

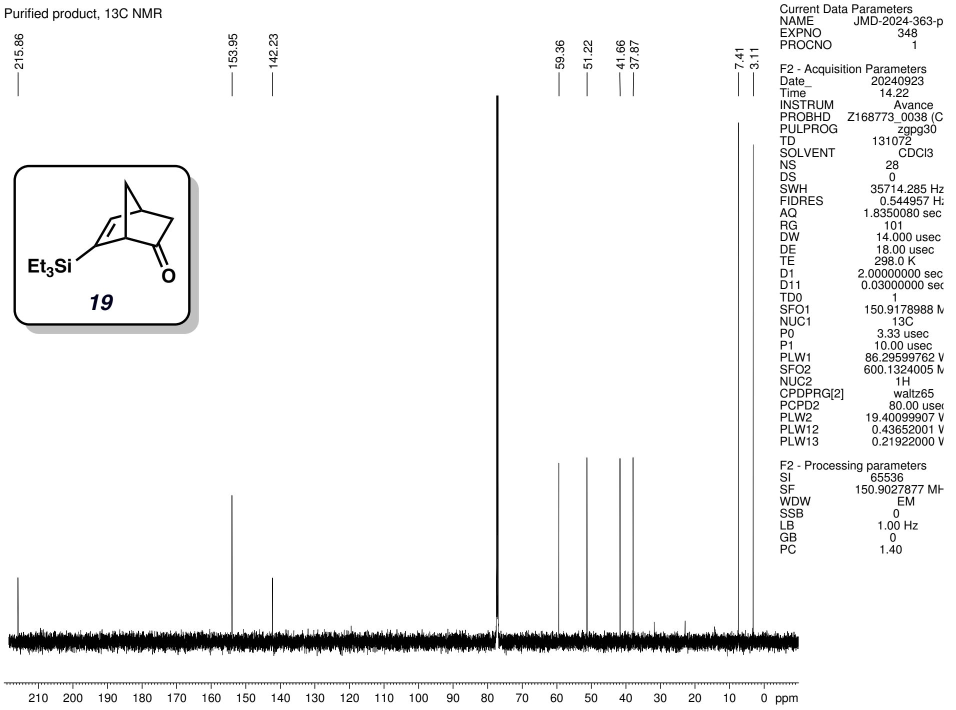

Fig. 13. ^{13}C NMR spectrum of compound **19** in $CDCl_3$ (150 MHz).

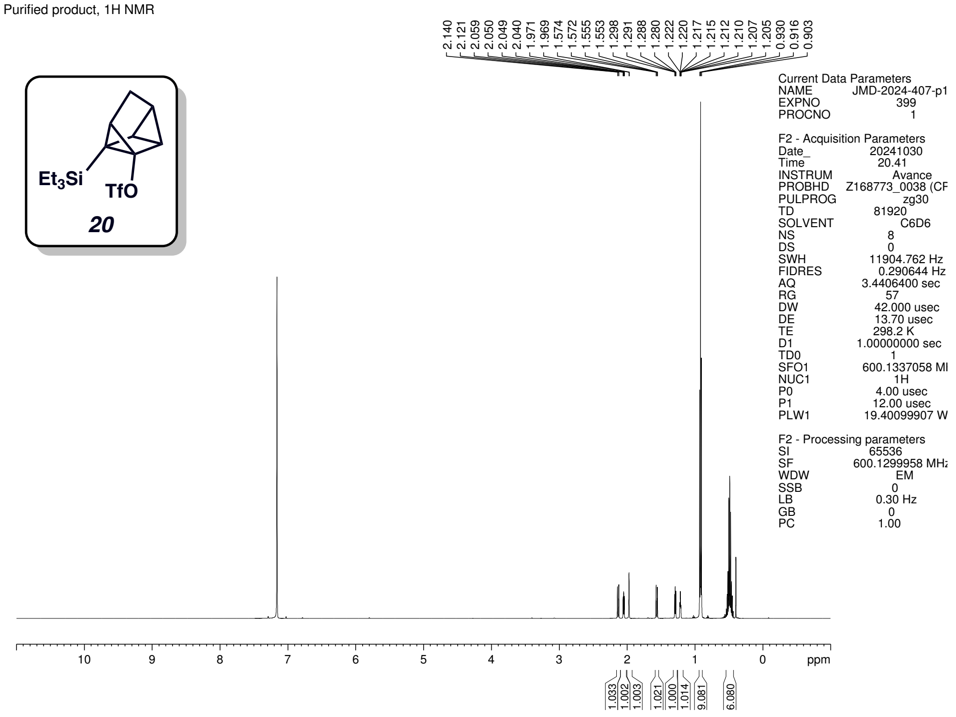

Fig. 14. ^{1}H NMR spectrum of compound **20** in C_6D_6 (600 MHz).

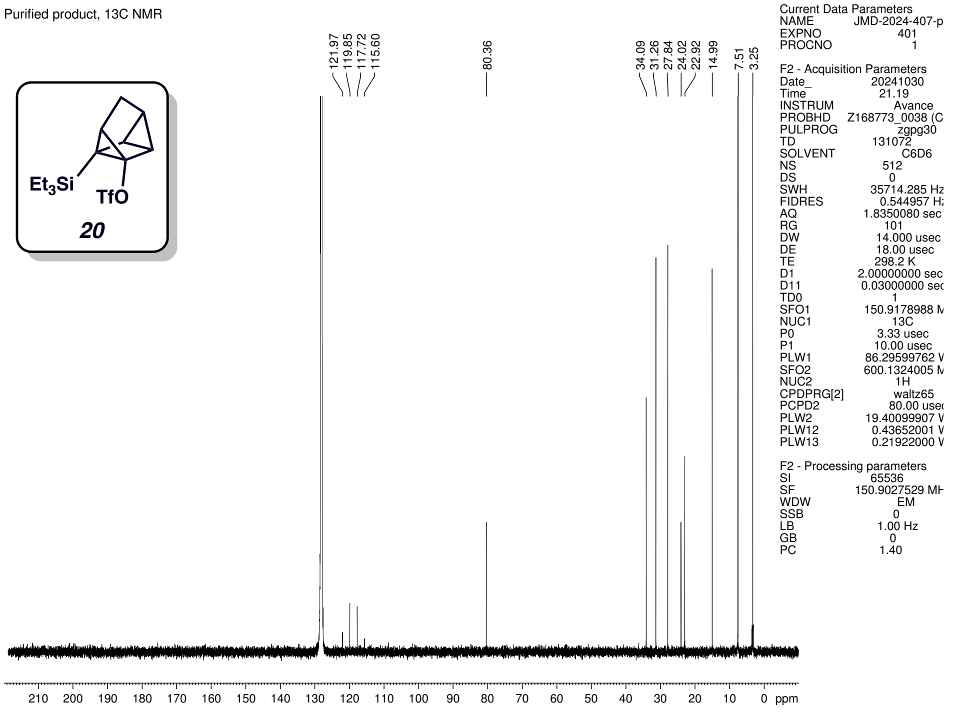

Fig. 15. ^{13}C NMR spectrum of compound **20** in C_6D_6 (150 MHz).

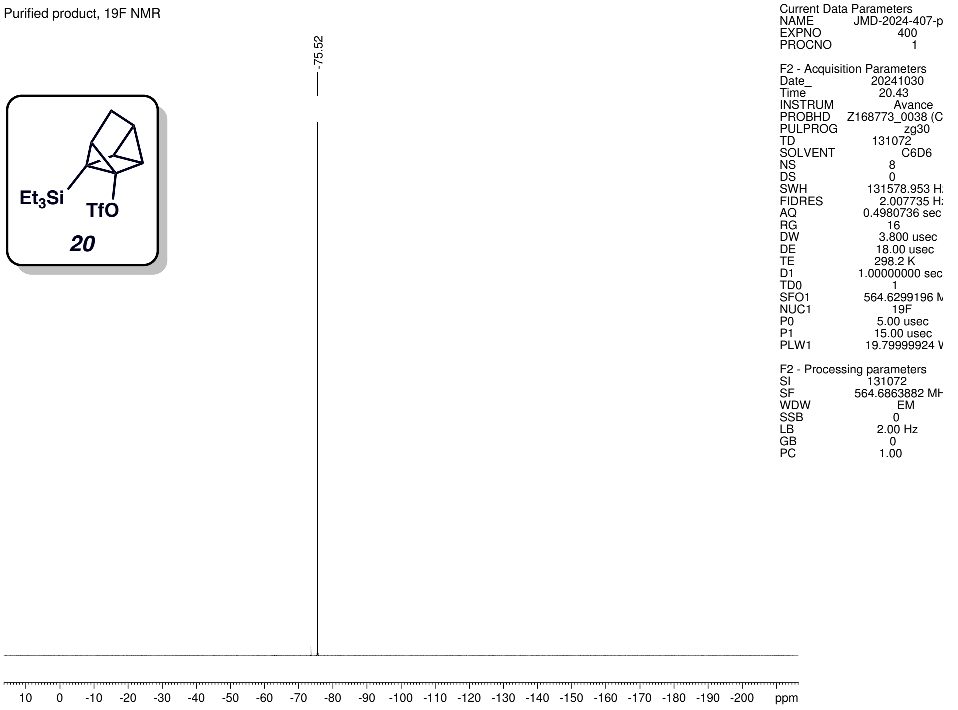

Fig. 16. ^{19}F NMR spectrum of compound **20** in C_6D_6 (564 MHz).

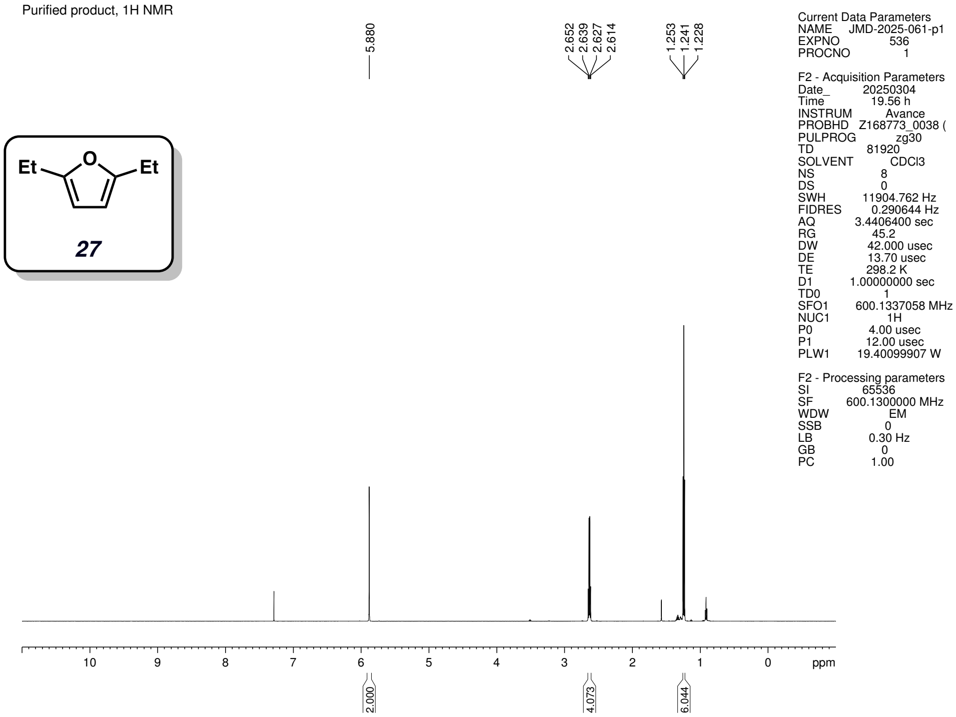

Fig. 17. ^{1}H NMR spectrum of compound **27** in $CDCl_3$ (600 MHz).

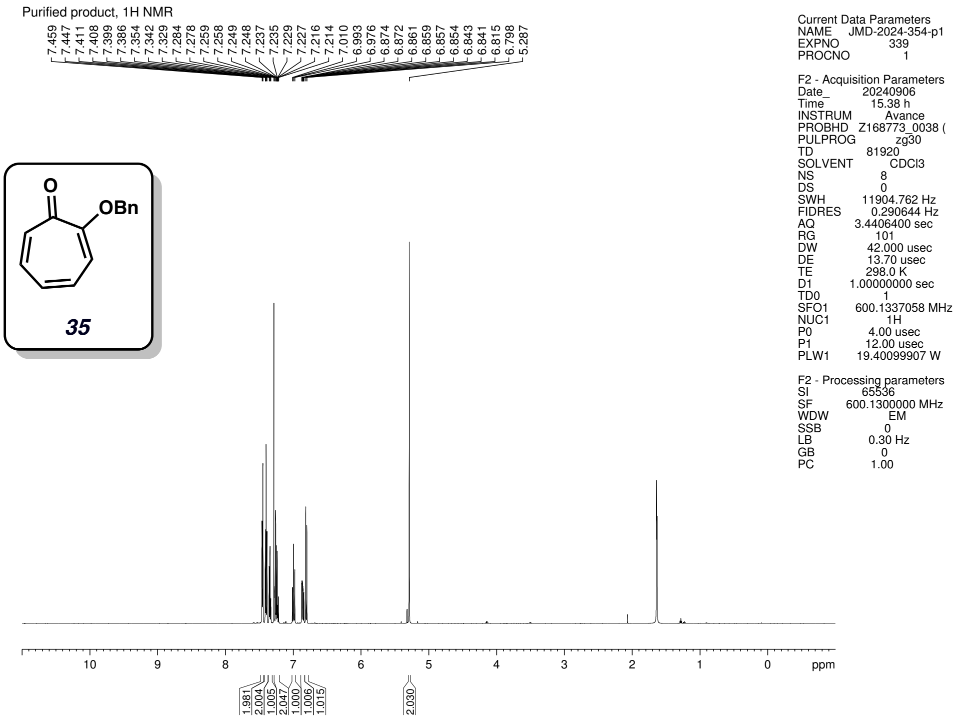

Fig. 18. ^{1}H NMR spectrum of compound **35** in $CDCl_3$ (600 MHz).

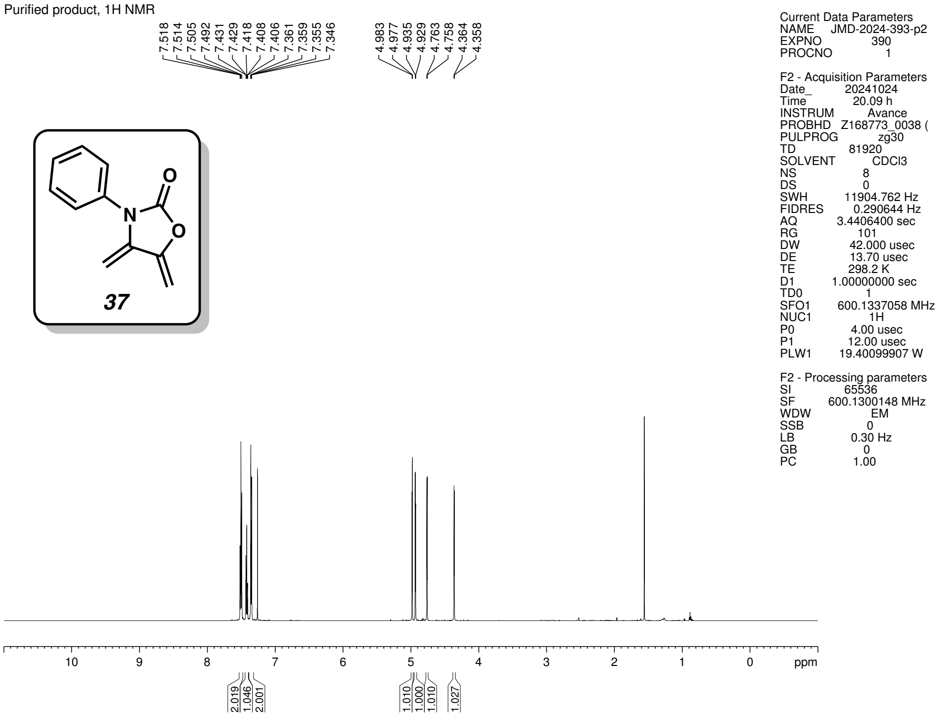

Fig. 19. ^{1}H NMR spectrum of compound **37** in $CDCl_3$ (600 MHz).

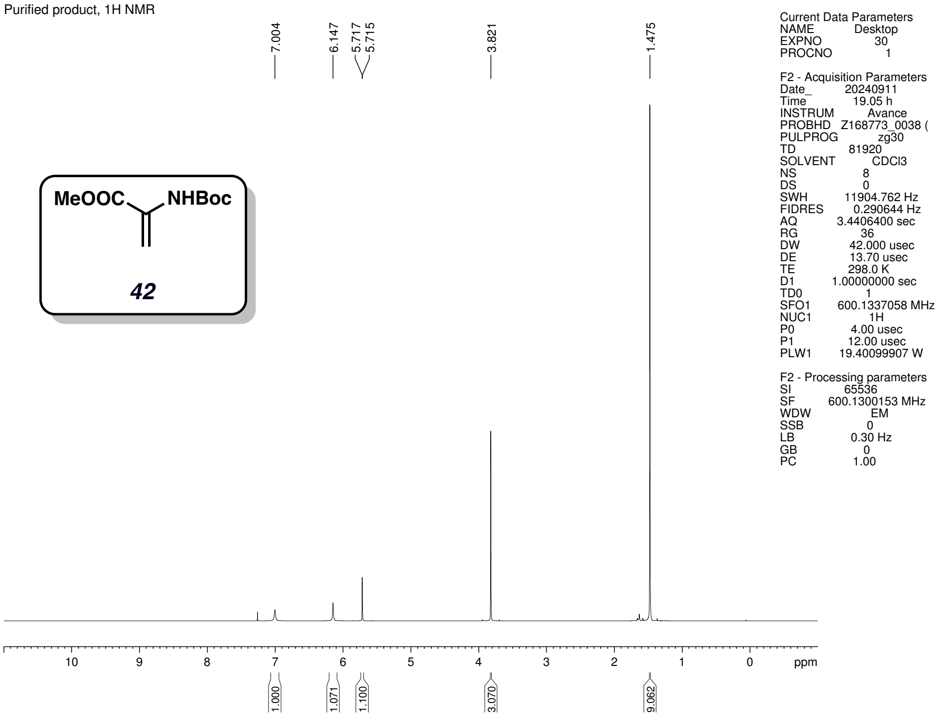

Fig. 20. ^{1}H NMR spectrum of compound **42** in $CDCl_3$ (600 MHz).

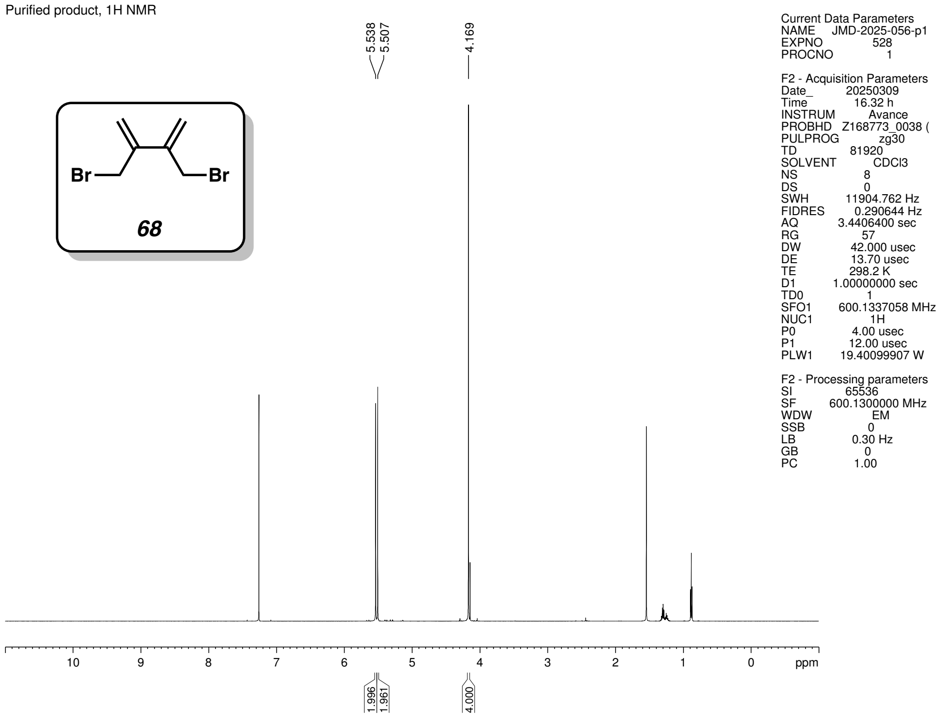

Fig. 21. ^{1}H NMR spectrum of compound **68** in $CDCl_3$ (600 MHz).

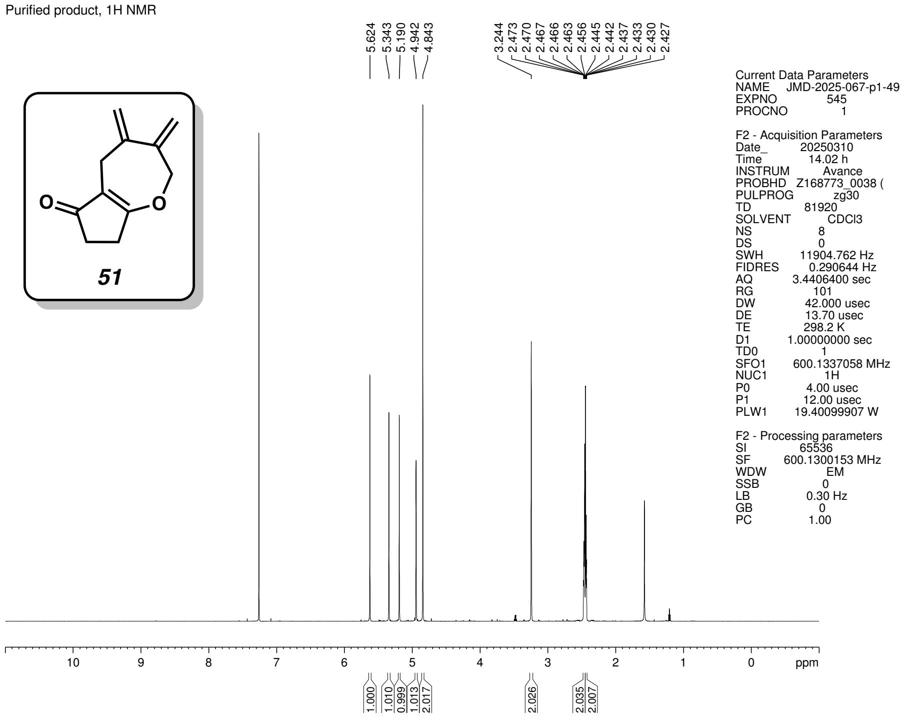

Fig. 22. ^{1}H NMR spectrum of compound **51** in $CDCl_3$ (600 MHz).

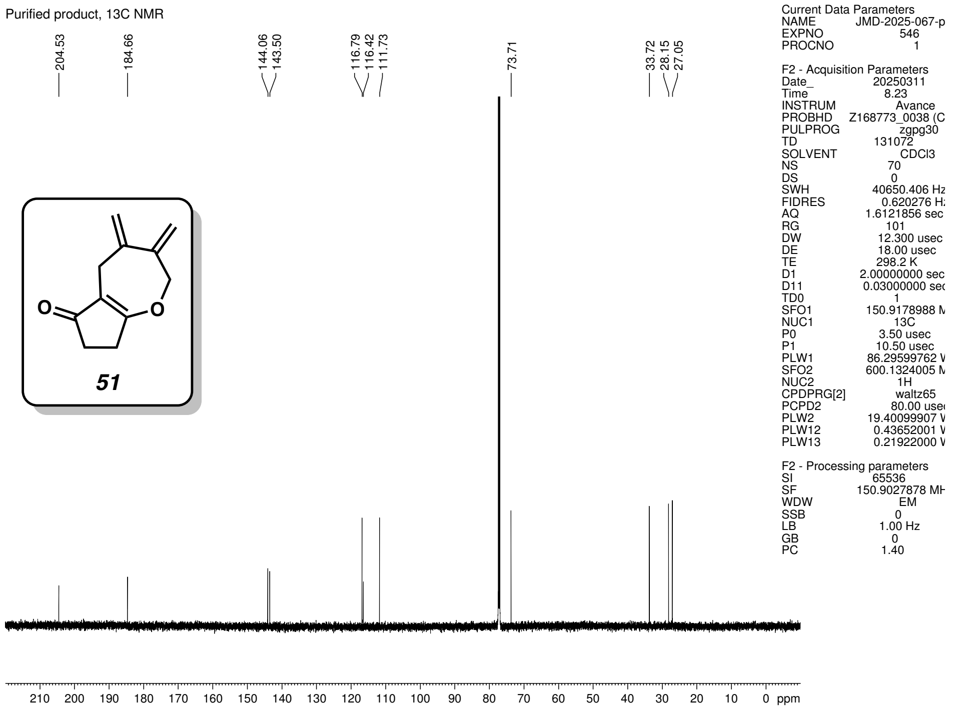

Fig. 23. ^{13}C NMR spectrum of compound **51** in $CDCl_3$ (150 MHz).

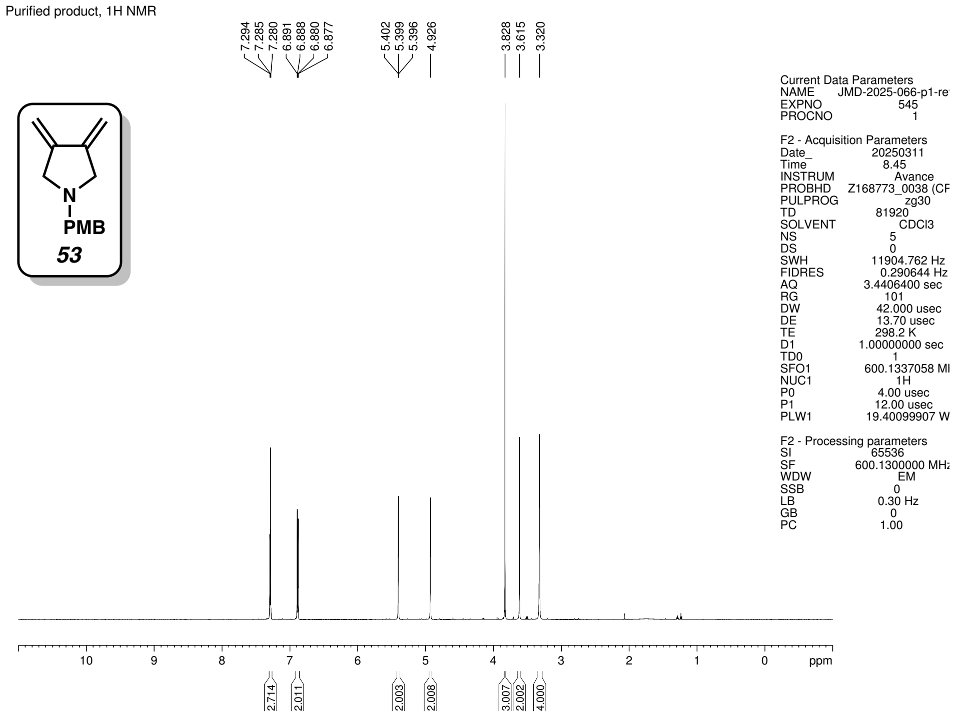

Fig. 24. ^{1}H NMR spectrum of compound **53** in $CDCl_3$ (600 MHz).

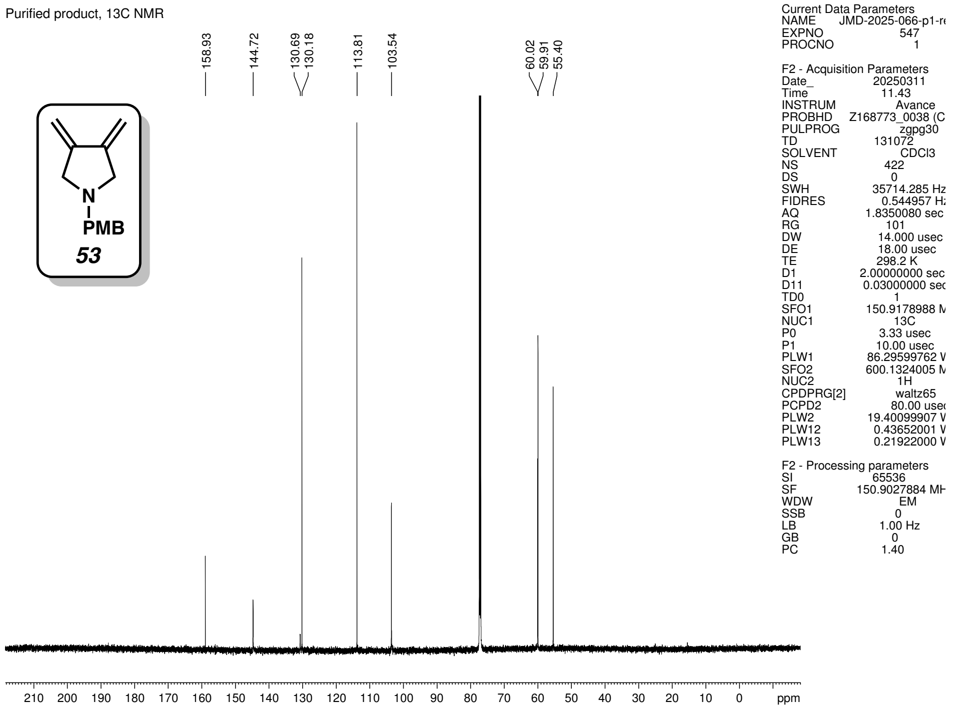

Fig. 25. ^{13}C NMR spectrum of compound **53** in $CDCl_3$ (150 MHz).

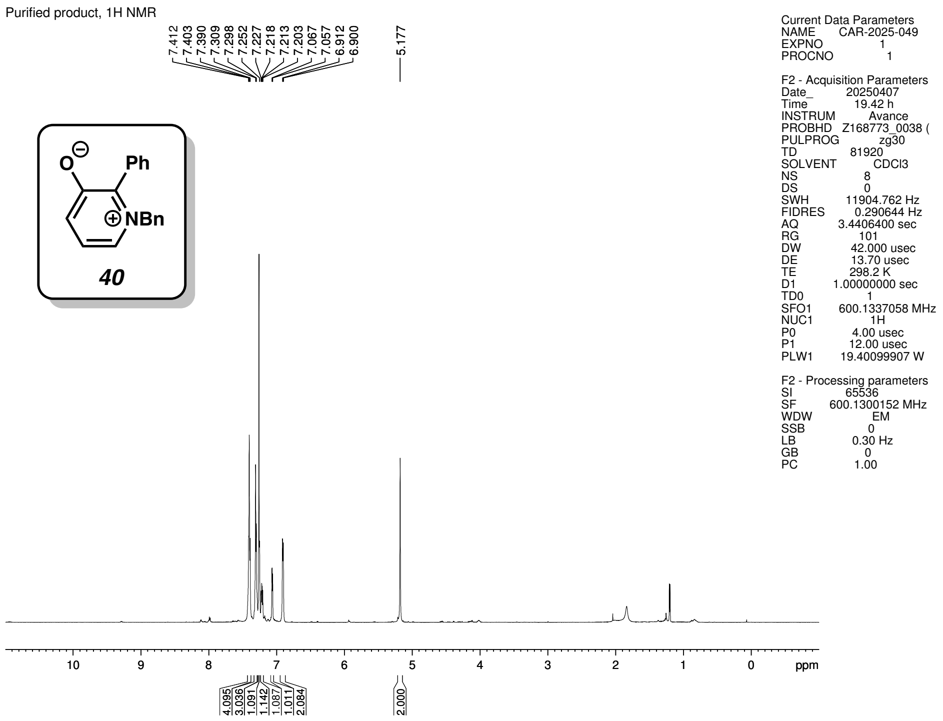

Fig. 26. ^{1}H NMR spectrum of compound **40** in $CDCl_3$ (600 MHz).

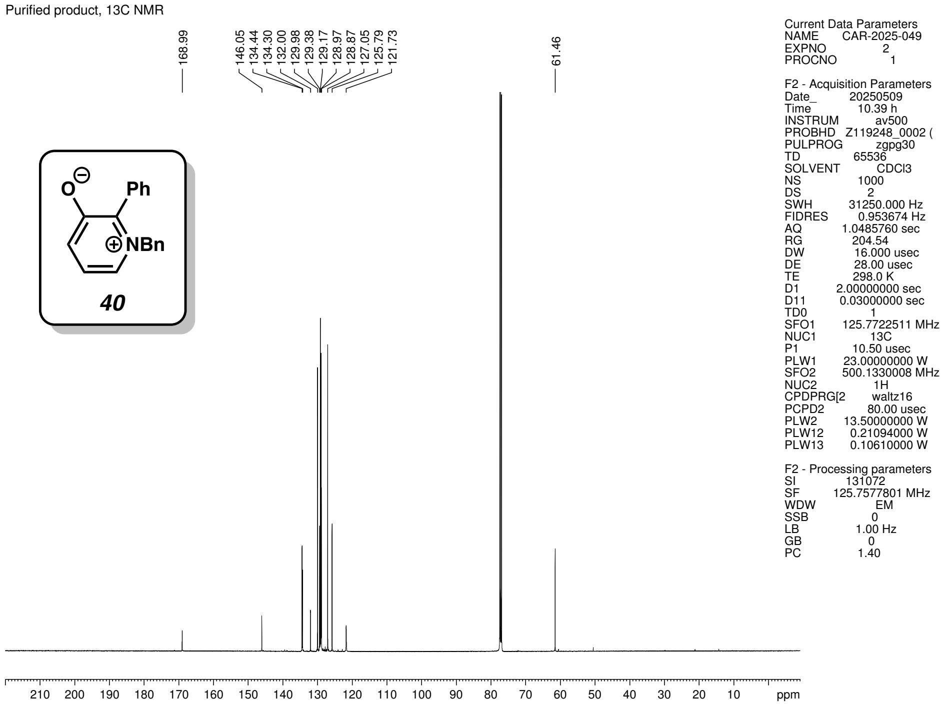

Fig. 27. ^{13}C NMR spectrum of compound **40** in $CDCl_3$ (125 MHz).

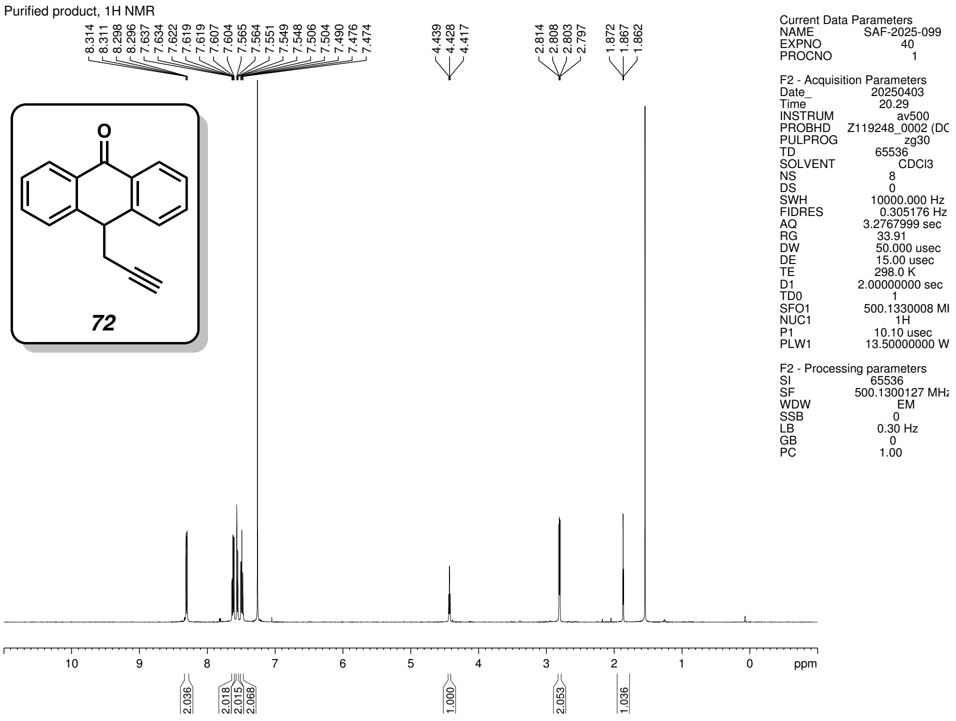

Fig. 28. ^{1}H NMR spectrum of compound **72** in $CDCl_3$ (500 MHz).

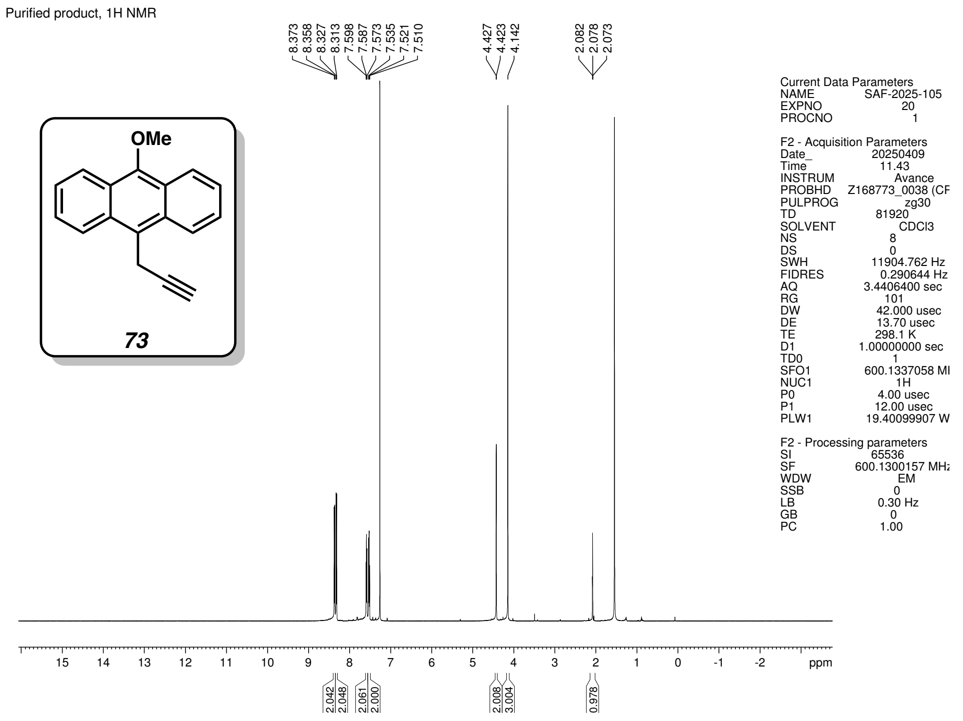

Fig. 29. ^{1}H NMR spectrum of compound **73** in $CDCl_3$ (600 MHz).

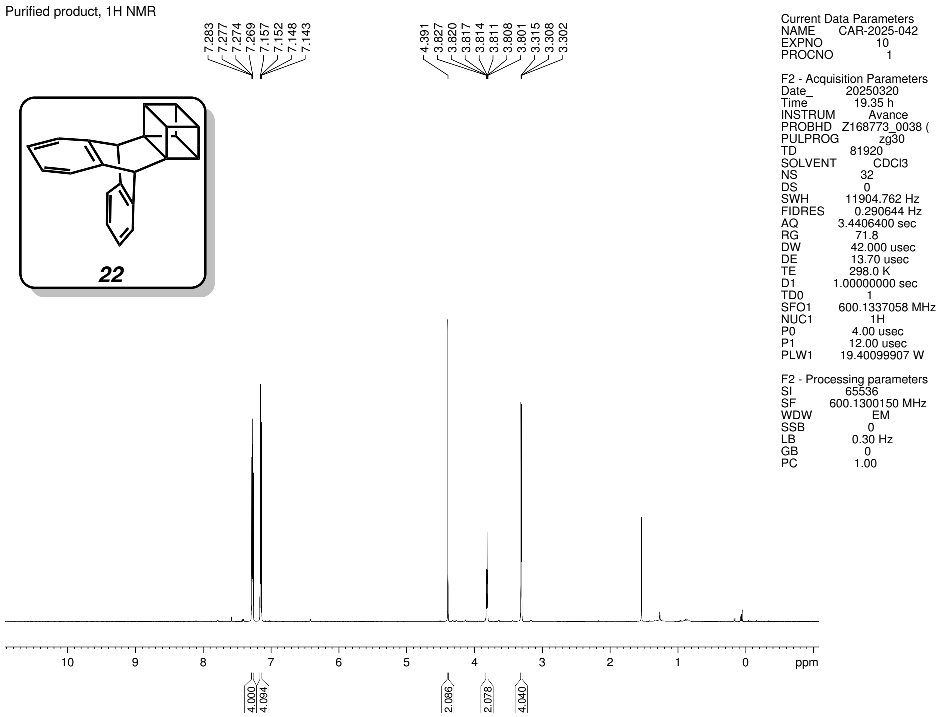

Fig. 30. ^{1}H NMR spectrum of compound **22** in $CDCl_3$ (600 MHz).

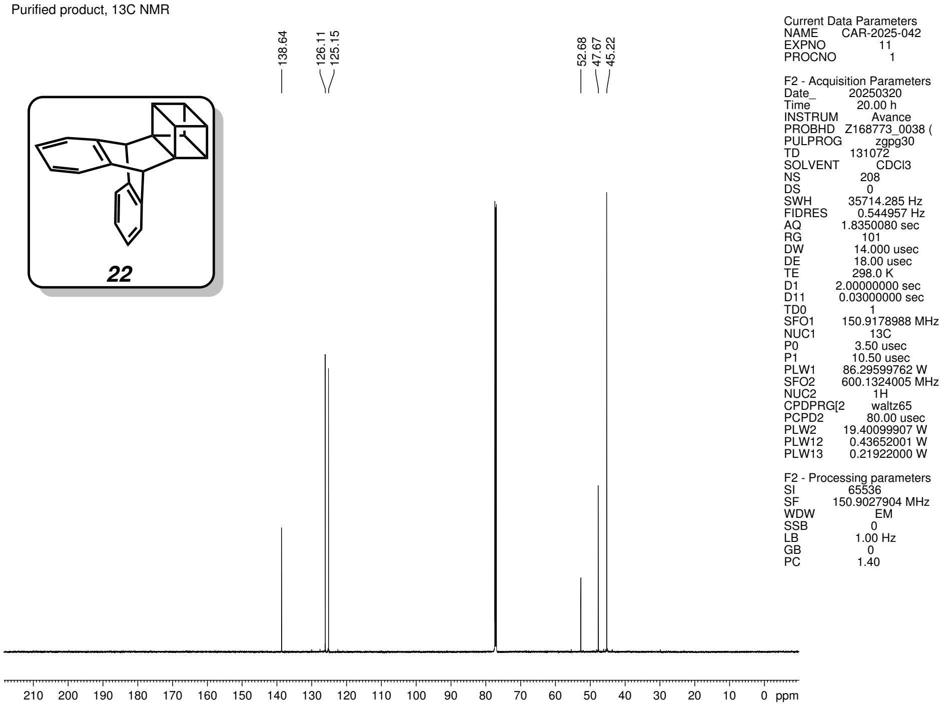

Fig. 31. ^{13}C NMR spectrum of compound **22** in $CDCl_3$ (150 MHz).

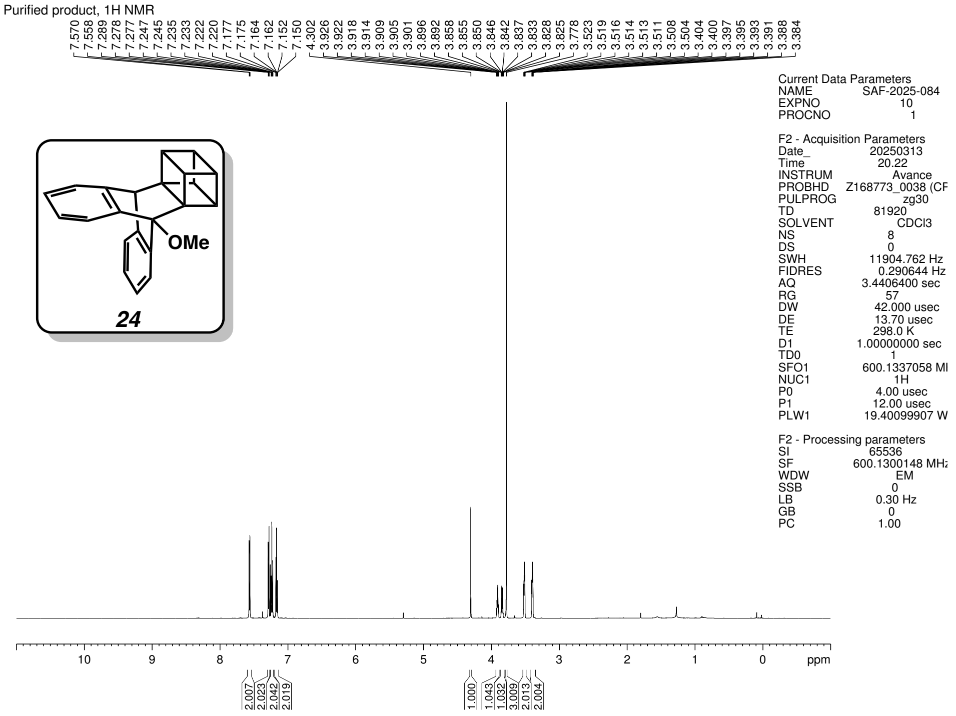

Fig. 32. ^{1}H NMR spectrum of compound **24** in $CDCl_3$ (600 MHz).

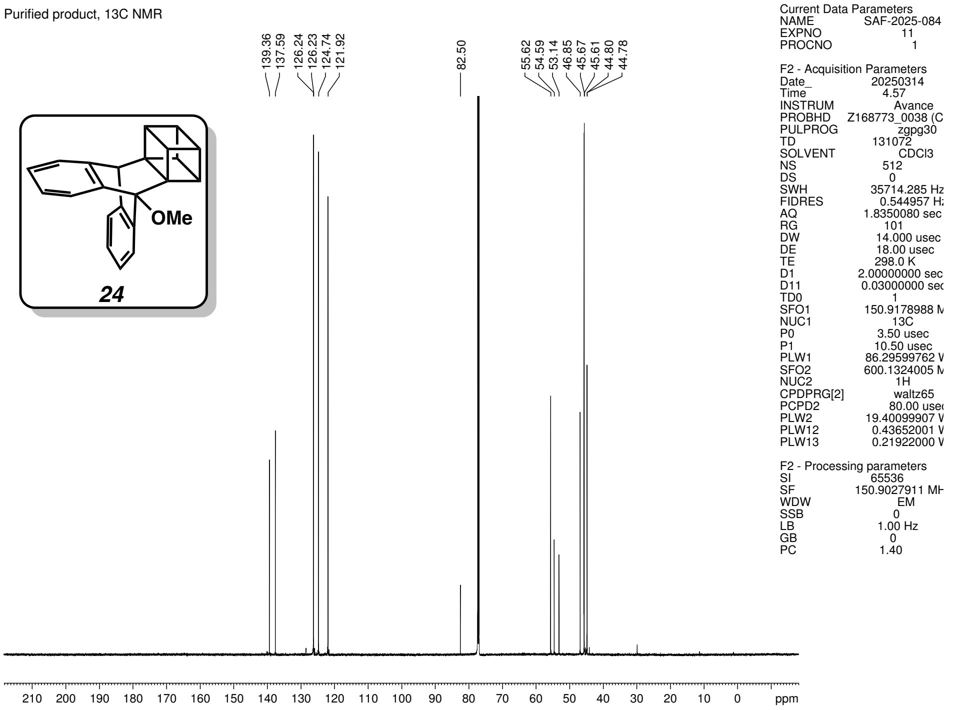

Fig. 33. ^{13}C NMR spectrum of compound **24** in $CDCl_3$ (150 MHz).

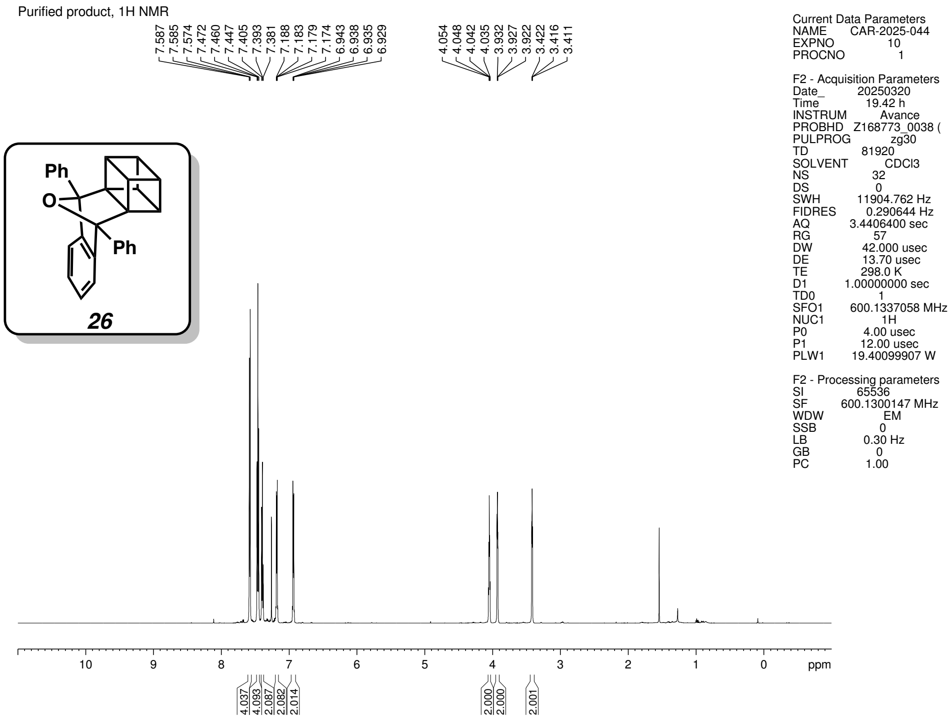

Fig. 34. ^{1}H NMR spectrum of compound **26** in $CDCl_3$ (600 MHz).

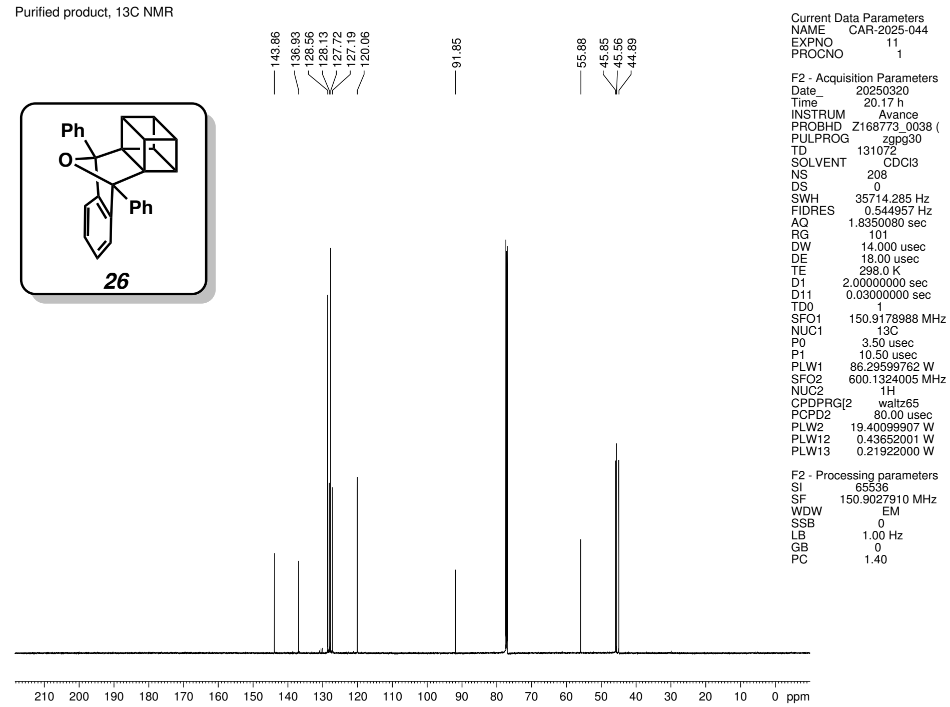

Fig. 35. ^{13}C NMR spectrum of compound **26** in $CDCl_3$ (150 MHz).

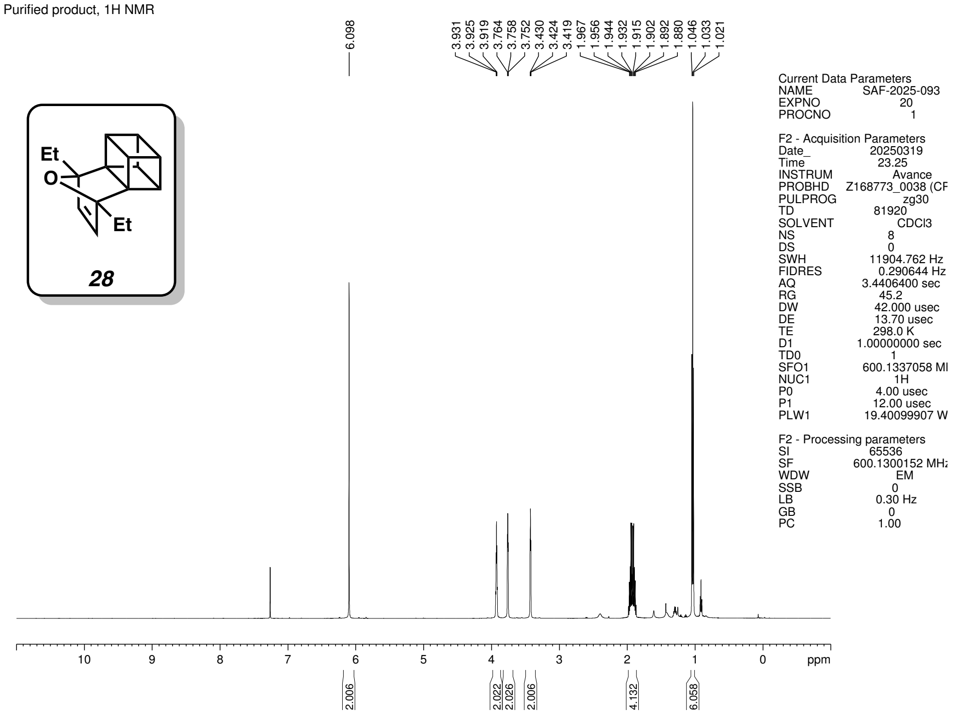

Fig. 36. ^{1}H NMR spectrum of compound **28** in $CDCl_3$ (600 MHz).

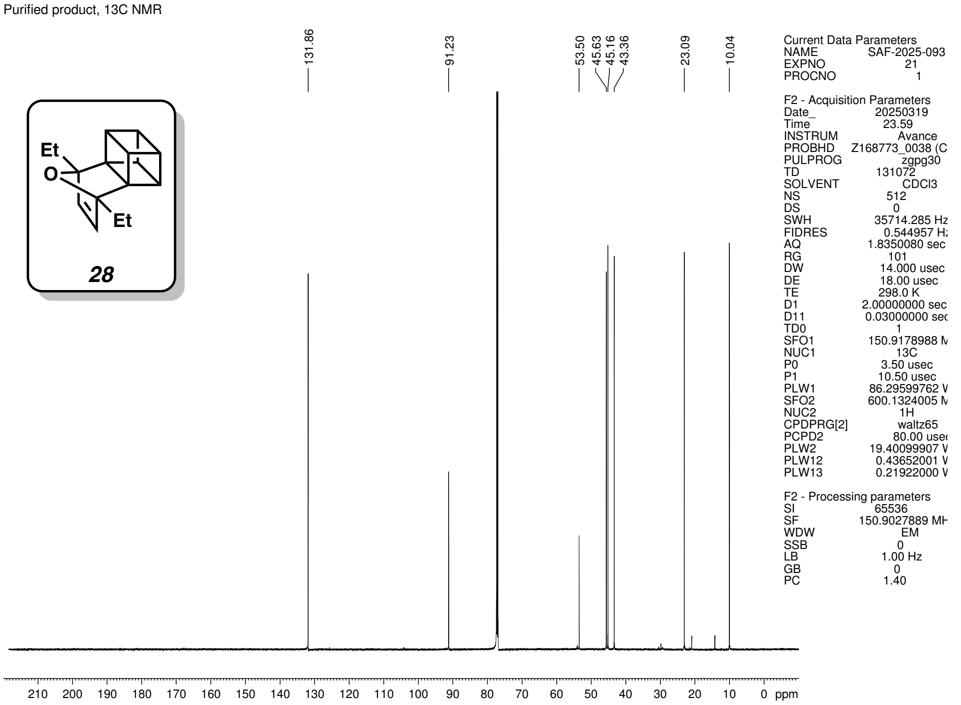

Fig. 37. ^{13}C NMR spectrum of compound **28** in $CDCl_3$ (150 MHz).

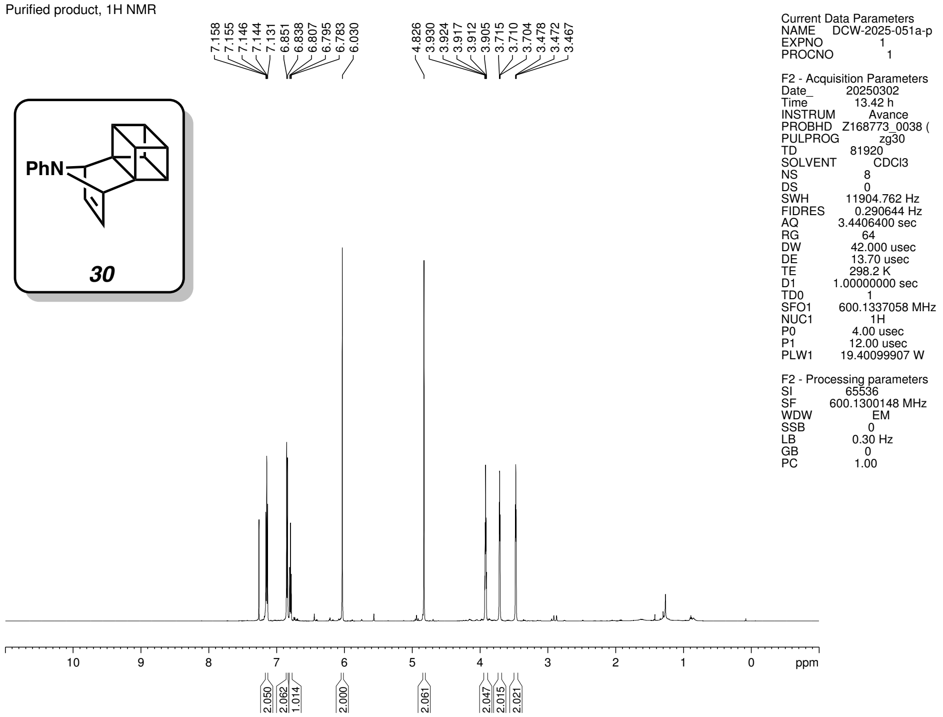

Fig. 38. ^{1}H NMR spectrum of compound **30** in $CDCl_3$ (600 MHz).

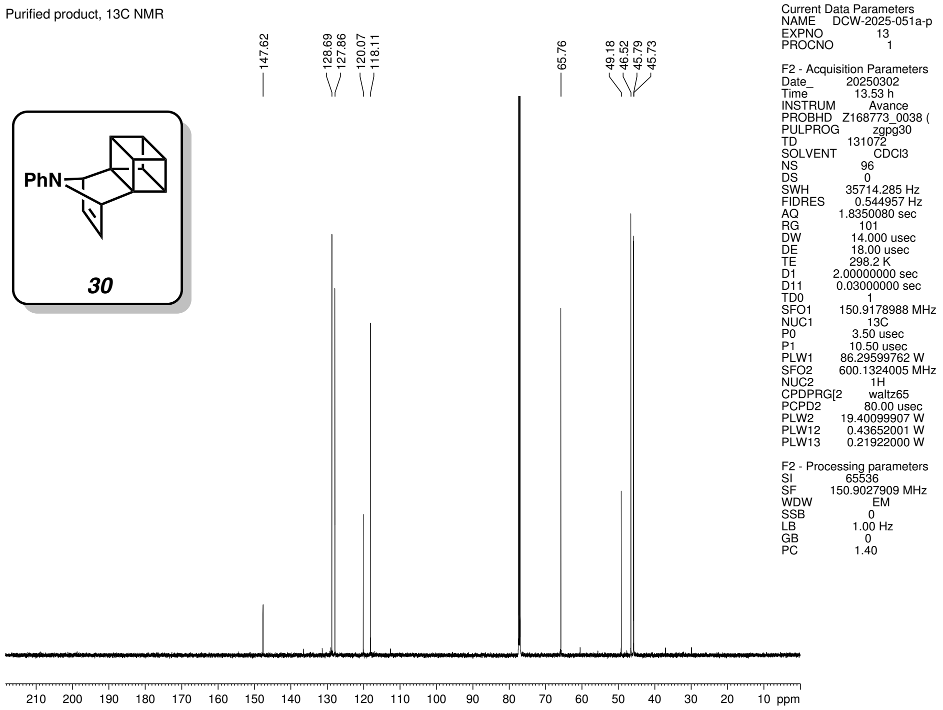

Fig. 39. ^{13}C NMR spectrum of compound **30** in $CDCl_3$ (150 MHz).

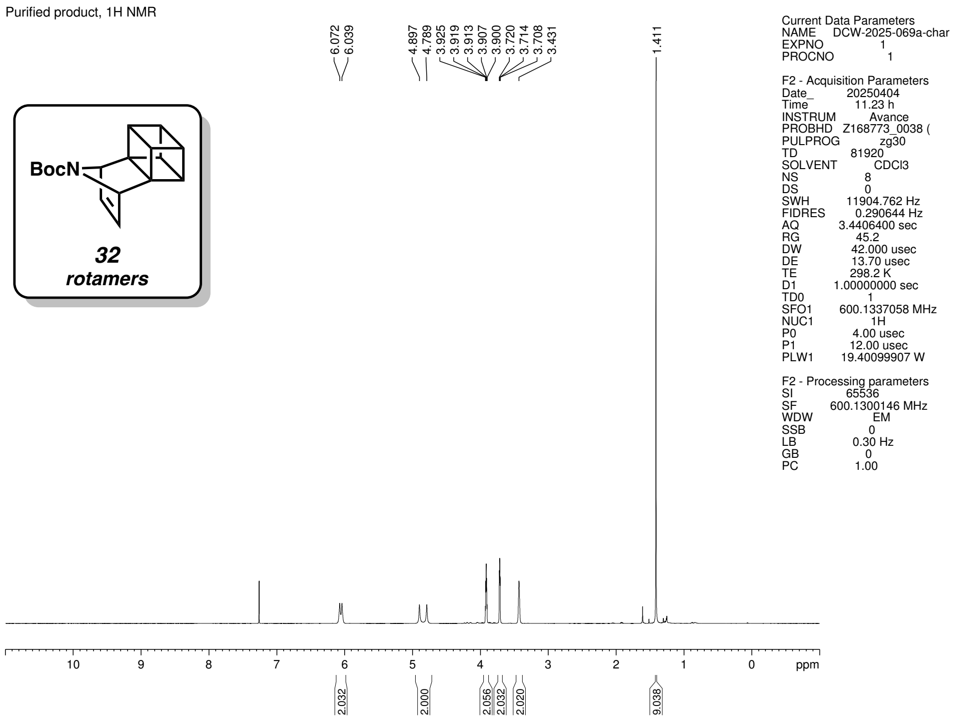

Fig. 40. ^{1}H NMR spectrum of compound **32** in $CDCl_3$ (600 MHz).

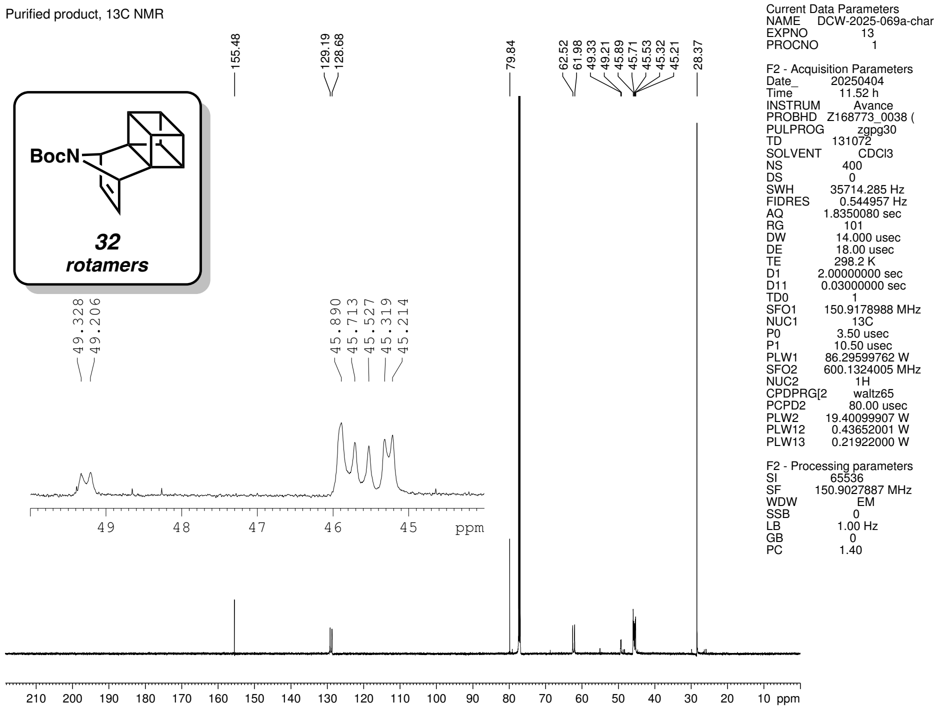

Fig. 41. ^{13}C NMR spectrum of compound **32** in $CDCl_3$ (150 MHz).

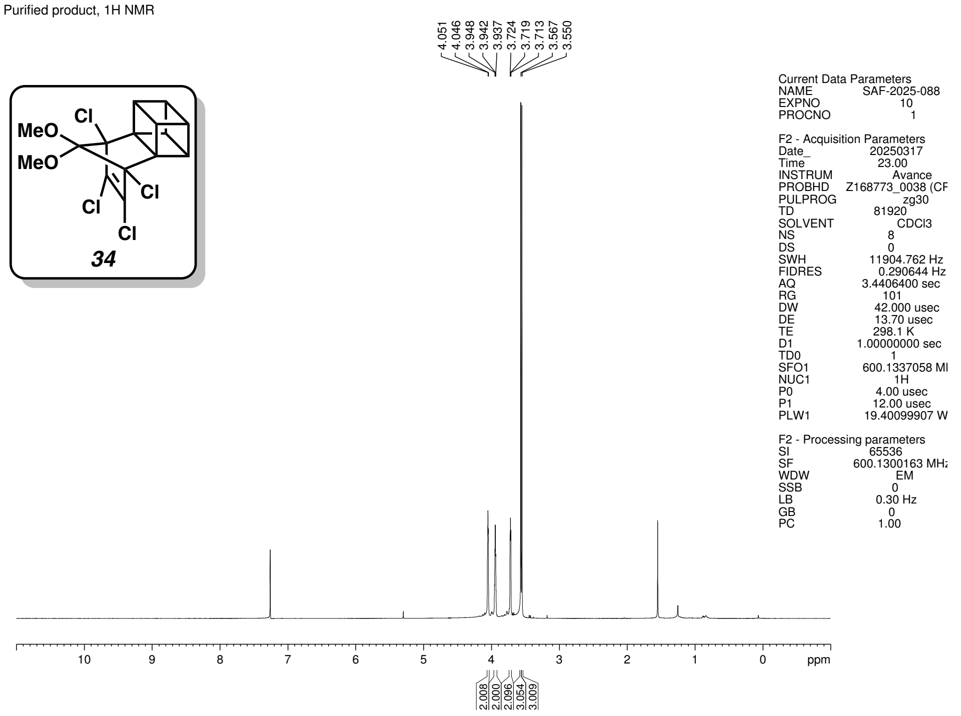

Fig. 42. ^{1}H NMR spectrum of compound **34** in $CDCl_3$ (600 MHz).

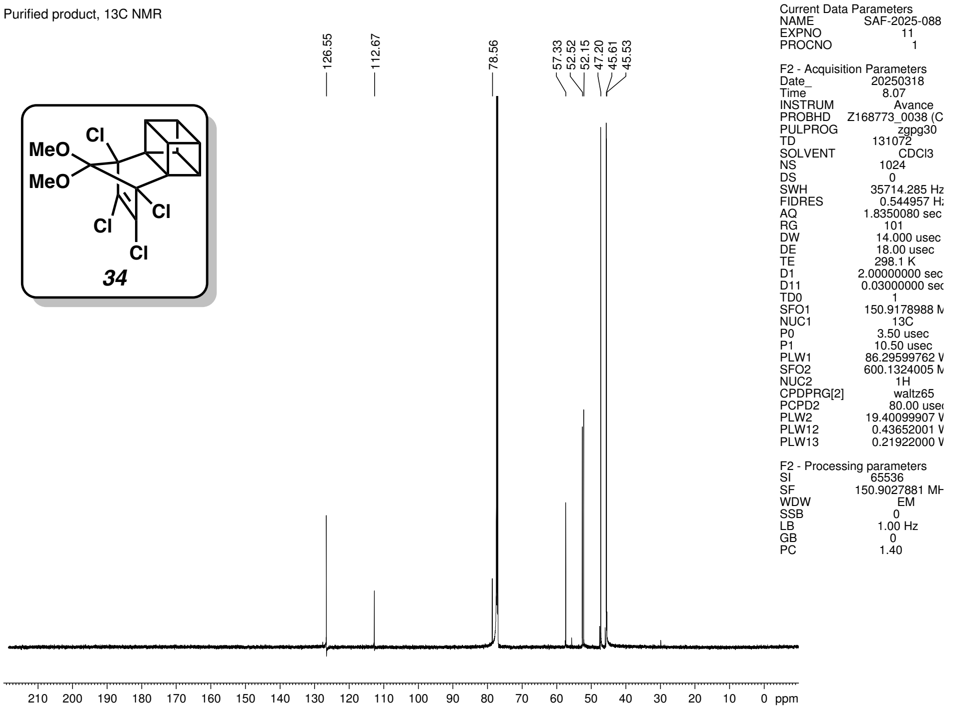

Fig. 43. ^{13}C NMR spectrum of compound **34** in $CDCl_3$ (150 MHz).

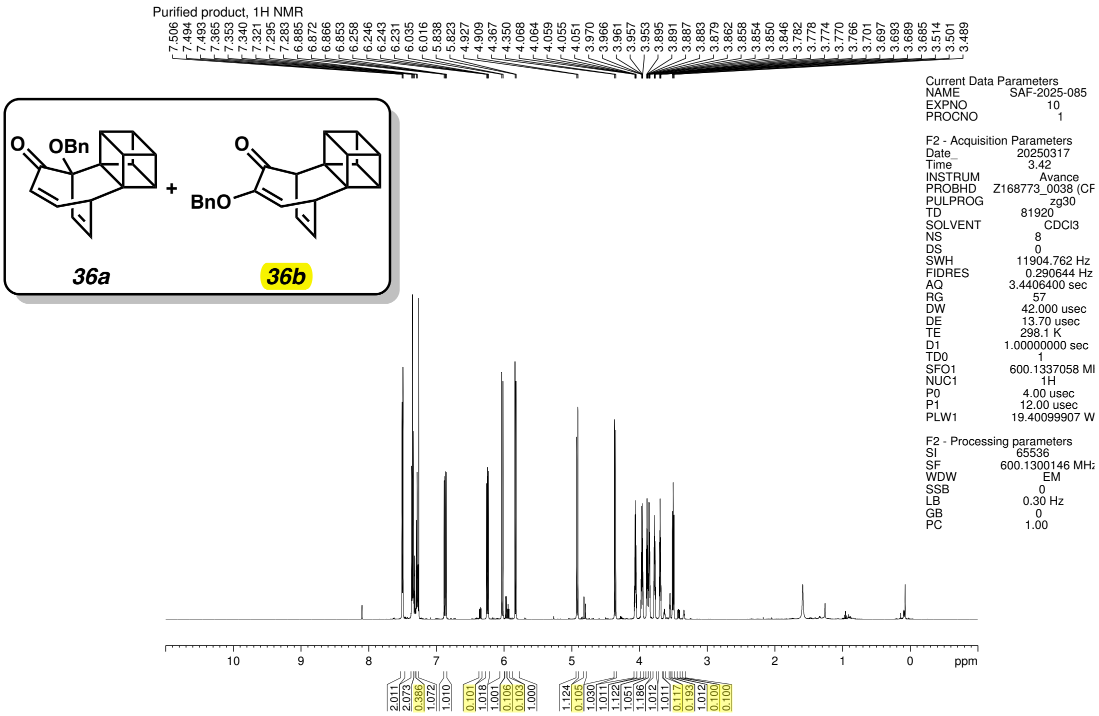

Fig. 44. ^{1}H NMR spectrum of compound **36a** and **36b** in $CDCl_3$ (600 MHz).

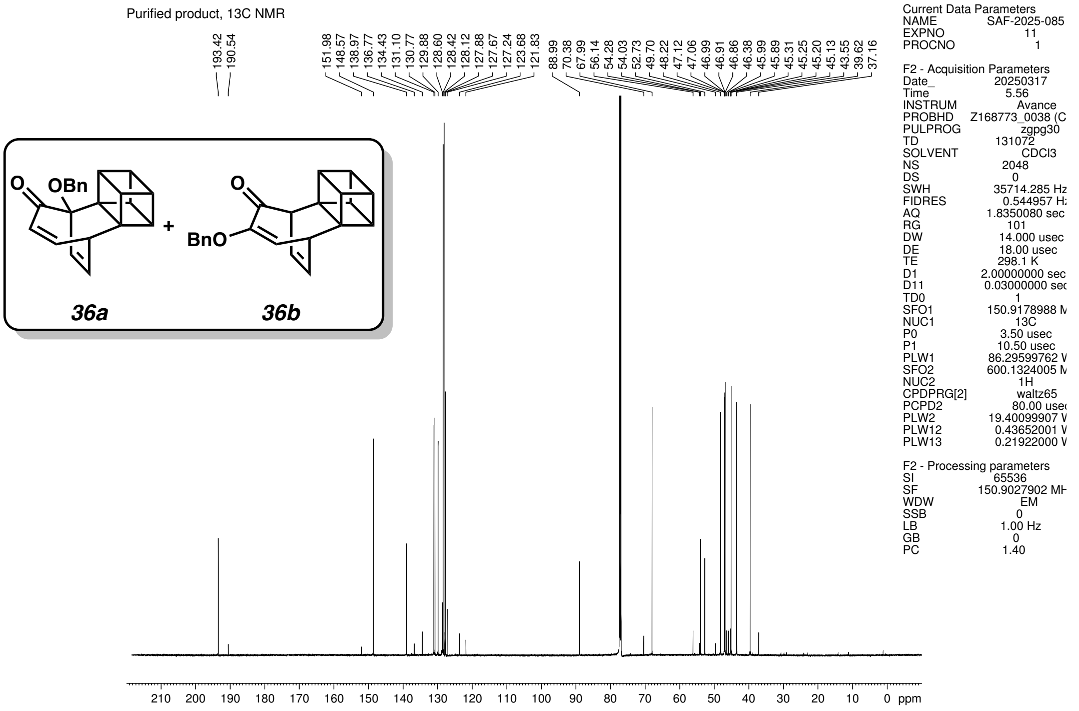

Fig. 45. ^{13}C NMR spectrum of compound **36a** and **36b** in $CDCl_3$ (150 MHz).

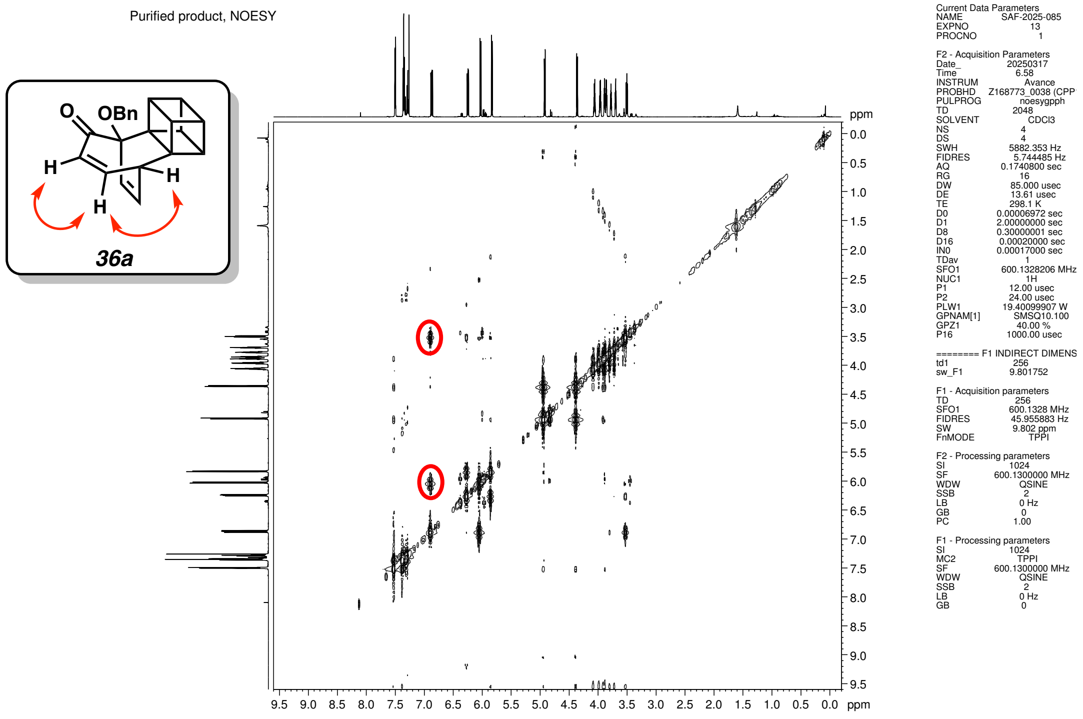

Fig. 46. NOESY spectrum of compound **36a** in $CDCl_3$ (600 MHz).

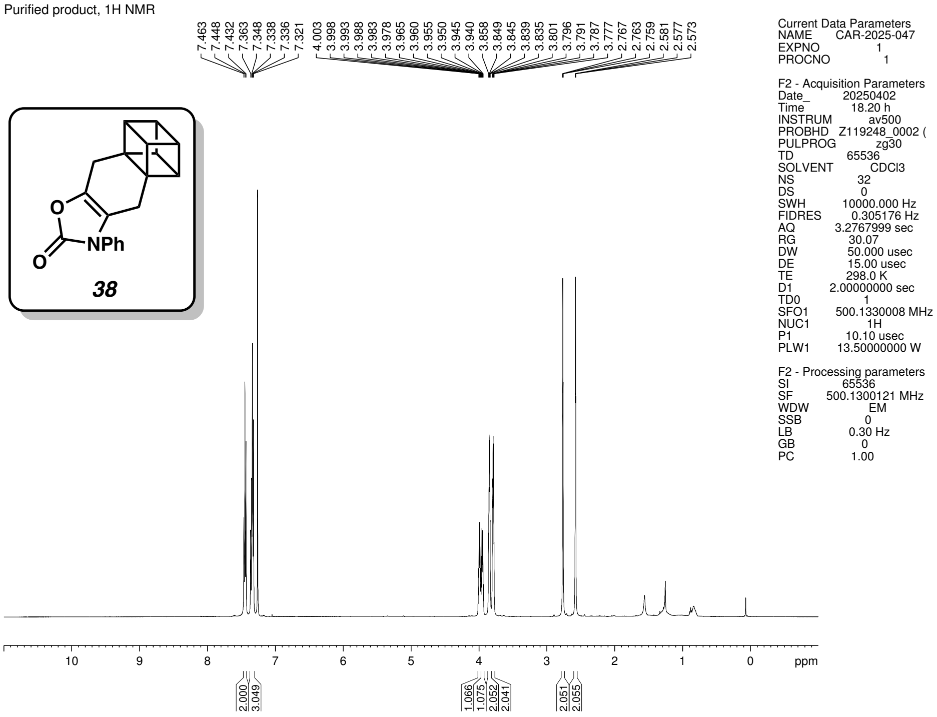

Fig. 47. ^{1}H NMR spectrum of compound **38** in $CDCl_3$ (500 MHz).

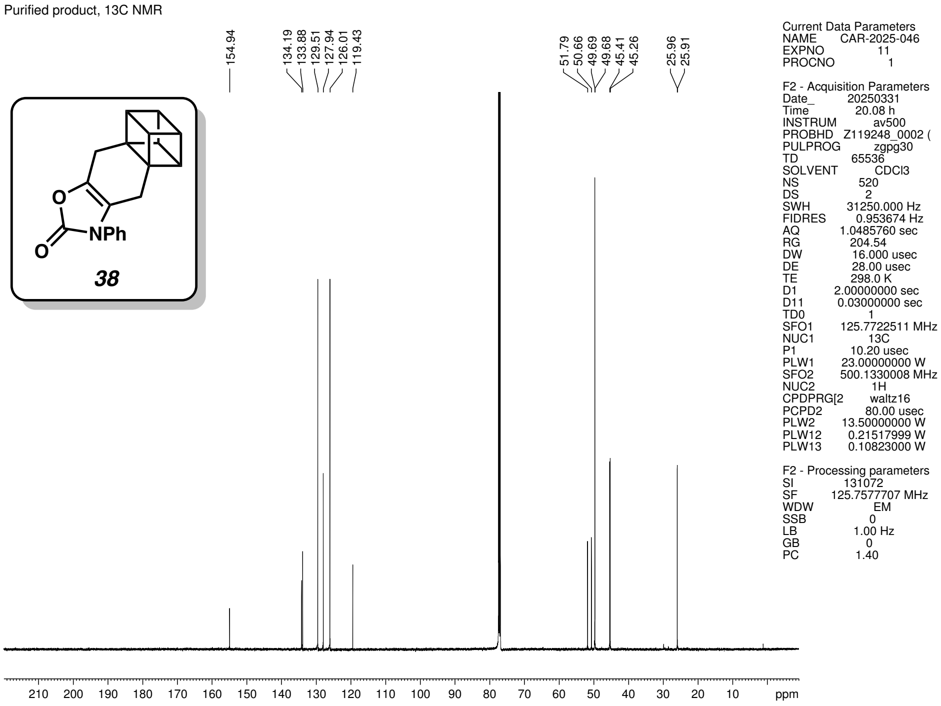

Fig. 48. ^{13}C NMR spectrum of compound **38** in $CDCl_3$ (125 MHz).

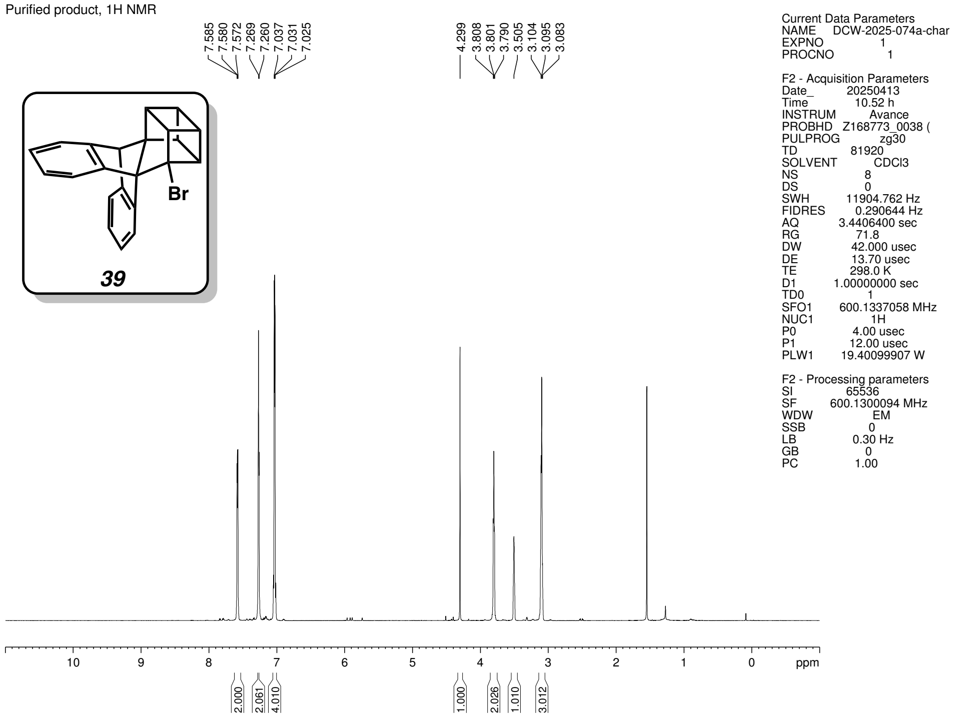

Fig. 49. ^{1}H NMR spectrum of compound **39** in $CDCl_3$ (600 MHz).

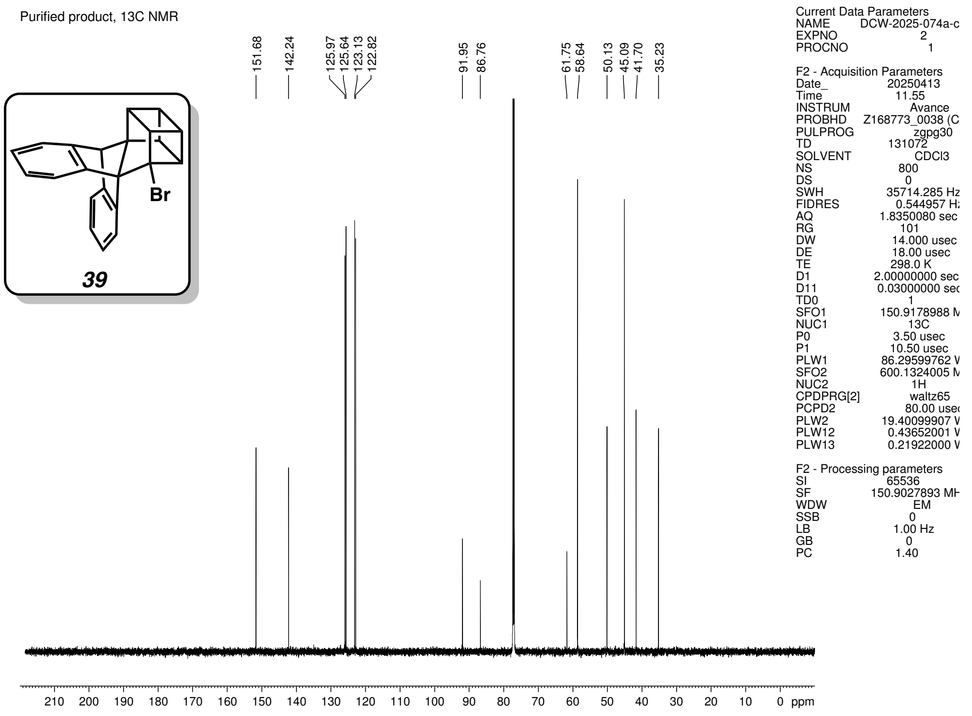

Fig. 50. ^{13}C NMR spectrum of compound **39** in $CDCl_3$ (150 MHz).

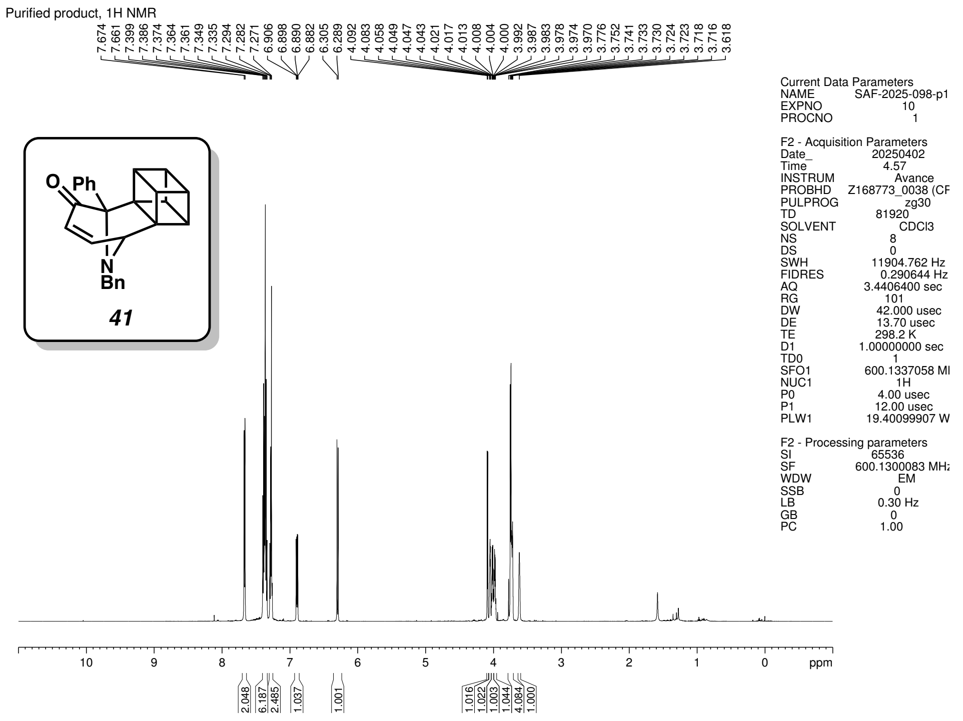

Fig. 51. ^{1}H NMR spectrum of compound **41** in $CDCl_3$ (600 MHz).

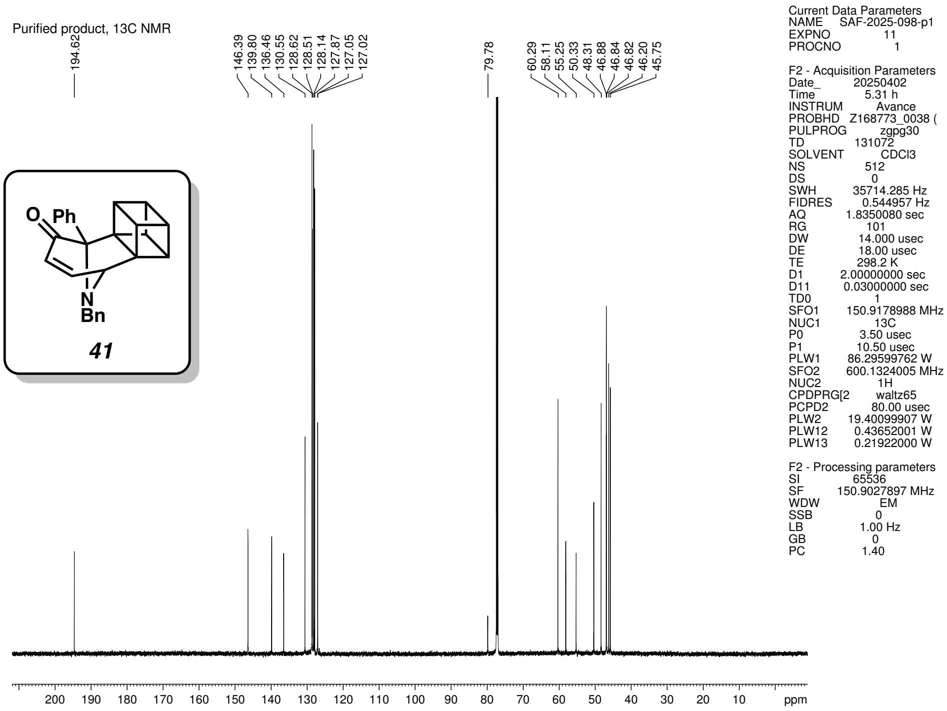

Fig. 52. ^{13}C NMR spectrum of compound **41** in $CDCl_3$ (150 MHz).

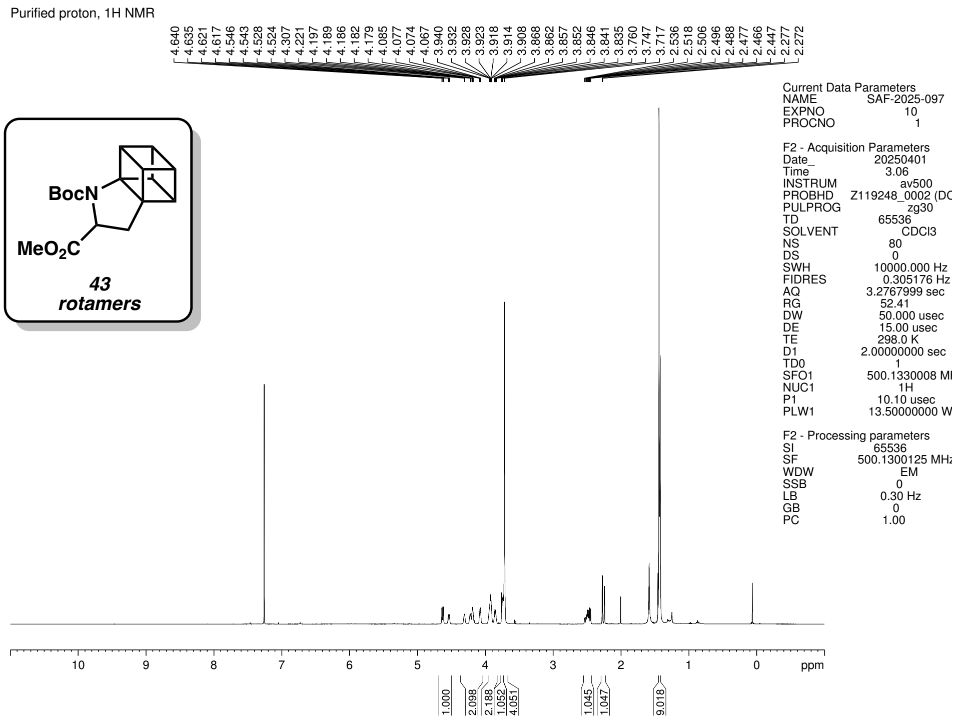

Fig. 53. ^{1}H NMR spectrum of compound **43** in $CDCl_3$ (500 MHz).

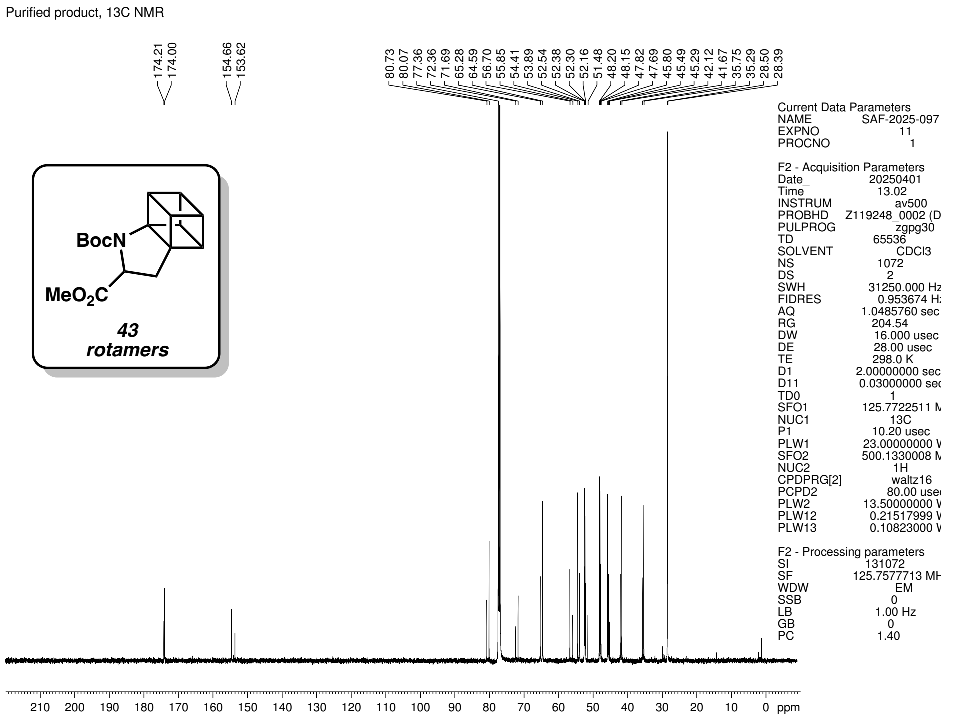

Fig. 54. ^{13}C NMR spectrum of compound **43** in $CDCl_3$ (125 MHz).

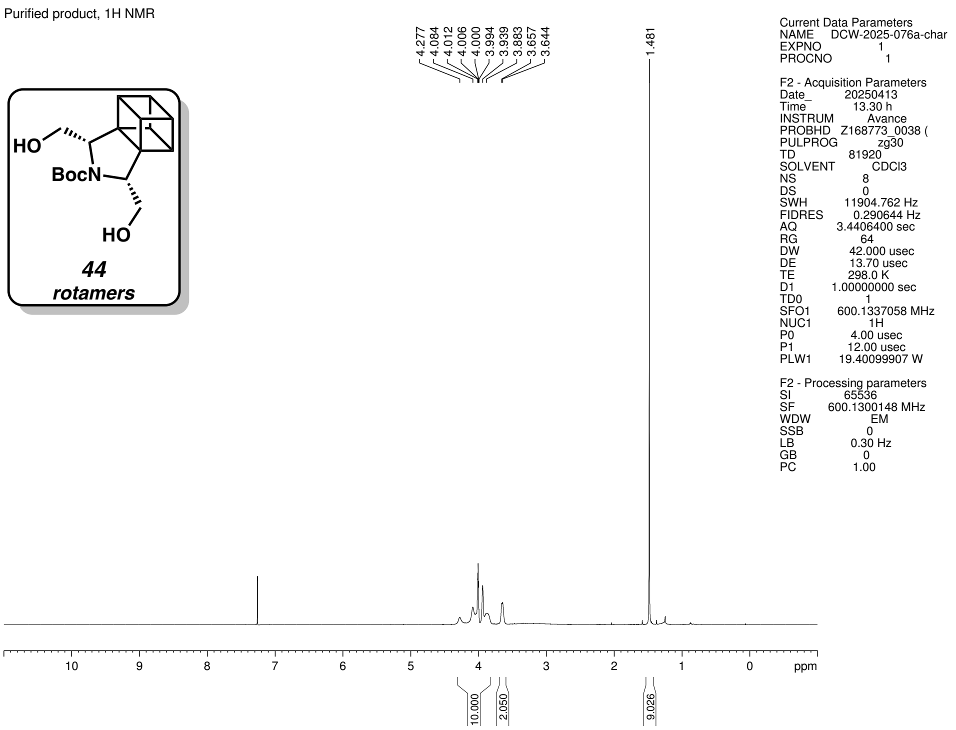

Fig. 55. ^{1}H NMR spectrum of compound **44** in $CDCl_3$ (600 MHz).

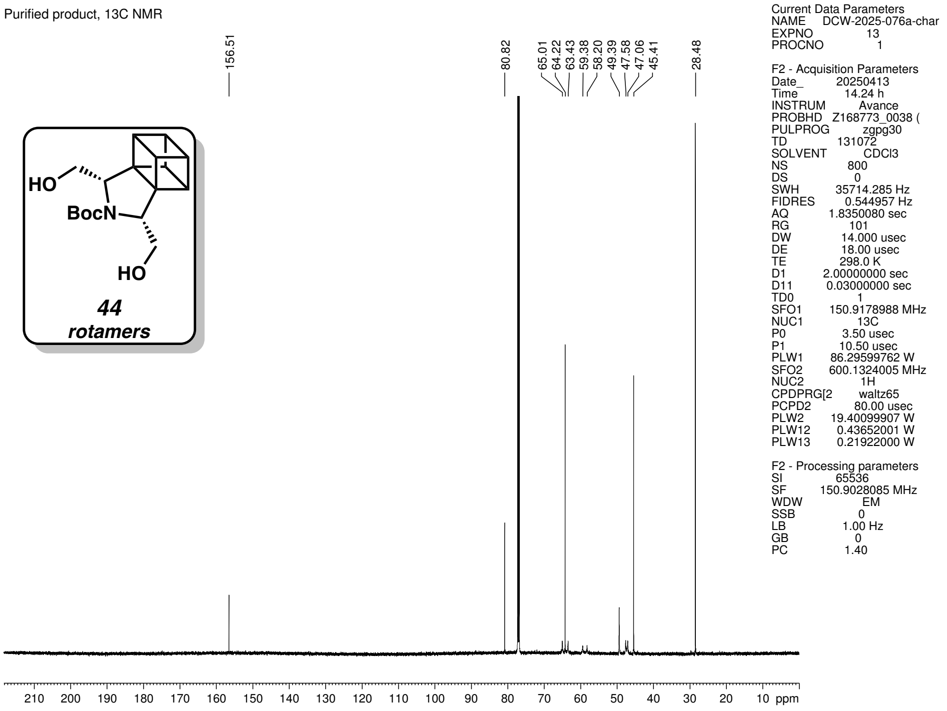

Fig. 56. ^{13}C NMR spectrum of compound **44** in $CDCl_3$ (150 MHz).

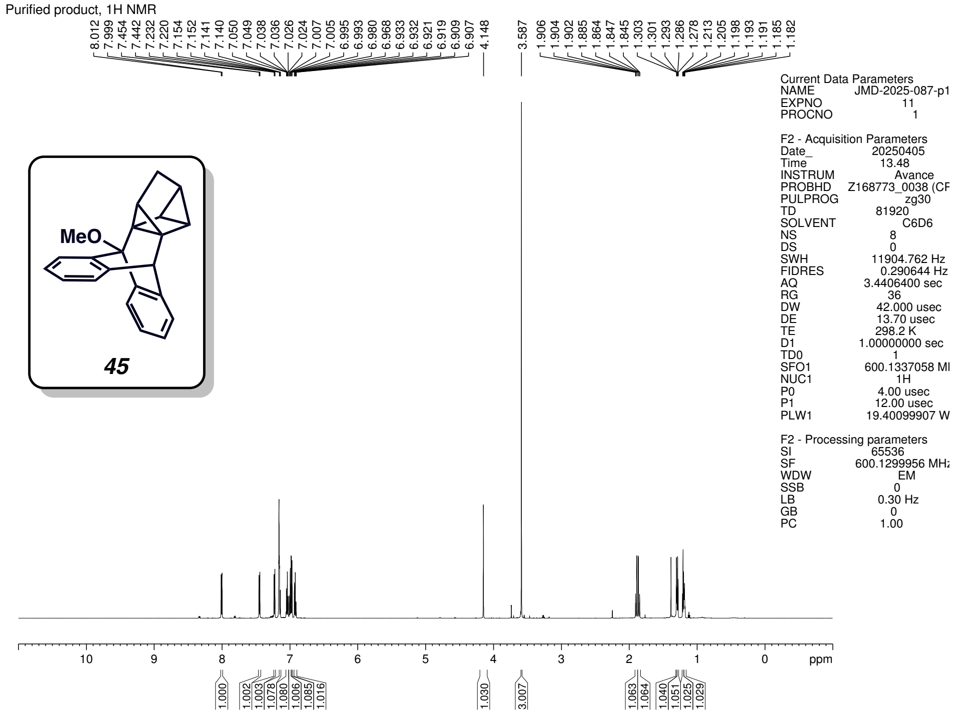

Fig. 57. ^{1}H NMR spectrum of compound **45** in C_6D_6 (600 MHz).

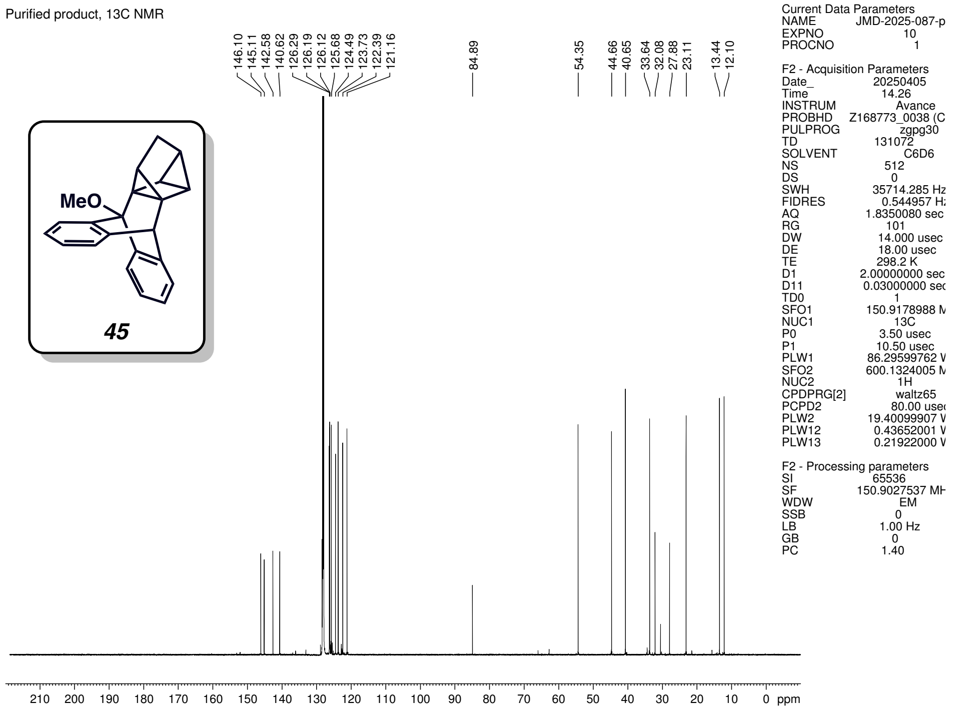

Fig. 58. ^{13}C NMR spectrum of compound **45** in C_6D_6 (150 MHz).

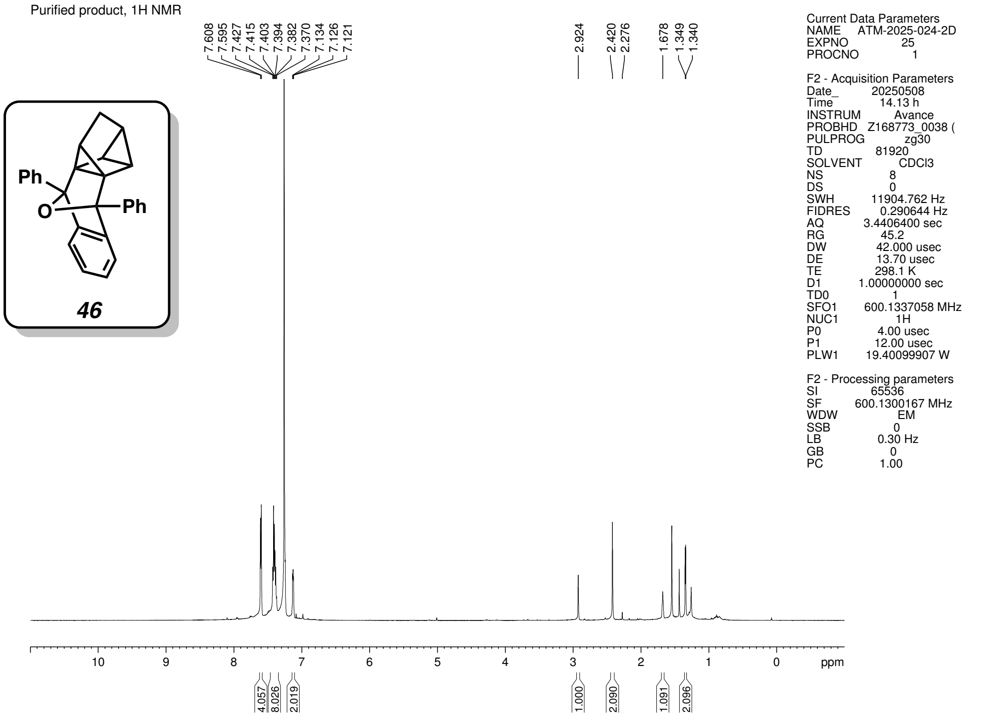

Fig. 59. ^{1}H NMR spectrum of compound **46** in $CDCl_3$ (600 MHz).

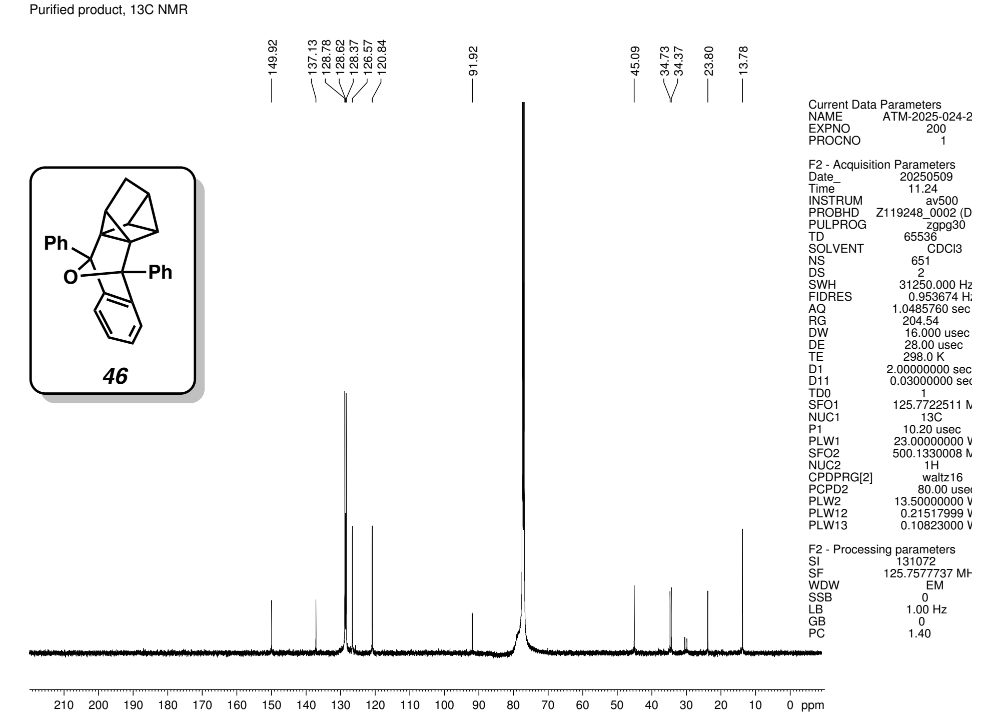

Fig. 60. ^{13}C NMR spectrum of compound **46** in $CDCl_3$ (125 MHz).

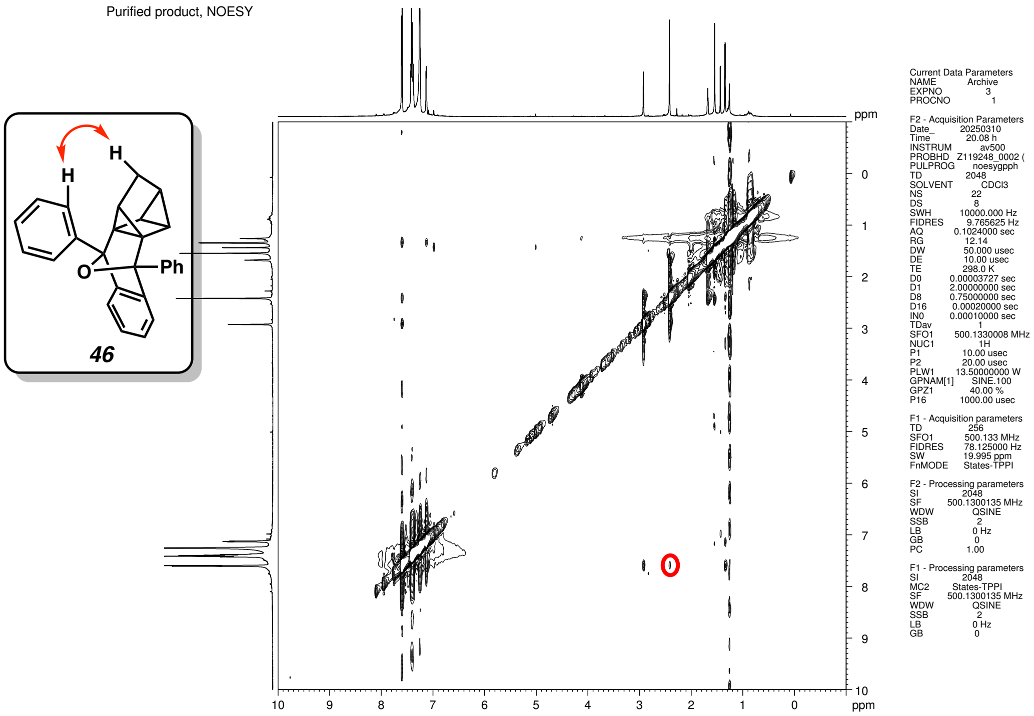

Fig. 61. NOESY spectrum of compound **46** in $CDCl_3$ (500 MHz).

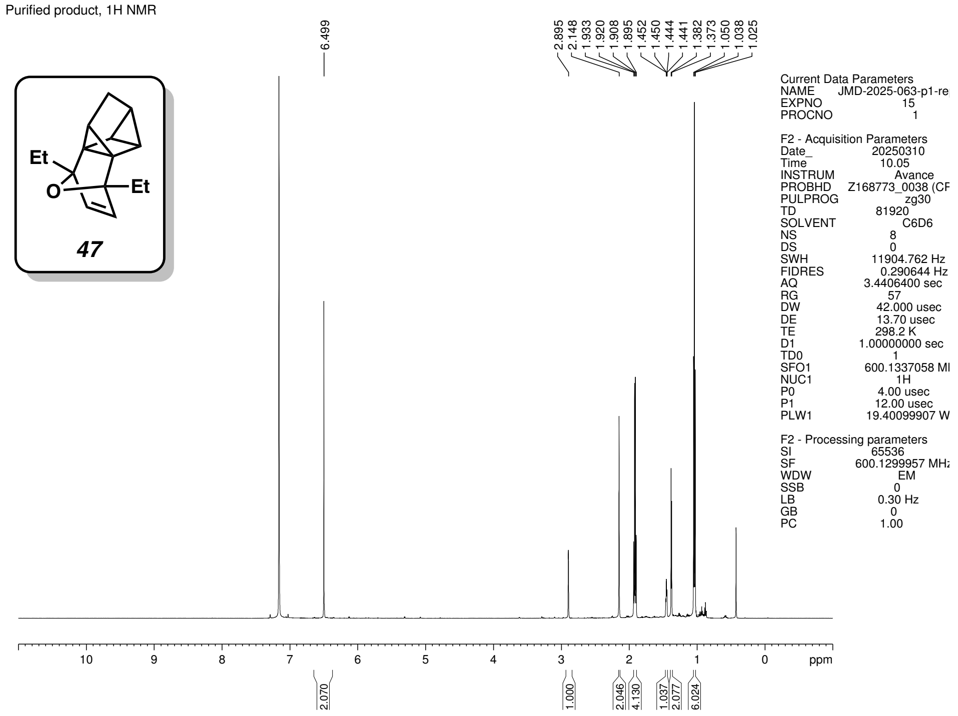

Fig. 62. ^{1}H NMR spectrum of compound **47** in C_6D_6 (600 MHz).

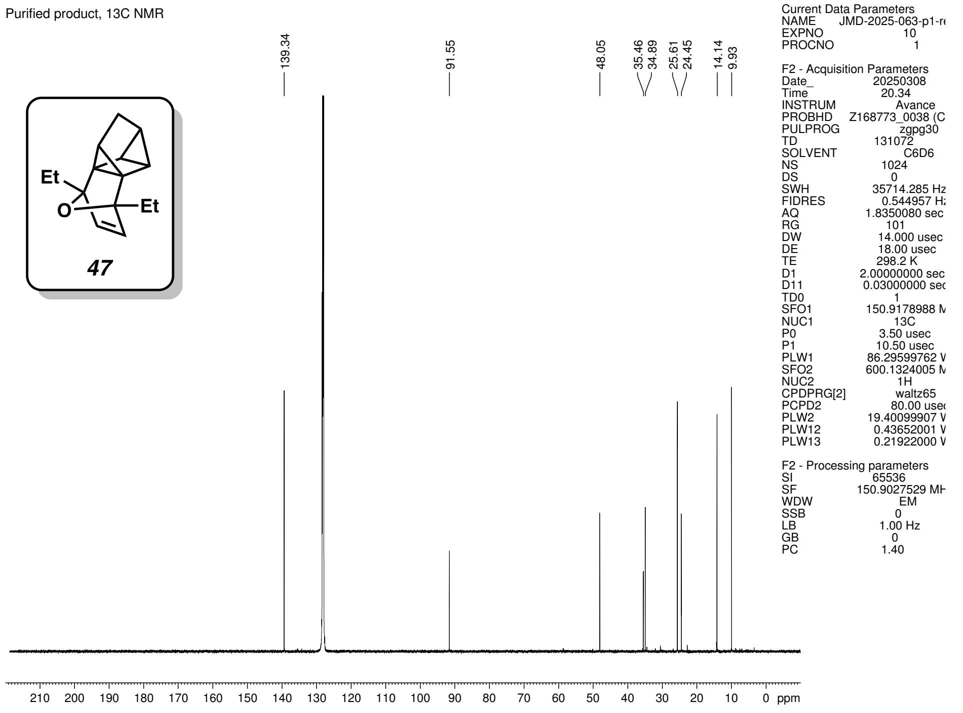

Fig. 63. ^{13}C NMR spectrum of compound **47** in C_6D_6 (150 MHz).

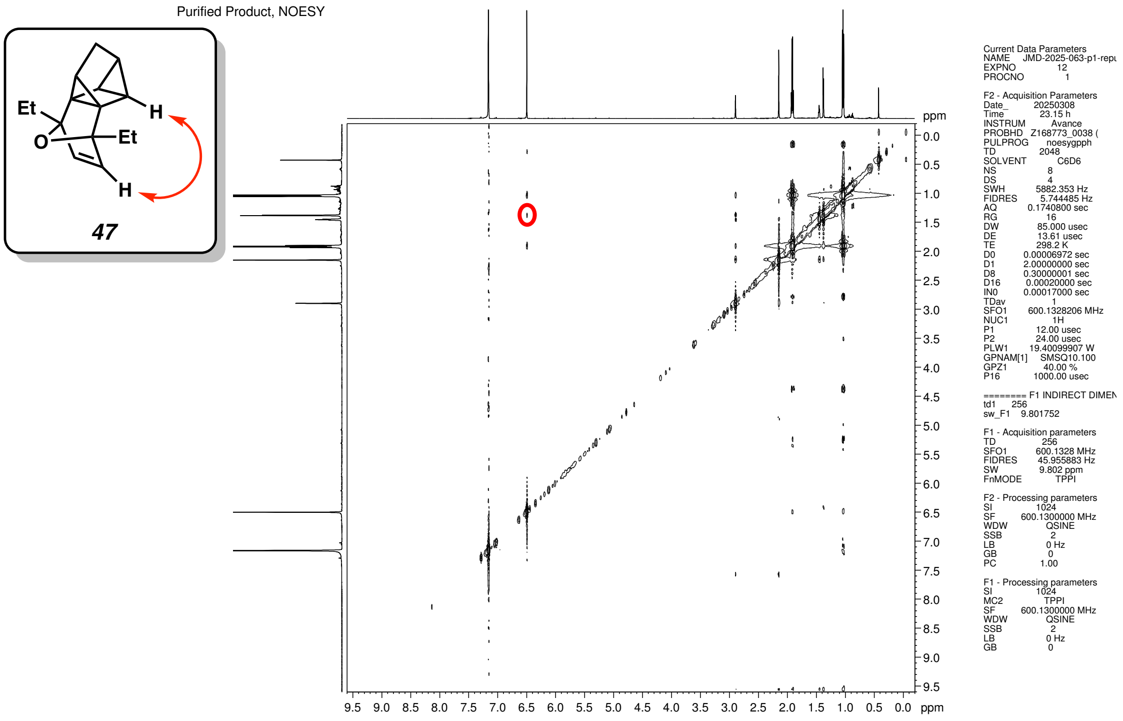

Fig. 64. NOESY spectrum of compound **47** in C_6D_6 (600 MHz).

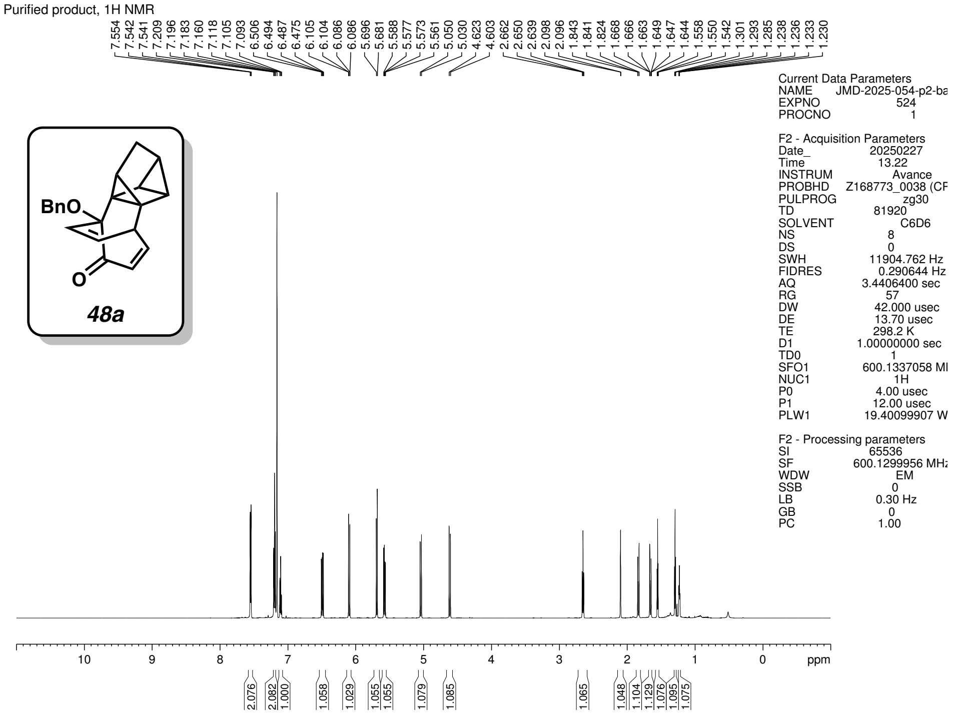

Fig. 65. ^{1}H NMR spectrum of compound **48a** in C_6D_6 (600 MHz).

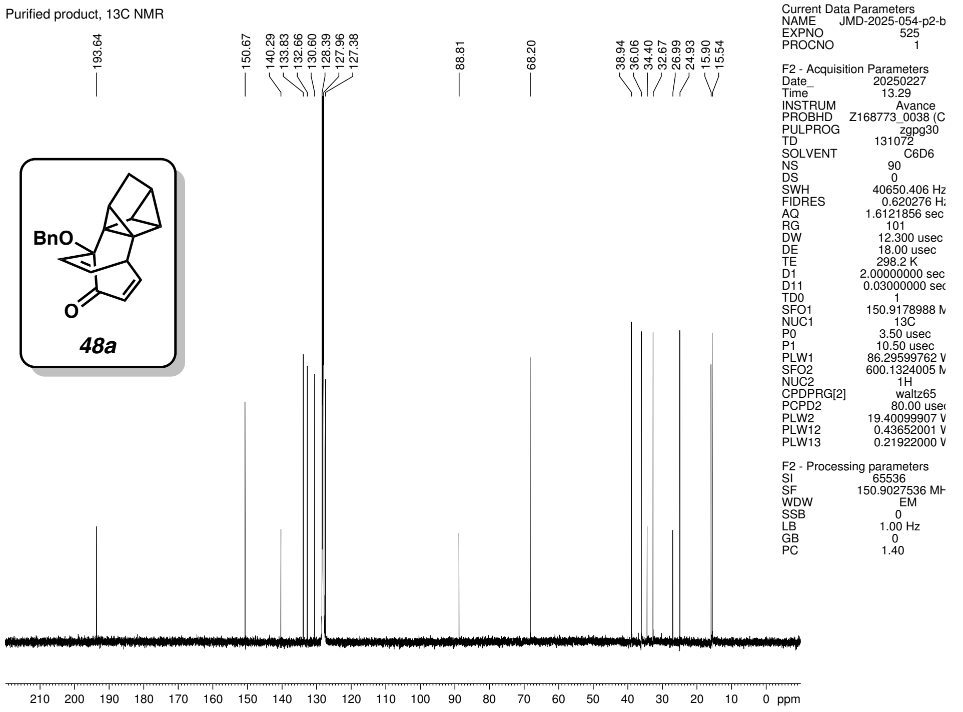

Fig. 66. ^{13}C NMR spectrum of compound **48a** in C_6D_6 (150 MHz).

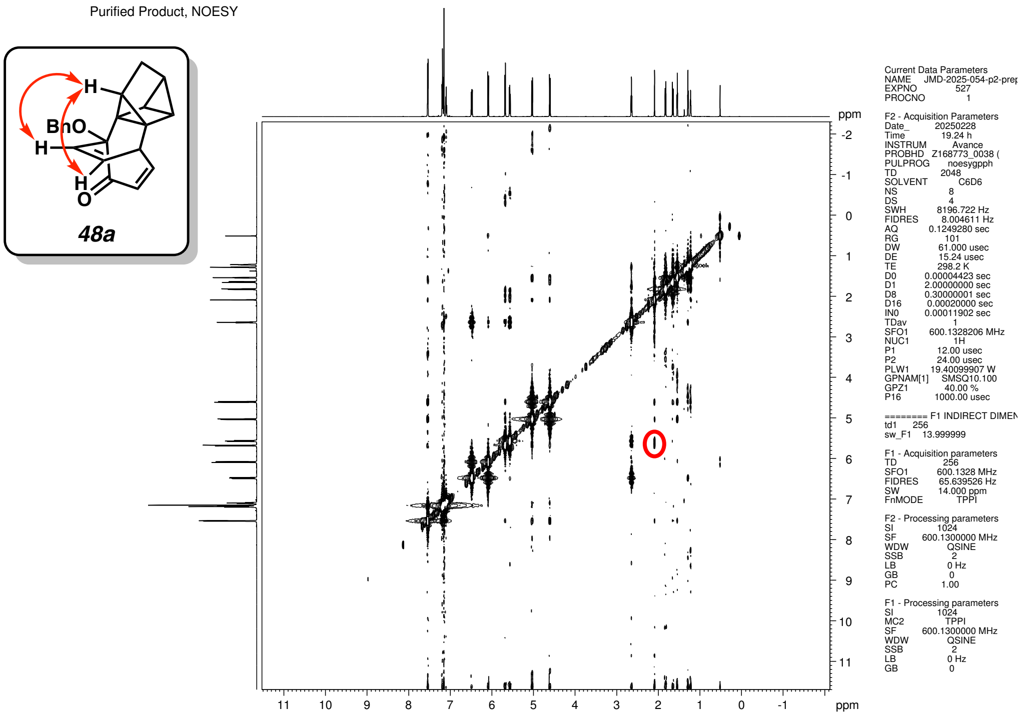

Fig. 67. NOESY spectrum of compound **48a** in C_6D_6 (600 MHz).

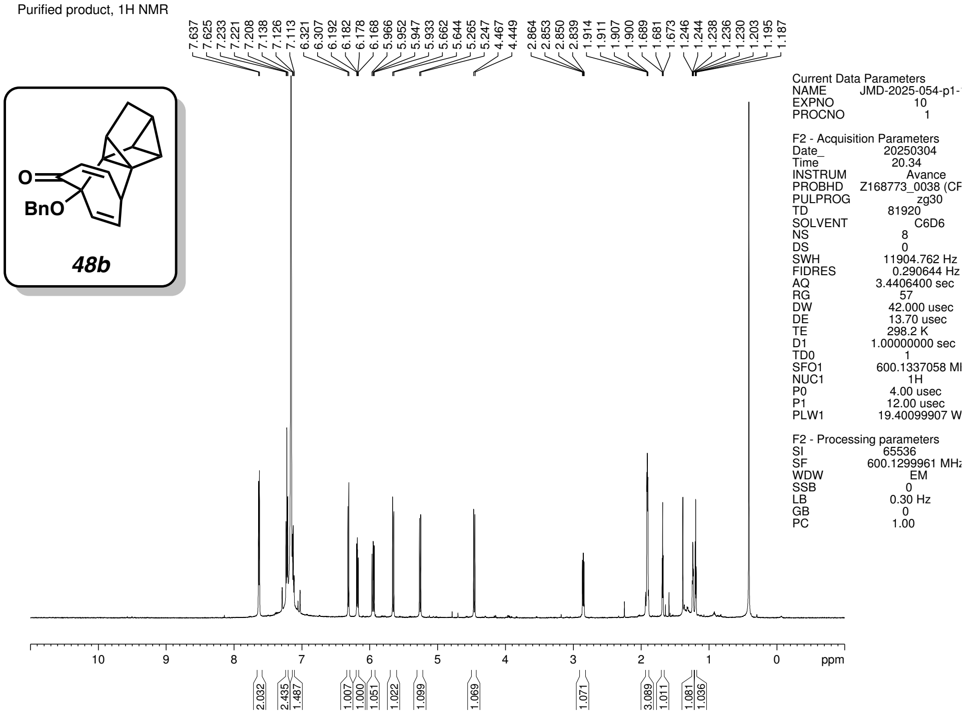

Fig. 68. ^{1}H NMR spectrum of compound **48b** in C_6D_6 (600 MHz).

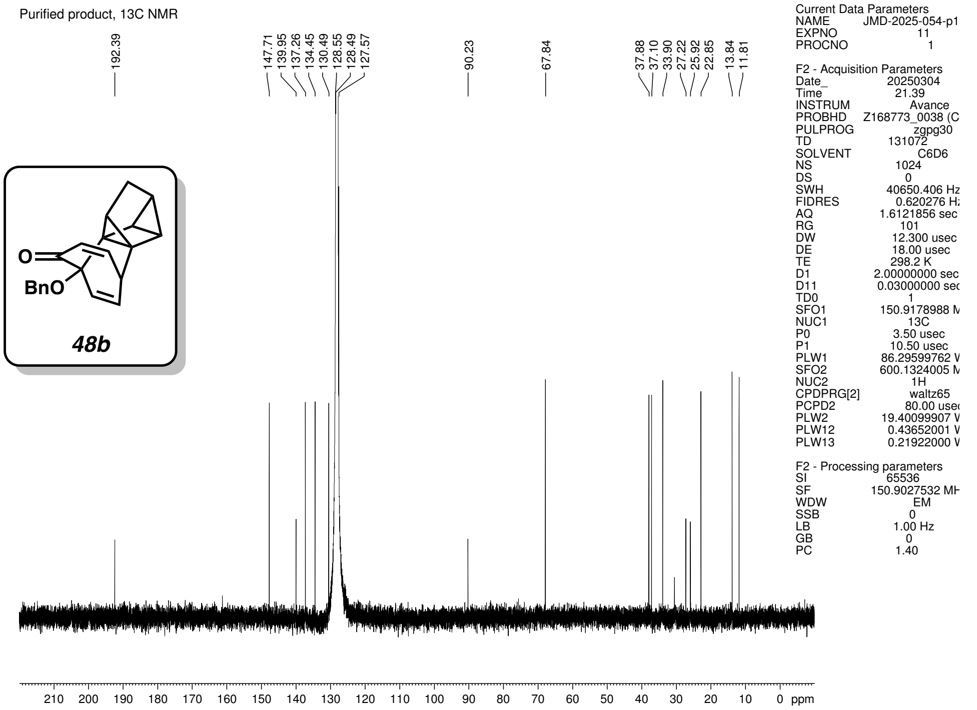

Fig. 69. ^{13}C NMR spectrum of compound **48b** in C_6D_6 (150 MHz).

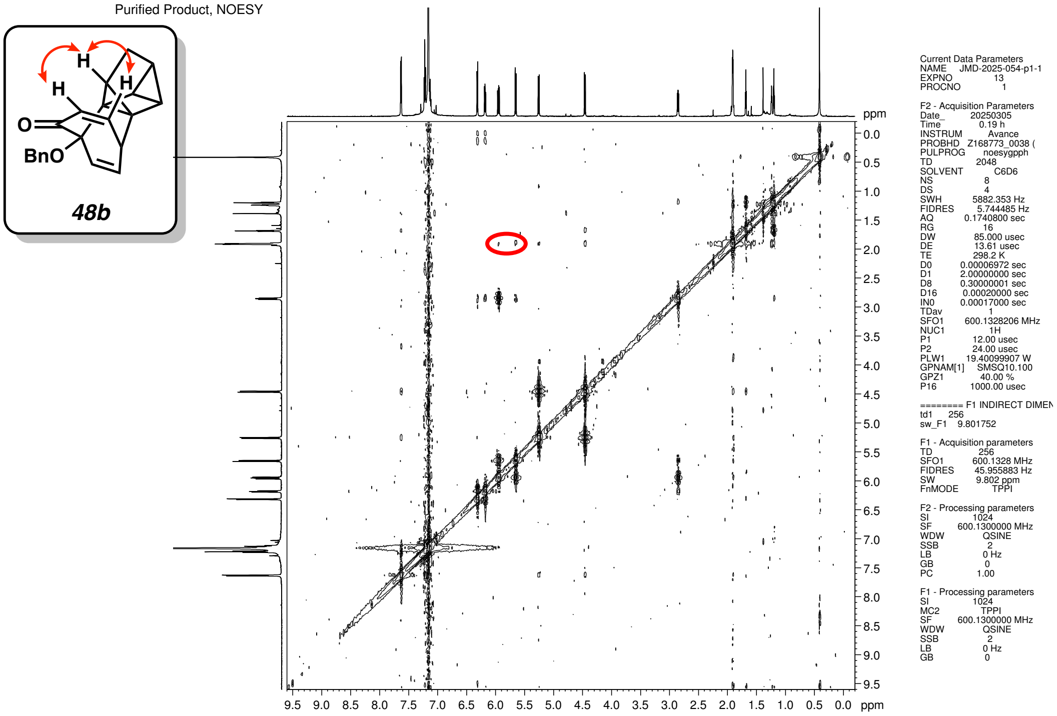

Fig. 70. NOESY spectrum of compound **48b** in C_6D_6 (600 MHz).

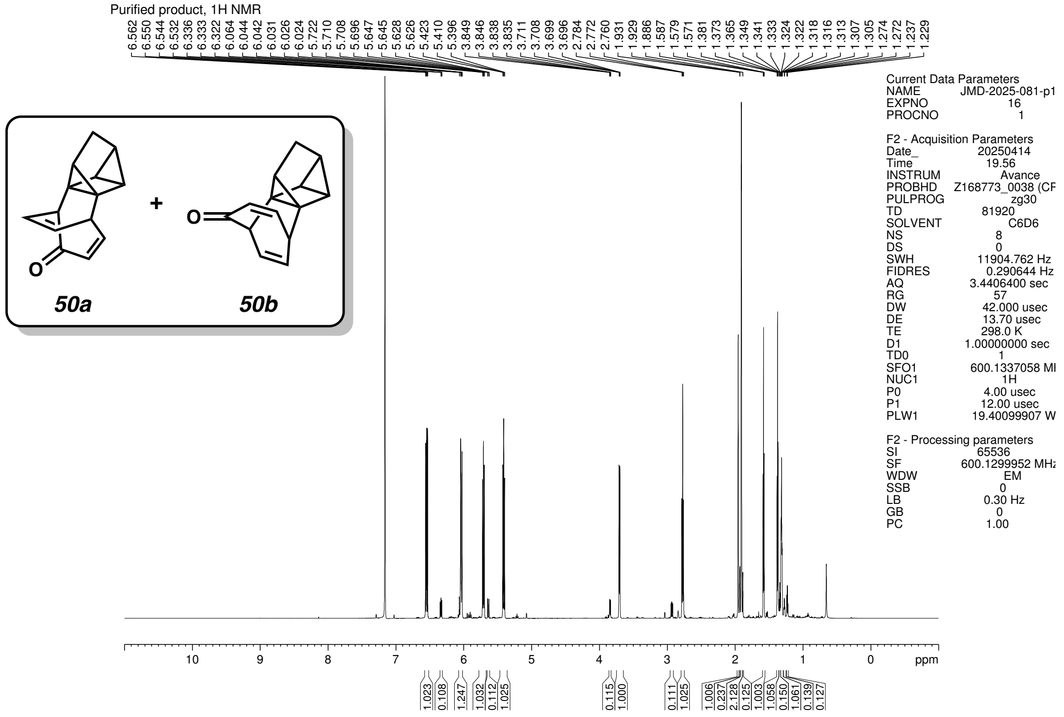

Fig. 71. ^{1}H NMR spectrum of compound **50a** and **50b** in C_6D_6 (600 MHz).

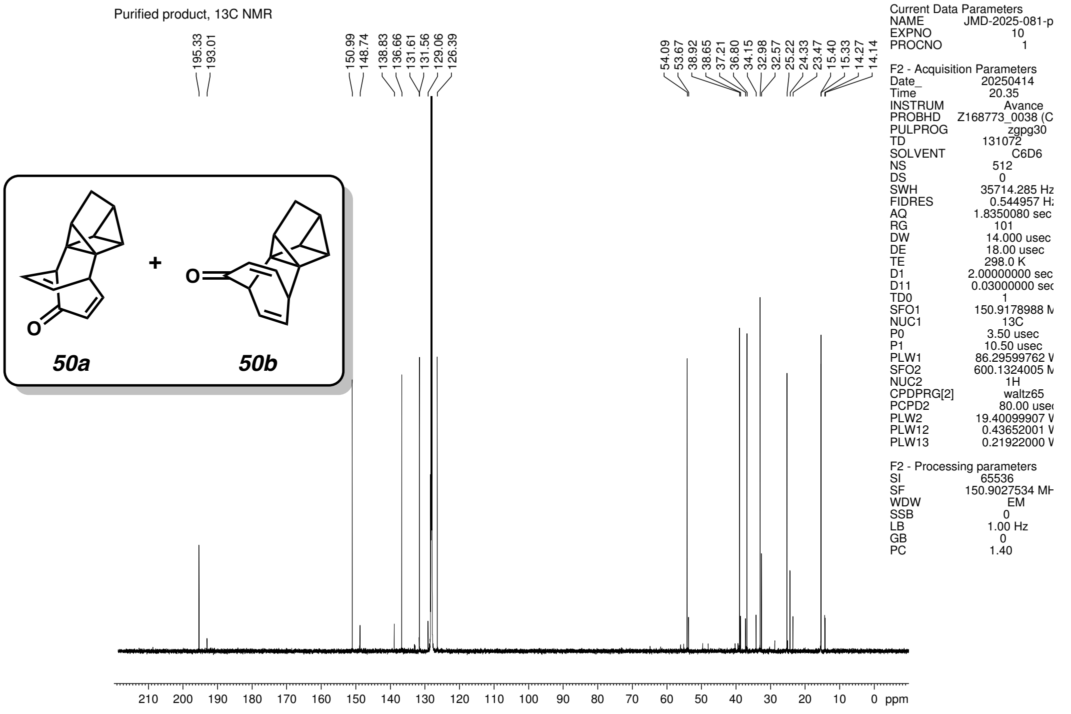

Fig. 72. ^{13}C NMR spectrum of compound **50a** and **50b** in C_6D_6 (150 MHz).

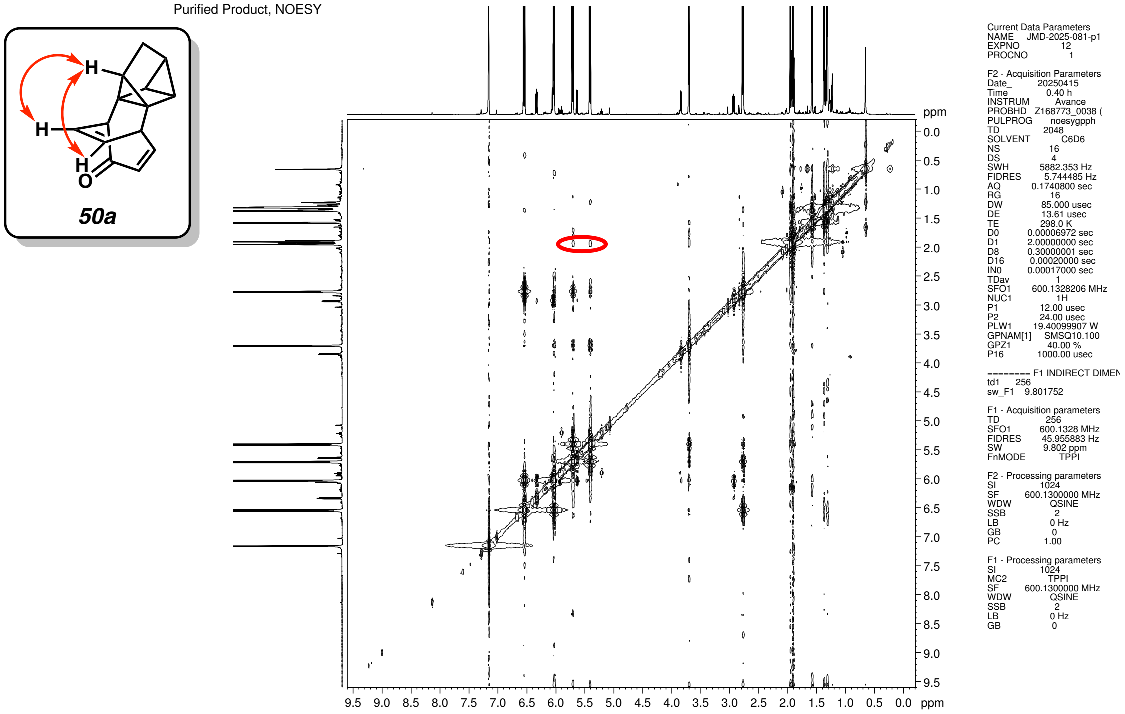

Fig. 73. NOESY spectrum of compound **50a** in C_6D_6 (600 MHz).

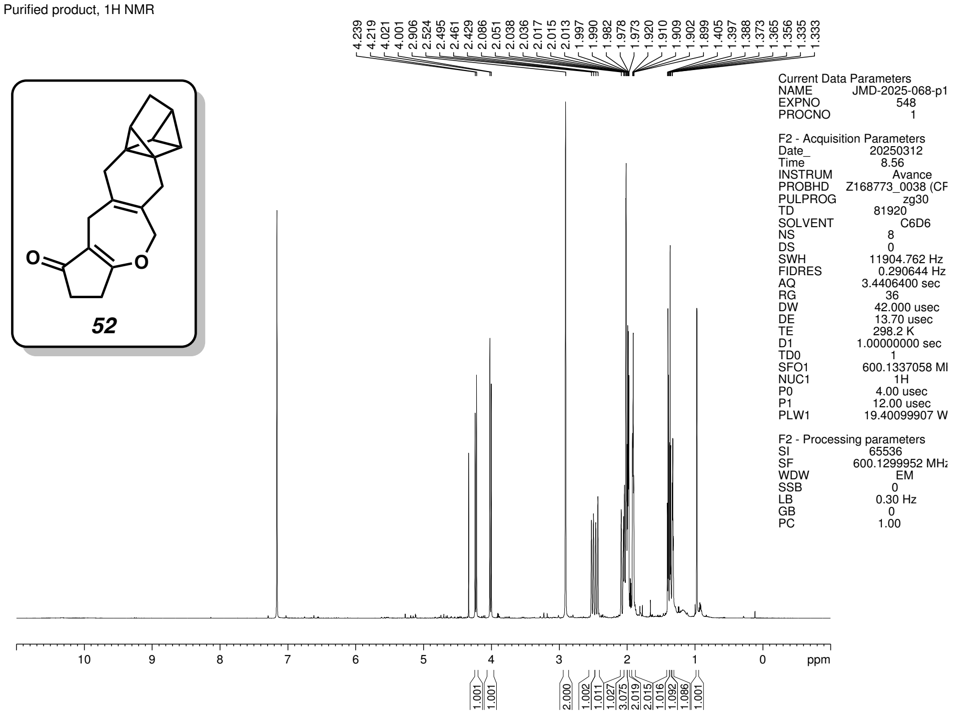

Fig. 74. ^{1}H NMR spectrum of compound **52** in C_6D_6 (600 MHz).

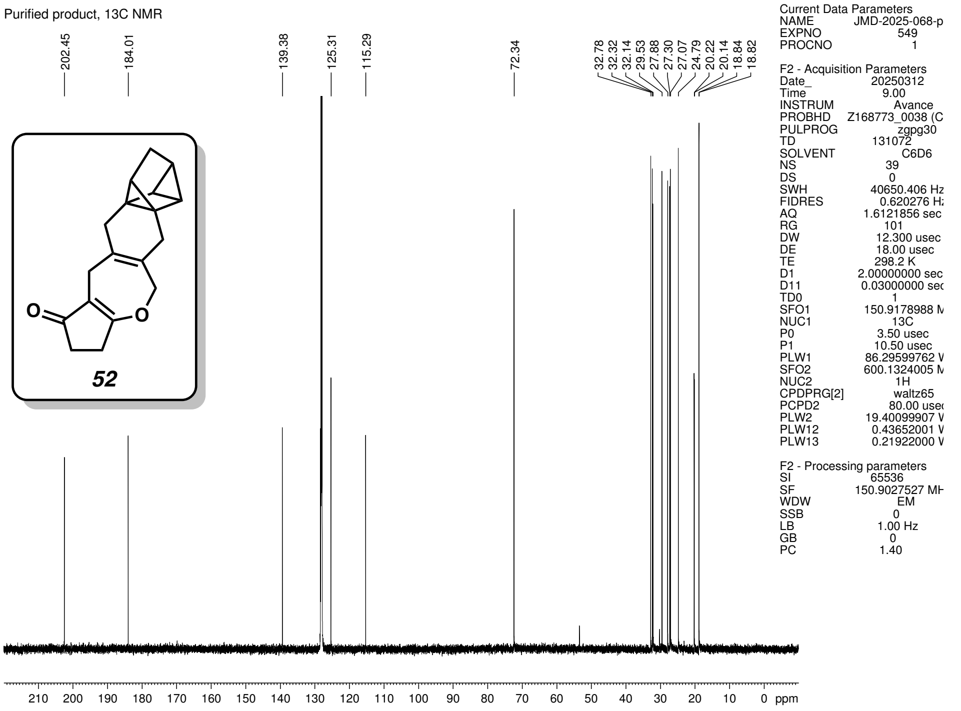

Fig. 75. ^{13}C NMR spectrum of compound **52** in C_6D_6 (150 MHz).

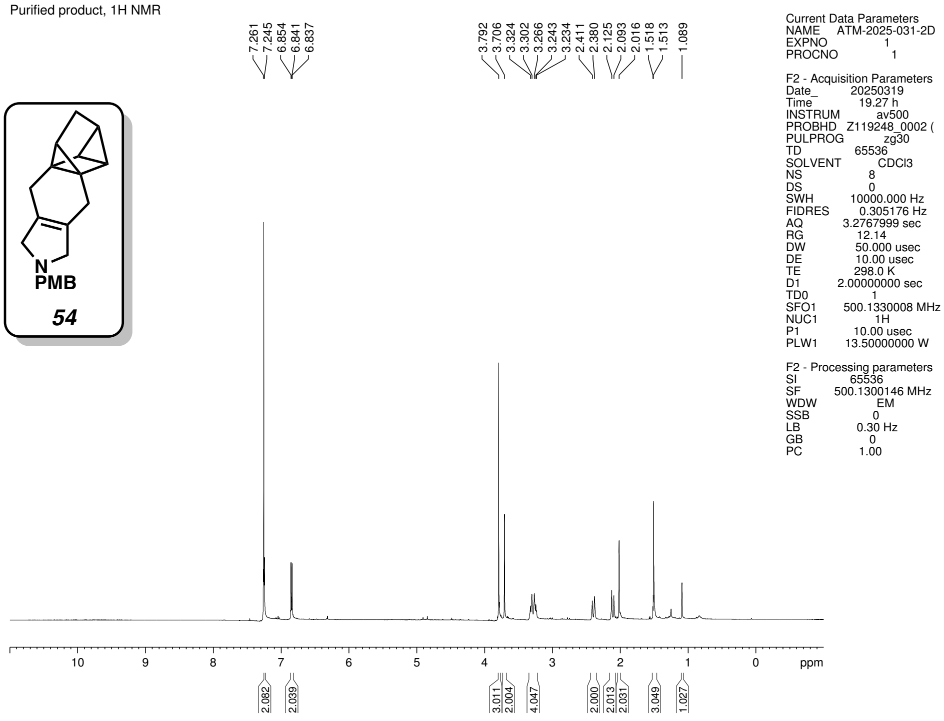

Fig. 76. ^{1}H NMR spectrum of compound **54** in $CDCl_3$ (500 MHz).

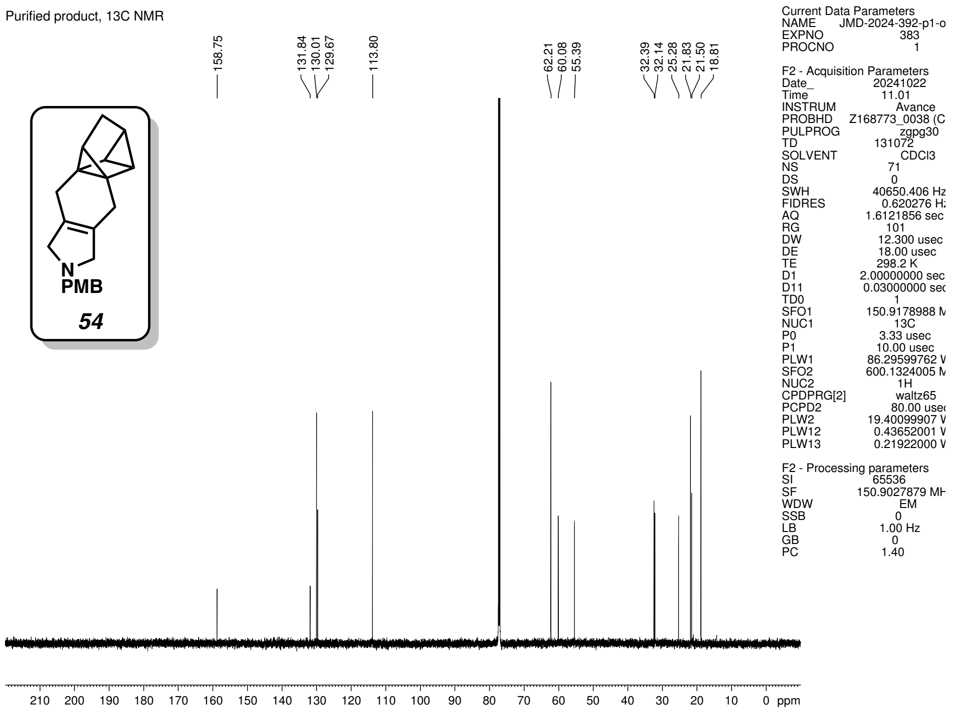

Fig. 77. ^{13}C NMR spectrum of compound **54** in $CDCl_3$ (150 MHz).

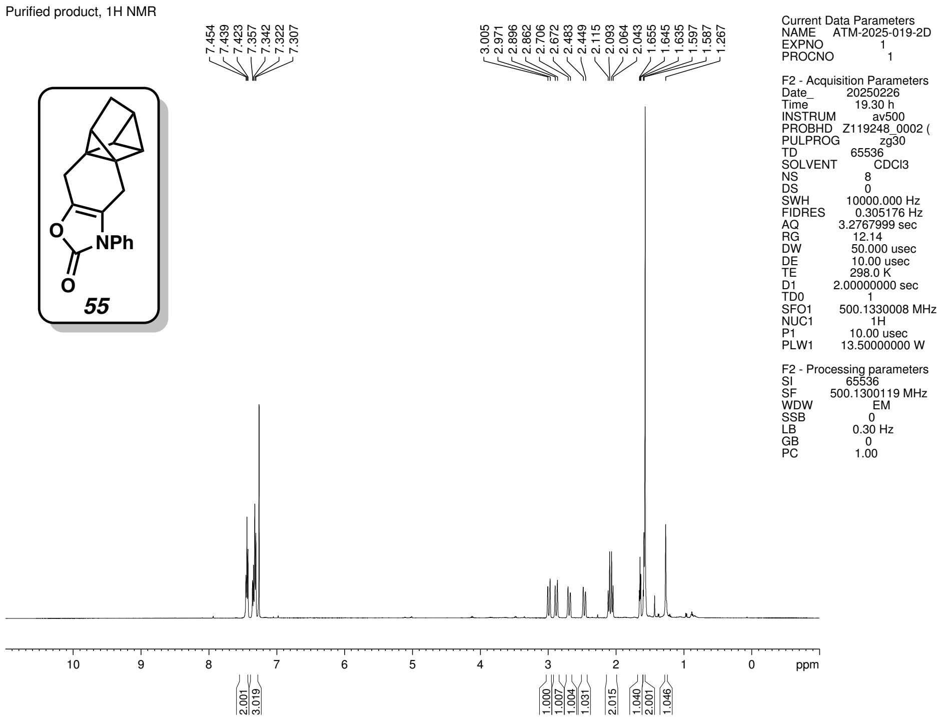

Fig. 78. ^{1}H NMR spectrum of compound **55** in $CDCl_3$ (500 MHz).

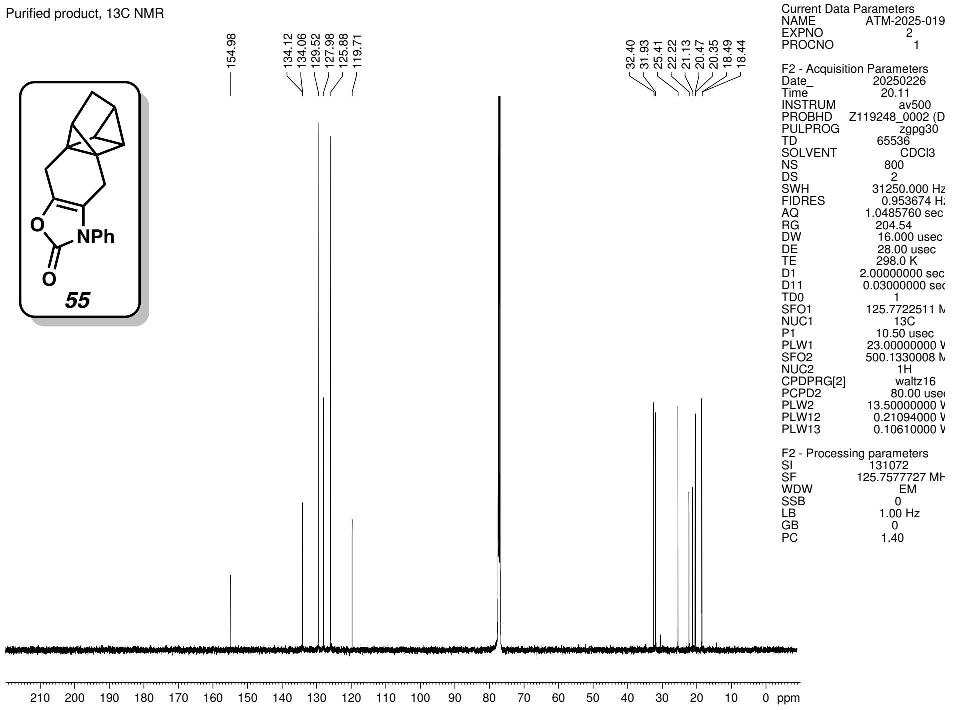

Fig. 79. ^{13}C NMR spectrum of compound **55** in $CDCl_3$ (125 MHz).

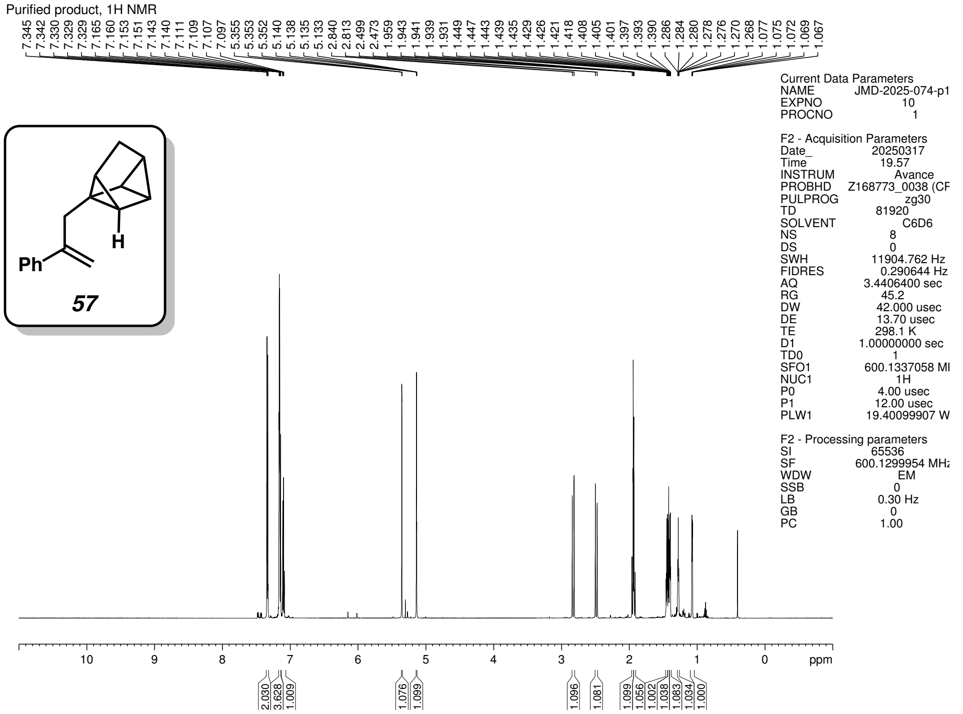

Fig. 80. ^{1}H NMR spectrum of compound **57** in C_6D_6 (600 MHz).

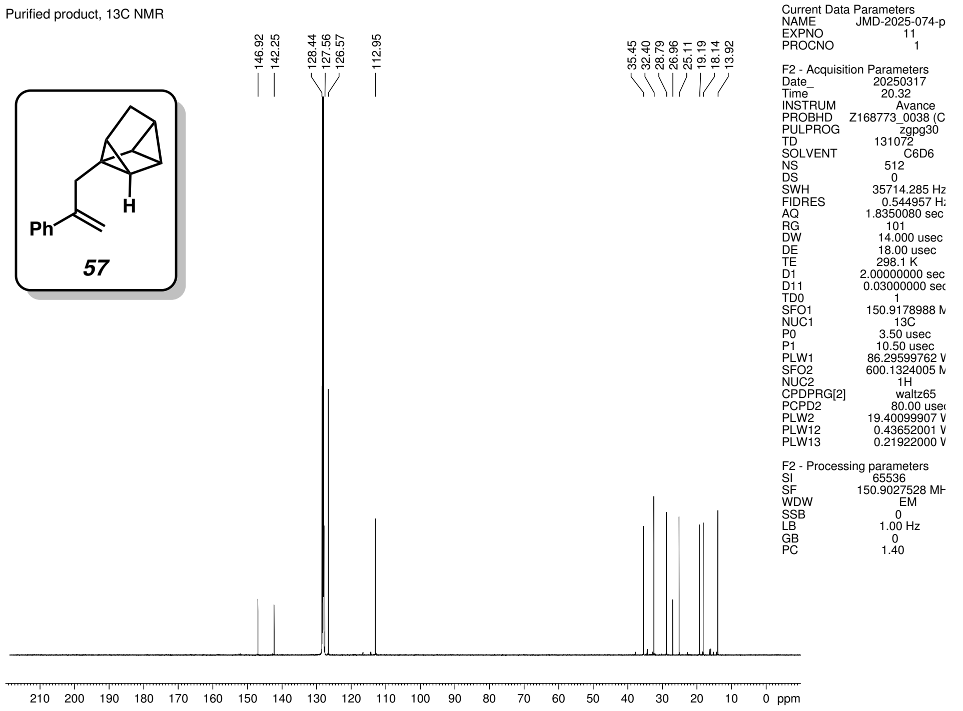

Fig. 81. ^{13}C NMR spectrum of compound **57** in C_6D_6 (150 MHz).

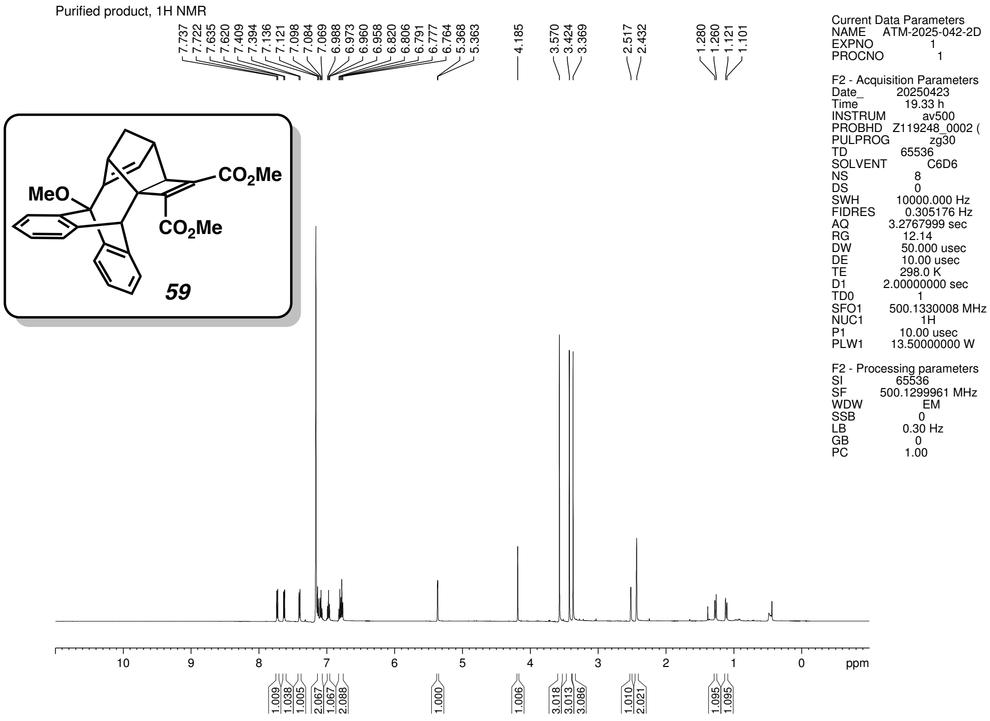

Fig. 82. ^{1}H NMR spectrum of compound **59** in C_6D_6 (500 MHz).

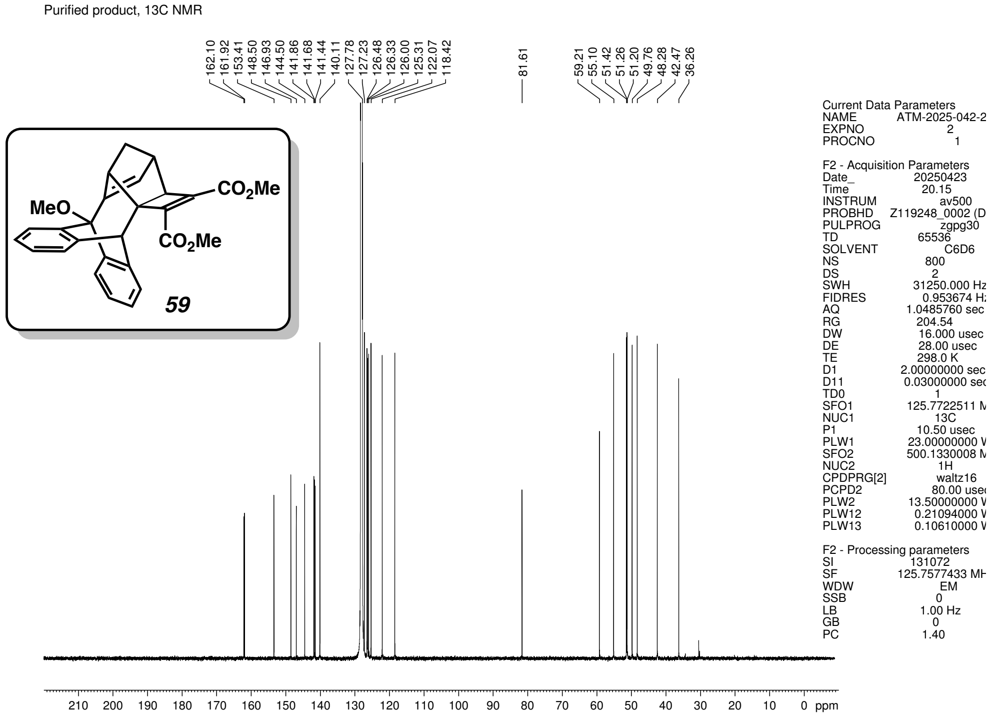

Fig. 83. ^{13}C NMR spectrum of compound **59** in C_6D_6 (125 MHz).

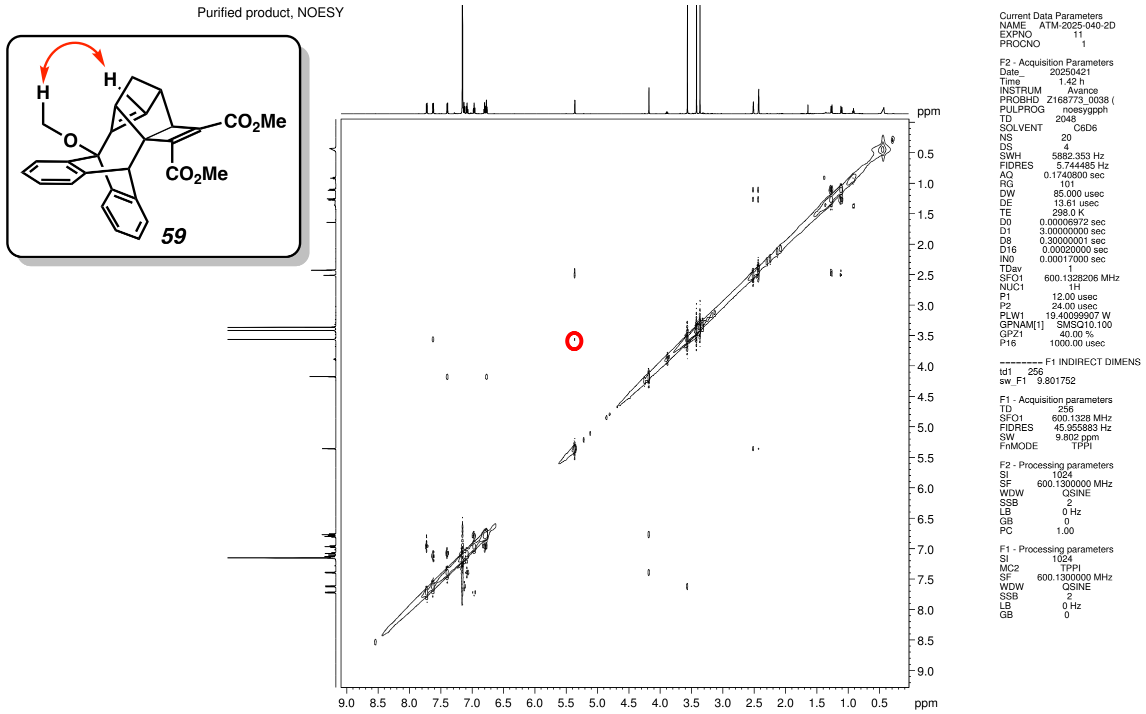

Fig. 84. NOESY spectrum of compound **59** in C_6D_6 (600 MHz).

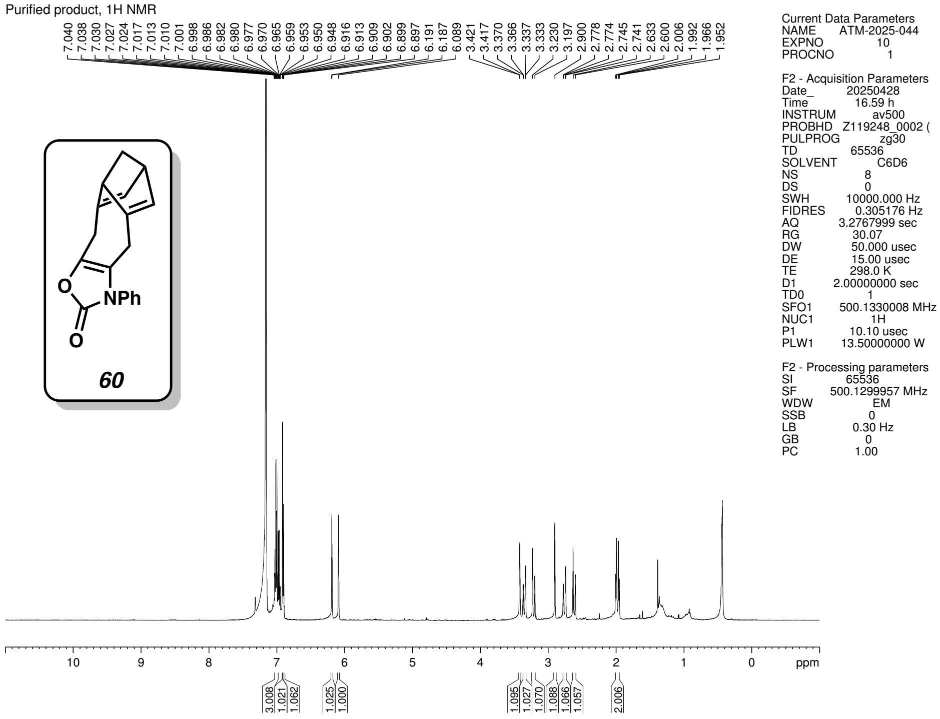

Fig. 85. ^{1}H NMR spectrum of compound **60** in C_6D_6 (500 MHz).

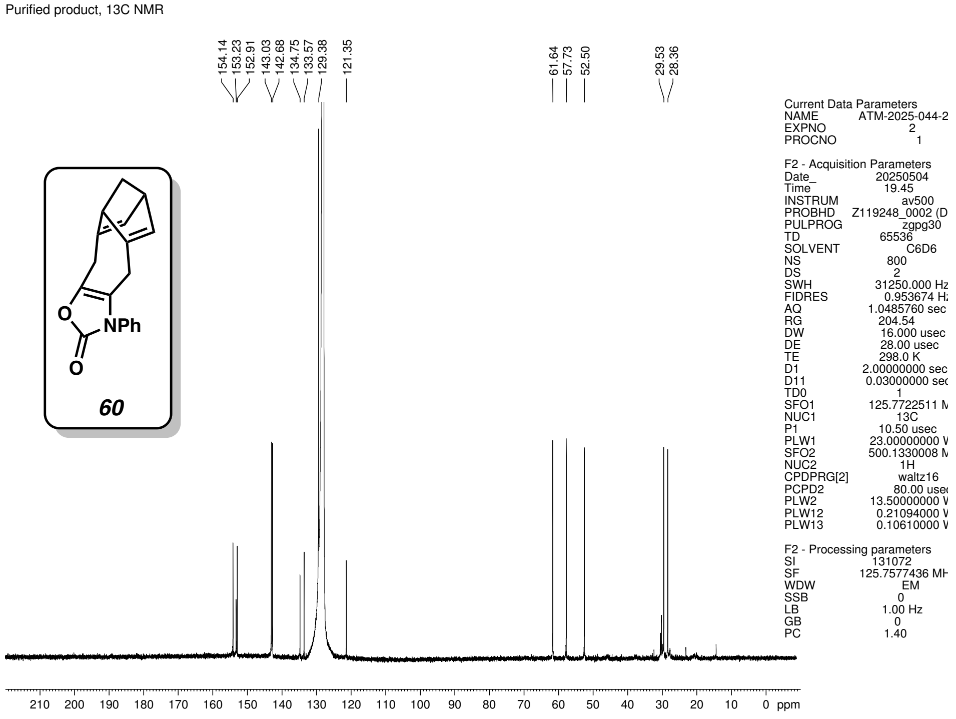

Fig. 86. ^{13}C NMR spectrum of compound **60** in C_6D_6 (125 MHz).

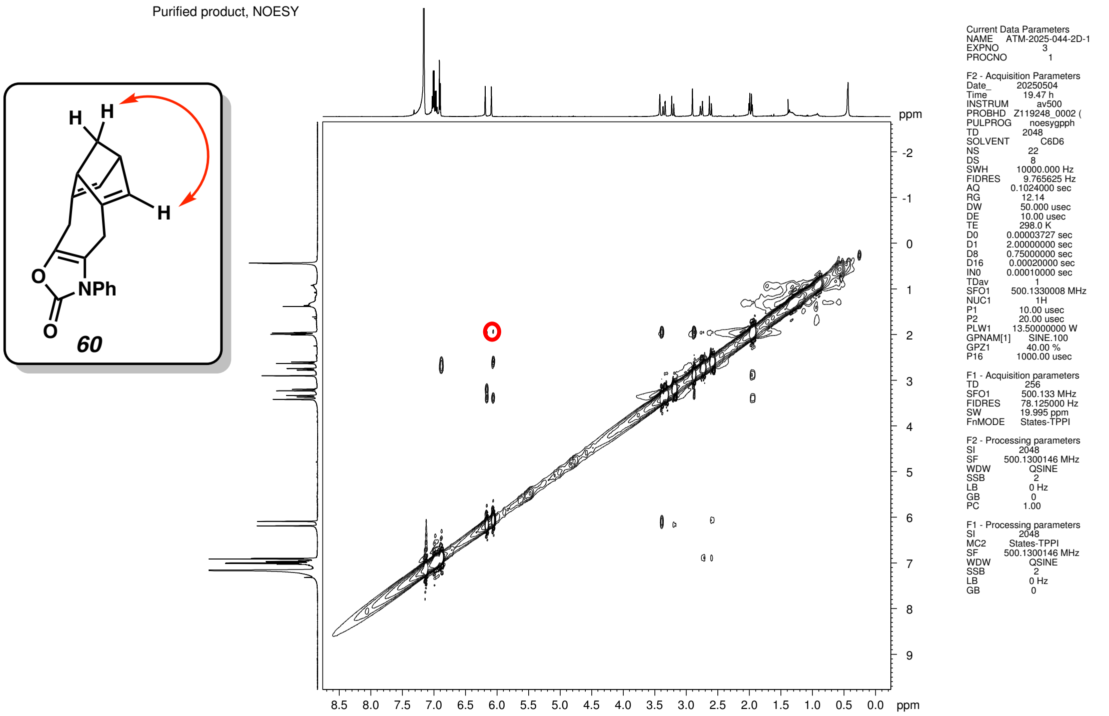

Fig. 87. NOESY spectrum of compound **60** in C_6D_6 (500 MHz).

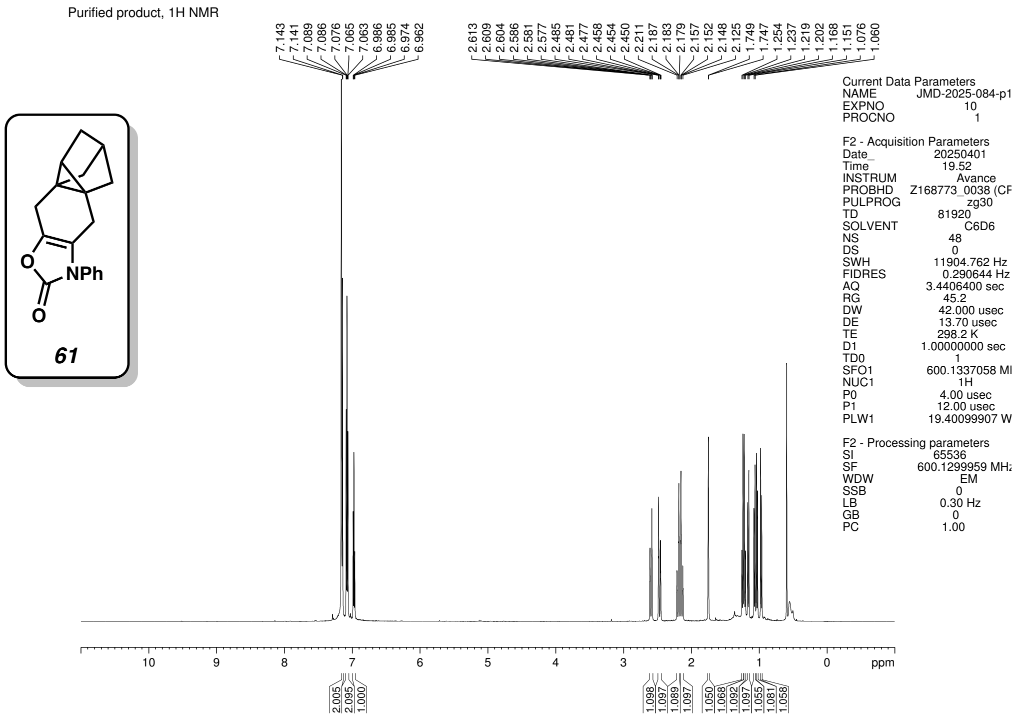

Fig. 88. ^{1}H NMR spectrum of compound **61** in C_6D_6 (600 MHz).

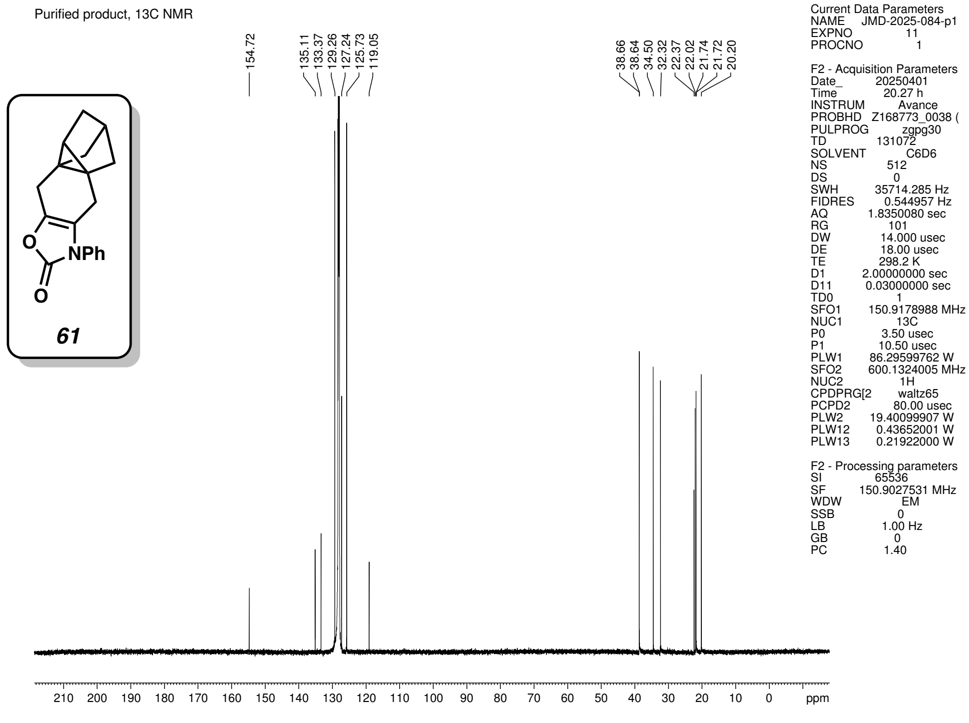

Fig. 89. ^{13}C NMR spectrum of compound **61** in C_6D_6 (150 MHz).

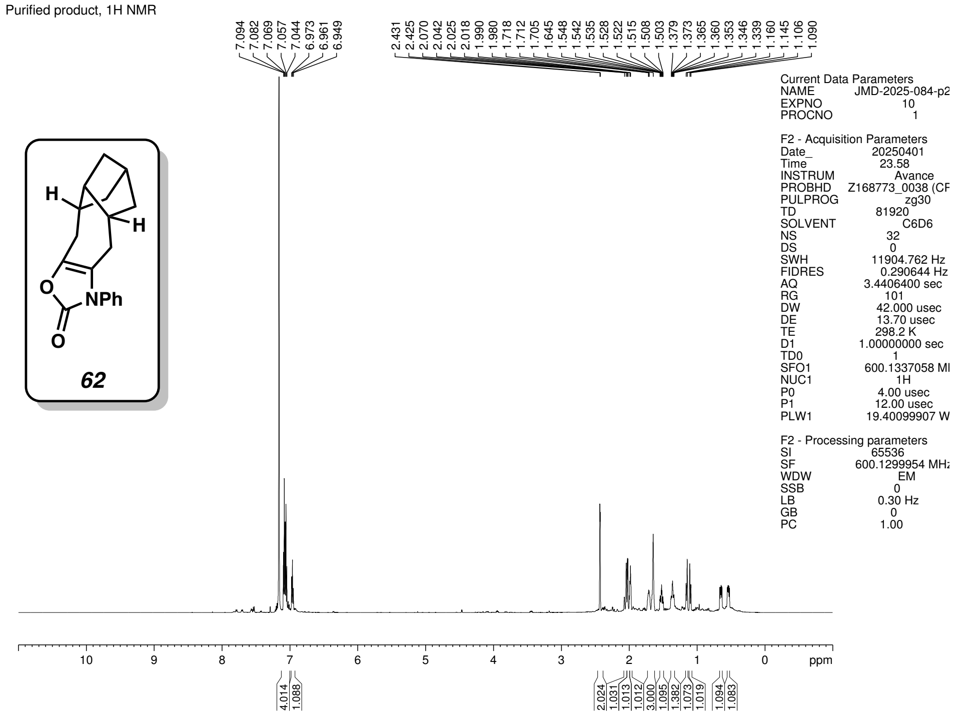

Fig. 90. ^{1}H NMR spectrum of compound **62** in C_6D_6 (600 MHz).

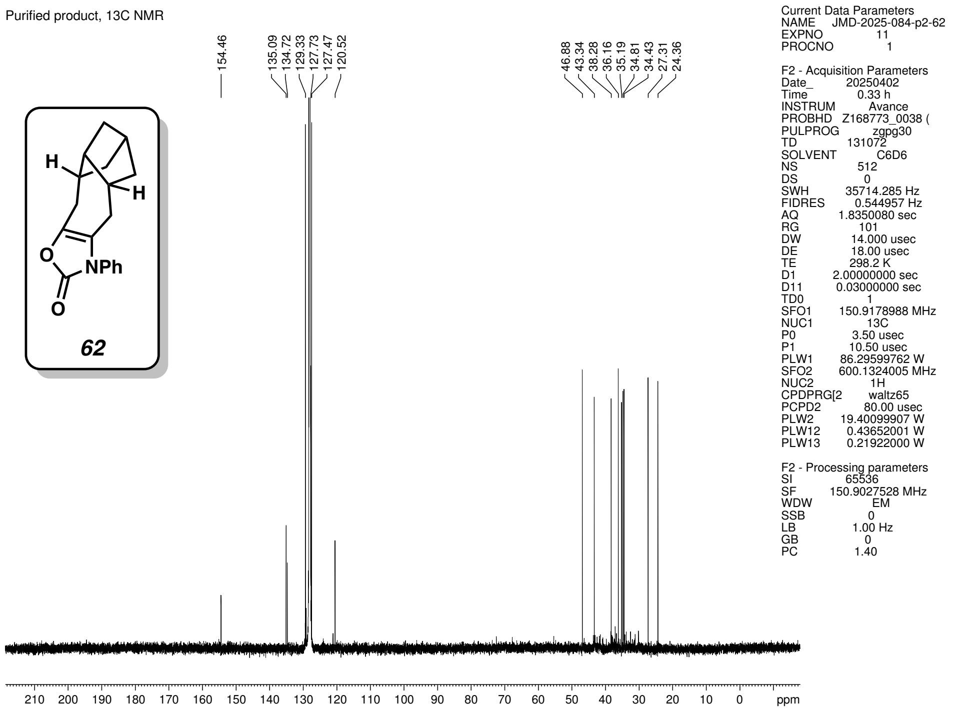

Fig. 91. ^{13}C NMR spectrum of compound **62** in C_6D_6 (150 MHz).

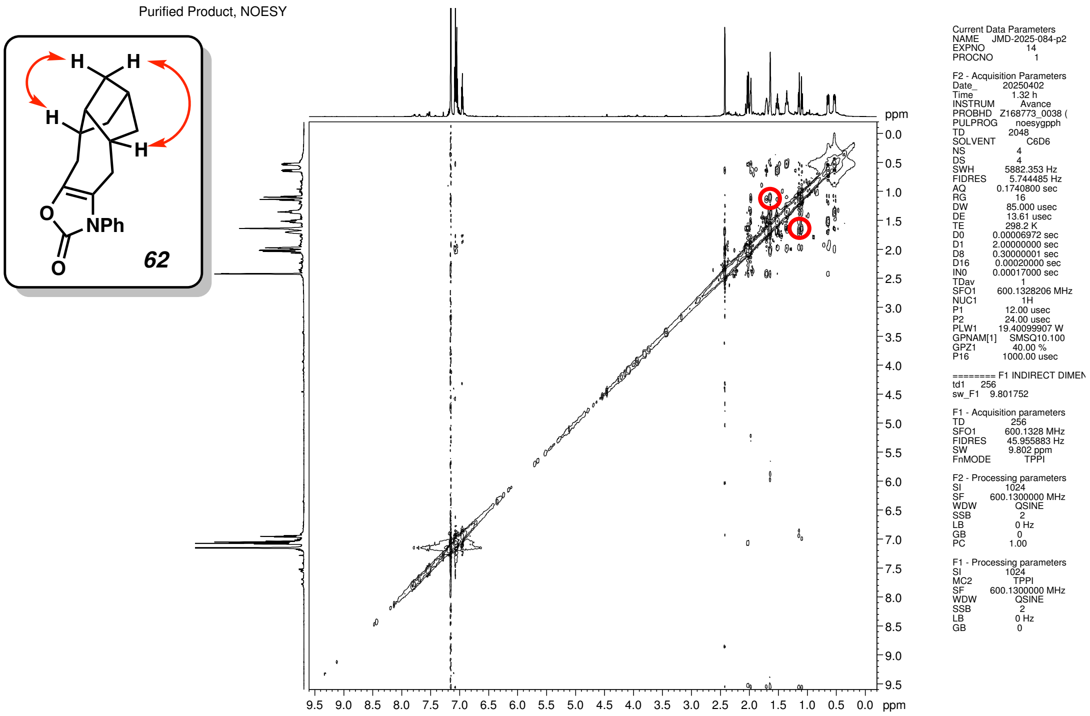

Fig. 92. NOESY spectrum of compound **62** in C_6D_6 (600 MHz).

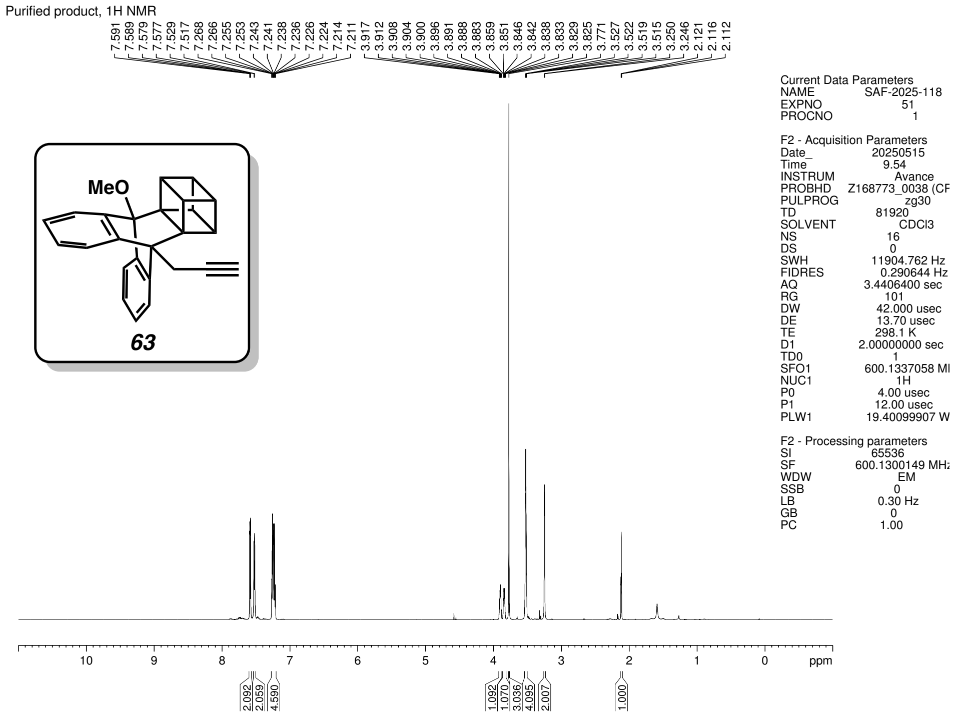

Fig. 93. ^{1}H NMR spectrum of compound **63** in $CDCl_3$ (600 MHz).

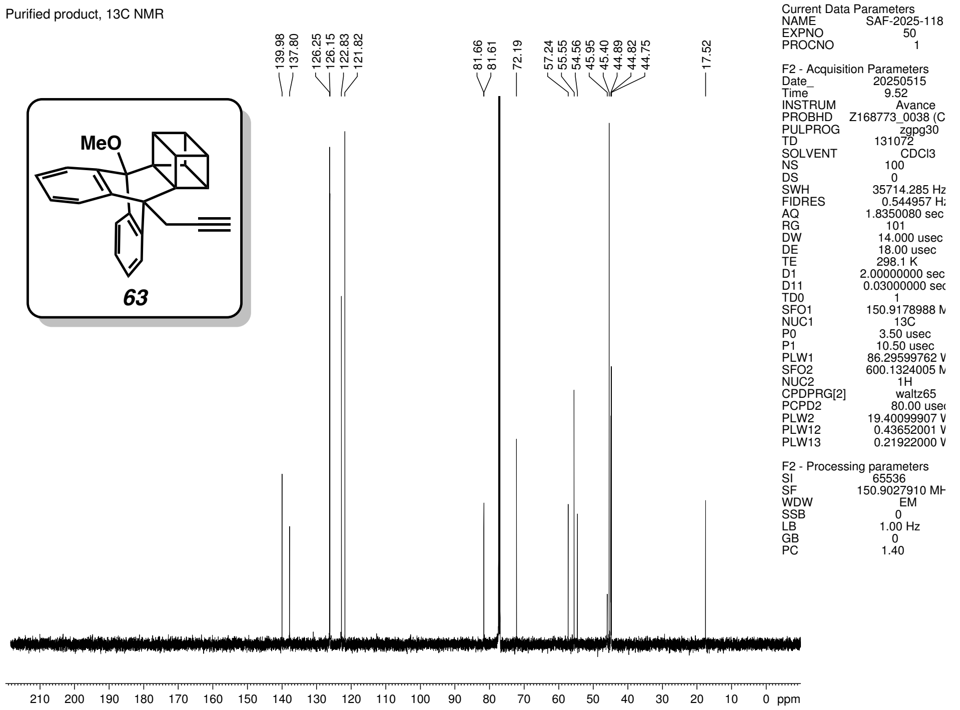

Fig. 94. ^{13}C NMR spectrum of compound **63** in $CDCl_3$ (150 MHz).

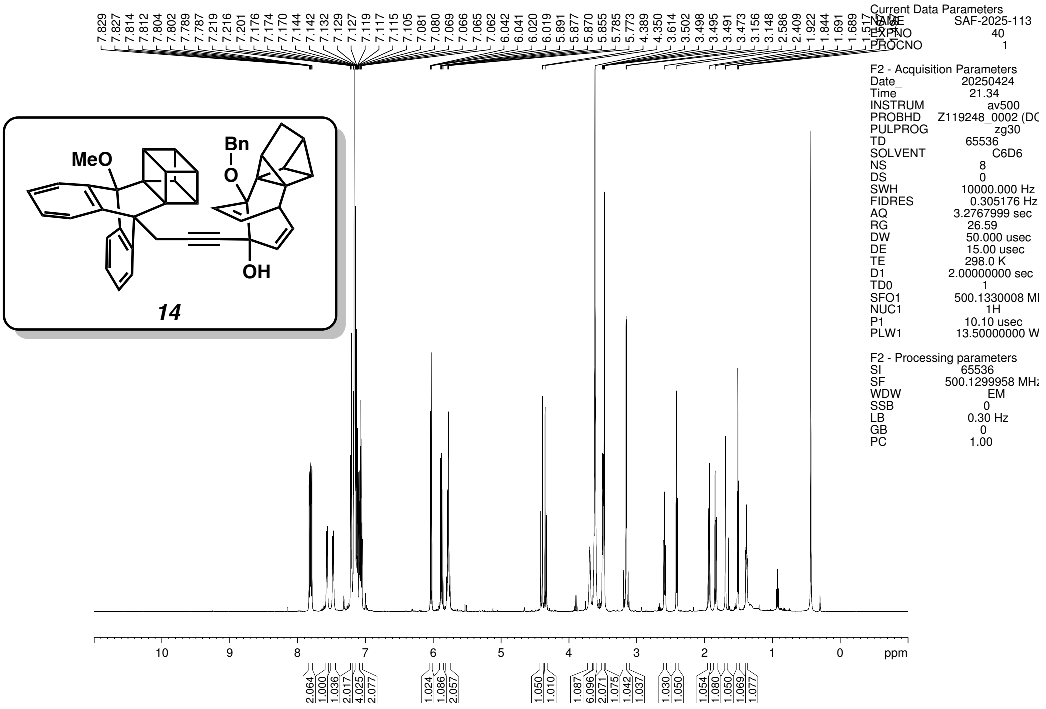

Fig. 95. ^{1}H NMR spectrum of compound **14** in C_6D_6 (500 MHz).

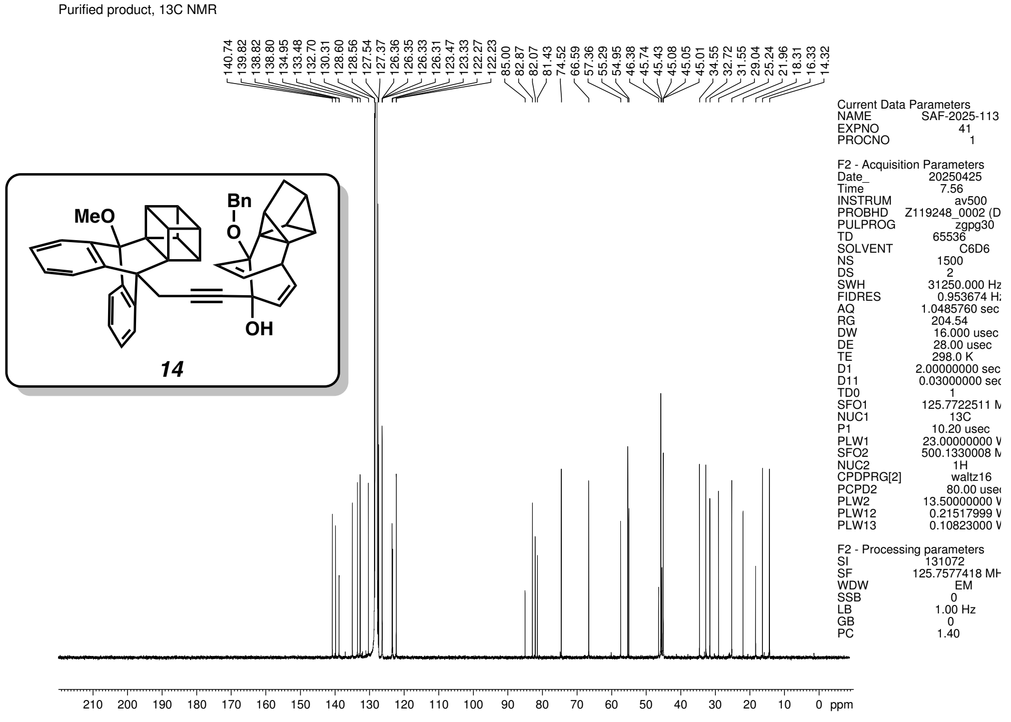

Fig. 96. ^{13}C NMR spectrum of compound **14** in C_6D_6 (125 MHz).

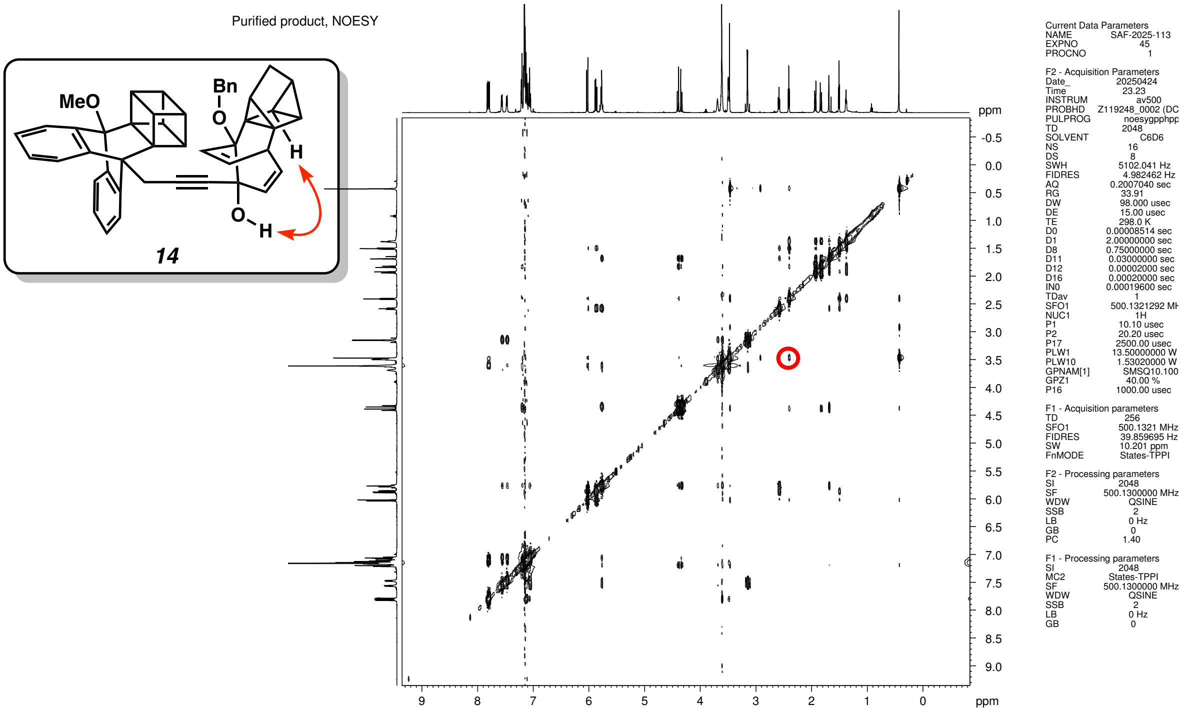

Fig. 97. NOESY spectrum of compound **14** in C_6D_6 (500 MHz).

Part II: Computational Section

A. Computational Methods

Initial structures were prepared in Spartan '24 Version 1.1.1[20] and optimized using Molecular Mechanics. All DFT calculations were performed using Gaussian 16.[21] All computations using multireference Complete Active Space Self–Consistent field (CASSCF)[22,23] and second order perturbation (CASPT2)[24,25] methods were performed using ORCA 6.0.1.[26] Unless otherwise specified, all geometry optimizations were performed using the ωB97X-D[27] functional with the def2-TZVP[28] basis set. GoodVibes (v.3.2)[29] with quasi-harmonic entropy[30] and enthalpy[31] treatment (frequency cut-off value: 100 cm^{-1}) was used to obtain corrected Gibbs free energies and enthalpies at 298.15 K and 1 atm. The molecular orbital structures and energies were obtained by single point energy calculations at the HF/6-31G(d,p) level of theory. Olefin strain energies were obtained by single point energy calculations at the CCSD(T)[32]/cc-pVTZ[33] level of theory. Stability checks of the restricted wavefunction were performed to ensure that open-shell wavefunctions are not more stable. Unless otherwise specified, all geometries were verified as stationary points on the potential energy surface and characterized as local minima by frequency calculations. All Mayer bond order (MBO) analysis[34] was performed using Gaussian 16 by using the *pop=(nboread,always)* keyword and adding *$nbo bndidx mulorb $end* at the end of Gaussian input file using NBO 3.1 module[35] available on Gaussian16, or using Multiwfn v3.8[36,37] from the *.fchk* file generated from Gaussian16.[21] MBOs obtained from either software were identical for the compounds included in this study.

B. Choice of Computation Method and Benchmark Studies

In order to study the ground state geometries of **10** and **11**, a variety of methods (DFT, HF, MP2, and CASSCF) used in prior studies on bent and pyramidalized alkenes[38] were evaluated. Pyramidalization angles were calculated following POAV1 method[39] utilizing the spreadsheet available at http://carbonsolution.com. For cubene (C_{2V} symmetry), the pyramidalization angles as defined by Borden ($\Phi_{p(\text{Borden})}$)[40] are also evaluated and listed alongside. In the case of both cubene (**10**) and 1,7-quadricyclene (**11**) (Tables 3 and 4, respectively), all geometries optimized with DFT methods show reasonably good agreement with each other and with those geometries optimized with configuration interaction method at the CAS(4,4) level. The geometries obtained from MP2 and Hartree–Fock methods display greater deviations. ωB97X-D/def2-TZVP was chosen as our

primary method for geometry optimizations and transition state analysis since it has shown good agreement with higher level CASSCF methods and has been proven effective for the modeling of other distorted alkenes.[41] Our results are shown in Tables 3 and 4 below.

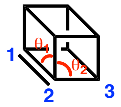

10

Level of Theory	Length (C1=C2) (Å)	θ1 (°)[2]	θ2 (°)	Pyramidalization (Φ_p) (°)	Pyramidalization ($\Phi_{p(Borden)}$) (°)
ωB97X-D/def2-TZVP	1.38	93.1	98.9	31.6	85.2
M06-2X/def2-TZVP	1.38	93.0	99.4	31.5	85.4
B3LYP/6-31G(d,p)	1.39	93.1	98.7	31.6	85.2
CASSCF(4,4)/def2-TZVP[1]	1.39	93.1	97.1	32.0	85.3
HF/6-31G(d,p)	1.36	93.5	98.5	31.5	84.6
UHF/6-31G(d,p)	1.46	91.9	94.8	33.2	87.2
MP2/6-31G(d,p)	1.42	92.5	99.3	31.8	86.1
TCSCF/3-21G (ref. 42)	1.42	93.0	95.2	32.6	85.5

[1] Frequency calculation not performed
[2] Due to symmetry, the third bond angle (∠C1–C2–C3) is identical to θ1

Table 3. Computed key geometrical properties of cubene (**10**) at differing level of theory.

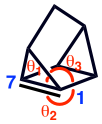

11

Level of Theory	Length (C1=C7) (Å)	θ1 (°)	θ2 (°)	θ3 (°)	Pyramidalization (Φ_p) (°)
ωB97X-D/def2-TZVP	1.35	63.4	92.7	111.7	33.2
M06-2X/def2-TZVP	1.35	63.3	92.7	111.8	33.1
B3LYP/6-31G(d,p)	1.36	63.3	92.6	111.4	33.3
CASSCF(4,4)/def2-TZVP[1]	1.36	63.2	92.5	110.5	33.7
HF/6-31G(d,p)	1.33	63.6	92.9	111.8	33.1
UHF/6-31G(d,p)	1.41	61.9	91.6	108.4	34.7
MP2/6-31G(d,p)	1.39	62.6	92.0	111.1	33.6
TCSCF/6-31G(d,p) (ref 43)	1.37	62.8	92.3	110.4	33.8

[1] Frequency calculation not performed

Table 4. Computed key geometrical properties and of 1,7-quadricyclene (**11**) at differing level of theory.

Next, benchmark studies were carried out determining the Mayer bond order (MBO) analysis of a variety of unsaturated compounds with non-integer bond orders. *trans*-Cyclooctene (**6**) *cis*-cyclooctene (*cis*-**6**), s-*trans*-1,3-butadiene (**8**) and benzene (**9**) were studied herein. ωB97X-D/def2-TZVP was used for geometry optimizations and MBO analysis. As shown in Fig. 98, the MBOs of C=C bonds in 1,3-butadiene (**8**) and benzene (**9**) are non-integer (1.41 and 1.87, respectively), as the expected consequence of aromaticity/resonance. Moreover, we observed a decreased alkene bond order in *trans*-cyclooctene (**6**, MBO = 1.91) compare to that of the *cis*-isomer (*cis*-**6**, MBO = 1.96). This indicates that factors other than conjugation and aromaticity such as geometric distortion can also lead to a decreased alkene bond order.

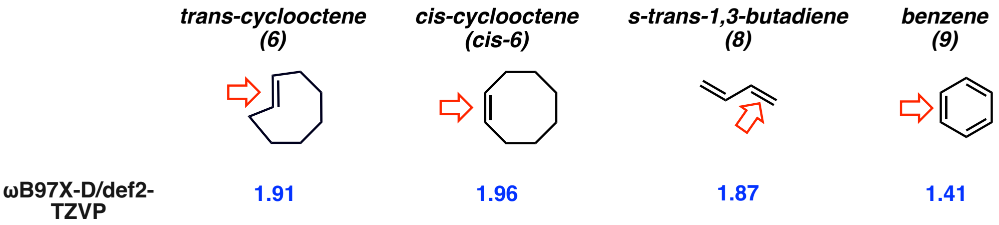

Fig. 98. Mayer bond orders of **6**, *cis*-**6**, **8** and **9**.

Lastly, we sought to probe the functional/basis set dependence of the Mayer bond order analysis.[44] Three different DFT methods were used to obtain the optimized geometry and MBOs of four strained intermediates (i.e., cubene **10**, quadricyclene **11**, [2.2.1] ABO **7**, and benzyne **75**). Of note, diffuse functions were avoided in all Mayer bond order computations, as it is known that the use of basis sets containing diffuse functions in MBO analysis would lead to "unrealistic results".[45] Our results are summarized in Fig. 99 below. While the resulting MBOs of these strained alkenes/alkynes at different level of theory did show some fluctuation, significant decrease of MBOs from ideal values (e.g. ~2 for alkenes) were observed in all cases, with the MBOs of **10** and **11** decreasing to near 1.5. From there, we concluded that Mayer bond order is capable of providing a *qualitative estimation* of the extent to which π bonds are weakened as the result of distortion.

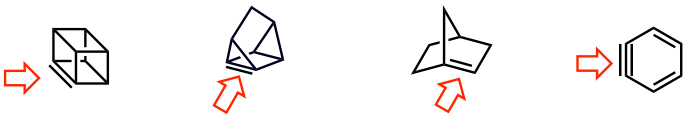

	Cubene (10)	Quadricyclene (11)	[2.2.1] ABO (7)	Benzyne (75)
B3LYP/6-31G(d,p)	1.56	1.56	1.82	2.28
ωB97X-D/def2-TZVP	1.59	1.55	1.76	2.31
M06-2X/def2-TZVP	1.62	1.47	1.75	2.31

Fig. 99. Mayer bond order analysis of strained intermediates **10**, **11**, **7**, and **75** using differing DFT methods.

C. Olefin Strain Energies of Cubene (10) and 1,7-Quadricyclene (11)

Homodesmotic[46] equations were used to estimate the total strain energies in cubene (**10**), cubane (**76**), 1,7-quadricyclene (**11**) and quadricyclane (**77**). Energies of **10**, **11** and each reference compounds were calculated at the CCSD(T)/cc-pVTZ//ωB97X-D/def2-TZVP level of theory. The energy of an aliphatic –CH_2– group was computed by the difference in energy between *n*-pentane and *n*-butane. As described by Maier and Schleyer,[47] Olefin Strain Energies (OSEs) of cubene (**10**) and 1,7-quadricyclene (**11**) were derived by subtracting the total strain energies of each saturated hydrocarbons (cubane (**76**) and quadricyclane (**77**), respectively) from the total strain energies of strained alkenes (cubene (**10**) and 1,7-quadricyclene (**11**), respectively). Detailed computation results are summarized in Figs 100 and 101 as follows.

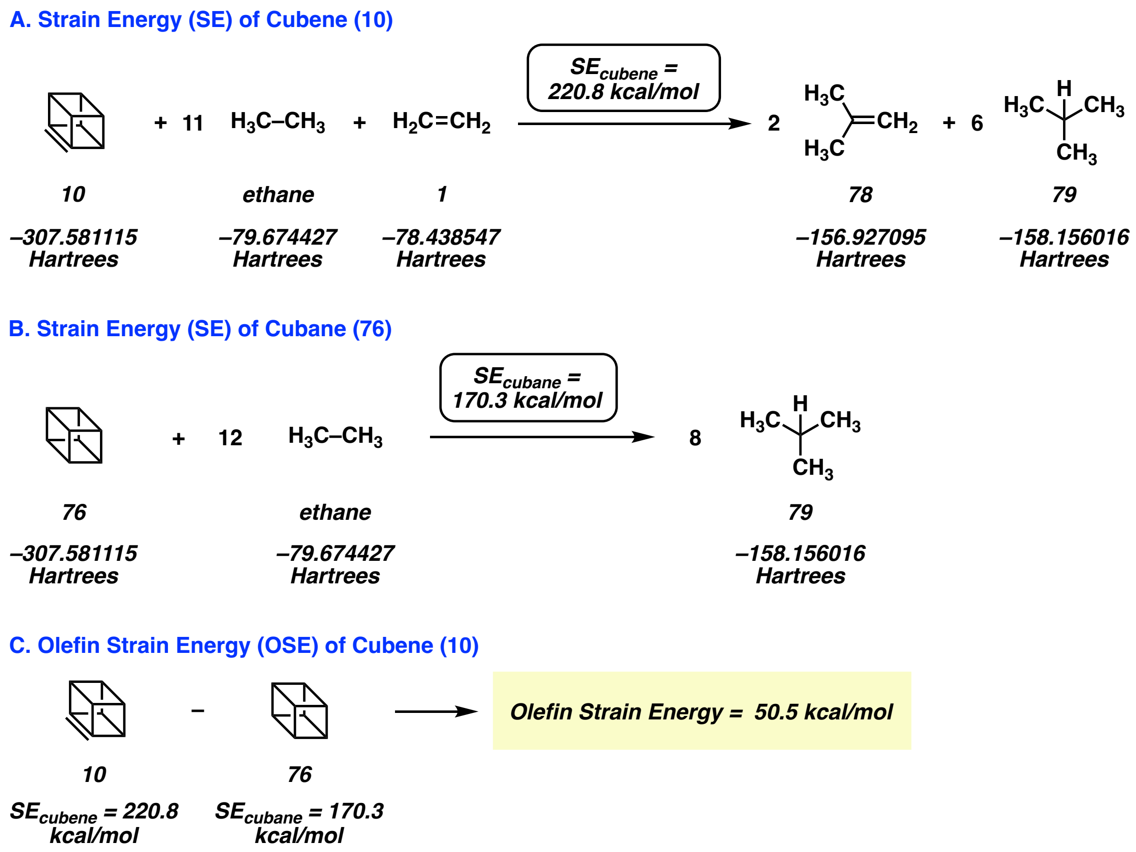

Fig. 100. Olefin strain energy of cubene (**10**) calculated using homodesmotic equations.

The olefin strain energy of cubene (**10**) obtained herein was within reasonable agreement, albeit slightly lower than the value (58.9 kcal/mol) reported by Borden and co-workers,[48] and the value (63 ± 4 kcal/mol) reported by Kass and co-workers.[49]

Energy of CH_2

$E(CH_2) = E(n\text{-pentane}) - E(n\text{-butane}) = (-197.393425) - (-158.153732) = -39.239693$ Hartrees

---

Strain Energy of 1,7-Quadricyclene (11)

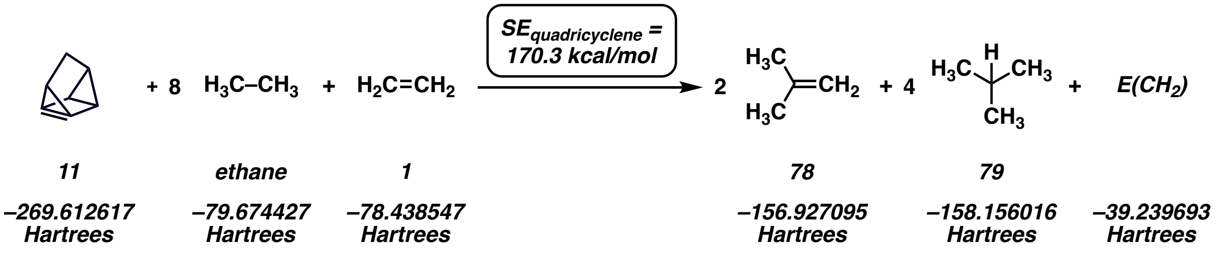

Strain Energy of Quadricyclane (77)

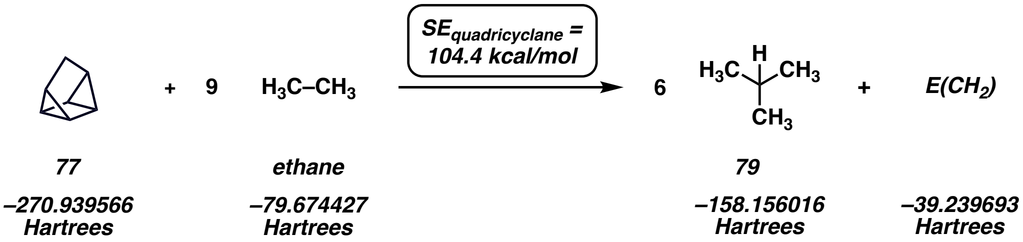

Olefin Strain Energy (OSE) of 1,7-Quadricyclene (11)

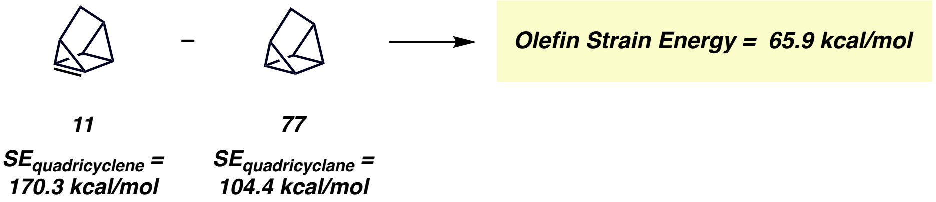

Fig. 101. Olefin strain energy of 1,7-quadricyclene (**11**) calculated using homodesmotic equations.

The olefin strain energy of 1,7-quadricyclene (**11**) obtained is in good agreement with the value (67 kcal/mol) reported by Szeimies and coworkers.[43]

D. Diradical Character of Ethylene (1), Cubene (10) and 1,7-Quadricyclene (11)

In order to evaluate the diradical character of ethylene (**1**), cubene (**10**) and 1,7-quadricyclene (**11**), single point energy computations were performed using complete active space self–consistent field (CASSCF) calculations. These were conducted at the CASPT2/CASSCF(6,6)/aug-cc-pVDZ level of theory, utilizing geometries optimized at ωB97X-D/def2-TZVP level of theory. The computed

occupation numbers of the lowest unoccupied natural orbitals (n_{LUNO}) from CASSCF calculations represent the diradical character (y_0) values.[50] Variations of basis sets (def2-TZVP versus aug-cc-pVDZ) led to negligible fluctuations in the resulting diradical character. To probe the consistency of our chosen method comparing to the prior works, two previously studied diradicaloids, i.e., cyclic allene **80**[51] and [2.2.1] anti-Bredt olefin **7**[41] (reported y_0 = 14% and 17%, respectively) were tested as benchmarks. Our results are summarized in Figs. 102 and 103 as follows. Orbitals in the active space are visualized using Avogadro 1.2.0[52] with isovalue of ±0.05 au.

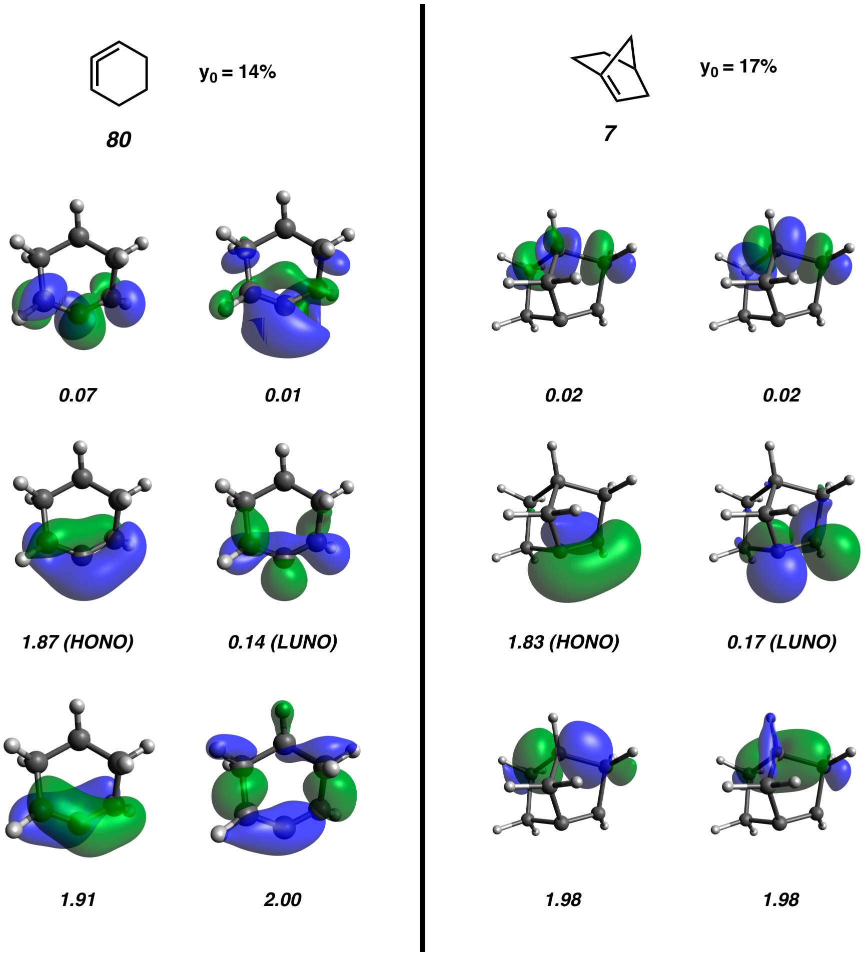

Fig. 102. Benchmark studies on diradical character (y_0) of cyclic allene **80** and ABO **7**.

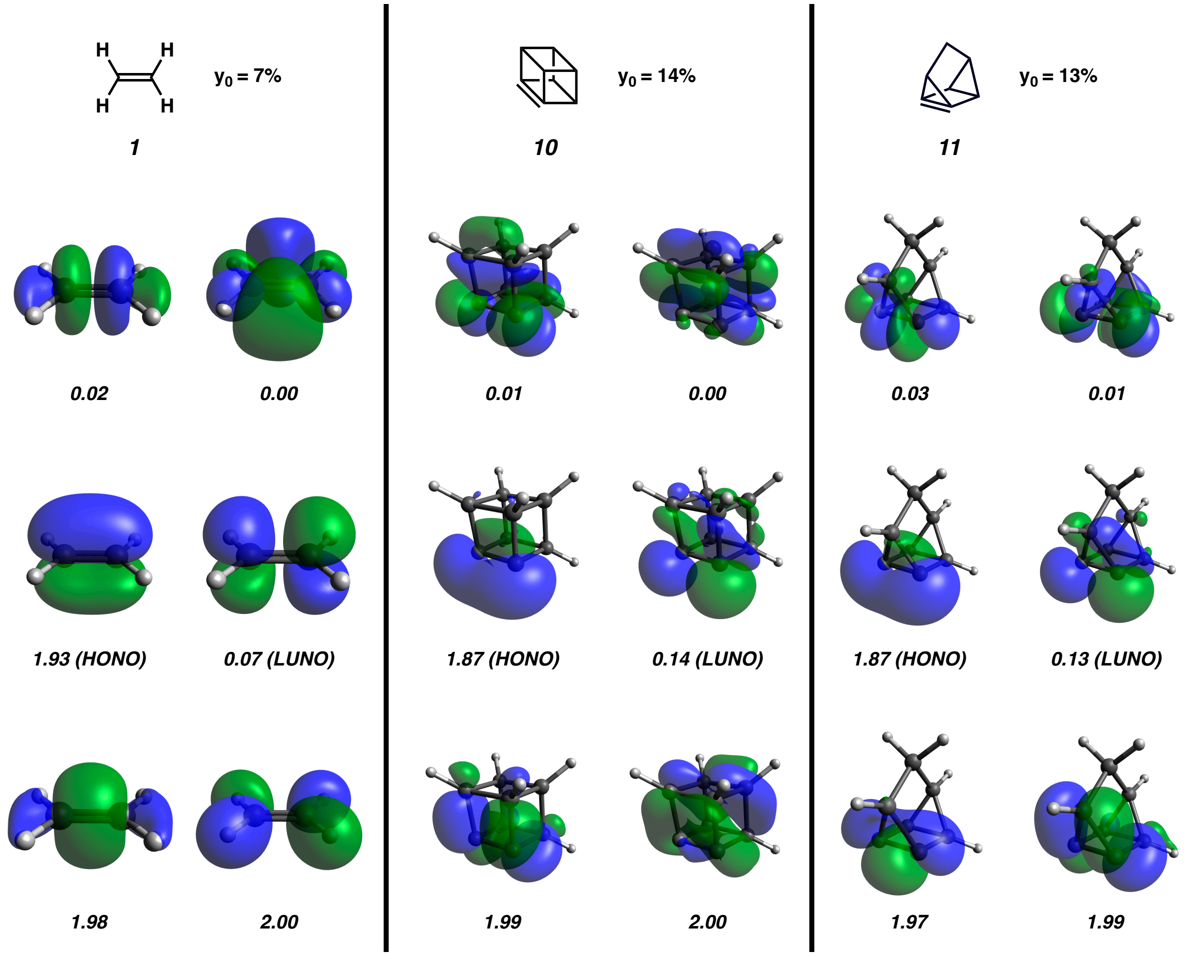

Fig. 103. Computed diradical character (y_0) and CAS(6,6) active space specification of ethylene (**1**), cubene (**10**) and 1,7-quadricyclene (**11**).

The results suggest that our choice of computational method is consistent with those used in the prior studies,[41,51] and the change of basis sets does not significantly alter the resulting computed diradical characters. Our results show that hyperpyramidalized cubene (**10**) and 1,7-quadricyclene (**11**) have notably greater diradical character (14% and 13%, respectively) compared to a stable, non-pyramidalized alkene (ethylene (**1**), y_0 = 7%), rendering them diradicaloids. They would presumably behave largely like closed-shell species, but could potentially display some diradical–like reactivities. Notably, our computed diradical character of **11** was also in reasonable agreement with the lower reported value (9%)[43,53] obtained using the configuration interaction method (TCSCF/6-31G(d,p)) available at the time.

E. Orbital Occupancy-Perturbed Mayer Bond Order Analysis of 1c, 1d and 1e

In order to evaluate the contributions of occupied σ- and π MOs towards the total alkene bonding, and to probe how the relative weight of σ- and π-bonding change upon pyramidalization (**1c**→**1d**→**1e**), orbital occupancy-perturbed Mayer bond order (OOP–MBO)[54] analysis is carried out on **1c**, **1d** and **1e** based on their localized molecular orbitals (LMOs). All calculations in this section were carried out using wavefunctions obtained at the HF/6-31G(d,p) level of theory. Transformation of canonical molecular orbitals (CMOs) to LMOs were carried out using Multiwfn v3.8[36,37] with Pipek–Mezey localization method using Becke charges.[55] Structures of LMOs are visualized using an in-house 3D rendering tool, with isovalue of the surfaces set at ±0.05 au. The corresponding LMO plots are shown in Fig. 104.

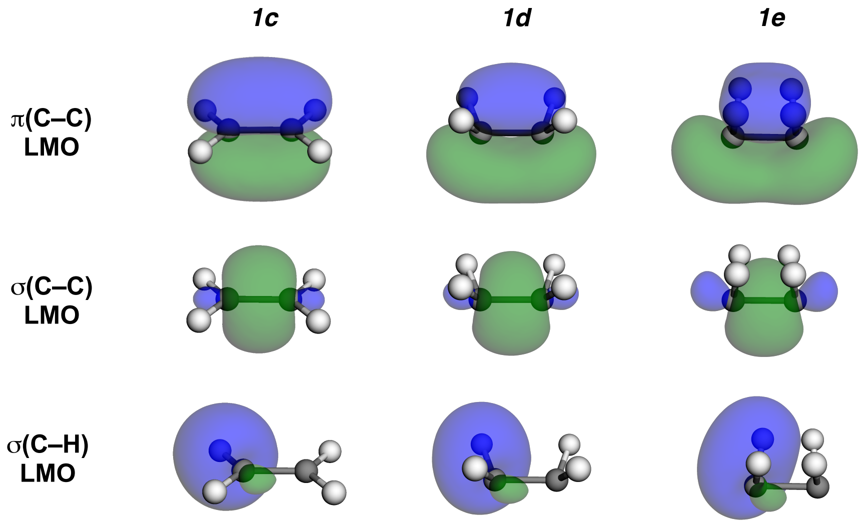

Fig. 104. LMOs of **1c**, **1d** and **1e**. Only one of the four σ(C–H) LMOs is shown.

The results of OOP–MBO analysis are summarized in Table 5. In planer ethylene **1c,** the omission of occupancy in filled σ or π LMOs led to decreases of MBO by 1.07 or 0.97, respectively. This indicates the contributions of σ and π bonds to the total alkene bond order in **1c** are roughly equal and near one. This is expected for a planar, undistorted alkene. Upon pyramidalization to distorted alkene **1d**, a smaller decrease of perturbed MBO (–0.94, shown in blue) was observed as π MO occupancy is removed. This indicates a smaller overall contribution from the π-bond compared to that from the σ-bond (–1.09) to the overall alkene bonding. Further hyperpyramidalization in alkene **1e** leads to a greater decrease in the π-bonding contribution (–0.74, shown in blue), whereas

the contribution from the C-C σ-bond to the alkene bond remained roughly the same (–1.12).

From these results shown herein, we conclude that the lowering of alkene bond order in pyramidalized/hyperpyramidalized alkenes is attributed to the weakening of π-bonds rather than σ-bonds.

	**1c (MBO$_{C=C}$)**	**1d (MBO$_{C=C}$)**	**1e(MBO$_{C=C}$)**
**MBO without occupancy-perturbation**	1.96	1.87	1.47
**MBO after σ(C–H) LMO occupancy-perturbation**	1.98 (+0.02)	1.91 (+0.04)	1.53 (+0.07)
**MBO after σ(C–C) LMO occupancy-perturbation**	0.89 (–1.07)	0.78 (–1.09)	0.35 (–1.12)
**MBO after π(C–C) LMO occupancy-perturbation**	0.97 (–0.99)	0.93 (–0.94)	0.73 (–0.74)

Table 5. OOP–MBOs of **1c**, **1d**, and **1e**. Differences of alkene MBOs after occupancy-perturbation are shown in parentheses.

F. Frontier Molecular Orbitals of Cubene (10), and 1,7-Quadricyclene (11)

The structures and energies of Frontier Molecular Orbitals (FMOs) of cubene (**10**), 1,7-quadricyclene (**11**), and ethylene (**1**) were calculated at the HF/6-31G(d,p)//ωB97X-D/def2-TZVP level of theory.

FMO surfaces (Figs. 105 and 106) were generated by exporting the *.cub* file from the *.chk* file using GaussView 6.0.16,[56] and visualization of MO surfaces was carried out using an in-house 3D rendering tool. Fine grids were used for all *.cub* files. The isovalue of the MO surfaces shown in Figs. 105 and 106 are set at ±0.05 au.

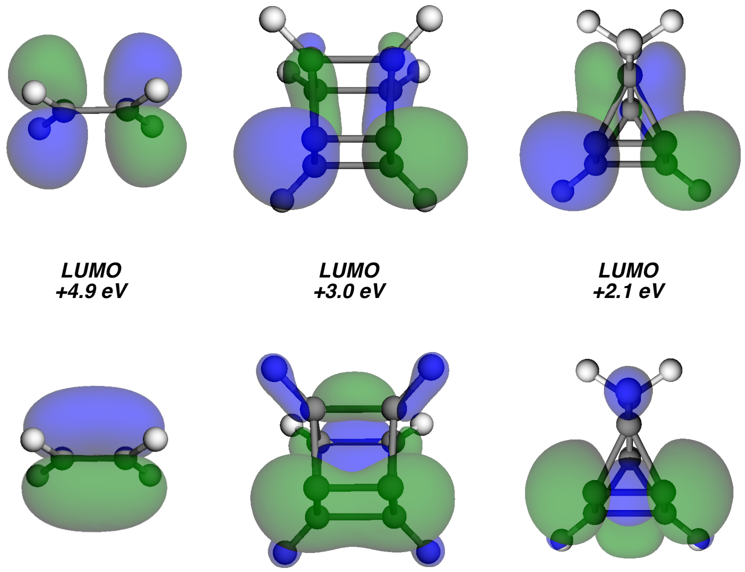

Fig. 105. FMO structures (front view) and energies of cubene (**10**) and 1,7-quadricyclene (**11**). The corresponding molecular orbitals of ethylene (**1**) are shown for comparison.

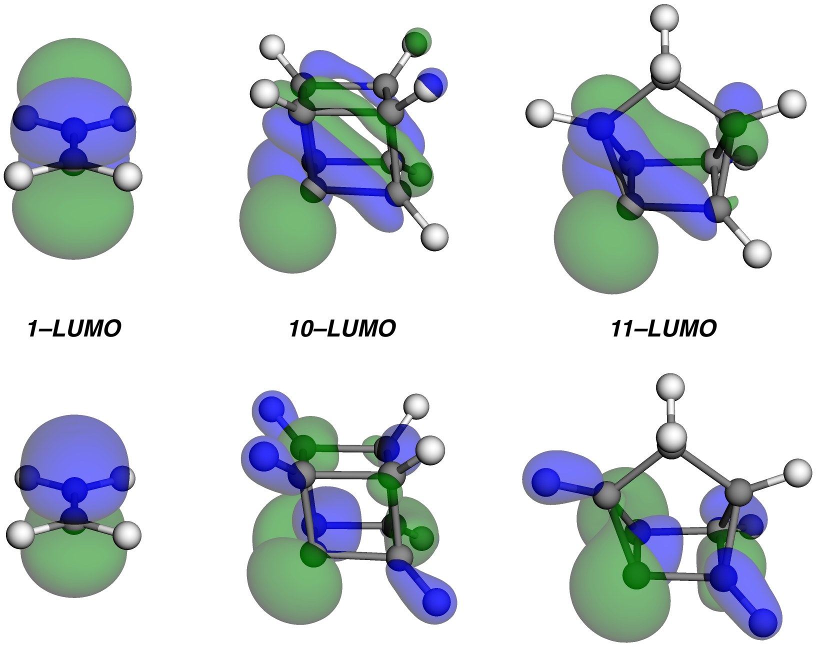

Fig. 106. FMO structures (side view) of cubene (**10**) and 1,7-quadricyclene (**11**). The corresponding molecular orbitals of ethylene (**1**) are shown for comparison.

G. Orbital Composition Analysis of 1c, 1d and 1e

To evaluate the relative contributions of each atomic orbital (AO) pair to molecular orbitals (MOs), and how the hybridization may change upon progressive alkene pyramidalization, orbital composition analysis[57] was carried out on structures **1c**, **1d**, and **1e** (Tables 6, 7, and 8, respectively). MO structures and energies for structures **1c**, **1d**, and **1e** were calculated at HF/6-31G(d,p) level of theory. AO composition analysis was carried out using Multiwfn v3.8[36,37] with Mulliken and natural atomic orbital (NAO) approaches. For AO composition analysis using the Mulliken approach,[57] basis-functions on both shells of the split-valence double-zeta basis set corresponding to a certain atomic orbital on both alkene carbons were used in the AO composition analysis. For example, basis functions 2s and 3s on both carbons (four basis functions in total) were selected to represent the composition of 2s atomic orbitals. For AO composition analysis using the NAO approach,[57] the MO coefficient matrices on NAO basis were generated by including keyword *pop=nboread* in the route line and the keyword *$nbo NAOMO $end* at the end of Gaussian input file using NBO3.1 module[58] available on Gaussian16.

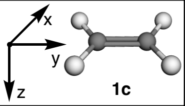 1c	Carbon AOs				Hydrogen AOs
	2s (%) NAO/Mulliken	$2P_x$ (%) NAO/Mulliken	$2P_y$ (%) NAO/Mulliken	$2P_z$ (%) NAO/Mulliken	1s NAO/Mulliken
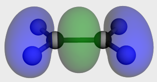 HOMO-2 (–15.6 eV)	0.7/< 0.5	0.0/0.0	74.1/71.7	0.0/0.0	24.5/27.3
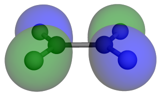 HOMO-1 (–13.7 eV)	0.0/0.0	53.0/45.5	0.0/0.0	0.0/0.0	46.6/52.9
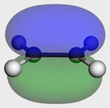 HOMO (–10.2 eV)	0.0/0.0	0.0/0.0	0.0/0.0	99.5/98.7	0.0/0.5
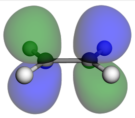 LUMO (5.0 eV)	0.0/0.0	0.0/0.0	0.0/0.0	90.9/98.7	0.1/1.1

Table 6. Orbital visualizations, energies, and AO composition analysis of MOs of **1c**. The AO components of π MO are shown in blue font.

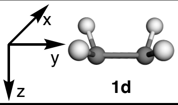 1d	Carbon AOs<br>2s (%)<br>NAO/Mulliken	2P$_x$ (%)<br>NAO/Mulliken	2P$_y$ (%)<br>NAO/Mulliken	2P$_z$ (%)<br>NAO/Mulliken	Hydrogen AO<br>1s<br>NAO/Mulliken
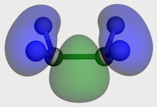 HOMO-2 (–15.5 eV)	0.0/< 0.5	0.0/0.0	54.0/52.5	23.2/21.2	22.2/25.3
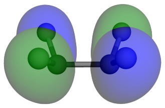 HOMO-1 (–12.6 eV)	0.0/0.0	52.0/44.8	0.0/0.0	0.0/0.0	47.7/53.7
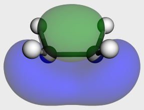 HOMO (–10.2 eV)	7.6/7.4	0.0/0.0	22.9/23.2	67.7/67.2	0.9/1.3
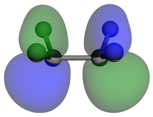 LUMO (2.9 eV)	8.2/6.0	0.0/0.0	10.0/16.0	66.5/68.7	9.3/8.9

Table 7. Orbital visualizations, energies, and AO composition analysis of MOs of **1d**. The AO components of π MO are shown in blue font.

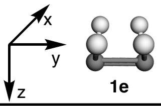 1e	Carbon AOs<br>2s (%)<br>NAO/Mulliken	2P$_x$ (%)<br>NAO/Mulliken	2P$_y$ (%)<br>NAO/Mulliken	2P$_z$ (%)<br>NAO/Mulliken	Hydrogen AOs<br>1s<br>NAO/Mulliken
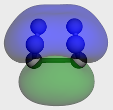 HOMO-2 (–16.6 eV)	7.4/8.4	0.0/0.0	21.0/30.0	51.2/45.6	19.4/23.4
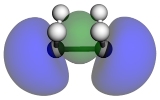 HOMO-1 (–10.7 eV)	21.5/22.8	0.0/0.0	58.0/56.4	16.4/15.2	2.9/5.2
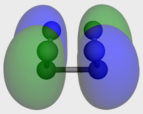 HOMO (–9.3 eV)	0.0/0.0	63.0/55.9	0.0/0.0	0.0/0.0	36.6/43.0
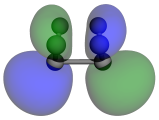 LUMO (1.2 eV)	9.3/3.6	0.0/0.0	16.7/26.4	57.6/58.8	12.3/11.4

Table 8. Orbital visualizations, energies, and AO composition analysis of MOs of **1e**. The AO components of π MO are shown in blue font.

To probe how the mixing of 2s and $2P_y$ atomic orbitals influences the structure of π MOs, we analyzed the π MO structures of **1d**, **1e**, cubene (**10**), and 1,7-quadricyclene (**11**) when contributions from certain atomic orbitals were deleted.[59] All MO structures involved in this section are calculated at the HF/6-31G(d,p) level of theory. All MO contour plots were generated with Gaussview[56] using the *.fchk* file. Cross–section contour plots of FMOs (Fig. 107) were generated using GaussView 6.0.16. Cross–section planes of **10** and **11** were defined as follows: 1) Contain both carbons on the alkene termini; 2) Contain the bisectors of two groups at each alkene termini. Isovalues of ±0.001, ±0.002, ±0.004, ±0.008, ±0.02, ±0.04, ±0.08, ±0.2, ±0.4, ±0.8, ±2, ±4, ±8, ±20 were set for all MO contour plots. Our results are summarized as follows.

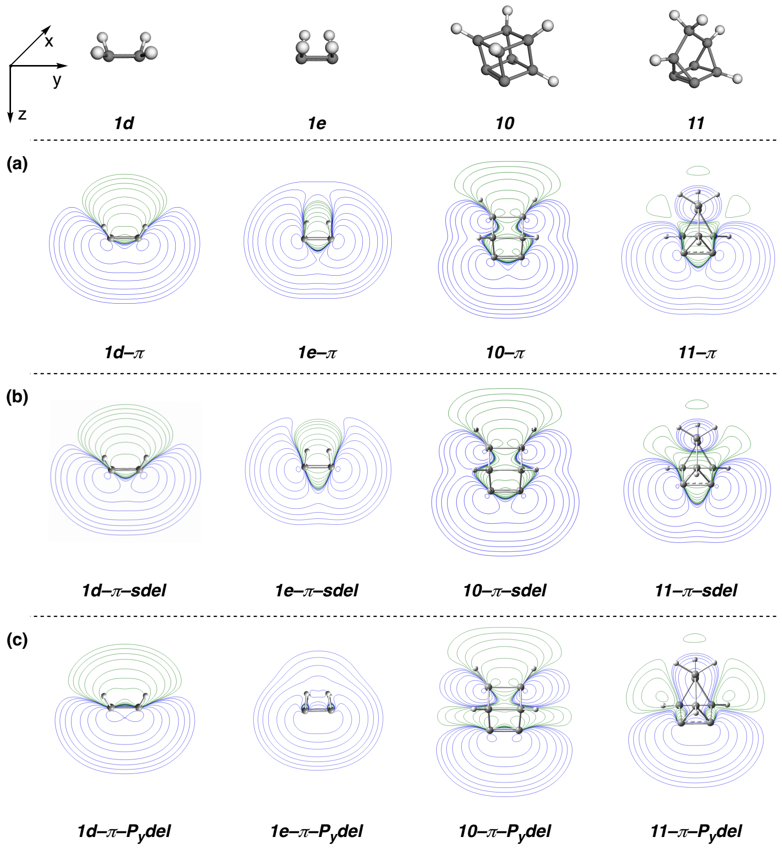

Fig. 107. Contour plots of π MOs for **1d**, **1e**, **10**, and **11**. (**a**) Original π MOs. (**b**) π MOs with 2s basis function coefficients deleted. (**c**) π MOs with $2P_y$ basis function coefficients deleted.

As shown in Figs. 5c and 107a, and Tables 6, 7, and 8, pyramidalization and hyperpyramidalization leads to a substantial change in the π MO structure, characterized as "nonequivalent extension"[60] of orbital lobes and the change of hybrid orbital directionality. As shown in Fig. 107b, following

deletion of the MO coefficients corresponding to the 2s AO component, it was observed that a substantial expansion of the minor green lobe, and shrinking of the major blue lobe in all the orbital contours (**1d**–π-sdel, **1e**–π-sdel, **10**–π-sdel, **11**–π-sdel). Moreover, upon deletion of the MO coefficients corresponding to the P_y AO component, the major lobes of the hybrid orbitals are no longer tilted toward the y axis (**1d**–π-$P_y$del, **1e**–π-$P_y$del, **10**–π-$P_y$del, **11**–π-$P_y$del, see Fig. 107c). These observations are in line with the findings reported for pyramidalized *syn*-sesquinorbornene by Houk and co-workers.[59]

H. Transition State Geometries for Trapping of each Dienophile with Anthracene (21)

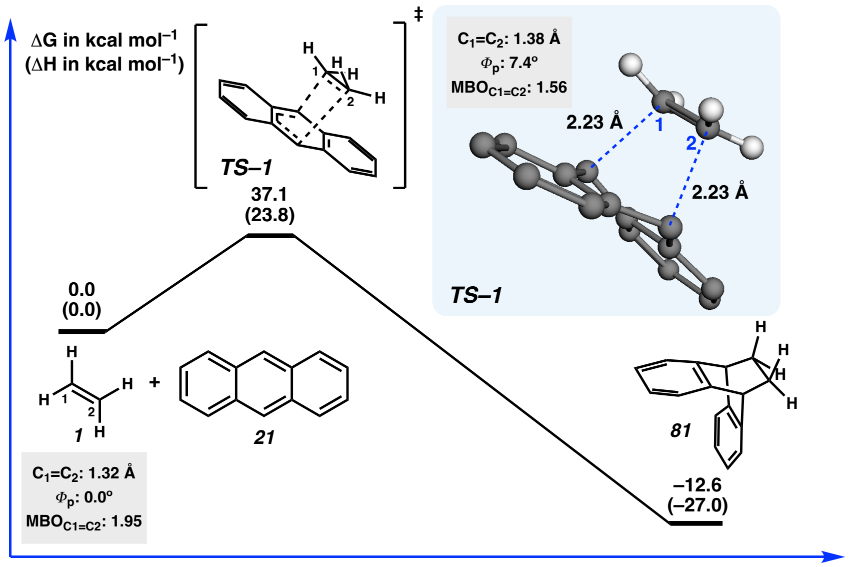

The transition state (TS) geometry of the Diels–Alder cycloaddition of ethylene (**1**) with anthracene (**21**) to give cycloadduct **81** was found at the ωB97X-D/def2-TZVP/SMD(THF) level of theory.

Structure	E	ZPE	H	T.S	T.qh-S	G(T)	qh-G(T)	qh-G(T) (kcal/mol)
**1**	–78.590392	0.049957	–78.536441	0.025515	0.025515	–78.561955	–78.561956	–49297.63
**21**	–539.534721	0.190922	–539.333284	0.045376	0.045132	–539.378660	–539.378416	–338459.96
**81**	–618.174005	0.249189	–617.912749	0.048109	0.047683	–617.960858	–617.960431	–387770.17
**TS-1**	–618.088160	0.243394	–617.831765	0.049848	0.049484	–617.881613	–617.881249	–387720.48

Table 9. Computed energies of ethylene **1,** anthracene **21**, cycloadduct **81** and **TS-1** at 25 °C at the ωB97XD/def2-TZVP/SMD(THF) level of theory. All units are in Hartrees unless marked otherwise. E = electronic energy; ZPE = zero point energy; H = enthalpy; T = temperature; S =

entropy; G(T) = Gibbs free energy; qh-S = quasi-harmonic corrected entropy; qh-G(T) = quasi-harmonic corrected Gibbs free energy.

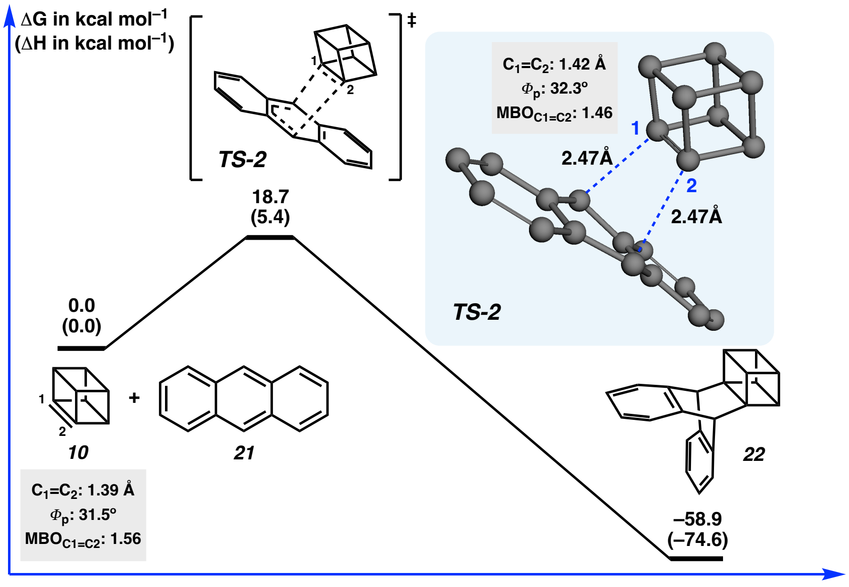

The transition state (TS) geometry of the Diels–Alder cycloaddition of cubene (**10**) with anthracene (**21**) to give cycloadduct **22** was found at the ωB97X-D/def2-TZVP/SMD(THF) level of theory.

Structure	E	ZPE	H	T.S	T.qh-S	G(T)	qh-G(T)	qh-G(T) (kcal/mol)
**10**	–308.180581	0.107781	–308.067282	0.032944	0.032946	–308.100226	–308.100227	–193332.89
**21**	–539.534721	0.190922	–539.333284	0.045376	0.045132	–539.378660	–539.378416	–338459.96
**22**	–847.839187	0.305052	–847.519448	0.053935	0.053087	–847.573383	–847.572535	–531851.77
**TS-2**	–847.707185	0.299206	–847.391967	0.058972	0.056843	–847.450939	–847.448810	–531774.13

Table 10. Computed energies of cubene **10,** anthracene **21**, cycloadduct **22** and **TS-2** at 25 °C at the ωB97XD/def2-TZVP/SMD(THF) level of theory. All units are in Hartrees unless marked otherwise. E = electronic energy; ZPE = zero point energy; H = enthalpy; T = temperature; S = entropy; G(T) = Gibbs free energy; qh-S = quasi-harmonic corrected entropy; qh-G(T) = quasi-harmonic corrected Gibbs free energy.

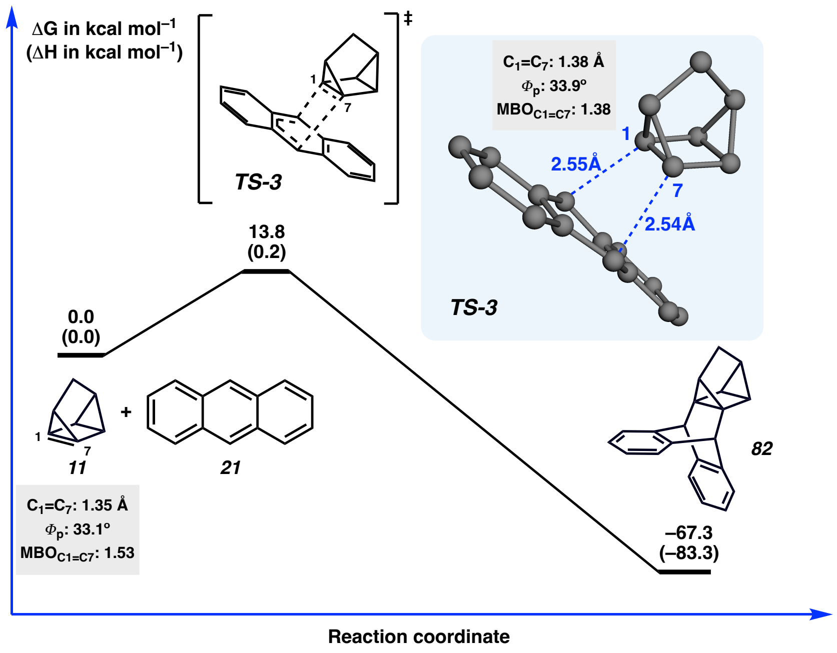

The TS geometry of the Diels–Alder cycloaddition of 1,7-quadricyclene (**11**) with anthracene (**21**) to give cycloadduct **82**[61] was found at the ωB97XD/def2-TZVP/SMD(THF) level of theory.

Structure	E	ZPE	H	T.S	T.qh-S	G(T)	qh-G(T)	qh-G(T) (kcal/mol)
**11**	–270.133305	0.101718	–270.025797	0.033775	0.033779	–270.059571	–270.059576	–169464.95
**21**	–539.534721	0.190922	–539.333284	0.045376	0.045132	–539.378660	–539.378416	–338459.96
**82**	–809.806403	0.299955	–809.491902	0.054030	0.053342	–809.545932	–809.545244	–507989.64
**TS-3**	–809.668727	0.293770	–809.358821	0.059500	0.057103	–809.418321	–809.415924	–507908.49

Table 11. Computed energies of 1,7-quadricyclene **11,** anthracene **21**, cycloadduct **82** and **TS-3** at 25 °C at the ωB97XD/def2-TZVP/SMD(THF) level of theory. All units are in Hartrees unless marked otherwise. E = electronic energy; ZPE = zero point energy; H = enthalpy; T = temperature; S = entropy; G(T) = Gibbs free energy; qh-S = quasi-harmonic corrected entropy; qh-G(T) = quasi-harmonic corrected Gibbs free energy.

Key findings from the TS analysis of the Diels–Alder cycloadditions of cubene (**10**) or 1,7-quadricyclene (**11**) with anthracene (**21**) reveal that both reactions proceed with relatively low activation free energies ($\Delta G^{\ddagger}$ = 18.7 and 13.8 kcal/mol, respectively) and substantial exothermicity (ΔH_r = –74.6 and –83.3 kcal/mol, respectively). Moreover, both reactions have early transition

states, reflective of minimal geometric distortion and changes of alkene bond order in each fragment to achieve their transition states. Of note, one-electron stepwise cycloaddition pathways were not calculated. Our prior computational study on the Diels–Alder reaction of another distorted alkene (i.e. [2.2.1] ABO **7**) with anthracene (**21**) was found to undergo a concerted pathway, despite **7** having higher diradical character (y_0 = 17%)[41] than **10** and **11** (y_0 = 14% and 13%, respectively).

I. Mayer Bond Order Analysis of Systematically Distorted Ethylene (1)

1-Bent

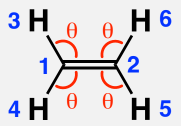

θ = Angles scanned
Total scans = 15
Increments = 4°
Angle = 93.67–149.67°

Constraints
Length (Å):
C1–C2 = 1.321
H3–C1 = 1.083, H4–C1 = 1.083
H5–C1 = 1.083, H6–C1 = 1.083
Torsion (°):
H3–C1–C2–H6 = 0
H4–C1–C2–H5 = 0
H3–C1–C2–H5 = 0
H4–C1–C2–H6 = 0

1-Pyramidalized

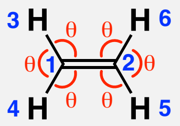

θ = Angles scanned
Total scans = 14
Increments = 3°
Angle = 84–120°
Pyramidization
Φ_p (C_1) and Φ_p (C_2)
= 0–39.4°

Constraints
Length (Å):
C1–C2 = 1.321
H3–C1 = 1.083, H4–C1 = 1.083
H5–C1 = 1.083, H6–C1 = 1.083
Torsion (°):
H3–C1–C2–H6 = 0
H4–C1–C2–H5 = 0

1-Length

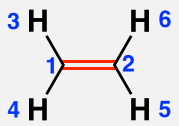

C1–C2 Bond length scan
Total scans = 13
Increments = 0.04 A
Length = 1.161–1.641 A

Constraints
Length (Å):
H3–C1 = 1.083, H4–C1 = 1.083
H5–C1 = 1.083, H6–C1 = 1.083
Torsion (°):
H3–C1–C2–H6 = 0
H4–C1–C2–H5 = 0
H3–C1–C2–H5 = 0
H4–C1–C2–H6 = 0
Angle (°):
H3–C1–H4 = 120, H3–C1–C2 = 120
H4–C1–C2 = 120, H5–C2–C1 = 120
H5–C2–H6 = 120, H6–C2–C1 = 120

To interrogate how distortion of each geometric parameters could individually impact the alkene bond order, we performed Mayer bond order analysis on systemically distorted ethylene **1**. The equilibrium geometry of ethylene (**1–eq)** was obtained at the ωB97X-D/def2-TZVP level of theory. Rigid scans as shown above were carried out to evaluate the effect of each individual geometric distortion parameter on Mayer bond order, with all C–H bonds constrained to the bond length obtained from the equilibrium geometry (1.083 Å). In each scan involving the changing of bond angles (i.e., **1-Bent** and **1-Pyramidalized**), all scanned angles (θ) are changed simultaneously at the same step increment throughout the scan. In addition to pyramidalization and bending, the impact of C=C bond length on alkene MBO was also studied (Table 14, also see ref. 62 for prior study by Mayer). The keyword *pop=(nboread, always)* was implemented in the route line and the keywords *$nbo bndidx mulorb $end* were included at the end of Gaussian input file. Since Mayer Bond Order has been known to be somewhat sensitive to the choice of computation methods,[44] three DFT methods were evaluated, and the results were summarized herein. The wavefunctions of the maximally distorted structures in each scan (**1a**, **1b**, **1e**, **1ac**, **1ao**) were subjected to a stability check, confirming these structures are not diradical species albeit highly distorted.

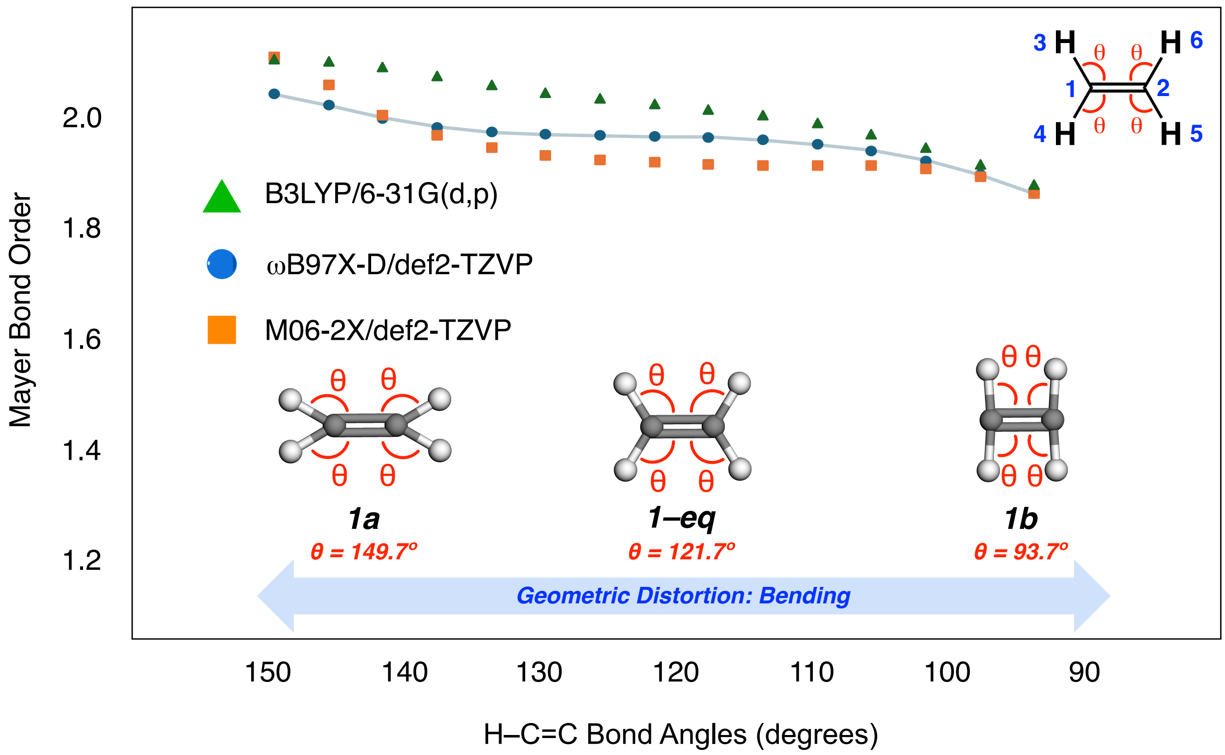

Structure	H–C=C Bond Angles (θ, degrees)	Mayer Bond Orders (MBOs) of C=C bond		
		ωB97X-D/ def2-TZVP	M06-2X/ def2-TZVP	B3LYP/ 6-31G(d,p)
**1b**	93.7	1.86	1.86	1.88
**1f**	97.7	1.90	1.89	1.91
**1g**	101.7	1.92	1.91	1.95
**1h**	105.7	1.94	1.91	1.97
**1i**	109.7	1.95	1.91	1.99
**1j**	113.7	1.96	1.91	2.00
**1k**	117.7	1.96	1.92	2.02
**1–eq**	121.7	1.97	1.92	2.02
**1l**	125.7	1.97	1.92	2.03
**1m**	129.7	1.97	1.93	2.05
**1n**	133.7	1.97	1.94	2.06
**1o**	137.7	1.98	1.97	2.08
**1p**	141.7	2.00	2.01	2.09
**1q**	145.7	2.02	2.06	2.10
**1a**	149.7	2.04	2.11	2.11

Table 12. Mayer bond orders (MBOs) of systemically bent ethylene **1-Bent**.

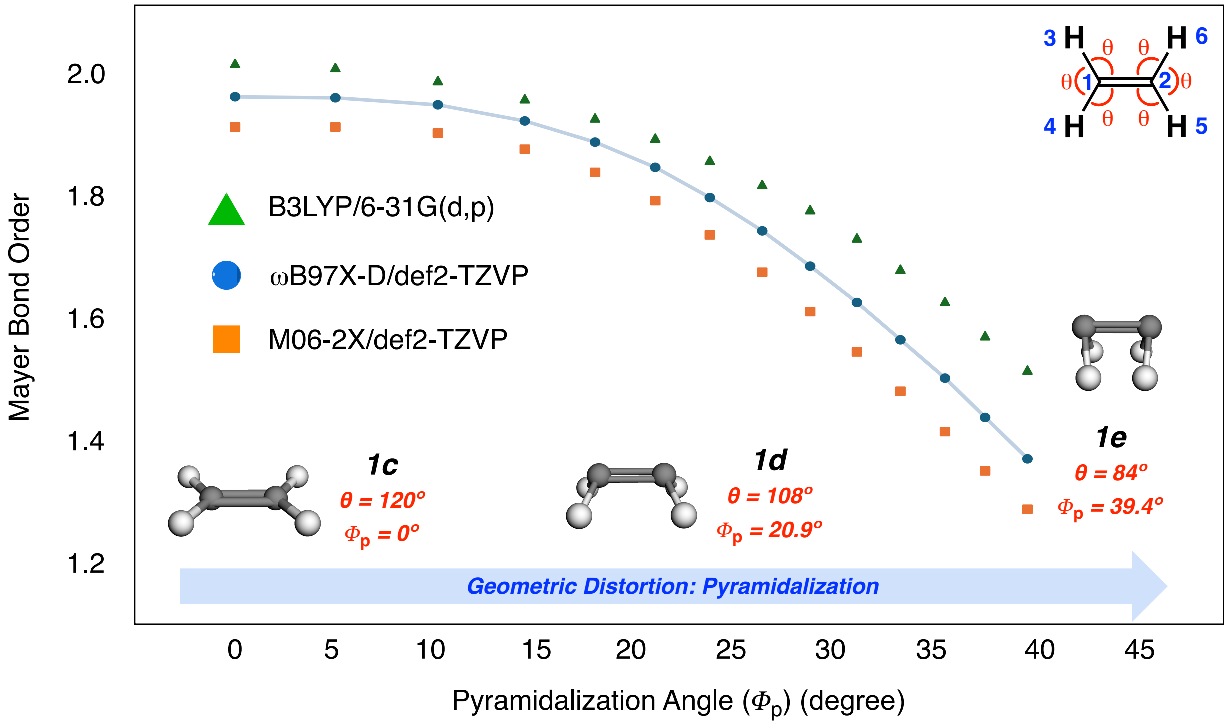

Structure	θ (º)	Φ_p (º)	$\Phi_{p(Borden)}$ (º)	Mayer Bond Orders (MBOs) of C=C bond		
				ωB97X-D/ def2-TZVP	M06-2X/ def2-TZVP	B3LYP/ 6-31G(d,p)
**1c**	120.0	0.0	0.0	1.97	1.92	2.02
**1r**	119.3	5.0	14.9	1.96	1.92	2.01
**1s**	117.0	10.1	29.7	1.95	1.91	1.99
**1t**	114.0	14.4	41.7	1.93	1.88	1.96
**1u**	111.0	17.9	50.8	1.89	1.84	1.93
**1d**	108.0	20.9	58.3	1.85	1.80	1.90
**1v**	105.0	23.6	64.8	1.80	1.74	1.86
**1w**	102.0	26.2	70.7	1.75	1.68	1.82
**1x**	99.0	28.6	76.1	1.69	1.61	1.78
**1y**	96.0	30.9	81.0	1.63	1.55	1.73
**1z**	93.0	33.1	85.6	1.57	1.48	1.68
**1aa**	90.0	35.3	90.0	1.51	1.42	1.63
**1ab**	87.0	37.3	94.1	1.44	1.35	1.57
**1e**	84.0	39.4	98.1	1.37	1.29	1.52

Table 13. Mayer bond orders (MBOs) of systematically pyramidalized ethylene **1-Pyramidalized**.

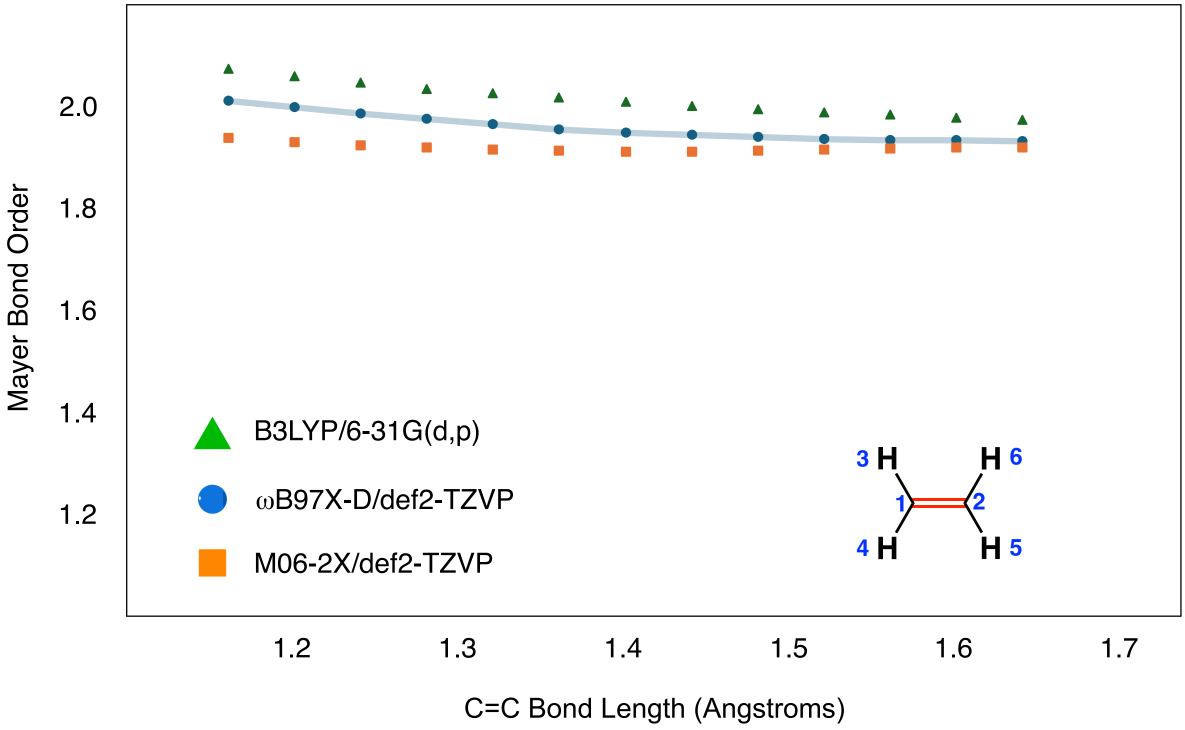

Structure	C=C Length (Å)	Mayer Bond Orders (MBOs) of C=C bond		
		ωB97X-D/def2-TZVP	M06-2X/def2-TZVP	B3LYP/6-31G(d,p)
**1ac**	1.161	2.01	1.94	2.07
**1ad**	1.201	2.00	1.93	2.06
**1ae**	1.241	1.99	1.93	2.04
**1af**	1.281	1.96	1.92	2.03
**1–eq**	1.321	1.97	1.92	2.02
**1ag**	1.361	1.96	1.92	2.02
**1ah**	1.401	1.95	1.92	2.01
**1ai**	1.441	1.95	1.92	2.00
**1aj**	1.481	1.94	1.92	2.00
**1ak**	1.521	1.94	1.92	1.99
**1al**	1.561	1.94	1.92	1.98
**1am**	1.601	1.94	1.92	1.98
**1an**	1.641	1.94	1.92	1.97

Table 14. Mayer bond orders (MBOs) of systematically elongated/shortened ethylene **1-Length**.

As shown in Fig. 108, Mayer Bond Orders (MBOs) of alkenes **2–7**, **10**, **11** were plotted against their respective Haddon pyramidalization angles (Φ_p), alongside the MBOs of systematically

pyramidalized ethylene structures (**1c** to **1e**, see Table 13). The graph shows a general trend that larger alkene pyramidalization angles typically lead to lower alkene bond orders. It should be noted, however, that other factors in alkenes **2–7**, **10**, **11** that are not present in ethylene, such as twisting or hyperconjugation, can also affect alkene bond order. This helps to explain the imperfect correlation between systematically distorted ethylene and alkenes **2–7**, **10**, **11** when considering the MBO analysis shown in Fig. 108.

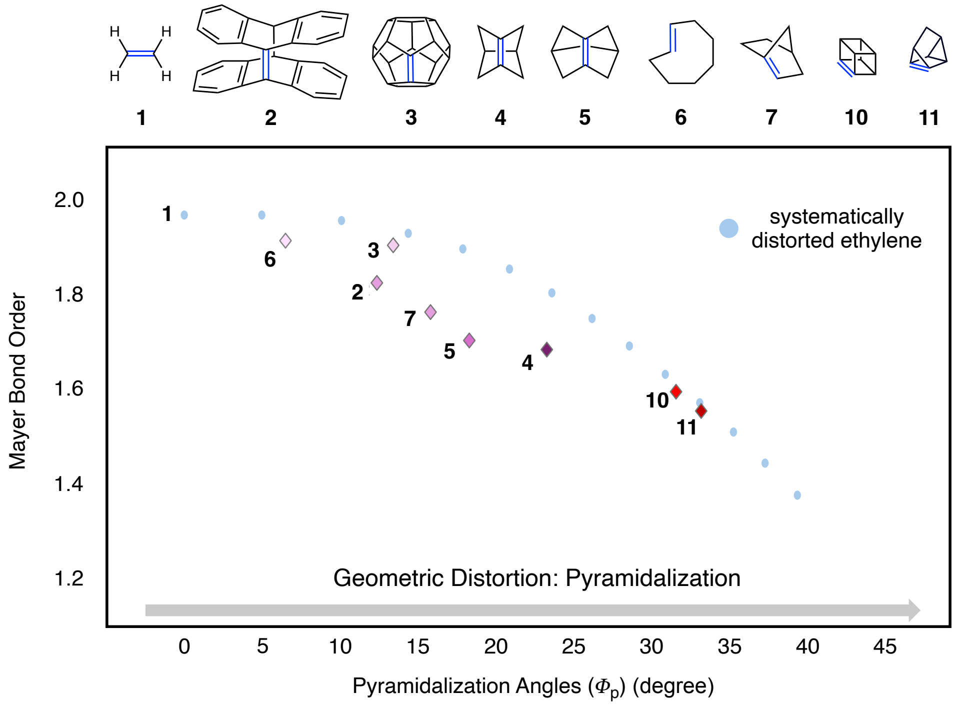

Fig. 108. Mayer bond order (MBO) plotted against pyramidalization angles (Φ_p) of systematically distorted theoretical ethylene (**1**) and alkenes **2–7**, **10** and **11**.

J. Full Mayer Bond Orders Output of Compounds 2–9, *cis*-8, 10 and 11

All geometry optimizations and MBO analysis for compounds reported in this section were carried out at ωB97X-D/def2-TZVP level of theory. All Cartesian coordinates of optimized geometries are reported in Part II, Section K. Full Mayer bond order outputs of compounds **2–9,** *cis*-**6, 10** and **11** were generated using Multiwfn v3.8[36,37] from the *.fchk* file obtained from Gaussian16 runs, and are reported in this section. For compounds **2**–**7** and *cis*-**6**, the bond orders of corresponding double bonds of interest are highlighted and bolded in blue.

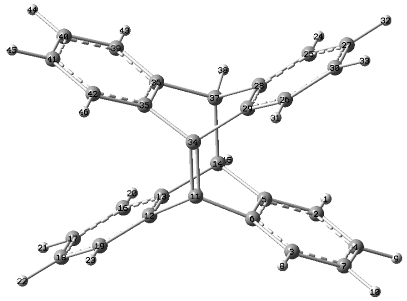

2 (Φ_p = 12.4º)

	1	2	3	4	5
1	0.97625069	0.99144912	0.00072507	-0.01867825	-0.02466272
2	0.99144912	3.87803038	0.08411331	1.38345386	1.34085434
3	0.00072507	0.08411331	3.88353349	0.06472916	0.02331889
4	-0.01867825	1.38345386	0.06472916	3.87988863	0.01519785
5	-0.02466272	1.34085434	0.02331889	0.01519785	4.08461129
6	0.01315369	0.01918628	1.31651443	0.03596847	1.44328111
7	0.00800642	0.05729742	1.37550093	1.39922537	0.04317160
8	0.00012492	0.00070726	1.00656433	0.00855450	0.01131483
9	-0.00090673	-0.02014679	0.00736100	0.99345132	0.00716305
10	0.00059545	0.00734518	-0.02009104	-0.01877600	0.00131451
11	0.00084494	0.00434028	0.00473356	-0.00015168	-0.02018864
12	-0.00014239	-0.00448802	0.00911924	0.00375577	-0.01896773
13	0.00073914	0.01547059	-0.00626007	-0.00159362	0.19647926
14	-0.00029473	-0.01391551	0.01229565	0.00555230	0.92162708
15	0.00426426	0.00155380	0.00003076	-0.00016399	0.01678728
16	0.00019792	0.00485052	0.00146208	0.00025041	0.01547059
17	0.00000051	0.00025041	0.00090898	0.00089180	-0.00159362
18	0.00001192	0.00082600	0.00029532	0.00009249	0.00295640
19	-0.00003848	0.00146208	0.00433414	0.00090898	-0.00626007
20	0.00000181	0.00019792	-0.00003848	0.00000051	0.00073914
21	0.00000053	0.00003021	-0.00001555	0.00000741	0.00007433
22	0.00000088	0.00000195	0.00003118	0.00002678	0.00009902
23	0.00000441	-0.00002056	0.00013784	-0.00000742	-0.00001867
24	0.00007921	-0.00005519	0.00001748	0.00003452	0.00058996
25	-0.00005519	-0.00696576	0.00064081	0.00061967	0.01358707
26	0.00001748	0.00064081	-0.00707966	-0.00014299	-0.00408623
27	0.00003452	0.00061967	-0.00014299	0.00063665	0.00067923
28	0.00058996	0.01358707	-0.00408623	0.00067923	0.04958870
29	0.00010154	-0.00563481	0.02063836	0.00275227	-0.01371423
30	0.00002696	0.00007537	0.00053969	0.00014007	0.00100015
31	0.00000741	0.00006091	0.00033034	0.00005598	0.00010956
32	0.00000019	-0.00001306	0.00003095	0.00002122	0.00002516

33	0.00000487	0.00007364	-0.00009244	-0.00006068	-0.00001621
34	0.00085056	0.00418314	-0.01550841	-0.00014761	0.01234856
35	-0.00002572	0.00089487	0.00145095	0.00111791	0.00091162
36	0.00005891	-0.00080054	0.00139910	0.00048637	0.00704168
37	-0.00088756	-0.00525694	-0.00190807	0.00045885	0.04647578
38	-0.00000102	0.00129912	-0.00012633	-0.00000735	0.00044541
39	0.00001916	-0.00009361	0.00008682	0.00018072	-0.00080054
40	-0.00001316	0.00018072	0.00020247	0.00004367	0.00048637
41	0.00000268	0.00030384	0.00012369	0.00009183	0.00004601
42	0.00002648	0.00008682	0.00112605	0.00020247	0.00139910
43	0.00001176	0.00001916	0.00002648	-0.00001316	0.00005891
44	0.00000029	0.00000694	0.00000373	0.00000124	0.00000766
45	-0.00000005	0.00000176	0.00002742	0.00000880	0.00001506
46	0.00000377	-0.00000318	0.00006253	0.00003296	0.00025469

	6	7	8	9	10
1	0.01315369	0.00800642	0.00012492	-0.00090673	0.00059545
2	0.01918628	0.05729742	0.00070726	-0.02014679	0.00734518
3	1.31651443	1.37550093	1.00656433	0.00736100	-0.02009104
4	0.03596847	1.39922537	0.00855450	0.99345132	-0.01877600
5	1.44328111	0.04317160	0.01131483	0.00716305	0.00131451
6	3.88164137	-0.00401042	-0.02707443	0.00121210	0.00699044
7	-0.00401042	3.84812553	-0.02679025	-0.01840260	0.99288378
8	-0.02707443	-0.02679025	0.97882272	0.00054450	-0.00089019
9	0.00121210	-0.01840260	0.00054450	0.97092943	-0.00103565
10	0.00699044	0.99288378	-0.00089019	-0.00103565	0.97039661
11	0.91802449	0.00664140	-0.00128913	0.00008311	0.00137708
12	0.08733436	-0.00127233	0.00052958	0.00017846	0.00007845
13	-0.01896773	0.00295640	-0.00001867	0.00007433	0.00009902
14	-0.04199350	0.00267591	0.00115229	0.00114319	0.00010197
15	0.01036661	0.00029159	0.00012599	0.00001867	0.00004012
16	-0.00448802	0.00082600	-0.00002056	0.00003021	0.00000195
17	0.00375577	0.00009249	-0.00000742	0.00000741	0.00002678
18	-0.00127233	0.00071565	0.00000570	0.00003186	0.00001585
19	0.00911924	0.00029532	0.00013784	-0.00001555	0.00003118
20	-0.00014239	0.00001192	0.00000441	0.00000053	0.00000088
21	0.00017846	0.00003186	-0.00000067	0.00000268	0.00000073
22	0.00007845	0.00001585	0.00000026	0.00000073	0.00000102
23	0.00052958	0.00000570	0.00004484	-0.00000067	0.00000026
24	0.00010154	0.00002696	0.00000741	0.00000019	0.00000487
25	-0.00563481	0.00007537	0.00006091	-0.00001306	0.00007364
26	0.02063836	0.00053969	0.00033034	0.00003095	-0.00009244
27	0.00275227	0.00014007	0.00005598	0.00002122	-0.00006068
28	-0.01371423	0.00100015	0.00010956	0.00002516	-0.00001621
29	0.06641742	-0.00002778	0.00033967	0.00000723	0.00011963
30	-0.00002778	0.00089973	0.00005108	-0.00002332	0.00005434

31	0.00033967	0.00005108	0.00020902	0.00000202	-0.00000006
32	0.00000723	-0.00002332	0.00000202	0.00000490	0.00000411
33	0.00011963	0.00005434	-0.00000006	0.00000411	0.00000576
34	0.01833484	0.00030395	0.00328980	-0.00001248	0.00012586
35	0.01135160	0.00034477	0.00034101	0.00000541	0.00001344
36	0.00091162	0.00004601	0.00025469	0.00000766	0.00001506
37	0.00789698	0.00356974	-0.00010059	0.00001999	-0.00000477
38	0.00025979	0.00031259	0.00000344	0.00003480	0.00000178
39	0.00089487	0.00030384	-0.00000318	0.00000694	0.00000176
40	0.00111791	0.00009183	0.00003296	0.00000124	0.00000880
41	0.00034477	0.00013211	-0.00001954	0.00000713	0.00000044
42	0.00145095	0.00012369	0.00006253	0.00000373	0.00002742
43	-0.00002572	0.00000268	0.00000377	0.00000029	-0.00000005
44	0.00000541	0.00000713	0.00000004	-0.00000001	0.00000013
45	0.00001344	0.00000044	0.00000161	0.00000013	0.00000042
46	0.00034101	-0.00001954	0.00007035	0.00000004	0.00000161
	11	12	13	14	15
1	0.00084494	-0.00014239	0.00073914	-0.00029473	0.00426426
2	0.00434028	-0.00448802	0.01547059	-0.01391551	0.00155380
3	0.00473356	0.00911924	-0.00626007	0.01229565	0.00003076
4	-0.00015168	0.00375577	-0.00159362	0.00555230	-0.00016399
5	-0.02018864	-0.01896773	0.19647926	0.92162708	0.01678728
6	0.91802449	0.08733436	-0.01896773	-0.04199350	0.01036661
7	0.00664140	-0.00127233	0.00295640	0.00267591	0.00029159
8	-0.00128913	0.00052958	-0.00001867	0.00115229	0.00012599
9	0.00008311	0.00017846	0.00007433	0.00114319	0.00001867
10	0.00137708	0.00007845	0.00009902	0.00010197	0.00004012
11	3.73593746	0.91802449	-0.02018864	-0.02825267	-0.01313570
12	0.91802449	3.88164137	1.44328111	-0.04199350	0.01036661
13	-0.02018864	1.44328111	4.08461129	0.92162708	0.01678728
14	-0.02825267	-0.04199350	0.92162708	3.72048300	0.89410202
15	-0.01313570	0.01036661	0.01678728	0.89410202	0.95317884
16	0.00434028	0.01918628	1.34085434	-0.01391551	0.00155380
17	-0.00015168	0.03596847	0.01519785	0.00555230	-0.00016399
18	0.00664140	-0.00401042	0.04317160	0.00267591	0.00029159
19	0.00473356	1.31651443	0.02331889	0.01229565	0.00003076
20	0.00084494	0.01315369	-0.02466272	-0.00029473	0.00426426
21	0.00008311	0.00121210	0.00716305	0.00114319	0.00001867
22	0.00137708	0.00699044	0.00131451	0.00010197	0.00004012
23	-0.00128913	-0.02707443	0.01131483	0.00115229	0.00012599
24	0.00085056	-0.00002572	0.00005891	-0.00088756	-0.00000102
25	0.00418314	0.00089487	-0.00080054	-0.00525694	0.00129912
26	-0.01550841	0.00145095	0.00139910	-0.00190807	-0.00012633
27	-0.00014761	0.00111791	0.00048637	0.00045885	-0.00000735
28	0.01234856	0.00091162	0.00704168	0.04647578	0.00044541

29	0.01833484	0.01135160	0.00091162	0.00789698	0.00025979
30	0.00030395	0.00034477	0.00004601	0.00356974	0.00031259
31	0.00328980	0.00034101	0.00025469	-0.00010059	0.00000344
32	-0.00001248	0.00000541	0.00000766	0.00001999	0.00003480
33	0.00012586	0.00001344	0.00001506	-0.00000477	0.00000178
34	**1.81602378**	0.01833484	0.01234856	0.08086246	0.00407237
35	0.01833484	0.06641742	-0.01371423	0.00789698	0.00025979
36	0.01234856	-0.01371423	0.04958870	0.04647578	0.00044541
37	0.08086246	0.00789698	0.04647578	0.89292411	0.00363095
38	0.00407237	0.00025979	0.00044541	0.00363095	-0.00656543
39	0.00418314	-0.00563481	0.01358707	-0.00525694	0.00129912
40	-0.00014761	0.00275227	0.00067923	0.00045885	-0.00000735
41	0.00030395	-0.00002778	0.00100015	0.00356974	0.00031259
42	-0.01550841	0.02063836	-0.00408623	-0.00190807	-0.00012633
43	0.00085056	0.00010154	0.00058996	-0.00088756	-0.00000102
44	-0.00001248	0.00000723	0.00002516	0.00001999	0.00003480
45	0.00012586	0.00011963	-0.00001621	-0.00000477	0.00000178
46	0.00328980	0.00033967	0.00010956	-0.00010059	0.00000344
	16	17	18	19	20
1	0.00019792	0.00000051	0.00001192	-0.00003848	0.00000181
2	0.00485052	0.00025041	0.00082600	0.00146208	0.00019792
3	0.00146208	0.00090898	0.00029532	0.00433414	-0.00003848
4	0.00025041	0.00089180	0.00009249	0.00090898	0.00000051
5	0.01547059	-0.00159362	0.00295640	-0.00626007	0.00073914
6	-0.00448802	0.00375577	-0.00127233	0.00911924	-0.00014239
7	0.00082600	0.00009249	0.00071565	0.00029532	0.00001192
8	-0.00002056	-0.00000742	0.00000570	0.00013784	0.00000441
9	0.00003021	0.00000741	0.00003186	-0.00001555	0.00000053
10	0.00000195	0.00002678	0.00001585	0.00003118	0.00000088
11	0.00434028	-0.00015168	0.00664140	0.00473356	0.00084494
12	0.01918628	0.03596847	-0.00401042	1.31651443	0.01315369
13	1.34085434	0.01519785	0.04317160	0.02331889	-0.02466272
14	-0.01391551	0.00555230	0.00267591	0.01229565	-0.00029473
15	0.00155380	-0.00016399	0.00029159	0.00003076	0.00426426
16	3.87803038	1.38345386	0.05729742	0.08411331	0.99144912
17	1.38345386	3.87988863	1.39922537	0.06472916	-0.01867825
18	0.05729742	1.39922537	3.84812553	1.37550093	0.00800642
19	0.08411331	0.06472916	1.37550093	3.88353349	0.00072507
20	0.99144912	-0.01867825	0.00800642	0.00072507	0.97625069
21	-0.02014679	0.99345132	-0.01840260	0.00736100	-0.00090673
22	0.00734518	-0.01877600	0.99288378	-0.02009104	0.00059545
23	0.00070726	0.00855450	-0.02679025	1.00656433	0.00012492
24	0.00001916	-0.00001316	0.00000268	0.00002648	0.00001176
25	-0.00009361	0.00018072	0.00030384	0.00008682	0.00001916
26	0.00008682	0.00020247	0.00012369	0.00112605	0.00002648

27	0.00018072	0.00004367	0.00009183	0.00020247	-0.00001316
28	-0.00080054	0.00048637	0.00004601	0.00139910	0.00005891
29	0.00089487	0.00111791	0.00034477	0.00145095	-0.00002572
30	0.00030384	0.00009183	0.00013211	0.00012369	0.00000268
31	-0.00000318	0.00003296	-0.00001954	0.00006253	0.00000377
32	0.00000694	0.00000124	0.00000713	0.00000373	0.00000029
33	0.00000176	0.00000880	0.00000044	0.00002742	-0.00000005
34	0.00418314	-0.00014761	0.00030395	-0.01550841	0.00085056
35	-0.00563481	0.00275227	-0.00002778	0.02063836	0.00010154
36	0.01358707	0.00067923	0.00100015	-0.00408623	0.00058996
37	-0.00525694	0.00045885	0.00356974	-0.00190807	-0.00088756
38	0.00129912	-0.00000735	0.00031259	-0.00012633	-0.00000102
39	-0.00696576	0.00061967	0.00007537	0.00064081	-0.00005519
40	0.00061967	0.00063665	0.00014007	-0.00014299	0.00003452
41	0.00007537	0.00014007	0.00089973	0.00053969	0.00002696
42	0.00064081	-0.00014299	0.00053969	-0.00707966	0.00001748
43	-0.00005519	0.00003452	0.00002696	0.00001748	0.00007921
44	-0.00001306	0.00002122	-0.00002332	0.00003095	0.00000019
45	0.00007364	-0.00006068	0.00005434	-0.00009244	0.00000487
46	0.00006091	0.00005598	0.00005108	0.00033034	0.00000741

	21	22	23	24	25
1	0.00000053	0.00000088	0.00000441	0.00007921	-0.00005519
2	0.00003021	0.00000195	-0.00002056	-0.00005519	-0.00696576
3	-0.00001555	0.00003118	0.00013784	0.00001748	0.00064081
4	0.00000741	0.00002678	-0.00000742	0.00003452	0.00061967
5	0.00007433	0.00009902	-0.00001867	0.00058996	0.01358707
6	0.00017846	0.00007845	0.00052958	0.00010154	-0.00563481
7	0.00003186	0.00001585	0.00000570	0.00002696	0.00007537
8	-0.00000067	0.00000026	0.00004484	0.00000741	0.00006091
9	0.00000268	0.00000073	-0.00000067	0.00000019	-0.00001306
10	0.00000073	0.00000102	0.00000026	0.00000487	0.00007364
11	0.00008311	0.00137708	-0.00128913	0.00085056	0.00418314
12	0.00121210	0.00699044	-0.02707443	-0.00002572	0.00089487
13	0.00716305	0.00131451	0.01131483	0.00005891	-0.00080054
14	0.00114319	0.00010197	0.00115229	-0.00088756	-0.00525694
15	0.00001867	0.00004012	0.00012599	-0.00000102	0.00129912
16	-0.02014679	0.00734518	0.00070726	0.00001916	-0.00009361
17	0.99345132	-0.01877600	0.00855450	-0.00001316	0.00018072
18	-0.01840260	0.99288378	-0.02679025	0.00000268	0.00030384
19	0.00736100	-0.02009104	1.00656433	0.00002648	0.00008682
20	-0.00090673	0.00059545	0.00012492	0.00001176	0.00001916
21	0.97092943	-0.00103565	0.00054450	0.00000029	0.00000694
22	-0.00103565	0.97039661	-0.00089019	-0.00000005	0.00000176
23	0.00054450	-0.00089019	0.97882272	0.00000377	-0.00000318
24	0.00000029	-0.00000005	0.00000377	0.97625069	0.99144912

25	0.00000694	0.00000176	-0.00000318	0.99144912	3.87803038
26	0.00000373	0.00002742	0.00006253	0.00072507	0.08411331
27	0.00000124	0.00000880	0.00003296	-0.01867825	1.38345386
28	0.00000766	0.00001506	0.00025469	-0.02466272	1.34085434
29	0.00000541	0.00001344	0.00034101	0.01315369	0.01918628
30	0.00000713	0.00000044	-0.00001954	0.00800642	0.05729742
31	0.00000004	0.00000161	0.00007035	0.00012492	0.00070726
32	-0.00000001	0.00000013	0.00000004	-0.00090673	-0.02014679
33	0.00000013	0.00000042	0.00000161	0.00059545	0.00734518
34	-0.00001248	0.00012586	0.00328980	0.00084494	0.00434028
35	0.00000723	0.00011963	0.00033967	-0.00014239	-0.00448802
36	0.00002516	-0.00001621	0.00010956	0.00073914	0.01547059
37	0.00001999	-0.00000477	-0.00010059	-0.00029473	-0.01391551
38	0.00003480	0.00000178	0.00000344	0.00426426	0.00155380
39	-0.00001306	0.00007364	0.00006091	0.00019792	0.00485052
40	0.00002122	-0.00006068	0.00005598	0.00000051	0.00025041
41	-0.00002332	0.00005434	0.00005108	0.00001192	0.00082600
42	0.00003095	-0.00009244	0.00033034	-0.00003848	0.00146208
43	0.00000019	0.00000487	0.00000741	0.00000181	0.00019792
44	0.00000490	0.00000411	0.00000202	0.00000053	0.00003021
45	0.00000411	0.00000576	-0.00000006	0.00000088	0.00000195
46	0.00000202	-0.00000006	0.00020902	0.00000441	-0.00002056

	26	27	28	29	30
1	0.00001748	0.00003452	0.00058996	0.00010154	0.00002696
2	0.00064081	0.00061967	0.01358707	-0.00563481	0.00007537
3	-0.00707966	-0.00014299	-0.00408623	0.02063836	0.00053969
4	-0.00014299	0.00063665	0.00067923	0.00275227	0.00014007
5	-0.00408623	0.00067923	0.04958870	-0.01371423	0.00100015
6	0.02063836	0.00275227	-0.01371423	0.06641742	-0.00002778
7	0.00053969	0.00014007	0.00100015	-0.00002778	0.00089973
8	0.00033034	0.00005598	0.00010956	0.00033967	0.00005108
9	0.00003095	0.00002122	0.00002516	0.00000723	-0.00002332
10	-0.00009244	-0.00006068	-0.00001621	0.00011963	0.00005434
11	-0.01550841	-0.00014761	0.01234856	0.01833484	0.00030395
12	0.00145095	0.00111791	0.00091162	0.01135160	0.00034477
13	0.00139910	0.00048637	0.00704168	0.00091162	0.00004601
14	-0.00190807	0.00045885	0.04647578	0.00789698	0.00356974
15	-0.00012633	-0.00000735	0.00044541	0.00025979	0.00031259
16	0.00008682	0.00018072	-0.00080054	0.00089487	0.00030384
17	0.00020247	0.00004367	0.00048637	0.00111791	0.00009183
18	0.00012369	0.00009183	0.00004601	0.00034477	0.00013211
19	0.00112605	0.00020247	0.00139910	0.00145095	0.00012369
20	0.00002648	-0.00001316	0.00005891	-0.00002572	0.00000268
21	0.00000373	0.00000124	0.00000766	0.00000541	0.00000713
22	0.00002742	0.00000880	0.00001506	0.00001344	0.00000044

23	0.00006253	0.00003296	0.00025469	0.00034101	-0.00001954
24	0.00072507	-0.01867825	-0.02466272	0.01315369	0.00800642
25	0.08411331	1.38345386	1.34085434	0.01918628	0.05729742
26	3.88353349	0.06472916	0.02331889	1.31651443	1.37550093
27	0.06472916	3.87988863	0.01519785	0.03596847	1.39922537
28	0.02331889	0.01519785	4.08461129	1.44328111	0.04317160
29	1.31651443	0.03596847	1.44328111	3.88164137	-0.00401042
30	1.37550093	1.39922537	0.04317160	-0.00401042	3.84812553
31	1.00656433	0.00855450	0.01131483	-0.02707443	-0.02679025
32	0.00736100	0.99345132	0.00716305	0.00121210	-0.01840260
33	-0.02009104	-0.01877600	0.00131451	0.00699044	0.99288378
34	0.00473356	-0.00015168	-0.02018864	0.91802449	0.00664140
35	0.00911924	0.00375577	-0.01896773	0.08733436	-0.00127233
36	-0.00626007	-0.00159362	0.19647926	-0.01896773	0.00295640
37	0.01229565	0.00555230	0.92162708	-0.04199350	0.00267591
38	0.00003076	-0.00016399	0.01678728	0.01036661	0.00029159
39	0.00146208	0.00025041	0.01547059	-0.00448802	0.00082600
40	0.00090898	0.00089180	-0.00159362	0.00375577	0.00009249
41	0.00029532	0.00009249	0.00295640	-0.00127233	0.00071565
42	0.00433414	0.00090898	-0.00626007	0.00911924	0.00029532
43	-0.00003848	0.00000051	0.00073914	-0.00014239	0.00001192
44	-0.00001555	0.00000741	0.00007433	0.00017846	0.00003186
45	0.00003118	0.00002678	0.00009902	0.00007845	0.00001585
46	0.00013784	-0.00000742	-0.00001867	0.00052958	0.00000570

	31	32	33	34	35
1	0.00000741	0.00000019	0.00000487	0.00085056	-0.00002572
2	0.00006091	-0.00001306	0.00007364	0.00418314	0.00089487
3	0.00033034	0.00003095	-0.00009244	-0.01550841	0.00145095
4	0.00005598	0.00002122	-0.00006068	-0.00014761	0.00111791
5	0.00010956	0.00002516	-0.00001621	0.01234856	0.00091162
6	0.00033967	0.00000723	0.00011963	0.01833484	0.01135160
7	0.00005108	-0.00002332	0.00005434	0.00030395	0.00034477
8	0.00020902	0.00000202	-0.00000006	0.00328980	0.00034101
9	0.00000202	0.00000490	0.00000411	-0.00001248	0.00000541
10	-0.00000006	0.00000411	0.00000576	0.00012586	0.00001344
11	0.00328980	-0.00001248	0.00012586	**1.81602378**	0.01833484
12	0.00034101	0.00000541	0.00001344	0.01833484	0.06641742
13	0.00025469	0.00000766	0.00001506	0.01234856	-0.01371423
14	-0.00010059	0.00001999	-0.00000477	0.08086246	0.00789698
15	0.00000344	0.00003480	0.00000178	0.00407237	0.00025979
16	-0.00000318	0.00000694	0.00000176	0.00418314	-0.00563481
17	0.00003296	0.00000124	0.00000880	-0.00014761	0.00275227
18	-0.00001954	0.00000713	0.00000044	0.00030395	-0.00002778
19	0.00006253	0.00000373	0.00002742	-0.01550841	0.02063836
20	0.00000377	0.00000029	-0.00000005	0.00085056	0.00010154

21	0.00000004	-0.00000001	0.00000013	-0.00001248	0.00000723
22	0.00000161	0.00000013	0.00000042	0.00012586	0.00011963
23	0.00007035	0.00000004	0.00000161	0.00328980	0.00033967
24	0.00012492	-0.00090673	0.00059545	0.00084494	-0.00014239
25	0.00070726	-0.02014679	0.00734518	0.00434028	-0.00448802
26	1.00656433	0.00736100	-0.02009104	0.00473356	0.00911924
27	0.00855450	0.99345132	-0.01877600	-0.00015168	0.00375577
28	0.01131483	0.00716305	0.00131451	-0.02018864	-0.01896773
29	-0.02707443	0.00121210	0.00699044	0.91802449	0.08733436
30	-0.02679025	-0.01840260	0.99288378	0.00664140	-0.00127233
31	0.97882272	0.00054450	-0.00089019	-0.00128913	0.00052958
32	0.00054450	0.97092943	-0.00103565	0.00008311	0.00017846
33	-0.00089019	-0.00103565	0.97039661	0.00137708	0.00007845
34	-0.00128913	0.00008311	0.00137708	3.73593746	0.91802449
35	0.00052958	0.00017846	0.00007845	0.91802449	3.88164137
36	-0.00001867	0.00007433	0.00009902	-0.02018864	1.44328111
37	0.00115229	0.00114319	0.00010197	-0.02825267	-0.04199350
38	0.00012599	0.00001867	0.00004012	-0.01313570	0.01036661
39	-0.00002056	0.00003021	0.00000195	0.00434028	0.01918628
40	-0.00000742	0.00000741	0.00002678	-0.00015168	0.03596847
41	0.00000570	0.00003186	0.00001585	0.00664140	-0.00401042
42	0.00013784	-0.00001555	0.00003118	0.00473356	1.31651443
43	0.00000441	0.00000053	0.00000088	0.00084494	0.01315369
44	-0.00000067	0.00000268	0.00000073	0.00008311	0.00121210
45	0.00000026	0.00000073	0.00000102	0.00137708	0.00699044
46	0.00004484	-0.00000067	0.00000026	-0.00128913	-0.02707443

	36	37	38	39	40
1	0.00005891	-0.00088756	-0.00000102	0.00001916	-0.00001316
2	-0.00080054	-0.00525694	0.00129912	-0.00009361	0.00018072
3	0.00139910	-0.00190807	-0.00012633	0.00008682	0.00020247
4	0.00048637	0.00045885	-0.00000735	0.00018072	0.00004367
5	0.00704168	0.04647578	0.00044541	-0.00080054	0.00048637
6	0.00091162	0.00789698	0.00025979	0.00089487	0.00111791
7	0.00004601	0.00356974	0.00031259	0.00030384	0.00009183
8	0.00025469	-0.00010059	0.00000344	-0.00000318	0.00003296
9	0.00000766	0.00001999	0.00003480	0.00000694	0.00000124
10	0.00001506	-0.00000477	0.00000178	0.00000176	0.00000880
11	0.01234856	0.08086246	0.00407237	0.00418314	-0.00014761
12	-0.01371423	0.00789698	0.00025979	-0.00563481	0.00275227
13	0.04958870	0.04647578	0.00044541	0.01358707	0.00067923
14	0.04647578	0.89292411	0.00363095	-0.00525694	0.00045885
15	0.00044541	0.00363095	-0.00656543	0.00129912	-0.00000735
16	0.01358707	-0.00525694	0.00129912	-0.00696576	0.00061967
17	0.00067923	0.00045885	-0.00000735	0.00061967	0.00063665
18	0.00100015	0.00356974	0.00031259	0.00007537	0.00014007

19	-0.00408623	-0.00190807	-0.00012633	0.00064081	-0.00014299
20	0.00058996	-0.00088756	-0.00000102	-0.00005519	0.00003452
21	0.00002516	0.00001999	0.00003480	-0.00001306	0.00002122
22	-0.00001621	-0.00000477	0.00000178	0.00007364	-0.00006068
23	0.00010956	-0.00010059	0.00000344	0.00006091	0.00005598
24	0.00073914	-0.00029473	0.00426426	0.00019792	0.00000051
25	0.01547059	-0.01391551	0.00155380	0.00485052	0.00025041
26	-0.00626007	0.01229565	0.00003076	0.00146208	0.00090898
27	-0.00159362	0.00555230	-0.00016399	0.00025041	0.00089180
28	0.19647926	0.92162708	0.01678728	0.01547059	-0.00159362
29	-0.01896773	-0.04199350	0.01036661	-0.00448802	0.00375577
30	0.00295640	0.00267591	0.00029159	0.00082600	0.00009249
31	-0.00001867	0.00115229	0.00012599	-0.00002056	-0.00000742
32	0.00007433	0.00114319	0.00001867	0.00003021	0.00000741
33	0.00009902	0.00010197	0.00004012	0.00000195	0.00002678
34	-0.02018864	-0.02825267	-0.01313570	0.00434028	-0.00015168
35	1.44328111	-0.04199350	0.01036661	0.01918628	0.03596847
36	4.08461129	0.92162708	0.01678728	1.34085434	0.01519785
37	0.92162708	3.72048300	0.89410202	-0.01391551	0.00555230
38	0.01678728	0.89410202	0.95317884	0.00155380	-0.00016399
39	1.34085434	-0.01391551	0.00155380	3.87803038	1.38345386
40	0.01519785	0.00555230	-0.00016399	1.38345386	3.87988863
41	0.04317160	0.00267591	0.00029159	0.05729742	1.39922537
42	0.02331889	0.01229565	0.00003076	0.08411331	0.06472916
43	-0.02466272	-0.00029473	0.00426426	0.99144912	-0.01867825
44	0.00716305	0.00114319	0.00001867	-0.02014679	0.99345132
45	0.00131451	0.00010197	0.00004012	0.00734518	-0.01877600
46	0.01131483	0.00115229	0.00012599	0.00070726	0.00855450

	41	42	43	44	45
1	0.00000268	0.00002648	0.00001176	0.00000029	-0.00000005
2	0.00030384	0.00008682	0.00001916	0.00000694	0.00000176
3	0.00012369	0.00112605	0.00002648	0.00000373	0.00002742
4	0.00009183	0.00020247	-0.00001316	0.00000124	0.00000880
5	0.00004601	0.00139910	0.00005891	0.00000766	0.00001506
6	0.00034477	0.00145095	-0.00002572	0.00000541	0.00001344
7	0.00013211	0.00012369	0.00000268	0.00000713	0.00000044
8	-0.00001954	0.00006253	0.00000377	0.00000004	0.00000161
9	0.00000713	0.00000373	0.00000029	-0.00000001	0.00000013
10	0.00000044	0.00002742	-0.00000005	0.00000013	0.00000042
11	0.00030395	-0.01550841	0.00085056	-0.00001248	0.00012586
12	-0.00002778	0.02063836	0.00010154	0.00000723	0.00011963
13	0.00100015	-0.00408623	0.00058996	0.00002516	-0.00001621
14	0.00356974	-0.00190807	-0.00088756	0.00001999	-0.00000477
15	0.00031259	-0.00012633	-0.00000102	0.00003480	0.00000178
16	0.00007537	0.00064081	-0.00005519	-0.00001306	0.00007364

17	0.00014007	-0.00014299	0.00003452	0.00002122	-0.00006068
18	0.00089973	0.00053969	0.00002696	-0.00002332	0.00005434
19	0.00053969	-0.00707966	0.00001748	0.00003095	-0.00009244
20	0.00002696	0.00001748	0.00007921	0.00000019	0.00000487
21	-0.00002332	0.00003095	0.00000019	0.00000490	0.00000411
22	0.00005434	-0.00009244	0.00000487	0.00000411	0.00000576
23	0.00005108	0.00033034	0.00000741	0.00000202	-0.00000006
24	0.00001192	-0.00003848	0.00000181	0.00000053	0.00000088
25	0.00082600	0.00146208	0.00019792	0.00003021	0.00000195
26	0.00029532	0.00433414	-0.00003848	-0.00001555	0.00003118
27	0.00009249	0.00090898	0.00000051	0.00000741	0.00002678
28	0.00295640	-0.00626007	0.00073914	0.00007433	0.00009902
29	-0.00127233	0.00911924	-0.00014239	0.00017846	0.00007845
30	0.00071565	0.00029532	0.00001192	0.00003186	0.00001585
31	0.00000570	0.00013784	0.00000441	-0.00000067	0.00000026
32	0.00003186	-0.00001555	0.00000053	0.00000268	0.00000073
33	0.00001585	0.00003118	0.00000088	0.00000073	0.00000102
34	0.00664140	0.00473356	0.00084494	0.00008311	0.00137708
35	-0.00401042	1.31651443	0.01315369	0.00121210	0.00699044
36	0.04317160	0.02331889	-0.02466272	0.00716305	0.00131451
37	0.00267591	0.01229565	-0.00029473	0.00114319	0.00010197
38	0.00029159	0.00003076	0.00426426	0.00001867	0.00004012
39	0.05729742	0.08411331	0.99144912	-0.02014679	0.00734518
40	1.39922537	0.06472916	-0.01867825	0.99345132	-0.01877600
41	3.84812553	1.37550093	0.00800642	-0.01840260	0.99288378
42	1.37550093	3.88353349	0.00072507	0.00736100	-0.02009104
43	0.00800642	0.00072507	0.97625069	-0.00090673	0.00059545
44	-0.01840260	0.00736100	-0.00090673	0.97092943	-0.00103565
45	0.99288378	-0.02009104	0.00059545	-0.00103565	0.97039661
46	-0.02679025	1.00656433	0.00012492	0.00054450	-0.00089019

	46
1	0.00000377
2	-0.00000318
3	0.00006253
4	0.00003296
5	0.00025469
6	0.00034101
7	-0.00001954
8	0.00007035
9	0.00000004
10	0.00000161
11	0.00328980
12	0.00033967
13	0.00010956
14	-0.00010059

15	0.00000344
16	0.00006091
17	0.00005598
18	0.00005108
19	0.00033034
20	0.00000741
21	0.00000202
22	-0.00000006
23	0.00020902
24	0.00000441
25	-0.00002056
26	0.00013784
27	-0.00000742
28	-0.00001867
29	0.00052958
30	0.00000570
31	0.00004484
32	-0.00000067
33	0.00000026
34	-0.00128913
35	-0.02707443
36	0.01131483
37	0.00115229
38	0.00012599
39	0.00070726
40	0.00855450
41	-0.02679025
42	1.00656433
43	0.00012492
44	0.00054450
45	-0.00089019
46	0.97882272

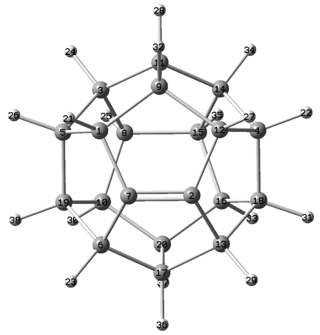

3 (Φ_p = 13.4º)

	1	2	3	4	5
1	3.91582789	-0.02609642	0.02817893	-0.00718485	0.95762322

2	-0.02609642	4.15482716	-0.00592738	0.06977058	0.02409930
3	0.02817893	-0.00592738	4.15751408	-0.00015298	0.99327454
4	-0.00718485	0.06977058	-0.00015298	4.20793417	0.00189667
5	0.95762322	0.02409930	0.99327454	0.00189667	4.20793412
6	0.01792845	-0.02609653	-0.00162306	-0.00727458	0.01077175
7	0.95014689	**1.89536094**	0.00401017	0.02409933	0.06977055
8	0.00128040	0.00486895	1.00669426	0.00526510	0.03988878
9	0.98521340	0.06518866	0.02883368	0.04881816	0.04881819
10	-0.00162306	-0.00592738	0.02900669	-0.00445120	0.03869758
11	0.02891606	0.02457549	1.01558815	0.04502424	0.04502425
12	0.02681375	0.95014684	0.00485825	0.95762315	-0.00718485
13	0.02706828	0.95014685	0.00724889	0.01077176	-0.00727459
14	0.00485825	0.00401015	0.02067720	0.99327453	-0.00015297
15	-0.00314526	0.00222872	0.03371419	0.03988881	0.00526506
16	0.00724888	0.00401018	0.00255147	0.03869756	-0.00445121
17	-0.00580932	0.06518865	-0.00395645	0.00751453	0.00751456
18	-0.00727458	0.06977057	-0.00445121	1.01975840	0.00667532
19	0.01077175	0.02409937	0.03869754	0.00667532	1.01975830
20	-0.01105876	0.02457549	0.00183378	0.00726683	0.00726683
21	0.92537612	0.00971496	0.00280845	0.00061179	-0.00293414
22	0.00009348	0.00075017	0.00005414	0.94012875	-0.00006029
23	0.00090363	0.00971497	0.00003060	-0.00009421	0.00109680
24	0.00257189	0.00006475	0.93828234	0.00040402	0.00134012
25	0.00017491	0.00028886	0.00003839	0.00016551	0.00255153
26	-0.00315710	0.00346156	0.00360638	-0.00006029	0.94012878
27	0.00144718	0.00155770	0.00021928	-0.00293414	0.00061179
28	0.00202976	0.00095076	0.00104892	0.00315649	0.00315648
29	0.00260907	0.00155768	0.00000790	0.00109680	-0.00009421
30	0.00007492	0.00006475	0.00258055	-0.00053351	0.00249040
31	-0.00015908	0.00075018	-0.00003069	-0.00038171	0.00015202
32	-0.00229916	0.00309572	0.00200954	0.00180582	0.00180581
33	0.00038362	0.00070013	0.00028967	0.00249039	-0.00053351
34	0.00007519	0.00070013	0.00251587	0.00134014	0.00040402
35	-0.00011469	-0.00004620	0.00247789	0.00255154	0.00016551
36	0.00052641	0.00309570	-0.00009321	0.00047603	0.00047603
37	-0.00056880	0.00095076	0.00008509	0.00027737	0.00027737
38	0.00200453	0.00346156	0.00252629	0.00015202	-0.00038171

	6	7	8	9	10
1	0.01792845	0.95014689	0.00128040	0.98521340	-0.00162306
2	-0.02609653	**1.89536094**	0.00486895	0.06518866	-0.00592738
3	-0.00162306	0.00401017	1.00669426	0.02883368	0.02900669
4	-0.00727458	0.02409933	0.00526510	0.04881816	-0.00445120
5	0.01077175	0.06977055	0.03988878	0.04881819	0.03869758
6	3.91582785	0.95014685	0.00128038	-0.00580925	0.02817892
7	0.95014685	4.15482700	0.00222874	0.06518859	0.00401014

8	0.00128038	0.00222874	4.22813202	0.00303167	1.00669432
9	-0.00580925	0.06518859	0.00303167	4.21850146	-0.00395645
10	0.02817892	0.00401014	1.00669432	-0.00395645	4.15751405
11	-0.01105876	0.02457546	0.03845030	0.99859189	0.00183382
12	0.02706825	-0.02609653	-0.00314526	0.98521340	0.00724889
13	0.02681376	-0.02609642	-0.00314527	-0.00580933	0.00485826
14	0.00724888	-0.00592737	0.03371421	0.02883370	0.00255142
15	-0.00314525	0.00486893	1.01763963	0.00303168	0.03371418
16	0.00485825	-0.00592737	0.03371418	-0.00395645	0.02067721
17	0.98521336	0.06518860	0.00303168	0.00684623	0.02883370
18	-0.00718484	0.02409928	0.00526506	0.00751455	-0.00015297
19	0.95762329	0.06977058	0.03988882	0.00751454	0.99327449
20	0.02891608	0.02457546	0.03845032	0.00759146	1.01558813
21	0.00090365	0.00155768	0.00037009	-0.00165800	0.00003060
22	-0.00015908	0.00346156	0.00029532	0.00242946	-0.00003069
23	0.92537615	0.00155771	0.00037008	-0.00024322	0.00280846
24	0.00007493	0.00070013	-0.00030221	0.00260756	0.00258056
25	0.00017491	-0.00004620	0.93845871	0.00022897	0.00003839
26	0.00200452	0.00075018	0.00210251	0.00242947	0.00252626
27	0.00260907	0.00971498	-0.00018483	-0.00165800	0.00000790
28	-0.00056880	0.00095075	0.00259974	0.00176126	0.00008509
29	0.00144720	0.00971496	-0.00018483	-0.00024322	0.00021928
30	0.00257189	0.00070013	-0.00030222	-0.00043035	0.93828234
31	0.00009348	0.00346156	0.00029532	0.00054670	0.00005414
32	0.00052642	0.00309570	0.00022933	0.93874157	-0.00009322
33	0.00007518	0.00006475	0.00245007	-0.00043035	0.00251589
34	0.00038362	0.00006475	0.00245010	0.00260755	0.00028966
35	-0.00011469	0.00028886	-0.00054299	0.00022898	0.00247787
36	-0.00229919	0.00309572	0.00022933	0.00002186	0.00200954
37	0.00202975	0.00095075	0.00259977	0.00031619	0.00104891
38	-0.00315709	0.00075017	0.00210249	0.00054669	0.00360638
	11	12	13	14	15
1	0.02891606	0.02681375	0.02706828	0.00485825	-0.00314526
2	0.02457549	0.95014684	0.95014685	0.00401015	0.00222872
3	1.01558815	0.00485825	0.00724889	0.02067720	0.03371419
4	0.04502424	0.95762315	0.01077176	0.99327453	0.03988881
5	0.04502425	-0.00718485	-0.00727459	-0.00015297	0.00526506
6	-0.01105876	0.02706825	0.02681376	0.00724888	-0.00314525
7	0.02457546	-0.02609653	-0.02609642	-0.00592737	0.00486893
8	0.03845030	-0.00314526	-0.00314527	0.03371421	1.01763963
9	0.99859189	0.98521340	-0.00580933	0.02883370	0.00303168
10	0.00183382	0.00724889	0.00485826	0.00255142	0.03371418
11	4.26862188	0.02891609	-0.01105876	1.01558812	0.03845030
12	0.02891609	3.91582772	0.01792847	0.02817891	0.00128038
13	-0.01105876	0.01792847	3.91582780	-0.00162307	0.00128041

14	1.01558812	0.02817891	-0.00162307	4.15751410	1.00669430
15	0.03845030	0.00128038	0.00128041	1.00669430	4.22813186
16	0.00183378	-0.00162306	0.02817891	0.02900669	1.00669423
17	0.00759146	-0.00580926	0.98521345	-0.00395645	0.00303167
18	0.00726683	0.01077174	0.95762312	0.03869760	0.03988873
19	0.00726683	-0.00727459	-0.00718486	-0.00445120	0.00526509
20	-0.00204664	-0.01105876	0.02891606	0.00183382	0.03845029
21	0.00335837	0.00144720	0.00260907	0.00021928	-0.00018483
22	0.00241672	-0.00315707	0.00200453	0.00360638	0.00210249
23	0.00001122	0.00260907	0.00144718	0.00000790	-0.00018483
24	0.00092990	0.00007518	0.00038362	0.00251589	0.00245007
25	0.00242417	-0.00011469	-0.00011469	0.00247787	-0.00054299
26	0.00241672	0.00009348	-0.00015908	0.00005414	0.00029532
27	0.00335836	0.92537615	0.00090364	0.00280846	0.00037008
28	0.94004498	0.00202975	-0.00056880	0.00104891	0.00259977
29	0.00001122	0.00090365	0.92537612	0.00003060	0.00037008
30	0.00028052	0.00038362	0.00007519	0.00028966	0.00245010
31	0.00008064	0.00200452	-0.00315709	0.00252626	0.00210251
32	0.00441545	-0.00229919	0.00052641	0.00200954	0.00022933
33	0.00028052	0.00007493	0.00257189	0.00258056	-0.00030220
34	0.00092989	0.00257189	0.00007492	0.93828235	-0.00030222
35	0.00242415	0.00017491	0.00017491	0.00003838	0.93845870
36	0.00009388	0.00052642	-0.00229917	-0.00009322	0.00022933
37	-0.00026437	-0.00056880	0.00202976	0.00008509	0.00259974
38	0.00008064	-0.00015908	0.00009348	-0.00003069	0.00029532
	16	17	18	19	20
1	0.00724888	-0.00580932	-0.00727458	0.01077175	-0.01105876
2	0.00401018	0.06518865	0.06977057	0.02409937	0.02457549
3	0.00255147	-0.00395645	-0.00445121	0.03869754	0.00183378
4	0.03869756	0.00751453	1.01975840	0.00667532	0.00726683
5	-0.00445121	0.00751456	0.00667532	1.01975830	0.00726683
6	0.00485825	0.98521336	-0.00718484	0.95762329	0.02891608
7	-0.00592737	0.06518860	0.02409928	0.06977058	0.02457546
8	0.03371418	0.00303168	0.00526506	0.03988882	0.03845032
9	-0.00395645	0.00684623	0.00751455	0.00751454	0.00759146
10	0.02067721	0.02883370	-0.00015297	0.99327449	1.01558813
11	0.00183378	0.00759146	0.00726683	0.00726683	-0.00204664
12	-0.00162306	-0.00580926	0.01077174	-0.00727459	-0.01105876
13	0.02817891	0.98521345	0.95762312	-0.00718486	0.02891606
14	0.02900669	-0.00395645	0.03869760	-0.00445120	0.00183382
15	1.00669423	0.00303167	0.03988873	0.00526509	0.03845029
16	4.15751411	0.02883369	0.99327449	-0.00015298	1.01558826
17	0.02883369	4.21850142	0.04881819	0.04881817	0.99859182
18	0.99327449	0.04881819	4.20793405	0.00189667	0.04502427
19	-0.00015298	0.04881817	0.00189667	4.20793419	0.04502423

20	1.01558826	0.99859182	0.04502427	0.04502423	4.26862195
21	0.00000790	-0.00024322	-0.00009421	0.00109680	0.00001122
22	0.00252629	0.00054669	-0.00038172	0.00015202	0.00008064
23	0.00021928	-0.00165799	0.00061179	-0.00293415	0.00335836
24	0.00028967	-0.00043035	-0.00053351	0.00249039	0.00028052
25	0.00247789	0.00022898	0.00016551	0.00255154	0.00242415
26	-0.00003069	0.00054670	0.00015202	-0.00038170	0.00008064
27	0.00003060	-0.00024322	0.00109680	-0.00009421	0.00001122
28	0.00008509	0.00031619	0.00027737	0.00027737	-0.00026437
29	0.00280845	-0.00165801	-0.00293414	0.00061179	0.00335837
30	0.00251587	0.00260756	0.00040402	0.00134014	0.00092989
31	0.00360638	0.00242946	0.94012878	-0.00006029	0.00241672
32	-0.00009322	0.00002186	0.00047603	0.00047603	0.00009388
33	0.93828234	0.00260756	0.00134013	0.00040402	0.00092989
34	0.00258055	-0.00043035	0.00249040	-0.00053351	0.00028052
35	0.00003840	0.00022897	0.00255153	0.00016551	0.00242417
36	0.00200954	0.93874158	0.00180581	0.00180582	0.00441546
37	0.00104891	0.00176127	0.00315648	0.00315649	0.94004498
38	0.00005414	0.00242946	-0.00006028	0.94012876	0.00241672

	21	22	23	24	25
1	0.92537612	0.00009348	0.00090363	0.00257189	0.00017491
2	0.00971496	0.00075017	0.00971497	0.00006475	0.00028886
3	0.00280845	0.00005414	0.00003060	0.93828234	0.00003839
4	0.00061179	0.94012875	-0.00009421	0.00040402	0.00016551
5	-0.00293414	-0.00006029	0.00109680	0.00134012	0.00255153
6	0.00090365	-0.00015908	0.92537615	0.00007493	0.00017491
7	0.00155768	0.00346156	0.00155771	0.00070013	-0.00004620
8	0.00037009	0.00029532	0.00037008	-0.00030221	0.93845871
9	-0.00165800	0.00242946	-0.00024322	0.00260756	0.00022897
10	0.00003060	-0.00003069	0.00280846	0.00258056	0.00003839
11	0.00335837	0.00241672	0.00001122	0.00092990	0.00242417
12	0.00144720	-0.00315707	0.00260907	0.00007518	-0.00011469
13	0.00260907	0.00200453	0.00144718	0.00038362	-0.00011469
14	0.00021928	0.00360638	0.00000790	0.00251589	0.00247787
15	-0.00018483	0.00210249	-0.00018483	0.00245007	-0.00054299
16	0.00000790	0.00252629	0.00021928	0.00028967	0.00247789
17	-0.00024322	0.00054669	-0.00165799	-0.00043035	0.00022898
18	-0.00009421	-0.00038172	0.00061179	-0.00053351	0.00016551
19	0.00109680	0.00015202	-0.00293415	0.00249039	0.00255154
20	0.00001122	0.00008064	0.00335836	0.00028052	0.00242415
21	0.94363795	0.00020166	0.00012379	0.00033549	0.00007934
22	0.00020166	0.95251284	0.00001869	0.00004864	0.00012762
23	0.00012379	0.00001869	0.94363800	0.00011604	0.00007934
24	0.00033549	0.00004864	0.00011604	0.95262677	-0.00240622
25	0.00007934	0.00012762	0.00007934	-0.00240622	0.94903486

26	-0.00199253	0.00001691	0.00018898	-0.00180883	0.00035988
27	0.00005208	-0.00199252	0.00052653	0.00010386	0.00003715
28	0.00038214	0.00025895	0.00004134	-0.00234558	0.00026165
29	0.00052653	0.00018898	0.00005208	-0.00000477	0.00003715
30	0.00011604	0.00008640	0.00033549	0.00033071	-0.00240622
31	0.00001869	-0.00248968	0.00020166	0.00008640	0.00012762
32	-0.00172469	0.00009350	0.00014411	0.00028032	0.00008254
33	-0.00000477	0.00025579	0.00010386	0.00010482	0.00024592
34	0.00010386	-0.00180883	-0.00000477	0.00037153	0.00024592
35	0.00003715	0.00035988	0.00003715	0.00024592	-0.00259260
36	0.00014411	0.00012495	-0.00172469	0.00004972	0.00008254
37	0.00004134	0.00013800	0.00038214	0.00008749	0.00026165
38	0.00018898	0.00002412	-0.00199252	0.00025579	0.00035988
	26	27	28	29	30
1	-0.00315710	0.00144718	0.00202976	0.00260907	0.00007492
2	0.00346156	0.00155770	0.00095076	0.00155768	0.00006475
3	0.00360638	0.00021928	0.00104892	0.00000790	0.00258055
4	-0.00006029	-0.00293414	0.00315649	0.00109680	-0.00053351
5	0.94012878	0.00061179	0.00315648	-0.00009421	0.00249040
6	0.00200452	0.00260907	-0.00056880	0.00144720	0.00257189
7	0.00075018	0.00971498	0.00095075	0.00971496	0.00070013
8	0.00210251	-0.00018483	0.00259974	-0.00018483	-0.00030222
9	0.00242947	-0.00165800	0.00176126	-0.00024322	-0.00043035
10	0.00252626	0.00000790	0.00008509	0.00021928	0.93828234
11	0.00241672	0.00335836	0.94004498	0.00001122	0.00028052
12	0.00009348	0.92537615	0.00202975	0.00090365	0.00038362
13	-0.00015908	0.00090364	-0.00056880	0.92537612	0.00007519
14	0.00005414	0.00280846	0.00104891	0.00003060	0.00028966
15	0.00029532	0.00037008	0.00259977	0.00037008	0.00245010
16	-0.00003069	0.00003060	0.00008509	0.00280845	0.00251587
17	0.00054670	-0.00024322	0.00031619	-0.00165801	0.00260756
18	0.00015202	0.00109680	0.00027737	-0.00293414	0.00040402
19	-0.00038170	-0.00009421	0.00027737	0.00061179	0.00134014
20	0.00008064	0.00001122	-0.00026437	0.00335837	0.00092989
21	-0.00199253	0.00005208	0.00038214	0.00052653	0.00011604
22	0.00001691	-0.00199252	0.00025895	0.00018898	0.00008640
23	0.00018898	0.00052653	0.00004134	0.00005208	0.00033549
24	-0.00180883	0.00010386	-0.00234558	-0.00000477	0.00033071
25	0.00035988	0.00003715	0.00026165	0.00003715	-0.00240622
26	0.95251287	0.00020166	0.00025895	0.00001869	0.00025579
27	0.00020166	0.94363799	0.00038214	0.00012379	-0.00000477
28	0.00025895	0.00038214	0.95708998	0.00004134	0.00008749
29	0.00001869	0.00012379	0.00004134	0.94363795	0.00010386
30	0.00025579	-0.00000477	0.00008749	0.00010386	0.95262677
31	0.00002412	0.00018898	0.00013800	-0.00199253	0.00004864

32	0.00009350	-0.00172469	-0.00162800	0.00014411	0.00004972
33	0.00008640	0.00011603	0.00008749	0.00033549	0.00037153
34	0.00004864	0.00033549	-0.00234558	0.00011604	0.00010482
35	0.00012762	0.00007934	0.00026165	0.00007934	0.00024592
36	0.00012495	0.00014411	-0.00000304	-0.00172469	0.00028032
37	0.00013800	0.00004134	0.00005633	0.00038214	-0.00234558
38	-0.00248968	0.00001869	0.00013800	0.00020166	-0.00180883
	31	32	33	34	35
1	-0.00015908	-0.00229916	0.00038362	0.00007519	-0.00011469
2	0.00075018	0.00309572	0.00070013	0.00070013	-0.00004620
3	-0.00003069	0.00200954	0.00028967	0.00251587	0.00247789
4	-0.00038171	0.00180582	0.00249039	0.00134014	0.00255154
5	0.00015202	0.00180581	-0.00053351	0.00040402	0.00016551
6	0.00009348	0.00052642	0.00007518	0.00038362	-0.00011469
7	0.00346156	0.00309570	0.00006475	0.00006475	0.00028886
8	0.00029532	0.00022933	0.00245007	0.00245010	-0.00054299
9	0.00054670	0.93874157	-0.00043035	0.00260755	0.00022898
10	0.00005414	-0.00009322	0.00251589	0.00028966	0.00247787
11	0.00008064	0.00441545	0.00028052	0.00092989	0.00242415
12	0.00200452	-0.00229919	0.00007493	0.00257189	0.00017491
13	-0.00315709	0.00052641	0.00257189	0.00007492	0.00017491
14	0.00252626	0.00200954	0.00258056	0.93828235	0.00003838
15	0.00210251	0.00022933	-0.00030220	-0.00030222	0.93845870
16	0.00360638	-0.00009322	0.93828234	0.00258055	0.00003840
17	0.00242946	0.00002186	0.00260756	-0.00043035	0.00022897
18	0.94012878	0.00047603	0.00134013	0.00249040	0.00255153
19	-0.00006029	0.00047603	0.00040402	-0.00053351	0.00016551
20	0.00241672	0.00009388	0.00092989	0.00028052	0.00242417
21	0.00001869	-0.00172469	-0.00000477	0.00010386	0.00003715
22	-0.00248968	0.00009350	0.00025579	-0.00180883	0.00035988
23	0.00020166	0.00014411	0.00010386	-0.00000477	0.00003715
24	0.00008640	0.00028032	0.00010482	0.00037153	0.00024592
25	0.00012762	0.00008254	0.00024592	0.00024592	-0.00259260
26	0.00002412	0.00009350	0.00008640	0.00004864	0.00012762
27	0.00018898	-0.00172469	0.00011603	0.00033549	0.00007934
28	0.00013800	-0.00162800	0.00008749	-0.00234558	0.00026165
29	-0.00199253	0.00014411	0.00033549	0.00011604	0.00007934
30	0.00004864	0.00004972	0.00037153	0.00010482	0.00024592
31	0.95251286	0.00012495	-0.00180883	0.00025579	0.00035988
32	0.00012495	0.95125172	0.00004972	0.00028032	0.00008254
33	-0.00180883	0.00004972	0.95262677	0.00033071	-0.00240622
34	0.00025579	0.00028032	0.00033071	0.95262678	-0.00240622
35	0.00035988	0.00008254	-0.00240622	-0.00240622	0.94903485
36	0.00009350	0.00000823	0.00028032	0.00004972	0.00008254
37	0.00025895	-0.00000304	-0.00234558	0.00008749	0.00026165

38	0.00001691	0.00012495	0.00004864	0.00008640	0.00012762

	36	37	38
1	0.00052641	-0.00056880	0.00200453
2	0.00309570	0.00095076	0.00346156
3	-0.00009321	0.00008509	0.00252629
4	0.00047603	0.00027737	0.00015202
5	0.00047603	0.00027737	-0.00038171
6	-0.00229919	0.00202975	-0.00315709
7	0.00309572	0.00095075	0.00075017
8	0.00022933	0.00259977	0.00210249
9	0.00002186	0.00031619	0.00054669
10	0.00200954	0.00104891	0.00360638
11	0.00009388	-0.00026437	0.00008064
12	0.00052642	-0.00056880	-0.00015908
13	-0.00229917	0.00202976	0.00009348
14	-0.00009322	0.00008509	-0.00003069
15	0.00022933	0.00259974	0.00029532
16	0.00200954	0.00104891	0.00005414
17	0.93874158	0.00176127	0.00242946
18	0.00180581	0.00315648	-0.00006028
19	0.00180582	0.00315649	0.94012876
20	0.00441546	0.94004498	0.00241672
21	0.00014411	0.00004134	0.00018898
22	0.00012495	0.00013800	0.00002412
23	-0.00172469	0.00038214	-0.00199252
24	0.00004972	0.00008749	0.00025579
25	0.00008254	0.00026165	0.00035988
26	0.00012495	0.00013800	-0.00248968
27	0.00014411	0.00004134	0.00001869
28	-0.00000304	0.00005633	0.00013800
29	-0.00172469	0.00038214	0.00020166
30	0.00028032	-0.00234558	-0.00180883
31	0.00009350	0.00025895	0.00001691
32	0.00000823	-0.00000304	0.00012495
33	0.00028032	-0.00234558	0.00004864
34	0.00004972	0.00008749	0.00008640
35	0.00008254	0.00026165	0.00012762
36	0.95125172	-0.00162800	0.00009350
37	-0.00162800	0.95708999	0.00025895
38	0.00009350	0.00025895	0.95251285

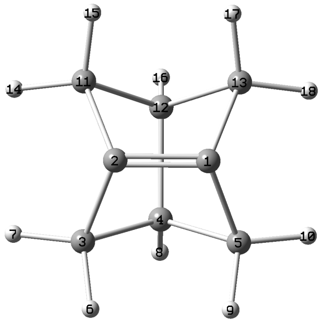

4 (Φ_p = 23.3º)

	1	2	3	4	5
1	3.83141539	**1.67838308**	0.03616255	0.01207911	0.99820371
2	**1.67838308**	3.83141539	0.99820371	0.01207911	0.03616255
3	0.03616255	0.99820371	4.04679988	0.98744746	0.02594485
4	0.01207911	0.01207911	0.98744746	3.88917441	0.98744746
5	0.99820371	0.03616255	0.02594485	0.98744746	4.04679988
6	-0.00329043	0.00907290	0.95142567	0.00325690	0.00542562
7	0.00468007	0.00386372	0.96189625	-0.00100618	0.00435050
8	0.01574453	0.01574453	-0.00064126	0.94846629	-0.00064126
9	0.00907290	-0.00329043	0.00542562	0.00325690	0.95142567
10	0.00386372	0.00468007	0.00435050	-0.00100618	0.96189625
11	0.03616255	0.99820371	0.04425656	0.00770855	0.01410754
12	0.01207911	0.01207911	0.00770855	0.91874419	0.00770855
13	0.99820371	0.03616255	0.01410754	0.00770855	0.04425656
14	0.00468007	0.00386372	0.00368381	-0.00400138	0.00054242
15	-0.00329043	0.00907290	0.00455870	0.00620086	0.00017204
16	0.01574453	0.01574453	0.00155492	-0.00140670	0.00155492
17	0.00907290	-0.00329043	0.00017204	0.00620086	0.00455870
18	0.00386372	0.00468007	0.00054242	-0.00400138	0.00368381

	6	7	8	9	10
1	-0.00329043	0.00468007	0.01574453	0.00907290	0.00386372
2	0.00907290	0.00386372	0.01574453	-0.00329043	0.00468007
3	0.95142567	0.96189625	-0.00064126	0.00542562	0.00435050
4	0.00325690	-0.00100618	0.94846629	0.00325690	-0.00100618
5	0.00542562	0.00435050	-0.00064126	0.95142567	0.96189625
6	0.98018876	-0.00032427	0.00093925	0.00012250	0.00069605
7	-0.00032427	0.97634581	-0.00027609	0.00069605	0.00153403
8	0.00093925	-0.00027609	0.98291947	0.00093925	-0.00027609
9	0.00012250	0.00069605	0.00093925	0.98018876	-0.00032427
10	0.00069605	0.00153403	-0.00027609	-0.00032427	0.97634581
11	0.00455870	0.00368381	0.00155492	0.00017204	0.00054242
12	0.00620086	-0.00400138	-0.00140670	0.00620086	-0.00400138
13	0.00017204	0.00054242	0.00155492	0.00455870	0.00368381
14	0.00037045	0.00012741	0.00007085	0.00004692	0.00009124

15	0.00108190	0.00037045	0.00030909	0.00012460	0.00004692
16	0.00030909	0.00007085	0.00045729	0.00030909	0.00007085
17	0.00012460	0.00004692	0.00030909	0.00108190	0.00037045
18	0.00004692	0.00009124	0.00007085	0.00037045	0.00012741

	11	12	13	14	15
1	0.03616255	0.01207911	0.99820371	0.00468007	-0.00329043
2	0.99820371	0.01207911	0.03616255	0.00386372	0.00907290
3	0.04425656	0.00770855	0.01410754	0.00368381	0.00455870
4	0.00770855	0.91874419	0.00770855	-0.00400138	0.00620086
5	0.01410754	0.00770855	0.04425656	0.00054242	0.00017204
6	0.00455870	0.00620086	0.00017204	0.00037045	0.00108190
7	0.00368381	-0.00400138	0.00054242	0.00012741	0.00037045
8	0.00155492	-0.00140670	0.00155492	0.00007085	0.00030909
9	0.00017204	0.00620086	0.00455870	0.00004692	0.00012460
10	0.00054242	-0.00400138	0.00368381	0.00009124	0.00004692
11	4.04679988	0.98744746	0.02594485	0.96189625	0.95142567
12	0.98744746	3.88917441	0.98744746	-0.00100618	0.00325690
13	0.02594485	0.98744746	4.04679988	0.00435050	0.00542562
14	0.96189625	-0.00100618	0.00435050	0.97634581	-0.00032427
15	0.95142567	0.00325690	0.00542562	-0.00032427	0.98018876
16	-0.00064126	0.94846629	-0.00064126	-0.00027609	0.00093925
17	0.00542562	0.00325690	0.95142567	0.00069605	0.00012250
18	0.00435050	-0.00100618	0.96189625	0.00153403	0.00069605

	16	17	18
1	0.01574453	0.00907290	0.00386372
2	0.01574453	-0.00329043	0.00468007
3	0.00155492	0.00017204	0.00054242
4	-0.00140670	0.00620086	-0.00400138
5	0.00155492	0.00455870	0.00368381
6	0.00030909	0.00012460	0.00004692
7	0.00007085	0.00004692	0.00009124
8	0.00045729	0.00030909	0.00007085
9	0.00030909	0.00108190	0.00037045
10	0.00007085	0.00037045	0.00012741
11	-0.00064126	0.00542562	0.00435050
12	0.94846629	0.00325690	-0.00100618
13	-0.00064126	0.95142567	0.96189625
14	-0.00027609	0.00069605	0.00153403
15	0.00093925	0.00012250	0.00069605
16	0.98291947	0.00093925	-0.00027609
17	0.00093925	0.98018876	-0.00032427
18	-0.00027609	-0.00032427	0.97634581

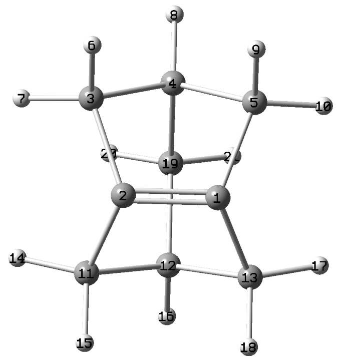

5 (Φ_p = 18.3º)

	1	2	3	4	5
1	3.74494924	**1.69580915**	0.01293159	-0.00137956	1.00256137
2	**1.69580915**	3.74494924	1.00256137	-0.00137956	0.01293159
3	0.01293159	1.00256137	4.08673948	0.97491718	0.04315340
4	-0.00137956	-0.00137956	0.97491718	3.84831472	0.97491718
5	1.00256137	0.01293159	0.04315340	0.97491718	4.08673948
6	0.00208209	0.00779927	0.94677435	0.00136089	0.00464512
7	0.00415308	-0.00029817	0.96245806	-0.00425566	0.00463478
8	0.01031855	0.01031855	-0.00632918	0.96941282	-0.00632918
9	0.00779927	0.00208209	0.00464512	0.00136089	0.94677435
10	-0.00029817	0.00415308	0.00463478	-0.00425566	0.96245806
11	0.01293159	1.00256137	0.06413027	-0.01458038	0.02059675
12	-0.00137956	-0.00137956	-0.01458038	0.00495503	-0.01458038
13	1.00256137	0.01293159	0.02059675	-0.01458038	0.06413027
14	0.00415308	-0.00029817	0.00499523	-0.00481125	0.00075226
15	0.00208209	0.00779927	0.00461752	0.00004827	0.00079560
16	0.01031855	0.01031855	0.00195421	0.00340186	0.00195421
17	-0.00029817	0.00415308	0.00075226	-0.00481125	0.00499523
18	0.00779927	0.00208209	0.00079560	0.00004827	0.00461752
19	-0.02867886	-0.02867886	0.05146718	0.97951555	0.05146718
20	0.00347125	-0.00198873	0.00291326	-0.00578475	0.00335090
21	-0.00198873	0.00347125	0.00335090	-0.00578475	0.00291326

	6	7	8	9	10
1	0.00208209	0.00415308	0.01031855	0.00779927	-0.00029817
2	0.00779927	-0.00029817	0.01031855	0.00208209	0.00415308
3	0.94677435	0.96245806	-0.00632918	0.00464512	0.00463478
4	0.00136089	-0.00425566	0.96941282	0.00136089	-0.00425566
5	0.00464512	0.00463478	-0.00632918	0.94677435	0.96245806
6	0.97778486	0.00000241	0.00014797	0.00160559	0.00045322
7	0.00000241	0.97594833	-0.00013959	0.00045322	0.00114100
8	0.00014797	-0.00013959	0.97536729	0.00014797	-0.00013959
9	0.00160559	0.00045322	0.00014797	0.97778486	0.00000241
10	0.00045322	0.00114100	-0.00013959	0.00000241	0.97594833

11	0.00461752	0.00499523	0.00195421	0.00079560	0.00075226
12	0.00004827	-0.00481125	0.00340186	0.00004827	-0.00481125
13	0.00079560	0.00075226	0.00195421	0.00461752	0.00499523
14	0.00005898	0.00101836	0.00001564	0.00002489	0.00012958
15	0.00062342	0.00005898	0.00034229	0.00013947	0.00002489
16	0.00034229	0.00001564	0.00020298	0.00034229	0.00001564
17	0.00002489	0.00012958	0.00001564	0.00005898	0.00101836
18	0.00013947	0.00002489	0.00034229	0.00062342	0.00005898
19	0.00518846	0.00429383	-0.00759528	0.00518846	0.00429383
20	0.00019643	0.00095713	-0.00133742	0.00087862	0.00036455
21	0.00087862	0.00036455	-0.00133742	0.00019643	0.00095713

	11	12	13	14	15
1	0.01293159	-0.00137956	1.00256137	0.00415308	0.00208209
2	1.00256137	-0.00137956	0.01293159	-0.00029817	0.00779927
3	0.06413027	-0.01458038	0.02059675	0.00499523	0.00461752
4	-0.01458038	0.00495503	-0.01458038	-0.00481125	0.00004827
5	0.02059675	-0.01458038	0.06413027	0.00075226	0.00079560
6	0.00461752	0.00004827	0.00079560	0.00005898	0.00062342
7	0.00499523	-0.00481125	0.00075226	0.00101836	0.00005898
8	0.00195421	0.00340186	0.00195421	0.00001564	0.00034229
9	0.00079560	0.00004827	0.00461752	0.00002489	0.00013947
10	0.00075226	-0.00481125	0.00499523	0.00012958	0.00002489
11	4.08673948	0.97491718	0.04315340	0.96245806	0.94677435
12	0.97491718	3.84831472	0.97491718	-0.00425566	0.00136089
13	0.04315340	0.97491718	4.08673948	0.00463478	0.00464512
14	0.96245806	-0.00425566	0.00463478	0.97594833	0.00000241
15	0.94677435	0.00136089	0.00464512	0.00000241	0.97778486
16	-0.00632918	0.96941282	-0.00632918	-0.00013959	0.00014797
17	0.00463478	-0.00425566	0.96245806	0.00114100	0.00045322
18	0.00464512	0.00136089	0.94677435	0.00045322	0.00160559
19	0.05146718	0.97951555	0.05146718	0.00429383	0.00518846
20	0.00291326	-0.00578475	0.00335090	0.00095713	0.00019643
21	0.00335090	-0.00578475	0.00291326	0.00036455	0.00087862

	16	17	18	19	20
1	0.01031855	-0.00029817	0.00779927	-0.02867886	0.00347125
2	0.01031855	0.00415308	0.00208209	-0.02867886	-0.00198873
3	0.00195421	0.00075226	0.00079560	0.05146718	0.00291326
4	0.00340186	-0.00481125	0.00004827	0.97951555	-0.00578475
5	0.00195421	0.00499523	0.00461752	0.05146718	0.00335090
6	0.00034229	0.00002489	0.00013947	0.00518846	0.00019643
7	0.00001564	0.00012958	0.00002489	0.00429383	0.00095713
8	0.00020298	0.00001564	0.00034229	-0.00759528	-0.00133742
9	0.00034229	0.00005898	0.00062342	0.00518846	0.00087862
10	0.00001564	0.00101836	0.00005898	0.00429383	0.00036455
11	-0.00632918	0.00463478	0.00464512	0.05146718	0.00291326

12	0.96941282	-0.00425566	0.00136089	0.97951555	-0.00578475
13	-0.00632918	0.96245806	0.94677435	0.05146718	0.00335090
14	-0.00013959	0.00114100	0.00045322	0.00429383	0.00095713
15	0.00014797	0.00045322	0.00160559	0.00518846	0.00019643
16	0.97536729	-0.00013959	0.00014797	-0.00759528	-0.00133742
17	-0.00013959	0.97594833	0.00000241	0.00429383	0.00036455
18	0.00014797	0.00000241	0.97778486	0.00518846	0.00087862
19	-0.00759528	0.00429383	0.00518846	4.06611876	0.96791901
20	-0.00133742	0.00036455	0.00087862	0.96791901	0.97067097
21	-0.00133742	0.00095713	0.00019643	0.96791901	-0.00180800

	21
1	-0.00198873
2	0.00347125
3	0.00335090
4	-0.00578475
5	0.00291326
6	0.00087862
7	0.00036455
8	-0.00133742
9	0.00019643
10	0.00095713
11	0.00335090
12	-0.00578475
13	0.00291326
14	0.00036455
15	0.00087862
16	-0.00133742
17	0.00095713
18	0.00019643
19	0.96791901
20	-0.00180800
21	0.97067097

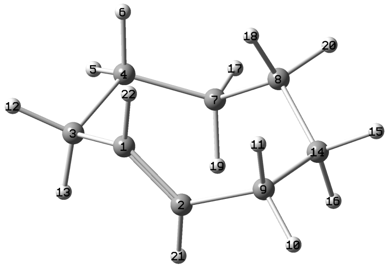

6 (Φ_p = 6.5º)

	1	2	3	4	5
1	3.91057252	**1.90597890**	0.96909425	0.00706484	0.00435919
2	**1.90597890**	3.91057253	0.04353775	0.00565871	0.00502219
3	0.96909425	0.04353775	3.93262601	0.97085362	-0.00540583

4	0.00706484	0.00565871	0.97085362	3.94488654	0.97194589
5	0.00435919	0.00502219	-0.00540583	0.97194589	0.97088772
6	0.00189869	0.00142450	-0.00968724	0.97855395	-0.00302480
7	-0.02137385	0.01815574	0.00856409	0.97180746	-0.00337961
8	0.01815627	-0.02137349	-0.00083566	0.03092823	0.00401149
9	0.04353801	0.96909380	0.00856066	0.00165106	0.00031989
10	0.00818945	-0.00442316	0.00179920	-0.00022265	-0.00002320
11	0.01130474	-0.01737881	0.00007244	-0.00006376	-0.00000501
12	-0.00442318	0.00818945	0.97425616	-0.00808333	-0.00103758
13	-0.01737892	0.01130473	0.97190069	-0.00162931	-0.00134045
14	0.00565857	0.00706474	0.00165108	0.00463799	0.00076509
15	0.00502217	0.00435917	0.00031989	0.00076510	0.00042243
16	0.00142449	0.00189876	-0.00004635	0.00068344	0.00006122
17	0.00023049	0.00197811	0.00518400	-0.00357671	-0.00260912
18	0.01157590	-0.00413008	-0.00203287	0.01254728	0.00035936
19	-0.00413019	0.01157618	0.00996297	-0.00684263	-0.00053851
20	0.00197810	0.00023049	0.00033266	0.00245053	0.00068070
21	-0.00835755	0.97076236	0.00146853	0.00025588	0.00011065
22	0.97076218	-0.00835753	-0.01692403	0.00550096	0.00019372

	6	7	8	9	10
1	0.00189869	-0.02137385	0.01815627	0.04353801	0.00818945
2	0.00142450	0.01815574	-0.02137349	0.96909380	-0.00442316
3	-0.00968724	0.00856409	-0.00083566	0.00856066	0.00179920
4	0.97855395	0.97180746	0.03092823	0.00165106	-0.00022265
5	-0.00302480	-0.00337961	0.00401149	0.00031989	-0.00002320
6	0.96942662	-0.01112819	-0.00252674	-0.00004635	0.00007762
7	-0.01112819	3.86306045	0.95358278	-0.00083567	0.00009284
8	-0.00252674	0.95358278	3.86306222	0.00856395	0.00563528
9	-0.00004635	-0.00083567	0.00856395	3.93262468	0.97425615
10	0.00007762	0.00009284	0.00563528	0.97425615	0.97620678
11	0.00007569	0.00010395	-0.00481376	0.97190067	-0.00039726
12	-0.00140837	0.00563532	0.00009284	0.00179920	0.00026833
13	0.00577567	-0.00481384	0.00010396	0.00007243	0.00000545
14	0.00068344	0.03092799	0.97180794	0.97085307	-0.00808333
15	0.00006122	0.00401150	-0.00337963	-0.00540582	-0.00103758
16	0.00006179	-0.00252680	-0.01112818	-0.00968736	-0.00140837
17	-0.00033642	0.97164223	-0.00520839	0.00033265	0.00007033
18	0.00225091	-0.01923113	0.97076790	0.00996280	0.00050118
19	0.00639677	0.97076789	-0.01923117	-0.00203291	0.00006646
20	0.00007926	-0.00520836	0.97164222	0.00518401	0.00059869
21	0.00003827	-0.00046061	-0.00327317	-0.01692409	-0.00030543
22	0.00020697	-0.00327326	-0.00046045	0.00146853	0.00054680

	11	12	13	14	15
1	0.01130474	-0.00442318	-0.01737892	0.00565857	0.00502217
2	-0.01737881	0.00818945	0.01130473	0.00706474	0.00435917

3	0.00007244	0.97425616	0.97190069	0.00165108	0.00031989
4	-0.00006376	-0.00808333	-0.00162931	0.00463799	0.00076510
5	-0.00000501	-0.00103758	-0.00134045	0.00076509	0.00042243
6	0.00007569	-0.00140837	0.00577567	0.00068344	0.00006122
7	0.00010395	0.00563532	-0.00481384	0.03092799	0.00401150
8	-0.00481376	0.00009284	0.00010396	0.97180794	-0.00337963
9	0.97190067	0.00179920	0.00007243	0.97085307	-0.00540582
10	-0.00039726	0.00026833	0.00000545	-0.00808333	-0.00103758
11	0.97540207	0.00000545	0.00020536	-0.00162939	-0.00134045
12	0.00000545	0.97620673	-0.00039734	-0.00022265	-0.00002320
13	0.00020536	-0.00039734	0.97540192	-0.00006376	-0.00000501
14	-0.00162939	-0.00022265	-0.00006376	3.94488605	0.97194588
15	-0.00134045	-0.00002320	-0.00000501	0.97194588	0.97088765
16	0.00577564	0.00007762	0.00007569	0.97855403	-0.00302479
17	0.00000559	0.00059869	0.00007949	0.00245053	0.00068070
18	0.00118700	0.00006646	0.00036253	-0.00684256	-0.00053853
19	0.00036253	0.00050118	0.00118701	0.01254736	0.00035936
20	0.00007950	0.00007033	0.00000559	-0.00357667	-0.00260912
21	0.00722283	0.00054680	0.00272908	0.00550088	0.00019372
22	0.00272912	-0.00030543	0.00722287	0.00025584	0.00011065
	16	17	18	19	20
1	0.00142449	0.00023049	0.01157590	-0.00413019	0.00197810
2	0.00189876	0.00197811	-0.00413008	0.01157618	0.00023049
3	-0.00004635	0.00518400	-0.00203287	0.00996297	0.00033266
4	0.00068344	-0.00357671	0.01254728	-0.00684263	0.00245053
5	0.00006122	-0.00260912	0.00035936	-0.00053851	0.00068070
6	0.00006179	-0.00033642	0.00225091	0.00639677	0.00007926
7	-0.00252680	0.97164223	-0.01923113	0.97076789	-0.00520836
8	-0.01112818	-0.00520839	0.97076790	-0.01923117	0.97164222
9	-0.00968736	0.00033265	0.00996280	-0.00203291	0.00518401
10	-0.00140837	0.00007033	0.00050118	0.00006646	0.00059869
11	0.00577564	0.00000559	0.00118700	0.00036253	0.00007950
12	0.00007762	0.00059869	0.00006646	0.00050118	0.00007033
13	0.00007569	0.00007949	0.00036253	0.00118701	0.00000559
14	0.97855403	0.00245053	-0.00684256	0.01254736	-0.00357667
15	-0.00302479	0.00068070	-0.00053853	0.00035936	-0.00260912
16	0.96942653	0.00007926	0.00639678	0.00225089	-0.00033645
17	0.00007926	0.96574277	0.00238897	-0.00545076	-0.00276607
18	0.00639678	0.00238897	0.98767274	0.00539191	-0.00545074
19	0.00225089	-0.00545076	0.00539191	0.98767306	0.00238896
20	-0.00033645	-0.00276607	-0.00545074	0.00238896	0.96574280
21	0.00020696	-0.00002255	0.00087985	0.00125988	-0.00000827
22	0.00003827	-0.00000827	0.00125983	0.00087987	-0.00002255
	21	22			
1	-0.00835755	0.97076218			

2	0.97076236	-0.00835753
3	0.00146853	-0.01692403
4	0.00025588	0.00550096
5	0.00011065	0.00019372
6	0.00003827	0.00020697
7	-0.00046061	-0.00327326
8	-0.00327317	-0.00046045
9	-0.01692409	0.00146853
10	-0.00030543	0.00054680
11	0.00722283	0.00272912
12	0.00054680	-0.00030543
13	0.00272908	0.00722287
14	0.00550088	0.00025584
15	0.00019372	0.00011065
16	0.00020696	0.00003827
17	-0.00002255	-0.00000827
18	0.00087985	0.00125983
19	0.00125988	0.00087987
20	-0.00000827	-0.00002255
21	0.97345007	0.01162606
22	0.01162606	0.97345015

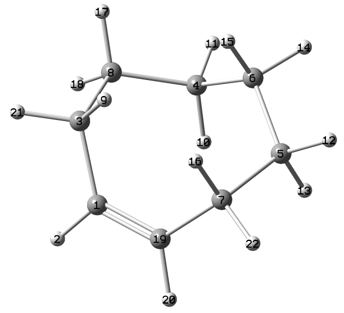

cis-**6**

	1	2	3	4	5
1	3.93153330	0.96381378	0.94330419	-0.00622785	0.00654429
2	0.96381378	0.97266491	-0.01026142	-0.00065225	0.00044869
3	0.94330419	-0.01026142	3.84085866	-0.02736959	-0.00228640
4	-0.00622785	-0.00065225	-0.02736959	3.80814930	0.02515799
5	0.00654429	0.00044869	-0.00228640	0.02515799	3.91812210
6	0.01294941	0.00018465	0.01449636	0.93356401	0.99080943
7	0.01958677	0.00921918	0.00796661	-0.00356278	0.94758221
8	0.02143776	0.00267514	0.96287161	0.99117250	0.00312237
9	-0.02158507	0.00577285	0.97744082	-0.00752792	-0.00576199
10	0.00788579	0.00027236	-0.00773580	0.96354753	0.01293646
11	0.00161089	0.00013090	0.00294037	0.97668285	0.00277782
12	0.00329288	0.00012414	0.00039350	0.00434654	0.97311666
13	0.00216759	0.00009635	0.00011447	-0.00380851	0.97566337

14	0.00050931	-0.00000381	0.00068982	-0.00248765	-0.00291870
15	0.00100933	-0.00005005	-0.00570547	-0.02599093	-0.00989476
16	0.01707647	0.00026451	-0.00464349	-0.00186735	-0.01319599
17	0.00448498	0.00028143	-0.00834499	-0.00010644	-0.00003258
18	0.00012168	0.00039095	-0.00189108	-0.00572241	0.00064206
19	**1.95586601**	0.00069811	0.01050672	-0.00421434	0.01683437
20	0.00021661	-0.00079367	0.00911947	0.00016354	0.00109768
21	-0.00660304	-0.00074900	0.97829776	0.00323394	0.00019266
22	0.00407151	0.00080206	0.00095518	-0.00018157	-0.00471356

	6	7	8	9	10
1	0.01294941	0.01958677	0.02143776	-0.02158507	0.00788579
2	0.00018465	0.00921918	0.00267514	0.00577285	0.00027236
3	0.01449636	0.00796661	0.96287161	0.97744082	-0.00773580
4	0.93356401	-0.00356278	0.99117250	-0.00752792	0.96354753
5	0.99080943	0.94758221	0.00312237	-0.00576199	0.01293646
6	3.95660601	0.01961408	0.02961253	0.01045284	-0.01394059
7	0.01961408	3.90358440	0.00122848	0.00919316	-0.00267565
8	0.02961253	0.00122848	3.98373481	-0.00202013	0.00696135
9	0.01045284	0.00919316	-0.00202013	0.98975812	0.00149897
10	-0.01394059	-0.00267565	0.00696135	0.00149897	0.98237993
11	-0.00571209	0.00064988	-0.00616545	0.00009385	-0.00551904
12	-0.00511875	-0.00333828	0.00021133	0.00025407	0.00061047
13	-0.00364595	-0.01250555	0.00061710	0.00003173	0.00234004
14	0.97115764	0.00433939	0.00336399	0.00020874	0.00098959
15	0.98374785	0.00329136	0.01486286	0.00284706	0.00608885
16	0.00313634	0.98514512	0.00031869	0.00618488	0.00092826
17	-0.00443298	0.00035037	0.97330447	-0.00131442	0.00581636
18	0.00465129	-0.00001034	0.97383557	0.00552810	-0.00156817
19	0.00901240	0.94981424	0.01336135	0.00984780	0.00324205
20	0.00000555	-0.00912443	0.00046496	0.00013098	0.00049261
21	0.00167815	0.00121424	-0.00829980	-0.00180357	0.00010846
22	0.00438383	0.97560634	0.00079813	0.00028537	0.00010001

	11	12	13	14	15
1	0.00161089	0.00329288	0.00216759	0.00050931	0.00100933
2	0.00013090	0.00012414	0.00009635	-0.00000381	-0.00005005
3	0.00294037	0.00039350	0.00011447	0.00068982	-0.00570547
4	0.97668285	0.00434654	-0.00380851	-0.00248765	-0.02599093
5	0.00277782	0.97311666	0.97566337	-0.00291870	-0.00989476
6	-0.00571209	-0.00511875	-0.00364595	0.97115764	0.98374785
7	0.00064988	-0.00333828	-0.01250555	0.00433939	0.00329136
8	-0.00616545	0.00021133	0.00061710	0.00336399	0.01486286
9	0.00009385	0.00025407	0.00003173	0.00020874	0.00284706
10	-0.00551904	0.00061047	0.00234004	0.00098959	0.00608885
11	0.96526151	0.00102307	0.00003404	-0.00212536	0.00117855
12	0.00102307	0.96971805	-0.00379053	-0.00244768	-0.00094836

13	0.00003404	-0.00379053	0.97152762	-0.00136757	0.00649317
14	-0.00212536	-0.00244768	-0.00136757	0.96869865	-0.00408178
15	0.00117855	-0.00094836	0.00649317	-0.00408178	0.97449373
16	-0.00010392	-0.00038770	0.00633127	0.00044918	0.00061232
17	-0.00176490	0.00004218	0.00028803	0.00002060	0.00115442
18	-0.00174260	0.00018853	0.00001571	0.00084153	0.00029254
19	0.00049443	0.00427077	0.00291736	0.00048118	-0.00083146
20	0.00004074	0.00019689	0.00034412	-0.00000028	-0.00006546
21	0.00073559	0.00000626	-0.00000103	0.00002031	0.00001516
22	0.00000186	-0.00232794	-0.00080759	0.00106020	0.00046855

	16	17	18	19	20
1	0.01707647	0.00448498	0.00012168	**1.95586601**	0.00021661
2	0.00026451	0.00028143	0.00039095	0.00069811	-0.00079367
3	-0.00464349	-0.00834499	-0.00189108	0.01050672	0.00911947
4	-0.00186735	-0.00010644	-0.00572241	-0.00421434	0.00016354
5	-0.01319599	-0.00003258	0.00064206	0.01683437	0.00109768
6	0.00313634	-0.00443298	0.00465129	0.00901240	0.00000555
7	0.98514512	0.00035037	-0.00001034	0.94981424	-0.00912443
8	0.00031869	0.97330447	0.97383557	0.01336135	0.00046496
9	0.00618488	-0.00131442	0.00552810	0.00984780	0.00013098
10	0.00092826	0.00581636	-0.00156817	0.00324205	0.00049261
11	-0.00010392	-0.00176490	-0.00174260	0.00049443	0.00004074
12	-0.00038770	0.00004218	0.00018853	0.00427077	0.00019689
13	0.00633127	0.00028803	0.00001571	0.00291736	0.00034412
14	0.00044918	0.00002060	0.00084153	0.00048118	-0.00000028
15	0.00061232	0.00115442	0.00029254	-0.00083146	-0.00006546
16	0.97528996	0.00021846	0.00011606	-0.02809407	0.00419079
17	0.00021846	0.96929721	-0.00279672	0.00317714	0.00015955
18	0.00011606	-0.00279672	0.97320665	0.00169170	0.00002424
19	-0.02809407	0.00317714	0.00169170	3.91637187	0.96562214
20	0.00419079	0.00015955	0.00002424	0.96562214	0.97197646
21	0.00037419	-0.00120053	-0.00138715	0.00648531	0.00079028
22	-0.00176409	-0.00000722	-0.00001484	-0.00481136	-0.00109986

	21	22
1	-0.00660304	0.00407151
2	-0.00074900	0.00080206
3	0.97829776	0.00095518
4	0.00323394	-0.00018157
5	0.00019266	-0.00471356
6	0.00167815	0.00438383
7	0.00121424	0.97560634
8	-0.00829980	0.00079813
9	-0.00180357	0.00028537
10	0.00010846	0.00010001
11	0.00073559	0.00000186

12	0.00000626	-0.00232794
13	-0.00000103	-0.00080759
14	0.00002031	0.00106020
15	0.00001516	0.00046855
16	0.00037419	-0.00176409
17	-0.00120053	-0.00000722
18	-0.00138715	-0.00001484
19	0.00648531	-0.00481136
20	0.00079028	-0.00109986
21	0.97322608	0.00011789
22	0.00011789	0.97292291

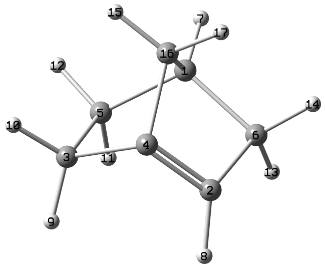

7 (Φ_p = 15.8º, average of both alkene terminus)

	1	2	3	4	5
1	3.94954321	0.01015183	-0.00878274	0.04629287	0.96180508
2	0.01015183	3.91165670	0.07947197	**1.76122811**	-0.00004240
3	-0.00878274	0.07947197	4.05038086	0.99788410	0.99628381
4	0.04629287	**1.76122811**	0.99788410	3.71655245	-0.05220452
5	0.96180508	-0.00004240	0.99628381	-0.05220452	3.88690978
6	0.99074576	1.01439043	0.01130063	-0.01914113	0.03028929
7	0.96150074	0.01313082	0.00559878	0.01247564	-0.00082213
8	0.00469393	0.98588303	0.00641095	-0.01952894	-0.00337207
9	0.00234772	0.00242526	0.96674116	-0.00265848	-0.00072881
10	-0.00154966	0.00762987	0.95570161	0.00306130	0.00009201
11	-0.00699433	0.00534439	0.00298840	0.00056678	0.96601292
12	0.00065215	0.00705175	-0.00044806	0.00301139	0.96108278
13	-0.00169667	0.00566434	0.00046592	0.00558312	0.00073173
14	-0.00027344	0.00289993	0.00081705	0.01474051	0.00606987
15	0.00078537	0.00366698	0.00348212	-0.00274285	0.00051094
16	0.98751407	0.01490650	0.02949602	0.96104044	0.01542021
17	0.00235052	-0.00214611	0.00296913	0.00694411	0.00578109

	6	7	8	9	10
1	0.99074576	0.96150074	0.00469393	0.00234772	-0.00154966
2	1.01439043	0.01313082	0.98588303	0.00242526	0.00762987
3	0.01130063	0.00559878	0.00641095	0.96674116	0.95570161
4	-0.01914113	0.01247564	-0.01952894	-0.00265848	0.00306130
5	0.03028929	-0.00082213	-0.00337207	-0.00072881	0.00009201
6	3.97177401	-0.00381250	-0.01254891	-0.00010438	0.00093811

7	-0.00381250	0.98264315	0.00037711	0.00029589	0.00008130
8	-0.01254891	0.00037711	0.97847512	0.00147690	0.00039529
9	-0.00010438	0.00029589	0.00147690	0.97800756	-0.00011524
10	0.00093811	0.00008130	0.00039529	-0.00011524	0.96819948
11	0.00313586	0.00093566	0.00027174	-0.00195875	0.00350927
12	0.00502823	-0.00064492	0.00018468	0.00181771	-0.00069675
13	0.96004271	-0.00031470	-0.00081900	0.00001604	0.00006710
14	0.94159128	0.00131004	0.00237596	0.00007868	0.00033992
15	0.00544498	-0.00039863	0.00096575	0.00022976	-0.00018340
16	0.03843627	-0.00696099	0.01161467	0.00694285	-0.00119867
17	0.00603738	-0.00010896	0.00009402	0.00120125	0.00012742

	11	12	13	14	15
1	-0.00699433	0.00065215	-0.00169667	-0.00027344	0.00078537
2	0.00534439	0.00705175	0.00566434	0.00289993	0.00366698
3	0.00298840	-0.00044806	0.00046592	0.00081705	0.00348212
4	0.00056678	0.00301139	0.00558312	0.01474051	-0.00274285
5	0.96601292	0.96108278	0.00073173	0.00606987	0.00051094
6	0.00313586	0.00502823	0.96004271	0.94159128	0.00544498
7	0.00093566	-0.00064492	-0.00031470	0.00131004	-0.00039863
8	0.00027174	0.00018468	-0.00081900	0.00237596	0.00096575
9	-0.00195875	0.00181771	0.00001604	0.00007868	0.00022976
10	0.00350927	-0.00069675	0.00006710	0.00033992	-0.00018340
11	0.98037157	-0.00076432	0.00100373	0.00012559	0.00026945
12	-0.00076432	0.97576528	0.00010832	0.00046984	0.00006921
13	0.00100373	0.00010832	0.97759947	-0.00032639	0.00173817
14	0.00012559	0.00046984	-0.00032639	0.97119435	0.00035237
15	0.00026945	0.00006921	0.00173817	0.00035237	0.98116059
16	0.00501283	-0.00141518	0.00483913	0.00049259	0.96755032
17	0.00091235	0.00025845	0.00049592	0.00013055	-0.00057996

	16	17
1	0.98751407	0.00235052
2	0.01490650	-0.00214611
3	0.02949602	0.00296913
4	0.96104044	0.00694411
5	0.01542021	0.00578109
6	0.03843627	0.00603738
7	-0.00696099	-0.00010896
8	0.01161467	0.00009402
9	0.00694285	0.00120125
10	-0.00119867	0.00012742
11	0.00501283	0.00091235
12	-0.00141518	0.00025845
13	0.00483913	0.00049592
14	0.00049259	0.00013055
15	0.96755032	-0.00057996

16	3.99313576	0.95944471
17	0.95944471	0.98391186

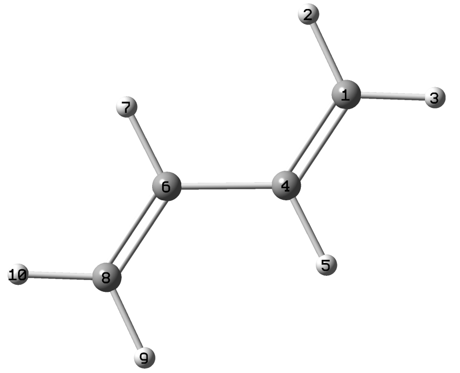

8

	1	2	3	4	5
1	3.94755644	0.96928160	0.97079762	1.87382633	0.01093760
2	0.96928160	0.97678147	-0.00113476	-0.00947247	0.00962387
3	0.97079762	-0.00113476	0.97542146	-0.00316204	0.00008344
4	1.87382633	-0.00947247	-0.00316204	3.87452543	0.95056310
5	0.01093760	0.00962387	0.00008344	0.95056310	0.97521941
6	0.02220564	0.00300634	0.00664732	1.04407807	-0.01316686
7	0.00501452	0.00404516	0.00022954	-0.01316686	0.00788903
8	0.09271726	0.00108949	0.00168637	0.02220564	0.00501452
9	0.00108949	0.00030916	0.00003307	0.00300634	0.00404516
10	0.00168637	0.00003307	0.00024091	0.00664732	0.00022954
	6	7	8	9	10
1	0.02220564	0.00501452	0.09271726	0.00108949	0.00168637
2	0.00300634	0.00404516	0.00108949	0.00030916	0.00003307
3	0.00664732	0.00022954	0.00168637	0.00003307	0.00024091
4	1.04407807	-0.01316686	0.02220564	0.00300634	0.00664732
5	-0.01316686	0.00788903	0.00501452	0.00404516	0.00022954
6	3.87452543	0.95056310	1.87382633	-0.00947247	-0.00316204
7	0.95056310	0.97521941	0.01093760	0.00962387	0.00008344
8	1.87382633	0.01093760	3.94755644	0.96928160	0.97079762
9	-0.00947247	0.00962387	0.96928160	0.97678147	-0.00113476
10	-0.00316204	0.00008344	0.97079762	-0.00113476	0.97542146

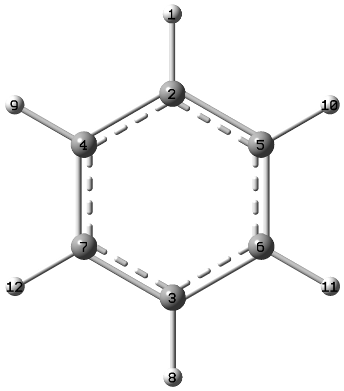

9

	1	2	3	4	5
1	0.96836039	0.97900124	0.00112001	-0.01266320	-0.01266679
2	0.97900124	3.91423850	0.07056936	1.40579667	1.40582097
3	0.00112001	0.07056936	3.91423850	0.03205775	0.03201628
4	-0.01266320	1.40579667	0.03205775	3.91423850	0.03201628
5	-0.01266679	1.40582097	0.03201628	0.03201628	3.91430869
6	0.00658661	0.03205775	1.40579667	0.07056936	1.40582097
7	0.00658601	0.03201628	1.40582097	1.40582097	0.07069165
8	0.00002122	0.00112001	0.97900124	0.00658661	0.00658601
9	-0.00049686	-0.01266320	0.00658661	0.97900124	0.00658601
10	-0.00050209	-0.01265249	0.00658532	0.00658532	0.97898508
11	0.00068769	0.00658661	-0.01266320	0.00112001	-0.01266679
12	0.00068655	0.00658532	-0.01265249	-0.01265249	0.00111904

	6	7	8	9	10
1	0.00658661	0.00658601	0.00002122	-0.00049686	-0.00050209
2	0.03205775	0.03201628	0.00112001	-0.01266320	-0.01265249
3	1.40579667	1.40582097	0.97900124	0.00658661	0.00658532
4	0.07056936	1.40582097	0.00658661	0.97900124	0.00658532
5	1.40582097	0.07069165	0.00658601	0.00658601	0.97898508
6	3.91423850	0.03201628	-0.01266320	0.00112001	-0.01265249
7	0.03201628	3.91430869	-0.01266679	-0.01266679	0.00111904
8	-0.01266320	-0.01266679	0.96836039	0.00068769	0.00068655
9	0.00112001	-0.01266679	0.00068769	0.96836039	0.00068655
10	-0.01265249	0.00111904	0.00068655	0.00068655	0.96836012
11	0.97900124	0.00658601	-0.00049686	0.00002122	-0.00050209
12	0.00658532	0.97898508	-0.00050209	-0.00050209	0.00002143

	11	12
1	0.00068769	0.00068655
2	0.00658661	0.00658532
3	-0.01266320	-0.01265249
4	0.00112001	-0.01265249

5	-0.01266679	0.00111904
6	0.97900124	0.00658532
7	0.00658601	0.97898508
8	-0.00049686	-0.00050209
9	0.00002122	-0.00050209
10	-0.00050209	0.00002143
11	0.96836039	0.00068655
12	0.00068655	0.96836012

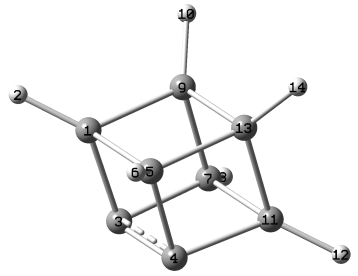

10 (Φ_p = 31.6º)

	1	2	3	4	5
1	3.88997775	0.94789683	0.92704003	0.09182132	0.87117668
2	0.94789683	0.95786422	0.00448257	0.00669935	-0.00620419
3	0.92704003	0.00448257	3.77413240	**1.58735585**	0.09182132
4	0.09182132	0.00669935	**1.58735585**	3.77413240	0.92704003
5	0.87117668	-0.00620419	0.09182132	0.92704003	3.88997775
6	-0.00620419	0.00001141	0.00669935	0.00448257	0.94789683
7	0.12105954	0.00409568	0.92704003	0.09182132	-0.01637993
8	0.00409568	0.00001414	0.00448257	0.00669935	0.00279638
9	0.91475292	-0.00838104	0.02160748	0.07940705	0.03555648
10	-0.00932651	-0.00077634	0.00740854	0.01826695	0.00569253
11	-0.01637993	0.00279638	0.09182132	0.92704003	0.12105954
12	0.00279638	0.00107077	0.00669935	0.00448257	0.00409568
13	0.03555648	0.00569078	0.07940705	0.02160748	0.91475292
14	0.00569253	0.00046788	0.01826695	0.00740854	-0.00932651

	6	7	8	9	10
1	-0.00620419	0.12105954	0.00409568	0.91475292	-0.00932651
2	0.00001141	0.00409568	0.00001414	-0.00838104	-0.00077634
3	0.00669935	0.92704003	0.00448257	0.02160748	0.00740854
4	0.00448257	0.09182132	0.00669935	0.07940705	0.01826695
5	0.94789683	-0.01637993	0.00279638	0.03555648	0.00569253
6	0.95786422	0.00279638	0.00107077	0.00569078	0.00046788
7	0.00279638	3.88997775	0.94789683	0.91475292	-0.00932651
8	0.00107077	0.94789683	0.95786422	-0.00838104	-0.00077634
9	0.00569078	0.91475292	-0.00838104	4.06620818	0.93866075
10	0.00046788	-0.00932651	-0.00077634	0.93866075	0.95130075

11	0.00409568	0.87117668	-0.00620419	0.03555648	0.00569253
12	0.00001414	-0.00620419	0.00001141	0.00569078	0.00046788
13	-0.00838104	0.03555648	0.00569078	1.13544234	-0.00414769
14	-0.00077634	0.00569253	0.00046788	-0.00414769	-0.00100292

	11	12	13	14
1	-0.01637993	0.00279638	0.03555648	0.00569253
2	0.00279638	0.00107077	0.00569078	0.00046788
3	0.09182132	0.00669935	0.07940705	0.01826695
4	0.92704003	0.00448257	0.02160748	0.00740854
5	0.12105954	0.00409568	0.91475292	-0.00932651
6	0.00409568	0.00001414	-0.00838104	-0.00077634
7	0.87117668	-0.00620419	0.03555648	0.00569253
8	-0.00620419	0.00001141	0.00569078	0.00046788
9	0.03555648	0.00569078	1.13544234	-0.00414769
10	0.00569253	0.00046788	-0.00414769	-0.00100292
11	3.88997775	0.94789683	0.91475292	-0.00932651
12	0.94789683	0.95786422	-0.00838104	-0.00077634
13	0.91475292	-0.00838104	4.06620818	0.93866075
14	-0.00932651	-0.00077634	0.93866075	0.95130075

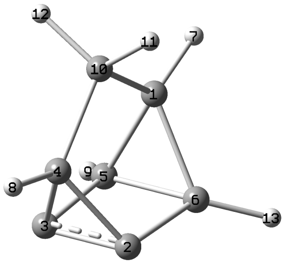

11 (Φ_{p} = 33.2°)

	1	2	3	4	5
1	3.93027781	0.05746893	0.05742901	0.00022798	0.91893021
2	0.05746893	3.67106285	**1.55230400**	1.01750849	0.04168067
3	0.05742901	**1.55230400**	3.67132629	1.01772413	0.96946828
4	0.00022798	1.01750849	1.01772413	4.08626678	0.04516286
5	0.91893021	0.04168067	0.96946828	0.04516286	3.88201608
6	0.91891227	0.96939918	0.04172404	0.04522635	0.94433872
7	0.96819461	0.00778319	0.00779237	0.00633080	-0.00496553
8	0.00632654	0.00325862	0.00325509	0.95675298	0.00541024
9	-0.00179107	0.00816253	-0.00353709	0.00401848	0.95929290
10	0.99522089	0.00901127	0.00901852	0.97182878	-0.00391179
11	0.00555065	0.00051603	0.00749413	0.00871651	0.00961666
12	0.00558258	0.00749262	0.00050691	0.00874753	0.00074756
13	-0.00177480	-0.00352268	0.00814689	0.00402188	-0.00375471

	6	7	8	9	10
1	0.91891227	0.96819461	0.00632654	-0.00179107	0.99522089
2	0.96939918	0.00778319	0.00325862	0.00816253	0.00901127
3	0.04172404	0.00779237	0.00325509	-0.00353709	0.00901852

4	0.04522635	0.00633080	0.95675298	0.00401848	0.97182878
5	0.94433872	-0.00496553	0.00541024	0.95929290	-0.00391179
6	3.88208723	-0.00494005	0.00541594	-0.00376487	-0.00385889
7	-0.00494005	0.97530082	0.00044221	-0.00084160	-0.00293450
8	0.00541594	0.00044221	0.97311396	-0.00011675	-0.00693028
9	-0.00376487	-0.00084160	-0.00011675	0.96292804	0.00058580
10	-0.00385889	-0.00293450	-0.00693028	0.00058580	3.86488598
11	0.00074611	-0.00036125	-0.00029392	0.00030016	0.94815051
12	0.00962374	-0.00036039	-0.00029038	0.00009422	0.94811330
13	0.95926468	-0.00083905	-0.00011632	0.00052533	0.00059236

	11	12	13
1	0.00555065	0.00558258	-0.00177480
2	0.00051603	0.00749262	-0.00352268
3	0.00749413	0.00050691	0.00814689
4	0.00871651	0.00874753	0.00402188
5	0.00961666	0.00074756	-0.00375471
6	0.00074611	0.00962374	0.95926468
7	-0.00036125	-0.00036039	-0.00083905
8	-0.00029392	-0.00029038	-0.00011632
9	0.00030016	0.00009422	0.00052533
10	0.94815051	0.94811330	0.00059236
11	0.97556624	-0.00496184	0.00009248
12	-0.00496184	0.97559636	0.00030052
13	0.00009248	0.00030052	0.96293659

K. Cartesian Coordinates for Optimized Structures

Ethylene 1 (ωB97X-D/def2-TZVP)

C	0.00000000	0.66082500	0.00000000
H	0.92264400	1.22921600	0.00000000
H	-0.92260700	1.22925400	0.00000000
C	0.00000000	-0.66082500	0.00000000
H	-0.92264400	-1.22921600	0.00000000
H	0.92260700	-1.22925400	0.00000000

Imaginary Frequencies: 0

2 (ωB97X-D/def2-TZVP)

H	-2.30014300	-2.01247100	2.43336700
C	-2.29038200	-1.97764800	1.34997100
C	-2.28089600	-1.88155200	-1.43546500
C	-3.35775000	-2.49975000	0.62594900
C	-1.22084000	-1.40913000	0.68687300
C	-1.22451200	-1.36646500	-0.71557100
C	-3.35341000	-2.45210100	-0.75710400
H	-2.27595100	-1.83229400	-2.51696600
H	-4.19486100	-2.94481000	1.14865000
H	-4.18736000	-2.85883400	-1.31485100
C	0.00000000	-0.67030600	-1.19778500
C	1.22451200	-1.36646500	-0.71557100
C	1.22084000	-1.40913000	0.68687300
C	0.00000000	-0.81257000	1.36897500
H	0.00000000	-1.11237500	2.41661700
C	2.29038200	-1.97764800	1.34997100
C	3.35775000	-2.49975000	0.62594900
C	3.35341000	-2.45210100	-0.75710400
C	2.28089600	-1.88155200	-1.43546500
H	2.30014300	-2.01247100	2.43336700
H	4.19486100	-2.94481000	1.14865000
H	4.18736000	-2.85883400	-1.31485100
H	2.27595100	-1.83229400	-2.51696600
H	-2.30014300	2.01247100	2.43336700
C	-2.29038200	1.97764800	1.34997100
C	-2.28089600	1.88155200	-1.43546500
C	-3.35775000	2.49975000	0.62594900
C	-1.22084000	1.40913000	0.68687300
C	-1.22451200	1.36646500	-0.71557100
C	-3.35341000	2.45210100	-0.75710400
H	-2.27595100	1.83229400	-2.51696600
H	-4.19486100	2.94481000	1.14865000
H	-4.18736000	2.85883400	-1.31485100
C	0.00000000	0.67030600	-1.19778500
C	1.22451200	1.36646500	-0.71557100
C	1.22084000	1.40913000	0.68687300
C	0.00000000	0.81257000	1.36897500
H	0.00000000	1.11237500	2.41661700
C	2.29038200	1.97764800	1.34997100
C	3.35775000	2.49975000	0.62594900
C	3.35341000	2.45210100	-0.75710400
C	2.28089600	1.88155200	-1.43546500
H	2.30014300	2.01247100	2.43336700
H	4.19486100	2.94481000	1.14865000
H	4.18736000	2.85883400	-1.31485100
H	2.27595100	1.83229400	-2.51696600

Imaginary Frequencies: 0

3 (ωB97X-D/def2-TZVP)

C	1.27438900	-1.25604300	-1.31177500
C	-0.00000100	0.66024900	-1.80971400
C	1.24988000	-1.24946800	1.17796800
C	0.78172800	2.03680800	-0.06299100
C	0.78172800	-2.03680800	-0.06298900
C	-1.27439000	-1.25604400	-1.31177500
C	0.00000000	-0.66025100	-1.80971300
C	0.00000100	-0.77153300	1.94728600
C	2.07832900	0.00000000	-0.84220900
C	-1.24987900	-1.24946800	1.17796800
C	2.02911300	0.00000100	0.70431200
C	1.27438900	1.25604200	-1.31177700
C	-1.27439000	1.25604200	-1.31177600
C	1.24988000	1.24946900	1.17796600
C	0.00000000	0.77153500	1.94728500
C	-1.24987900	1.24946900	1.17796700
C	-2.07832900	-0.00000100	-0.84220800
C	-0.78172800	2.03680800	-0.06299100
C	-0.78172800	-2.03680800	-0.06298900
C	-2.02911300	0.00000000	0.70431300
H	1.82350500	-1.88825400	-2.01099300
H	1.16827500	3.05540200	-0.05394100
H	-1.82350600	-1.88825400	-2.01099200
H	1.87931900	-1.87398100	1.81148900
H	0.00000100	-1.15996700	2.96547100
H	1.16827500	-3.05540200	-0.05393800
H	1.82350500	1.88825200	-2.01099500
H	3.03754700	0.00000100	1.11737400
H	-1.82350600	1.88825200	-2.01099400
H	-1.87931800	-1.87398100	1.81149000
H	-1.16827600	3.05540200	-0.05394100
H	3.10338100	0.00000000	-1.21243400
H	-1.87931800	1.87398300	1.81148800
H	1.87931900	1.87398300	1.81148700
H	0.00000100	1.15997000	2.96547000
H	-3.10338200	-0.00000100	-1.21243200
H	-3.03754600	0.00000000	1.11737500
H	-1.16827500	-3.05540200	-0.05393700

Imaginary Frequencies: 0

4 (ωB97X-D/def2-TZVP)

C	0.68384000	0.00000000	-0.95558200
C	-0.68384000	0.00000000	-0.95558200
C	-1.14583200	1.17693500	-0.09681700
C	0.00000000	0.82482000	0.91247900
C	1.14583200	1.17693500	-0.09681700
H	-1.03030100	2.17497000	-0.51202200
H	-2.16482600	1.06470000	0.26842400
H	0.00000000	1.28155800	1.90761600
H	1.03030100	2.17497000	-0.51202200
H	2.16482600	1.06470000	0.26842400
C	-1.14583200	-1.17693500	-0.09681700
C	0.00000000	-0.82482000	0.91247900
C	1.14583200	-1.17693500	-0.09681700
H	-2.16482600	-1.06470000	0.26842400
H	-1.03030100	-2.17497000	-0.51202200
H	0.00000000	-1.28155800	1.90761600
H	1.03030100	-2.17497000	-0.51202200
H	2.16482600	-1.06470000	0.26842400

Imaginary Frequencies: 0

5 (ωB97X-D/def2-TZVP)

C	0.00000000	0.67472300	-1.01543100
C	0.00000000	-0.67472300	-1.01543100
C	1.26953200	-1.16739200	-0.35206700
C	1.32123900	0.00000000	0.67586000
C	1.26953200	1.16739200	-0.35206700
H	2.16054700	-1.17225600	-0.98344500
H	1.15785000	-2.15731200	0.09149200

H	2.18677200	0.00000000	1.34923000
H	2.16054700	1.17225600	-0.98344500
H	1.15785000	2.15731200	0.09149200
C	-1.26953200	-1.16739200	-0.35206700
C	-1.32123900	0.00000000	0.67586000
C	-1.26953200	1.16739200	-0.35206700
H	-1.15785000	-2.15731200	0.09149200
H	-2.16054700	-1.17225600	-0.98344500
H	-2.18677200	0.00000000	1.34923000
H	-1.15785000	2.15731200	0.09149200
H	-2.16054700	1.17225600	-0.98344500
C	0.00000000	0.00000000	1.51070700
H	0.00000000	-0.87716200	2.16478800
H	0.00000000	0.87716200	2.16478800

Imaginary Frequencies: 0

6 (ωB97X-D/def2-TZVP)

C	-0.53015200	-1.36172400	0.39806300
C	0.53014900	-1.36172400	-0.39806500
C	-1.86392600	-0.90403500	-0.07898600
C	-1.87756400	0.63129600	0.13166600
H	-2.78230900	1.03689000	-0.32966400
H	-1.96220500	0.83990100	1.20305600
C	-0.65271600	1.38397600	-0.42426200
C	0.65271800	1.38397800	0.42426000
C	1.86392300	-0.90403900	0.07898800
H	2.70346700	-1.35469500	-0.45461900
H	1.98430600	-1.12256400	1.14342000
H	-2.70346900	-1.35469200	0.45462100
H	-1.98431100	-1.12256100	-1.14341800
C	1.87756600	0.63129400	-0.13166300
H	2.78231100	1.03688600	0.32966800
H	1.96220900	0.83989800	-1.20305300
H	-0.96086100	2.42400300	-0.55532000
H	0.43508900	1.01729700	1.43171100
H	-0.43508700	1.01728900	-1.43171100
H	0.96086600	2.42400600	0.55531200
H	0.37181900	-1.33789500	-1.47486100
H	-0.37181800	-1.33789700	1.47485800

Imaginary Frequencies: 0

***cis*-6** (ωB97X-D/def2-TZVP)

C	-0.55764400	-1.64451400	-0.31390900
H	-1.10474800	-2.29519800	-0.98982000
C	-1.36398900	-0.83806100	0.65637300
C	-0.78394700	1.34024100	-0.61580800
C	1.70570600	0.79982500	-0.04857700
C	0.45970700	1.64603400	0.23668100
C	1.65634100	-0.67253300	0.38592500
C	-1.86098200	0.48454600	0.05373400
H	-0.76389000	-0.61803400	1.54253200
H	-0.46762300	0.86168100	-1.54597000
H	-1.25621200	2.28127800	-0.90801800
H	2.55177600	1.26705200	0.46374900
H	1.93038100	0.85079200	-1.11900100
H	0.74325900	2.68830300	0.07167900
H	0.20976200	1.57519600	1.30120800
H	1.35676300	-0.71469900	1.43761300
H	-2.33741100	1.06274400	0.85143800
H	-2.64211600	0.27545500	-0.68344800
C	0.76008400	-1.55113100	-0.44517800
H	1.24349200	-2.13499500	-1.22266600
H	-2.22652400	-1.41280200	1.00117500
H	2.67143500	-1.07322100	0.34408500

Imaginary Frequencies: 0

7 (B3LYP/6-31G(d,p))

C	-0.45017500	-1.04702500	-0.12629800
C	-0.74702200	1.26850900	-0.12487900
C	1.55227700	0.54109500	-0.08344000
C	0.27081500	0.84677300	0.66954300
C	0.94654500	-0.75446200	-0.82006800
C	-1.55195900	0.01717900	-0.50368400
H	-0.75957000	-2.09532700	-0.22938000
H	-0.61604600	2.06848300	-0.85102400
H	1.84199600	1.30959700	-0.80406000
H	2.41575600	0.29145000	0.54190000
H	0.84441300	-0.57071100	-1.89484500
H	1.60230300	-1.62208100	-0.68920000
H	-1.80213000	0.06257700	-1.57028600
H	-2.48631500	-0.18736900	0.03862600
H	0.67738900	-1.01783700	1.79419400
C	-0.13872000	-0.50396800	1.27666300
H	-1.00835500	-0.44738700	1.93705400

Imaginary Frequencies: 0

7 (ωB97X-D/def2-TZVP)

C	-0.45224500	-1.03516400	-0.13967200
C	-0.73394800	1.26613600	-0.10721500
C	1.54896800	0.52968100	-0.07307800
C	0.27086600	0.82583200	0.67344500
C	0.93365300	-0.73504400	-0.82846100
C	-1.54453200	0.02993800	-0.49757100
H	-0.76208100	-2.07679200	-0.25615500
H	-0.59218300	2.06930300	-0.82178600
H	1.84368300	1.30710600	-0.77580200
H	2.40004900	0.26175100	0.55335600
H	0.82276700	-0.52311100	-1.89334300
H	1.58214200	-1.60637700	-0.72312800
H	-1.78913500	0.08878400	-1.56039100
H	-2.47386200	-0.17299700	0.04371500
H	0.67573200	-1.04177600	1.76654700
C	-0.13963400	-0.52081800	1.26341400
H	-1.00588200	-0.46925400	1.92181200

Imaginary Frequencies: 0

7 (M06-2X/def2-TZVP)

C	-0.45585900	-1.03590400	-0.13909300
C	-0.72845000	1.26838000	-0.10951500
C	1.55196600	0.52446400	-0.07304100
C	0.27382900	0.82727500	0.67312200
C	0.92946500	-0.73702500	-0.82976200
C	-1.54561100	0.03255600	-0.49598800
H	-0.76804400	-2.07636800	-0.25338300
H	-0.58440100	2.06968600	-0.82538000
H	1.84856100	1.30037100	-0.77557000
H	2.39949700	0.25054800	0.55416200
H	0.81426500	-0.51755000	-1.89225900
H	1.57593600	-1.60980500	-0.72966000
H	-1.79362800	0.09087500	-1.55746800
H	-2.47139800	-0.16606000	0.05157500
H	0.67648300	-1.03975300	1.76502700
C	-0.14033700	-0.51959500	1.26467500
H	-1.00728800	-0.46284500	1.92056300

Imaginary Frequencies: 0

8 (ωB97X-D/def2-TZVP)

C	-0.60347000	1.73660000	0.00000000
H	0.32384000	2.29896800	0.00000000
H	-1.52495700	2.30369300	0.00000000
C	-0.60347000	0.40784700	0.00000000
H	-1.54836900	-0.12916200	0.00000000
C	0.60347000	-0.40784700	0.00000000
H	1.54836900	0.12916200	0.00000000

C	0.60347000	-1.73660000	0.00000000
H	-0.32384000	-2.29896800	0.00000000
H	1.52495700	-2.30369300	0.00000000

Imaginary Frequencies: 0

9 (ωB97X-D/def2-TZVP)

H	0.00000000	2.13897200	1.23524700
C	0.00000000	1.20136600	0.69363900
C	0.00000000	-1.20136600	-0.69363900
C	0.00000000	1.20136600	-0.69363900
C	0.00000000	0.00000000	1.38743800
C	0.00000000	-1.20136600	0.69363900
C	0.00000000	0.00000000	-1.38743800
H	0.00000000	-2.13897200	-1.23524700
H	0.00000000	2.13897200	-1.23524700
H	0.00000000	0.00000000	2.47022300
H	0.00000000	-2.13897200	1.23524700
H	0.00000000	0.00000000	-2.47022300

Imaginary Frequencies: 0

75 (B3LYP/6-31G(d,p))

C	0.00000000	0.62542900	-1.23731100
C	0.00000000	-0.70361200	1.05798900
C	0.00000000	1.46173200	-0.13340800
C	0.00000000	-0.62542900	-1.23731100
C	0.00000000	-1.46173200	-0.13340800
C	0.00000000	0.70361200	1.05798900
H	0.00000000	-1.22819600	2.01043200
H	0.00000000	2.54627300	-0.13405300
H	0.00000000	-2.54627300	-0.13405300
H	0.00000000	1.22819600	2.01043200

Imaginary Frequencies: 0

benzyne 75 (ωB97X-D/def2-TZVP)

C	0.00000000	0.61830000	-1.22713000
C	0.00000000	-0.69990000	1.04887000
C	0.00000000	1.45410000	-0.13243000
C	0.00000000	-0.61830000	-1.22713000
C	0.00000000	-1.45410000	-0.13243000
C	0.00000000	0.69990000	1.04887000
H	0.00000000	-1.22230000	1.99837000
H	0.00000000	2.53470000	-0.13423000
H	0.00000000	-2.53470000	-0.13423000
H	0.00000000	1.22230000	1.99837000

Imaginary Frequencies: 0

75 (M06–2X/def2-TZVP)

C	0.00000000	0.61826800	-1.22746500
C	0.00000000	-0.70055200	1.04920000
C	0.00000000	1.45609600	-0.13252800
C	0.00000000	-0.61826800	-1.22746500
C	0.00000000	-1.45609600	-0.13252800
C	0.00000000	0.70055200	1.04920000
H	0.00000000	-1.22257600	1.99844800
H	0.00000000	2.53641900	-0.13369100
H	0.00000000	-2.53641900	-0.13369100
H	0.00000000	1.22257600	1.99844800

Imaginary Frequencies: 0

cubene 10 (ωB97X-D/def2-TZVP)

C	-1.17725400	0.77424800	-0.08082700
H	-2.01087300	1.47006000	-0.07681200
C	0.00000000	0.69159000	-1.08367300
C	0.00000000	-0.69159000	-1.08367300
C	-1.17725400	-0.77424800	-0.08082700
H	-2.01087300	-1.47006000	-0.07681200
C	1.17725400	0.77424800	-0.08082700
H	2.01087300	1.47006000	-0.07681200
C	0.00000000	0.76776900	0.96258100
H	0.00000000	1.38828000	1.85010100
C	1.17725400	-0.77424800	-0.08082700
H	2.01087300	-1.47006000	-0.07681200
C	0.00000000	-0.76776900	0.96258100
H	0.00000000	-1.38828000	1.85010100

Imaginary Frequencies: 0

cubene 10 (M06–2X/def2-TZVP)

C	-1.17994700	0.77308300	-0.08129500
H	-2.01127800	1.47079100	-0.07712600
C	0.00000000	0.69127700	-1.07775100
C	0.00000000	-0.69127700	-1.07775100
C	-1.17994700	-0.77308300	-0.08129500
H	-2.01127800	-1.47079100	-0.07712600
C	1.17994700	0.77308300	-0.08129500
H	2.01127800	1.47079100	-0.07712600
C	0.00000000	0.76741300	0.95839300
H	0.00000000	1.38775100	1.84594400
C	1.17994700	-0.77308300	-0.08129500
H	2.01127800	-1.47079100	-0.07712600
C	0.00000000	-0.76741300	0.95839300
H	0.00000000	-1.38775100	1.84594400

Imaginary Frequencies: 0

cubene 10 (B3LYP/6-31G(d,p))

C	-1.18126400	0.77910900	-0.08189000
H	-2.02020700	1.47637600	-0.07757400
C	0.00000000	0.69611000	-1.09343600
C	0.00000000	-0.69611000	-1.09343600
C	-1.18126400	-0.77910900	-0.08189000
H	-2.02020700	-1.47637600	-0.07757400
C	1.18126400	0.77910900	-0.08189000
H	2.02020700	1.47637600	-0.07757400
C	0.00000000	0.77293900	0.97260000
H	0.00000000	1.39780700	1.86284800
C	1.18126400	-0.77910900	-0.08189000
H	2.02020700	-1.47637600	-0.07757400
C	0.00000000	-0.77293900	0.97260000
H	0.00000000	-1.39780700	1.86284800

Imaginary Frequencies: 0

cubene 10 (CASSCF(4,4)/def2-TZVP)

C	-1.1581405	0.7768706	-0.0805278
H	-1.9942612	1.4564703	-0.0820620
C	-0.0000011	0.6955686	-1.1008102
C	-0.0000009	-0.6955676	-1.1007734
C	-1.1581500	-0.7768596	-0.0805159
H	-1.9942760	-1.4564514	-0.0820234
C	1.1581410	0.7768702	-0.0805277
H	1.9942618	1.4564698	-0.0820619
C	-0.0000002	0.7697357	0.9778236
H	0.0000003	1.3836188	1.8618366
C	1.1581505	-0.7768592	-0.0805156
H	1.9942767	-1.4564508	-0.0820227
C	-0.0000004	-0.7697353	0.9778374
H	-0.0000000	-1.3836801	1.8618050

cubene 10 (HF/6-31G(d,p))

C	-1.17010100	0.77383800	-0.08115400
H	-2.00130600	1.46289400	-0.08734000

C	0.00000000	0.68039900	-1.08591600
C	0.00000000	-0.68039900	-1.08591600
C	-1.17010100	-0.77383800	-0.08115400
H	-2.00130600	-1.46289400	-0.08734000
C	1.17010100	0.77383800	-0.08115400
H	2.00130600	1.46289400	-0.08734000
C	0.00000000	0.76897200	0.96844800
H	0.00000000	1.38452800	1.85333500
C	1.17010100	-0.77383800	-0.08115400
H	2.00130600	-1.46289400	-0.08734000
C	0.00000000	-0.76897200	0.96844800
H	0.00000000	-1.38452800	1.85333500

Imaginary Frequencies: 0

cubene 10 (UHF/6-31G(d,p))

C	-1.13842700	0.78043900	-0.07940800
H	-1.99561300	1.43723600	-0.07574800
C	0.00000000	0.72911100	-1.12566600
C	0.00000000	-0.72911100	-1.12566600
C	-1.13842700	-0.78043900	-0.07940800
H	-1.99561300	-1.43723600	-0.07574800
C	1.13842700	0.78043900	-0.07940800
H	1.99561300	1.43723600	-0.07574800
C	0.00000000	0.77429100	0.99610600
H	0.00000000	1.39145900	1.88175300
C	1.13842700	-0.78043900	-0.07940800
H	1.99561300	-1.43723600	-0.07574800
C	0.00000000	-0.77429100	0.99610600
H	0.00000000	-1.39145900	1.88175300

Imaginary Frequencies: 0

cubene 10 (MP2/6-31G(d,p))

C	-1.18351700	0.77684500	-0.08074100
H	-2.01629600	1.47563000	-0.07007800
C	0.00000000	0.70804200	-1.08459800
C	0.00000000	-0.70804200	-1.08459800
C	-1.18351700	-0.77684500	-0.08074100
H	-2.01629600	-1.47563000	-0.07007800
C	1.18351700	0.77684500	-0.08074100
H	2.01629600	1.47563000	-0.07007800
C	0.00000000	0.76971500	0.96066400
H	0.00000000	1.38621200	1.85264800
C	1.18351700	-0.77684500	-0.08074100
H	2.01629600	-1.47563000	-0.07007800
C	0.00000000	-0.76971500	0.96066400
H	0.00000000	-1.38621200	1.85264800

Imaginary Frequencies: 0

1,7-quadricyclene 11 (ωB97X-D/def2-TZVP)

C	0.37716100	1.10231000	0.00069200
C	-0.01329800	-1.08113900	0.67460900
C	-0.01363700	-1.08054700	-0.67508900
C	-1.25093100	-0.54986400	0.00022300
C	1.09005600	-0.02030300	-0.74832700
C	1.09097900	-0.02116800	0.74775800
H	0.82768600	2.08829900	0.00093400
H	-2.18737200	-1.08621300	0.00028600
H	1.85831400	0.13707200	-1.49131900
C	-1.14194800	0.96674600	0.00020000
H	-1.59498200	1.41478700	0.88706500
H	-1.59383300	1.41474700	-0.88740000
H	1.85989600	0.13510700	1.49003800

Imaginary Frequencies: 0

1,7-quadricyclene 11 (M06-2X/def2-TZVP)

C	-0.76833900	-0.77258000	0.74587200
H	-1.18503400	-1.43711900	1.48778400
C	-0.76833900	0.75685300	0.67485100
C	-0.76833900	0.75685300	-0.67485100
C	-0.76833900	-0.77258000	-0.74587200
H	-1.18503400	-1.43711900	-1.48778400
C	0.46414800	1.28312100	0.00000000
H	0.72005100	2.33093900	0.00000000
C	0.53416700	-1.03508700	0.00000000
H	0.93053200	-2.04335500	0.00000000
C	1.48744700	0.15644700	0.00000000
H	2.12252300	0.17424400	-0.88742400
H	2.12252300	0.17424400	0.88742400

Imaginary Frequencies: 0

1,7-quadricyclene 11 (B3LYP/6-31G(d,p))

C	-0.77633800	-0.77413500	0.75103300
H	-1.19449200	-1.44131000	1.49793100
C	-0.77633800	0.76383900	0.68047300
C	-0.77633800	0.76383900	-0.68047300
C	-0.77633800	-0.77413500	-0.75103300
H	-1.19449200	-1.44131000	-1.49793100
C	0.47294400	1.28728300	0.00000000
H	0.74160300	2.33741700	0.00000000
C	0.53737500	-1.04275100	0.00000000
H	0.93763700	-2.05475200	0.00000000
C	1.49951100	0.15314900	0.00000000
H	2.14144200	0.16871300	-0.88890100
H	2.14144200	0.16871300	0.88890100

Imaginary Frequencies: 0

1,7-quadricyclene 11 (CASSCF(4,4)/def2-TZVP)

C	0.3803219	1.1046095	0.0001612
C	-0.0108818	-1.0872193	0.6798976
C	-0.0110804	-1.0868714	-0.6807156
C	-1.2450802	-0.5483264	-0.0000634
C	1.0773856	-0.0093774	-0.7473492
C	1.0776195	-0.0097244	0.7467902
H	0.8307228	2.0811928	0.0002867
H	-2.1737452	-1.0805199	-0.0000460
H	1.8482520	0.1339216	-1.4774903
C	-1.1377133	0.9665028	0.0003466
H	-1.5881325	1.4112305	0.8812702
H	-1.5883552	1.4117264	-0.8802127
H	1.8487779	0.1326894	1.4767948

1,7-quadricyclene 11 (HF/6-31G(d,p))

C	-0.76836000	-0.77161100	0.74431400
H	-1.19503900	-1.42562700	1.48165200
C	-0.76836000	0.75941100	0.66620100
C	-0.76836000	0.75941100	-0.66620100
C	-0.76836000	-0.77161100	-0.74431400
H	-1.19503900	-1.42562700	-1.48165200
C	0.46821900	1.28289900	0.00000000
H	0.72954400	2.32326800	0.00000000
C	0.52926400	-1.03861400	0.00000000
H	0.92544700	-2.04119800	0.00000000
C	1.49033700	0.15234400	0.00000000
H	2.12440500	0.16789900	-0.88166500
H	2.12440500	0.16789900	0.88166500

Imaginary Frequencies: 0

1,7-quadricyclene 11 (UHF/6-31G(d,p))

C	-0.76217600	-0.74246500	0.74945500
H	-1.23788300	-1.39206900	1.45918300

C	-0.76217600	0.79330400	0.70774300
C	-0.76217600	0.79330400	-0.70774300
C	-0.76217600	-0.74246500	-0.74945500
H	-1.23788300	-1.39206900	-1.45918300
C	0.48282500	1.24718600	0.00000000
H	0.77114500	2.28197900	0.00000000
C	0.51197300	-1.06237900	0.00000000
H	0.87136300	-2.07756500	0.00000000
C	1.48649200	0.10629200	0.00000000
H	2.11887300	0.11152700	-0.88300100
H	2.11887300	0.11152700	0.88300100

Imaginary Frequencies: 0

1,7-quadricyclene 11 (MP2/6-31G(d,p))

C	-0.77135400	-0.76982600	0.74980900
H	-1.18067400	-1.43789800	1.49456400
C	-0.77135400	0.75939500	0.69531600
C	-0.77135400	0.75939500	-0.69531600
C	-0.77135400	-0.76982600	-0.74980900
H	-1.18067400	-1.43789800	-1.49456400
C	0.46629400	1.27921100	0.00000000
H	0.72394900	2.32876000	0.00000000
C	0.53654600	-1.03617400	0.00000000
H	0.93125400	-2.04679600	0.00000000
C	1.49146000	0.15376600	0.00000000
H	2.12642000	0.16909300	-0.88831800
H	2.12642000	0.16909300	0.88831800

Imaginary Frequencies: 0

cubane 76 (ωb97X-D/def2-TZVP)

C	1.22060700	0.37983800	-0.43706100
C	-0.11200900	1.14308000	-0.71128800
C	-0.81332400	-0.20639000	-1.05877300
C	0.51924500	-0.96952600	-0.78445000
C	0.81332500	0.20638900	1.05878400
C	0.11203500	-1.14308100	0.71128300
C	-1.22058800	-0.37984800	0.43705800
C	-0.51927600	0.96952900	0.78443400
H	2.20207700	0.68524700	-0.78851300
H	-0.20215700	2.06223000	-1.28321900
H	-1.46736500	-0.37230200	-1.91010000
H	0.93681200	-1.74913100	-1.41527300
H	1.46727500	0.37242200	1.91015700
H	0.20212500	-2.06221500	1.28324700
H	-2.20202700	-0.68534200	0.78852600
H	-0.93682600	1.74914300	1.41525100

Imaginary Frequencies: 0

quadricyclane 77 (ωb97X-D/def2-TZVP)

C	0.00000000	1.14500000	0.54712400
C	0.75410000	-0.77050000	-0.70477600
C	-0.75410000	-0.77050000	-0.70477600
C	0.00000000	-1.14500000	0.54712400
C	-0.75410000	0.77050000	-0.70477600
C	0.75410000	0.77050000	-0.70477600
H	0.00000000	2.17690000	0.86932400
H	1.44080000	-1.42040000	-1.22427600
H	-1.44080000	-1.42040000	-1.22427600
H	0.00000000	-2.17690000	0.86932400
H	-1.44080000	1.42040000	-1.22427600
H	1.44080000	1.42040000	-1.22427600
C	0.00000000	0.00000000	1.52982400
H	-0.88910000	0.00000000	2.16432400
H	0.88910000	0.00000000	2.16432400

Imaginary Frequencies: 0

78 (ωb97X-D/def2-TZVP)

C	0.00000000	-1.26890200	-0.67618500
H	0.87778100	-1.31300900	-1.32725800
H	-0.87768200	-1.31294100	-1.32743000
H	-0.00011900	-2.15181200	-0.03768100
C	0.00000000	0.00000000	0.12508200
C	0.00000000	0.00000000	1.45208600
H	-0.00001300	-0.92412100	2.01797500
H	0.00001300	0.92412100	2.01797500
C	0.00000000	1.26890200	-0.67618500
H	0.87768200	1.31294100	-1.32743000
H	-0.87778100	1.31300900	-1.32725800
H	0.00011900	2.15181200	-0.03768100

Imaginary Frequencies: 0

79 (ωb97X-D/def2-TZVP)

C	0.00000000	0.00000000	0.37368000
H	0.00000000	0.00000000	1.46924200
C	0.00000000	1.45186800	-0.09552800
H	-0.88346600	1.98494800	0.26223400
H	0.88346600	1.98494800	0.26223400
H	0.00000000	1.50178500	-1.18840700
C	-1.25735400	-0.72593400	-0.09552800
H	-1.30058400	-0.75089300	-1.18840700
H	-1.27728200	-1.75757800	0.26223400
H	-2.16074900	-0.22737000	0.26223400
C	1.25735400	-0.72593400	-0.09552800
H	2.16074900	-0.22737000	0.26223400
H	1.27728200	-1.75757800	0.26223400
H	1.30058400	-0.75089300	-1.18840700

Imaginary Frequencies: 0

Ethylene 1 (ωB97X-D/def2-TZVP/SMD(THF))

C	0.00000000	0.66147700	0.00000000
H	0.92364600	1.23052100	0.00000000
H	-0.92364000	1.23052700	0.00000000
C	0.00000000	-0.66147700	0.00000000
H	-0.92364600	-1.23052100	0.00000000
H	0.92364000	-1.23052700	0.00000000

Imaginary Frequencies: 0

TS-1 (ωB97X-D/def2-TZVP/SMD(THF))

H	-2.43670900	2.47986300	-0.43137900
C	-2.43776200	1.39575500	-0.44597600
C	-2.43776200	-1.39575500	-0.44597600
C	-3.59853800	0.70407700	-0.67408400
C	-1.22674200	0.70454800	-0.24042300
C	-1.22674200	-0.70454800	-0.24042300
C	-3.59853800	-0.70407700	-0.67408400
H	-2.43670900	-2.47986300	-0.43137900
H	-4.52321100	1.23939900	-0.85239000
H	-4.52321100	-1.23939900	-0.85239000
C	0.00000000	-1.34946900	0.08945800
C	1.22674200	-0.70454800	-0.24042300
C	1.22674200	0.70454800	-0.24042300
C	0.00000000	1.34946900	0.08945800
H	0.00000000	-2.43125500	0.17146700
H	0.00000000	2.43125500	0.17146700
C	2.43776200	1.39575500	-0.44597600
C	3.59853800	0.70407700	-0.67408400
C	3.59853800	-0.70407700	-0.67408400

C	2.43776200	-1.39575500	-0.44597600
H	2.43670900	2.47986300	-0.43137900
H	4.52321100	1.23939900	-0.85239000
H	4.52321100	-1.23939900	-0.85239000
H	2.43670900	-2.47986300	-0.43137900
C	0.00000000	0.69093800	2.22064100
H	0.91504600	1.22920400	2.43063900
H	-0.91504600	1.22920400	2.43063900
C	0.00000000	-0.69093800	2.22064100
H	-0.91504600	-1.22920400	2.43063900
H	0.91504600	-1.22920400	2.43063900

Imaginary Frequencies: 1

Cycloadduct 81 (ωB97X-D/def2-TZVP/SMD(THF))

H	-2.27878600	2.47917700	-0.52171700
C	-2.27869300	1.39489100	-0.52407300
C	-2.27869300	-1.39489100	-0.52407300
C	-3.34311300	0.69350200	-1.08316400
C	-1.21785900	0.69836100	0.02571900
C	-1.21785900	-0.69836100	0.02571900
C	-3.34311300	-0.69350200	-1.08316400
H	-2.27878600	-2.47917700	-0.52171700
H	-4.17353400	1.23374200	-1.52136900
H	-4.17353400	-1.23374200	-1.52136900
C	0.00000000	-1.29265000	0.69026000
C	1.21785900	-0.69836100	0.02571900
C	1.21785900	0.69836100	0.02571900
C	0.00000000	1.29265000	0.69026000
H	0.00000000	-2.38106600	0.65402700
H	0.00000000	2.38106600	0.65402700
C	2.27869300	1.39489100	-0.52407300
C	3.34311300	0.69350200	-1.08316400
C	3.34311300	-0.69350200	-1.08316400
C	2.27869300	-1.39489100	-0.52407300
H	2.27878600	2.47917700	-0.52171700
H	4.17353400	1.23374200	-1.52136900
H	4.17353400	-1.23374200	-1.52136900
H	2.27878600	-2.47917700	-0.52171700
C	0.00000000	-0.77274100	2.15515400
H	-0.87973400	-1.16168800	2.66893300
H	0.87973400	-1.16168800	2.66893300
C	0.00000000	0.77274100	2.15515400
H	0.87973400	1.16168800	2.66893300
H	-0.87973400	1.16168800	2.66893300

Imaginary Frequencies: 0

Cubene 10 (ωB97X-D/def2-TZVP/SMD(THF))

C	-1.18232700	0.77279800	-0.08002700
H	-2.01422800	1.46982900	-0.07248500
C	0.00000000	0.69366600	-1.07714300
C	0.00000000	-0.69366600	-1.07714300
C	-1.18232700	-0.77279800	-0.08002700
H	-2.01422800	-1.46982900	-0.07248500
C	1.18232700	0.77279800	-0.08002700
H	2.01422800	1.46982900	-0.07248500
C	0.00000000	0.76601600	0.95473700
H	0.00000000	1.38988100	1.83972800
C	1.18232700	-0.77279800	-0.08002700
H	2.01422800	-1.46982900	-0.07248500
C	0.00000000	-0.76601600	0.95473700
H	0.00000000	-1.38988100	1.83972800

Imaginary Frequencies: 0

Anthracene 21 (ωB97X-D/def2-TZVP/SMD(THF))

C	3.63649000	0.71156800	-0.00000900
C	2.46677300	1.40049400	0.00002900
C	1.21415200	0.71531500	0.00003600
C	1.21415200	-0.71531300	0.00000700
C	2.46677200	-1.40049500	-0.00002100
C	3.63649000	-0.71156900	-0.00003600
C	0.00000100	1.39597700	0.00002200
C	0.00000100	-1.39597600	0.00000500
C	-1.21415300	-0.71531300	0.00001500
C	-1.21415300	0.71531400	0.00000400
C	-2.46677200	1.40049400	-0.00003600
H	-2.46339100	2.48454100	-0.00007600
C	-3.63649100	0.71156700	-0.00003200
C	-3.63649100	-0.71156800	0.00000300
C	-2.46677200	-1.40049400	0.00002700
H	-0.00000300	2.48086400	0.00001800
H	4.58083900	1.24193200	-0.00002300
H	2.46339100	2.48454100	0.00006100
H	2.46338500	-2.48454100	-0.00003000
H	4.58083800	-1.24193600	-0.00006200
H	-0.00000300	-2.48086300	0.00000800
H	-4.58083700	1.24193600	-0.00003800
H	-4.58083800	-1.24193600	-0.00000600
H	-2.46338900	-2.48454100	0.00006300

Imaginary Frequencies: 0

TS-2 (ωB97X-D/def2-TZVP/SMD(THF))

C	-0.00000700	-1.11104100	-1.37851300
H	-0.00002500	-1.06278300	-2.46167900
C	-0.00002000	1.27024000	-0.70859900
C	1.16526700	2.29045100	-0.77451200
C	-0.00005500	3.34156300	-0.76991300
H	-0.00007800	4.23075200	-1.38984200
C	-1.16534200	2.29041300	-0.77448300
H	-2.00867000	2.28956900	-1.46005600
C	-1.16532300	2.29038500	0.77455400
C	-0.00003600	3.34153600	0.76999200
H	-0.00004300	4.23070200	1.38995300
C	1.16528600	2.29042400	0.77452500
H	2.00861300	2.28958300	1.46010000
C	-0.00000200	1.27021700	0.70860200
C	-1.22563300	-1.29643300	-0.70874700
C	-1.22561000	-1.29642800	0.70876000
C	0.00003600	-1.11103200	1.37848600
H	0.00005300	-1.06276700	2.46165100
C	1.22566500	-1.29640500	0.70872100
C	1.22564300	-1.29641000	-0.70878600
C	2.46072200	-1.39249600	-1.39641500
C	3.63356600	-1.50283800	-0.70698700
C	3.63358900	-1.50283400	0.70684800
C	2.46076600	-1.39248800	1.39631300
C	-2.46068800	-1.39253400	1.39639000
C	-3.63353000	-1.50290200	0.70696200
C	-3.63355200	-1.50290500	-0.70687200
C	-2.46073200	-1.39254100	-1.39633800
H	2.00857800	2.28963500	-1.46010700
H	-2.00863300	2.28951600	1.46015000
H	2.45747300	-1.38013800	-2.48047100
H	4.57219700	-1.58727000	-1.24089400
H	4.57223600	-1.58726400	1.24072600
H	2.45755100	-1.38012400	2.48036800
H	-2.45743800	-1.38017100	2.48044500
H	-4.57215900	-1.58734900	1.24087000
H	-4.57219800	-1.58735500	-1.24075000
H	-2.45751700	-1.38018200	-2.48039300

Imaginary Frequencies: 1

Cycloadduct 22 (ωB97X-D/def2-TZVP/SMD(THF))

C	0.00000000	-1.32298300	-0.28107300
H	0.00000000	-2.41067600	-0.34807300
C	0.00000000	-0.77662900	1.14016800
C	-1.11523700	-0.78225000	2.22846500

C	0.00000000	-0.77821000	3.32071700
H	0.00000000	-1.40521400	4.20794400
C	1.11523700	-0.78225000	2.22846500
H	1.99458200	-1.42114100	2.23069400
C	1.11523700	0.78225000	2.22846500
C	0.00000000	0.77821000	3.32071700
H	0.00000000	1.40521400	4.20794400
C	-1.11523700	0.78225000	2.22846500
H	-1.99458200	1.42114100	2.23069400
C	0.00000000	0.77662900	1.14016800
C	1.22127500	-0.69998500	-0.92602800
C	1.22127500	0.69998500	-0.92602800
C	0.00000000	1.32298300	-0.28107300
H	0.00000000	2.41067600	-0.34807300
C	-1.22127500	0.69998500	-0.92602800
C	-1.22127500	-0.69998500	-0.92602800
C	-2.29607600	-1.39311600	-1.45264000
C	-3.37172900	-0.69324300	-1.99034200
C	-3.37172900	0.69324300	-1.99034200
C	-2.29607600	1.39311600	-1.45264000
C	2.29607600	1.39311600	-1.45264000
C	3.37172900	0.69324300	-1.99034200
C	3.37172900	-0.69324300	-1.99034200
C	2.29607600	-1.39311600	-1.45264000
H	-1.99458200	-1.42114100	2.23069400
H	1.99458200	1.42114100	2.23069400
H	-2.29700900	-2.47728800	-1.44722200
H	-4.21116300	-1.23453600	-2.40958000
H	-4.21116300	1.23453600	-2.40958000
H	-2.29700900	2.47728800	-1.44722200
H	2.29700900	2.47728800	-1.44722200
H	4.21116300	1.23453600	-2.40958000
H	4.21116300	-1.23453600	-2.40958000
H	2.29700900	-2.47728800	-1.44722200

Imaginary Frequencies: 0

1,7-quadricyclene 11 (ωB97X-D/def2-TZVP/SMD(THF))

C	-0.37856700	1.10052000	0.00022500
C	0.01565500	-1.07873000	-0.67547800
C	0.01566900	-1.07873000	0.67536400
C	1.25487600	-0.54719700	0.00007300
C	-1.09287400	-0.02303600	0.74709500
C	-1.09269900	-0.02262800	-0.74727500
H	-0.83101100	2.08491400	0.00038300
H	2.19188800	-1.08303100	-0.00011200
H	-1.86225900	0.13282200	1.48916500
C	1.14081800	0.96679700	-0.00001700
H	1.59307300	1.41503300	-0.88702900
H	1.59285300	1.41478100	0.88723300
H	-1.86181100	0.13350600	-1.48956500

Imaginary Frequencies: 0

TS-3 (ωB97X-D/def2-TZVP/SMD(THF))

C	-0.15694900	-1.05005800	1.38258400
C	1.04646700	-1.32223400	0.70951700
C	1.04638900	-1.32323900	-0.70894800
C	2.27085500	-1.52105900	-1.39732800
C	3.42893900	-1.72229000	-0.70750900
C	3.42901300	-1.72127000	0.70838300
C	2.27100200	-1.51905700	1.39804400
C	0.11180200	1.38231600	0.68772000
C	-0.97561700	2.16360200	-0.00040600
H	-2.00077400	1.81736100	-0.00035200
C	0.11160100	1.38240600	-0.68937500
C	-0.15709600	-1.05211300	-1.38227200
H	-0.15342700	-1.01197600	-2.46569800
C	-1.39057500	-1.07685400	-0.70874200
C	-1.39051000	-1.07587200	0.70921300
C	-2.63047700	-1.02869600	1.39771400
C	-3.80588000	-1.00521000	0.70832900
C	-3.80595000	-1.00619800	-0.70772800
C	-2.63061700	-1.03064200	-1.39719000
C	1.40096200	2.21501600	-0.74857500
H	2.19402100	2.20364500	-1.48279900
C	0.94263900	3.45803900	-0.00045200
H	1.58823700	4.32836700	-0.00039100
C	-0.57087300	3.62668600	-0.00021800
C	1.40115000	2.21478200	0.74713600
H	2.19435000	2.20309600	1.48119800
H	2.26795300	-1.50984600	-2.48122700
H	4.35865100	-1.88101500	-1.24000000
H	4.35878300	-1.87923100	1.24100000
H	2.26822200	-1.50629400	2.48192600
H	-2.62517100	-1.01538800	2.48152400
H	-4.74832100	-0.98026300	1.24141400
H	-4.74844400	-0.98198900	-1.24075300
H	-2.62541200	-1.01884500	-2.48101800
H	-0.92669200	4.15641300	-0.88705800
H	-0.92643800	4.15611200	0.88690500
H	-0.15317800	-1.00846700	2.46595400

Imaginary Frequencies: 1

Cycloadduct 82 (ωB97X-D/def2-TZVP/SMD(THF))

C	0.09440100	-0.09829600	-1.31065900
C	-0.89809000	-1.07730900	-0.69983800
C	-0.89812700	-1.07727600	0.69982500
C	-1.75833000	-1.90402000	1.39409200
C	-2.62504000	-2.74002300	0.69254200
C	-2.62499600	-2.74006400	-0.69256100
C	-1.75824800	-1.90409400	-1.39410900
C	-0.30744100	1.26333400	-0.75796800
C	0.56314600	2.24085300	-0.00007300
H	1.63872900	2.14221600	-0.00015400
C	-0.30736400	1.26337100	0.75790000
C	0.09437200	-0.09827000	1.31063500
H	0.11022900	-0.15308200	2.39913700
C	1.43931600	-0.42814700	0.69944400
C	1.43932700	-0.42814100	-0.69942800
C	2.60485900	-0.68810100	-1.39438700
C	3.77624200	-0.96037600	-0.69310100
C	3.77622800	-0.96038300	0.69317300
C	2.60482600	-0.68811900	1.39442500
C	-1.72209600	1.87337400	0.75720500
H	-2.51744600	1.67775000	1.46088700
C	-1.54701800	3.16180400	0.00000000
H	-2.35887000	3.87577100	-0.00007600
C	-0.09976000	3.59566300	-0.00003800
C	-1.72213800	1.87329600	-0.75710400
H	-2.51779200	1.67789500	-1.46051400
H	-1.75877200	-1.90159900	2.47840000
H	-3.30066400	-3.39113500	1.23392800
H	-3.30058600	-3.39121200	-1.23394900
H	-1.75862500	-1.90173100	-2.47841600
H	2.60474600	-0.68107000	-2.47841000
H	4.69034500	-1.17312900	-1.23397300
H	4.69031600	-1.17315700	1.23406900
H	2.60468000	-0.68112600	2.47844900
H	0.15952300	4.17612200	0.88832100
H	0.15946900	4.17614200	-0.88840100
H	0.11031000	-0.15312000	-2.39915700

Imaginary Frequencies: 0

ethane (ωB97X-D/def2-TZVP)

C	0.00000000	0.00000000	0.76147900
H	0.00000000	1.01723000	1.15739800
H	0.88094700	-0.50861500	1.15739800
H	-0.88094700	-0.50861500	1.15739800

C	0.00000000	0.00000000	-0.76147900
H	0.88094700	0.50861500	-1.15739800
H	0.00000000	-1.01723000	-1.15739800
H	-0.88094700	0.50861500	-1.15739800

Imaginary Frequencies: 0

***n*-butane** (ωB97X-D/def2-TZVP)

C	-0.70100300	1.82190500	0.00000000
H	-0.18750400	2.21318500	0.88163100
H	-1.71613100	2.22263300	0.00000000
H	-0.18750400	2.21318500	-0.88163100
C	-0.70100300	0.29902400	0.00000000
H	-1.24719400	-0.06816800	0.87505200
H	-1.24719400	-0.06816800	-0.87505200
C	0.70100300	-0.29902400	0.00000000
H	1.24719400	0.06816800	0.87505200
H	1.24719400	0.06816800	-0.87505200
C	0.70100300	-1.82190500	0.00000000
H	0.18750400	-2.21318500	-0.88163100
H	1.71613100	-2.22263300	0.00000000
H	0.18750400	-2.21318500	0.88163100

Imaginary Frequencies: 0

***n*-pentane** (ωB97X-D/def2-TZVP)

C	-2.54131300	-0.32381400	-0.00002200
H	-2.58137300	-0.96815700	-0.88161800
H	-3.43840000	0.29776400	-0.00002300
H	-2.58139800	-0.96818900	0.88155100
C	-1.27485800	0.52199400	0.00001900
H	-1.27345300	1.17984600	-0.87504800
H	-1.27348400	1.17982100	0.87510700
C	0.00000000	-0.31264100	0.00002900
H	-0.00001000	-0.97227100	-0.87548200
H	0.00001000	-0.97221800	0.87558100
C	1.27485800	0.52199400	-0.00001800
H	1.27347800	1.17986500	0.87503600
H	1.27345900	1.17980200	-0.87511900
C	2.54131300	-0.32381400	-0.00000500
H	2.58136100	-0.96822300	-0.88155500
H	3.43840000	0.29776400	-0.00006600
H	2.58141000	-0.96812300	0.88161500

Imaginary Frequencies: 0

Ethylene 1–Bent

1a

C	0.0000000	0.0000000	0.6607030
C	0.0000000	0.0000000	-0.6607030
H	0.0000000	0.5471610	1.5957920
H	0.0000000	-0.5471610	1.5957920
H	0.0000000	-0.5471610	-1.5957920
H	0.0000000	0.5471610	-1.5957920

1q

C	0.0000000	0.0000000	0.6607030
C	0.0000000	0.0000000	-0.6607030
H	0.0000000	0.6110570	1.5553460
H	0.0000000	-0.6110570	1.5553460
H	0.0000000	-0.6110570	-1.5553460
H	0.0000000	0.6110570	-1.5553460

1p

C	0.0000000	0.0000000	0.6607030
C	0.0000000	0.0000000	-0.6607030
H	0.0000000	0.6719750	1.5105420
H	0.0000000	-0.6719750	1.5105420
H	0.0000000	-0.6719750	-1.5105420
H	0.0000000	0.6719750	-1.5105420

1o

C	0.0000000	0.0000000	0.6607030
C	0.0000000	0.0000000	-0.6607030
H	0.0000000	0.7296200	1.4615970
H	0.0000000	-0.7296200	1.4615970
H	0.0000000	-0.7296200	-1.4615970
H	0.0000000	0.7296200	-1.4615970

1n

C	0.0000000	0.0000000	0.6607030
C	0.0000000	0.0000000	-0.6607030
H	0.0000000	0.7837100	1.4087500
H	0.0000000	-0.7837100	1.4087500
H	0.0000000	-0.7837100	-1.4087500
H	0.0000000	0.7837100	-1.4087500

1m

C	0.0000000	0.0000000	0.6607030
C	0.0000000	0.0000000	-0.6607030
H	0.0000000	0.8339820	1.3522590
H	0.0000000	-0.8339820	1.3522590
H	0.0000000	-0.8339820	-1.3522590
H	0.0000000	0.8339820	-1.3522590

1l

C	0.0000000	0.0000000	0.6607030
C	0.0000000	0.0000000	-0.6607030
H	0.0000000	0.8801910	1.2923990
H	0.0000000	-0.8801910	1.2923990
H	0.0000000	-0.8801910	-1.2923990
H	0.0000000	0.8801910	-1.2923990

1–eq

C	0.0000000	0.0000000	0.6607030
C	0.0000000	0.0000000	-0.6607030
H	0.0000000	0.9221120	1.2294610
H	0.0000000	-0.9221120	1.2294610
H	0.0000000	-0.9221120	-1.2294610
H	0.0000000	0.9221120	-1.2294610

1k

C	0.0000000	0.0000000	0.6607030
C	0.0000000	0.0000000	-0.6607030
H	0.0000000	0.9595400	1.1637520
H	0.0000000	-0.9595400	1.1637520
H	0.0000000	-0.9595400	-1.1637520
H	0.0000000	0.9595400	-1.1637520

1j

C	0.0000000	0.0000000	0.6607030
C	0.0000000	0.0000000	-0.6607030
H	0.0000000	0.9922940	1.0955930
H	0.0000000	-0.9922940	1.0955930
H	0.0000000	-0.9922940	-1.0955930
H	0.0000000	0.9922940	-1.0955930

1i

C	0.0000000	0.0000000	0.6607030
C	0.0000000	0.0000000	-0.6607030
H	0.0000000	1.0202130	1.0253140
H	0.0000000	-1.0202130	1.0253140
H	0.0000000	-1.0202130	-1.0253140
H	0.0000000	1.0202130	-1.0253140

1h

C	0.0000000	0.0000000	0.6607030
C	0.0000000	0.0000000	-0.6607030
H	0.0000000	1.0431620	0.9532600
H	0.0000000	-1.0431620	0.9532600
H	0.0000000	-1.0431620	-0.9532600
H	0.0000000	1.0431620	-0.9532600

1g

C	0.0000000	0.0000000	0.6607030
C	0.0000000	0.0000000	-0.6607030
H	0.0000000	1.0610290	0.8797800
H	0.0000000	-1.0610290	0.8797800
H	0.0000000	-1.0610290	-0.8797800
H	0.0000000	1.0610290	-0.8797800

1f

C	0.0000000	0.0000000	0.6607030
C	0.0000000	0.0000000	-0.6607030
H	0.0000000	1.0737260	0.8052330
H	0.0000000	-1.0737260	0.8052330
H	0.0000000	-1.0737260	-0.8052330
H	0.0000000	1.0737260	-0.8052330

1b

C	0.0000000	0.0000000	0.6607030
C	0.0000000	0.0000000	-0.6607030
H	0.0000000	1.0811920	0.7299810
H	0.0000000	-1.0811920	0.7299810
H	0.0000000	-1.0811920	-0.7299810
H	0.0000000	1.0811920	-0.7299810

1–Pyramidalized

1c

C	0.0000000	0.0000000	0.6607030
C	0.0000000	0.0000000	-0.6607030
H	0.0000000	0.9382600	1.2024080
H	0.0000000	-0.9382600	1.2024080
H	0.0000000	-0.9382600	-1.2024080
H	0.0000000	0.9382600	-1.2024080

1r

C	0.0000000	0.6607030	-0.0352510
C	0.0000000	-0.6607030	-0.0352510
H	0.9346950	1.1900800	0.1057530
H	-0.9346950	1.1900800	0.1057530
H	0.9346950	-1.1900800	0.1057530
H	-0.9346950	-1.1900800	0.1057530

1s

C	0.0000000	0.6607030	0.0700550
C	0.0000000	-0.6607030	0.0700550
H	-0.9237590	1.1525610	-0.2101640
H	0.9237590	1.1525610	-0.2101640
H	0.9237590	-1.1525610	-0.2101640
H	-0.9237590	-1.1525610	-0.2101640

1t

C	0.0000000	0.6607030	0.0981060
C	0.0000000	-0.6607030	0.0981060
H	-0.9086240	1.1013650	-0.2943170
H	0.9086240	1.1013650	-0.2943170
H	0.9086240	-1.1013650	-0.2943170
H	-0.9086240	-1.1013650	-0.2943170

1u

C	0.0000000	0.6607030	0.1188020
C	0.0000000	-0.6607030	0.1188020
H	-0.8928660	1.0489620	-0.3564050
H	0.8928660	1.0489620	-0.3564050
H	0.8928660	-1.0489620	-0.3564050
H	-0.8928660	-1.0489620	-0.3564050

1d

C	0.0000000	0.6607030	0.1354260
C	0.0000000	-0.6607030	0.1354260
H	-0.8764970	0.9954950	-0.4062790
H	0.8764970	0.9954950	-0.4062790
H	0.8764970	-0.9954950	-0.4062790
H	-0.8764970	-0.9954950	-0.4062790

1v

C	0.0000000	0.6607030	0.1492400
C	0.0000000	-0.6607030	0.1492400
H	-0.8595270	0.9411100	-0.4477210
H	0.8595270	0.9411100	-0.4477210
H	0.8595270	-0.9411100	-0.4477210
H	-0.8595270	-0.9411100	-0.4477210

1w

C	0.0000000	0.6607030	0.1608820
C	0.0000000	-0.6607030	0.1608820
H	-0.8419670	0.8859560	-0.4826460
H	0.8419670	0.8859560	-0.4826460
H	0.8419670	-0.8859560	-0.4826460
H	-0.8419670	-0.8859560	-0.4826460

1x

C	0.0000000	0.6607030	0.1707250
C	0.0000000	-0.6607030	0.1707250
H	-0.8238310	0.8301860	-0.5121760
H	0.8238310	0.8301860	-0.5121760
H	0.8238310	-0.8301860	-0.5121760
H	-0.8238310	-0.8301860	-0.5121760

1y

C	0.0000000	0.6607030	0.1790110
C	0.0000000	-0.6607030	0.1790110
H	-0.8051300	0.7739500	-0.5370320
H	0.8051300	0.7739500	-0.5370320
H	0.8051300	-0.7739500	-0.5370320
H	-0.8051300	-0.7739500	-0.5370320

1z

C	0.0000000	0.6607030	0.1859030
C	0.0000000	-0.6607030	0.1859030
H	-0.7858780	0.7174040	-0.5577090
H	0.7858780	0.7174040	-0.5577090
H	0.7858780	-0.7174040	-0.5577090
H	-0.7858780	-0.7174040	-0.5577090

1aa

C	0.0000000	0.6607030	0.1915220
C	0.0000000	-0.6607030	0.1915220
H	-0.7660860	0.6607030	-0.5745650
H	0.7660860	0.6607030	-0.5745650
H	0.7660860	-0.6607030	-0.5745650
H	-0.7660860	-0.6607030	-0.5745650

1ab

C	0.0000000	0.6607030	0.1959570
C	0.0000000	-0.6607030	0.1959570
H	-0.7457700	0.6040020	-0.5878720
H	0.7457700	0.6040020	-0.5878720
H	0.7457700	-0.6040020	-0.5878720
H	-0.7457700	-0.6040020	-0.5878720

1e

C	0.0000000	0.6607030	0.1992820
C	0.0000000	-0.6607030	0.1992820
H	-0.7249430	0.5474560	-0.5978450
H	0.7249430	0.5474560	-0.5978450
H	0.7249430	-0.5474560	-0.5978450
H	-0.7249430	-0.5474560	-0.5978450

1–Length

1ac

C	0.0000000	0.0000000	0.5807030
H	0.0000000	0.9221120	1.1494610
H	0.0000000	-0.9221120	1.1494610
C	0.0000000	0.0000000	-0.5807030
H	0.0000000	-0.9221120	-1.1494610

H	0.0000000	0.9221120	-1.1494610

1ad

C	0.0000000	0.0000000	0.6007030
H	0.0000000	0.9221120	1.1694610
H	0.0000000	-0.9221120	1.1694610
C	0.0000000	0.0000000	-0.6007030
H	0.0000000	-0.9221120	-1.1694610
H	0.0000000	0.9221120	-1.1694610

1ae

C	0.0000000	0.0000000	0.6207030
H	0.0000000	0.9221120	1.1894610
H	0.0000000	-0.9221120	1.1894610
C	0.0000000	0.0000000	-0.6207030
H	0.0000000	-0.9221120	-1.1894610
H	0.0000000	0.9221120	-1.1894610

1af

C	0.0000000	0.0000000	0.6407030
H	0.0000000	0.9221120	1.2094610
H	0.0000000	-0.9221120	1.2094610
C	0.0000000	0.0000000	-0.6407030
H	0.0000000	-0.9221120	-1.2094610
H	0.0000000	0.9221120	-1.2094610

1ag

C	0.0000000	0.0000000	0.6807030
H	0.0000000	0.9221120	1.2494610
H	0.0000000	-0.9221120	1.2494610
C	0.0000000	0.0000000	-0.6807030
H	0.0000000	-0.9221120	-1.2494610
H	0.0000000	0.9221120	-1.2494610

1ah

C	0.0000000	0.0000000	0.7007030
H	0.0000000	0.9221120	1.2694610
H	0.0000000	-0.9221120	1.2694610
C	0.0000000	0.0000000	-0.7007030
H	0.0000000	-0.9221120	-1.2694610
H	0.0000000	0.9221120	-1.2694610

1ai

C	0.0000000	0.0000000	0.7207030
H	0.0000000	0.9221120	1.2894610
H	0.0000000	-0.9221120	1.2894610
C	0.0000000	0.0000000	-0.7207030
H	0.0000000	-0.9221120	-1.2894610
H	0.0000000	0.9221120	-1.2894610

1aj

C	0.0000000	0.0000000	0.7407030
H	0.0000000	0.9221120	1.3094610
H	0.0000000	-0.9221120	1.3094610
C	0.0000000	0.0000000	-0.7407030
H	0.0000000	-0.9221120	-1.3094610
H	0.0000000	0.9221120	-1.3094610

1ak

C	0.0000000	0.0000000	0.7607030
H	0.0000000	0.9221120	1.3294610
H	0.0000000	-0.9221120	1.3294610
C	0.0000000	0.0000000	-0.7607030
H	0.0000000	-0.9221120	-1.3294610
H	0.0000000	0.9221120	-1.3294610

1al

C	0.0000000	0.0000000	0.7807030
H	0.0000000	0.9221120	1.3494610
H	0.0000000	-0.9221120	1.3494610
C	0.0000000	0.0000000	-0.7807030
H	0.0000000	-0.9221120	-1.3494610
H	0.0000000	0.9221120	-1.3494610

1am

C	0.0000000	0.0000000	0.8007030
H	0.0000000	0.9221120	1.3694610
H	0.0000000	-0.9221120	1.3694610
C	0.0000000	0.0000000	-0.8007030
H	0.0000000	-0.9221120	-1.3694610
H	0.0000000	0.9221120	-1.3694610

1an

C	0.0000000	0.0000000	0.8207030
H	0.0000000	0.9221120	1.3894610
H	0.0000000	-0.9221120	1.3894610
C	0.0000000	0.0000000	-0.8207030
H	0.0000000	-0.9221120	-1.3894610
H	0.0000000	0.9221120	-1.3894610

References:

[1] Houston, S. D. et al. The cubane paradigm in bioactive molecule discovery. *Org. Biomol. Chem.* **17**, 6790–6798 (2019).

[2] Lai, K. & Salomon, R. G. 6-Substituted bicyclo[2.2.1]hept-5-en-2-one ketals. *J. Org. Chem.* **54**, 2628–2632 (1989).

[3] Khrizman, A. et al. Synthesis and *in vitro* protozoocidal evalution of novel diazabicyclic tropolone derivatives. *Arch. Pharm. Chem. Life Sci.* **340**, 569–576 (2007).

[4] Kalaitzakis, D., Sofiadis, M., Tsopanakis, V., Montagnon, T. & Vassilikogiannakis, G. Merging singlet-oxygen induced furan oxidations with organocatalysis: synthesis of enantiopure cyclopentanones and hydrindanes. *Org. Biomol. Chem.* **18**, 2817–2822 (2020).

[5] Mandal, A. B. et al. One-step synthesis and highly regio- and stereoselective Diels-Alder cycloadditions of novel exo-2-oxazoidinone dienes. *J. Org. Chem.* **62**, 4105–4115 (1997).

[6] Hirao, Y., Saito, T., Kurata, H. & Kubo, T. Isolation of a hydrogen-bonded complex based on the anthranol/anthroxyl pair: formation of a hydrogen-atom self-exchange system. *Angew. Chem., Int. Ed.* **54**, 2402–2405 (2015).

[7] Sabat, N. et al. Gold-catalyzed spirocyclization reactions of *N*-propargyl tryptamines and tryptophans in aqueous media. *Org. Lett.* **22**, 4344–4349 (2020).

[8] Gaoni, Y. & Sadeh, S. Preparation and selected reactions of 2,3-bis(bromomethyl)-1,3-butadiene. *J. Org. Chem.* **45**, 870–881 (1980).

[9] Lukin, K. & Eaton, P. E. Dimerization of cubene. 1-Iodoadamantane as a probe for radical intermediates. *J. Am. Chem. Soc.* **117**, 7652–7656 (1995).

[10] He, S. -J. et al. Phosphoric acid-catalyzed enantioselective synthesis of axially chiral anthrone-based compounds. *Angew. Chem., Int. Ed.* **62**, e202213914 (2023).

[11] Majumdar, K. C., Chattopadhyay, S. K. & Khan, A. T. Phase-transfer-catalyzed alkylation of anthrone and 10-propargylanthrone. *Synthesis* **7**, 552–553 (1988).

[12] Bruker, SAINT, V8.41, Bruker AXS Inc., Madison, Wisconsin, USA.

[13] Krause, L., Herbst-Irmer, R., Sheldrick, G. M. & Stalke, D. Comparison of silver and molybdenum microfocus X-ray sources for single-crystal structure determination. *J. Appl. Cryst.* **48**, 3–10 (*2015*).

[14] Sheldrick, G. M. Crystal structure refinement with SHELXL. *Acta Cryst.* **A71**, 3–8 (2015).

[15] Sheldrick, G. M. SHELXT – Integrated space-group and crystal structure determination. *Acta Cryst.* **C71**, 3–8 (2015).

[16] Kratzert, D. *FinalCif*, *V151*, https://dkratzert.de/finalcif.html.

[17] Baumgärtel, O. et al. Diels-Alder-addukte des 1,7-dehydroquadricyclans. *Chem. Ber*. **116**, 2205–2218 (1983).

[18] Kabsch, W. XDS *Acta Cryst.* **D66**, 125–132 (2010).

[19] Peng, L.-M. Electron atomic scattering factors and scattering potentials of crystals. Micron 30 625–648 (1999).

[20] Spartan'24 Wavefunction, Inc., Irvine, CA.

[21] Frisch, M. J., Trucks, G. W., Schlegel, H. B., Scuseria, G. E., Robb, M. A., Cheeseman, J. R., Scalmani, G., Barone, V., Petersson, G. A., Nakatsuji, H., Li, X., Caricato, M., Marenich, A. V., Bloino, J., Janesko, B. G., Gomperts, R.m Mennucci, B., Hratchian, H. P., Ortiz, J. V., Izmaylov, A. F., Sonnenberg, J. L., Williams-Young, D., Ding, F., Lipparini, F., Egidi, F., Goings, J., Peng, B., Petrone, A., Henderson, T., Ranasinghe, D., Zakrzewski, V. G., Gao, J., Rega, N., Zheng, G., Liang, W., Hada, M., Ehara, M., Toyota, K., Fukuda, R., Hasegawa, J., Ishida, M., Nakajima, T., Honda, Y., Kitao, O., Nakai, H., Vreven, T., Throssell, K., Montgomery, Jr., J. A., Peralta, J. E., Ogliaro, F., Bearpark, M. J., Heyd, J. J., Brothers, E. N., Kudin, K. N., Staroverov, V. N., Keith, T. A., Kobayashi, R., Normand, J., Raghavachari, K., Rendell, A. P., Burant, J. C., Iyengar, S. S., Generation and Reactivity of Unsymmetrical Strained Heterocyclic Allenes Kelleghan et al. Supplementary Information– S111 S-111 Tomasi, J., Cossi, M., Millam, J. M., Klene, M., Adamo, C., Cammi, R., Ochterski, J. W., Martin, R. L., Morokuma, K., Farkas, O., Foresman, J. B. & Fox, D. J. Gaussian 16 Revision A.03 (Gaussian, Inc., 2016).

[22] Roos, B. O., Taylor, P. R. & Sigbahn, P. E. A complete active space SCF method (CASSCF) using a density matrix formulated super-CI approach. *Chem. Phys.* **48**, 157−173 (1980).

[23] Kollmar, C., Sivalingam, K., Helmich-Paris, B., Angeli, C. & Neese, F. A perturbation-based super-CI approach for the orbital optimization of a CASSCF wave function. *J. Comput. Chem.* **40**, 1463–1470 (2019).

[24] Andersson, K., Malmqvist, P. A., Roos, B. O., Sadlej, A. J. & Wolinski, K. Second-order perturbation theory with a CASSCF reference function. *J. Phys. Chem.* **94**, 5483−5488 (1990).

[25] Andersson, K., Malmqvist, P. Å. & Roos, B. O. Second-order perturbation theory with a consistent field reference function. *J. Chem. Phys.* **96**, 1218−1226 (1992).

[26] Neese, F. Software update: the ORCA program system, version 5.0. *WIRES Comput. Mol. Sci.* **12**, e1606 (2022).

[27] Chai, J. -D. & Head-Gordon, M. Long-range corrected hybrid density functionals with damped atom-atom dispersion corrections. *Phys. Chem. Chem. Phys.* **10**, 6615–6620 (2008).

[28] Weigend, F. & Ahlrichs, A. Balanced basis sets of split valence, triple zeta valence and quadruple zeta valence quality for H to Rn: Design and assessment of accuracy. *Phys. Chem. Chem. Phys.* **7**, 3297–3305 (2005).

[29] Luchini, G., Alegre-Requena, J. V., Funes-Ardoiz, I. & Paton, R. S. GoodVibes: Automated thermochemistry for heterogeneous computational chemistry data. *F1000Research* **9**, 291 (2020).

[30] Grimme, S. Supramolecular binding thermodynamics by dispersion-corrected density functional theory. *Chem. Eur. J.* **18**, 9955–9964 (2012).

[31] Li, Y., Gomes, J., Sharada, S. M., Bell, A. T. & Head-Gordon, M. Improved force-field parameters for QM/MM simulations of the energies of adsorption for molecules in zeolites and a free rotor correction to the rigid rotor harmonic oscillator model for adsorption enthalpies. *J. Phys. Chem. C* **119**, 1840–1850 (2015).

[32] Raghavachari, K., Trucks, G. W., Pople, J. A. & Head-Gordon, M. A fifth-order perturbation comparison of electron correlation theories. *Chem. Phys. Lett.* **157**, 479–483 (1989).

[33] Dunning, T. H. Jr. Gaussian basis sets for use in correlated molecular calculations. I. The atoms boron through neon and hydrogen. *J. Chem. Phys.* **90**, 1007–1023 (1989).

[34] Mayer, I. Charge, bond order and valence in the AB initio SCF theory. *Chem. Phys. Lett.* **97**, 270–274 (1983).

[35] Glendening, E. D., Reed, A. E., Carpenter, J. E. & Weinhold, F. *NBO*, Version 3.1.

[36] Lu, T. & Chen, F. Multiwfn: A multifunctional wavefunction analyzer. *J. Comput. Chem.* **33**, 580–592 (2012).

[37] Lu, T. A comprehensive electron wavefunction analysis toolbox for chemists, Multiwfn. *J. Chem. Phys.* **161**, 082503 (2024).

[38] Fernández, J. A. & Vázquez, S. A theoretical study of tricyclo[4.2.1.0^{2,5}]non-2(5)-ene, tricyclo[4.2.2.0^{2,5}]dec-2(5)-ene and relate pyramidalized alkenes. *Eur. J. Org. Chem.* **2007**, 4493–4498 (2007).

[39] Haddon, R. C. Comment on the relationship of the pyramidalization angle at a conjugated carbon atom to the σ bond angles. *J. Phys. Chem. A* **105**, 4164–4165 (2001).

[40] Volland, W. V., Davidson, E. R. & Borden, W. T. Effect of carbon atom pyramidalization on the bonding in ethylene. *J. Am. Chem. Soc.* **101**, 533–537 (1979).

[41] McDermott, L. et al. A solution to the anti-Bredt olefin synthesis problem. *Science* **386**, eadq3519 (2024).

[42] Hrovat, D. A. & Borden, W. T. Ab initio calculations of the relative energies of 1,2-, 1,3-, and 1,4-dehydrocubane: prediction of dominant through-bond interaction in 1,4-dehydrocubane. *J. Am. Chem. Soc.* **112**, 875–876 (1990).

[43] Podlech, J., Polborn, K. & Szeimies, G. J. Tetracyclo[3.2.0.0^{2,7}.0^{4,6}] hept-1-ene: Formation and trapping of 1,2-dehydroquadricyclane. Ab initio calculations on dehydroquadricyclanes. *J. Org. Chem.* **58**, 4113–4117 (1993).

[44] Bridgeman, A. J., Cavigliasso, G., Ireland, L. R. & Rothery, J. Mayer bond order as a tool in inorganic chemistry. *J. Chem. Soc., Dalton Trans.* 2095–2108 (2001).

[45] Matito, E., Poater, J., Solà, M., Duran, M. & Salvador, P. Comparison of the AIM delocalization index and the Mayer and fuzzy atom bond orders. *J. Phys. Chem. A* **109**, 9904–9910 (2005).

[46] Wheeler, S. E., Houk, K. N., Schleyer, P. v. R. & Allen, W. D. A Hierarchy of homodesmotic reactions for thermochemistry. *J. Am. Chem. Soc.* **131**, 2547–2560 (2009).

[47] Maier, W. F. & Schleyer, P. v. R. Evaluation and prediction of the stability of bridgehead olefins. *J. Am. Chem. Soc.* **103**, 1891–1900 (1981).

[48] Hrovat, D. A. & Borden, W. T. Ab initio calculations of the olefin strain energies of some pyramidalized alkenes. *J. Am. Chem. Soc.* **110**, 4710–4718 (1988).

[49] Staneke, P. O. et al. Formation of the radical anion of cubene and determination of the heat of formation, heat of hydrogenation, and olefin strain energy of cubene. *J. Am. Chem. Soc.* **116**, 6445–6446 (1994).

[50] Doehnert, D. & Koutecky, J. Occupation numbers of natural orbitals as a criterion for biradical character. Different kinds of biradicals. *J. Am. Chem. Soc.* **102**, 1789–1796 (1980).

[51] Tena Meza et al. σ-Bond insertion reactions of two strained diradicaloids. *Nature* **640**, 683–690 (2025).

[52] Avogadro: an open-source molecular builder and visualization tool. Version 1.2.0.

[53] Vázquez, S. & Camps, P. Chemistry of pyramidalized alkenes. *Tetrahedron* **61**, 5147–5208 (2005).

[54] Gorelsky, S. I. Complexes with a single metal–metal bond as a sensitive probe of quality of exchange–correlation functionals. *J. Chem. Theory Comput.* **8**, 908–914 (2012).

[55] Lehtola, S. & Jónsson, H. Pipek−Mezey Orbital Localization Using Various Partial Charge Estimates. *J. Chem. Theory Comput.* **10**, 642–649 (2014).

[56] Dennington, R. D. II, Keith, T. A. & Millam, J. M. GaussView 6.0.16 (Semichem, Inc. 2000-2016).

[57] Lu, T. & Chen, F. Calculation of Molecular Orbital Composition. *Acta Chim. Sinica*, **69**, 2393–2406 (2011).

[58] Glendening, E. D., Reed, A. E., Carpenter, J. E. & Weinhold, F. *NBO*, Version 3.1.

[59] Houk, K. N. et al. Electronic origins and consequences of pyramidalization of asymmetric alkenes in ground and triplet excited states. *J. Am. Chem. Soc.* **105**, 5980–5988 (1983).

[60] Inagaki, S. Fujimoto, H. & Fukui, K. Orbital mixing rule. *J. Am. Chem. Soc.* **98**, 4054–4061 (1976).

[61] Harnisch, J. et al. Intermediacy of tetracyclo[3.2.0.02.7.04.6]hept-1(7)-ene, a highly strained bridgehead olefin. *J. Am. Chem. Soc.* **101**, 3370–3371 (1979).

[62] Mayer, I. Improved definition of bond orders for correlated wave functions. *Chem. Phys. Lett.* **544**, 83–86 (2012).
